# The Highly Regioselective Synthesis of Novel Imidazolidin-2-Ones via the Intramolecular Cyclization/Electrophilic Substitution of Urea Derivatives and the Evaluation of Their Anticancer Activity

**DOI:** 10.3390/molecules26154432

**Published:** 2021-07-22

**Authors:** Almir S. Gazizov, Andrey V. Smolobochkin, Elizaveta A. Kuznetsova, Dinara S. Abdullaeva, Alexander R. Burilov, Michail A. Pudovik, Alexandra D. Voloshina, Victor V. Syakaev, Anna P. Lyubina, Syumbelya K. Amerhanova, Julia K. Voronina

**Affiliations:** 1 Arbuzov Institute of Organic and Physical Chemistry, FRC Kazan Scientific Center, Russian Academy of Sciences, Arbuzova Str. 8, 420088 Kazan, Russia; smolobochkin@iopc.ru (A.V.S.); sherlok@live.ru (E.A.K.); burilov@iopc.ru (A.R.B.); pudovik@iopc.ru (M.A.P.); microbi@iopc.ru (A.D.V.); vvs@iopc.ru (V.V.S.); anna.l@mail.ru (A.P.L.); s.amerkhanova@mail.ru (S.K.A.); 2 Chemistry Department, Kazan Federal University, Kremlyovskaya Str. 18, 420008 Kazan, Russia; dinka0902@mail.ru; 3 N.S. Kurnakov Institute of General and Inorganic Chemistry, Russian Academy of Sciences, Leninsky Ave. 31, 119991 Moscow, Russia; juliavoronina@mail.ru; 4 G.V. Plekhanov Russian University of Economics, Stremyanny Per. 36, 117997 Moscow, Russia

**Keywords:** imidazolidine-2-one, regioselectivity, urea, cyclization, anti-tumor activity, anti-cancer activity, cytotoxicity

## Abstract

A series of novel 4-(het)arylimidazoldin-2-ones were obtained by the acid-catalyzed reaction of (2,2-diethoxyethyl)ureas with aromatic and heterocyclic *C*-nucleophiles. The proposed approach to substituted imidazolidinones benefits from excellent regioselectivity, readily available starting materials and a simple procedure. The regioselectivity of the reaction was rationalized by quantum chemistry calculations and control experiments. The anti-cancer activity of the obtained compounds was tested in vitro.

## 1. Introduction

**Figure 1:**
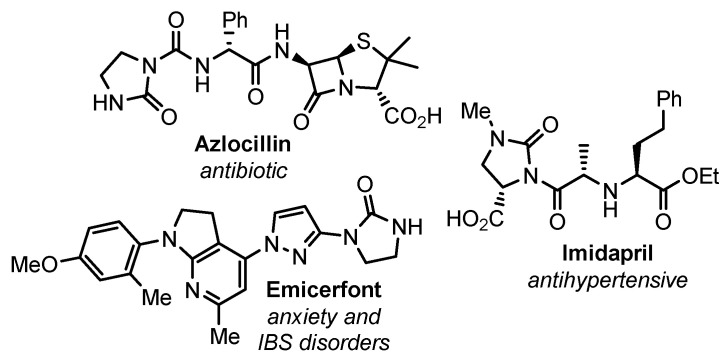
Some known drugs possessing imidazolidin-2-one moiety.

Various cyclic ureas are the well-established pivotal functionalities in the drug discovery and medicinal chemistry. Among them, the imidazolidine-2-one scaffold is often found in bioactive compounds and constitute the key structural part of a plethora of FDA approved drugs, such as emicerfont, imidapril, azlocillin and others (Figure 1). Moreover, these compounds may serve as precursors for vicinal diamines, which are valuable building blocks in organic and medicinal chemistry [1,2]. Taking these into account, it is not surprising that a lot of effort is devoted to the development of the methods of synthesis of imidazolidinones [3].

The most straightforward approach to cyclic ureas is the carbonylation of variously substituted diamines. This approach is somewhat controversial due to the abovementioned utility of imidazolidine-2-ones as 1,2-diamines precursors. However, it is still used in some cases, especially with chiral diamines [4,5,6,7]. Novel approaches to these compounds have been developed in 2019–2020 by various research groups, indicating a growing area of interest. These include silver-catalyzed cycloaddition of nitrones with methylene isocyanides [8], stereoselective diamination of alkenes with 1,3-ditosylurea [9] and Rh-catalyzed intramolecular C–H amination of *N*-oxyurea derivatives [10,11]. Pd-catalyzed amidation of vinylethylcarbamates followed by cyclization of intermediate allyl ureas should also be mentioned [12]. Finally, the amidoalkylation of indoles by hydroxyimidazolidin-2-one under the Lewis acid catalysis has also been reported [13].

**Scheme 1:**
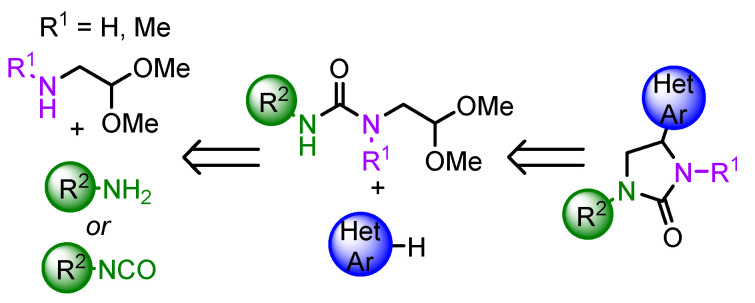
The proposed synthetic approach to 4-(hetero) arylimidazolidine-2-ones.

Nitrogen-containing aldehydes and acetals are widely used in the heterocycles synthesis [14,15]. Earlier, we have described the approach to 2-substituted pyrrolidine derivatives based on the in situ generation of a cyclic iminium ion from 4-aminobutanal acetals (4,4-diethoxybutan-1-amines) and its trapping by various nucleophiles [16,17,18]. Our preliminary studies indicate that *N*-(2,2-dialkoxyethyl) ureas are also capable of the formation of cyclic imidazolinium cations and the reaction results in cyclic urea derivatives—imidazolidinones or benzo [*d*] [1,3] diazepinones [19]. As the continuation of our efforts, herein, we report the successful application of this methodology to the synthesis of the series of 4-(hetero) arylimidazolidin-2-ones starting from *N*-(2,2-dialkoxyethyl) ureas and (hetero) aromatic nucleophiles (Scheme 1). The main advantages of the described method are the mild reaction conditions, good to high product yield and the high regioselectivity. Notably, *N*-(2,2-dialkoxyethyl) ureas are easily accessible from commercially available 2,2-dimethoxyethan-1-amine and amines or isocyanates in just one step, which also adds value to the proposed approach. Some considerations on the regioselectivity of the reaction are discussed based on quantum chemistry calculations, as well as evaluation of the in vitro anti-cancer activities of the obtained compounds.

## 2. Results and Discussion

### 2.1. Chemistry

**Scheme 2:**
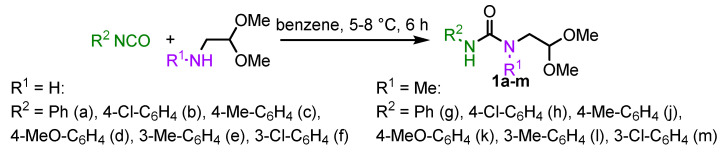
The synthesis of starting *N*-(2,2-dialkoxyethyl)ureas **1.**

We initiated our studies with the expanding the scope of the starting *N*-(2,2-dialkoxyethyl) ureas **1** (Scheme 2). Di- and trisubstituted ureas possessing various aryl (**1a**–**f**) and aryl/alkyl (**1g**–**m**) groups were easily obtained from appropriate isocyanates by known procedures [20].

**Scheme 3:**
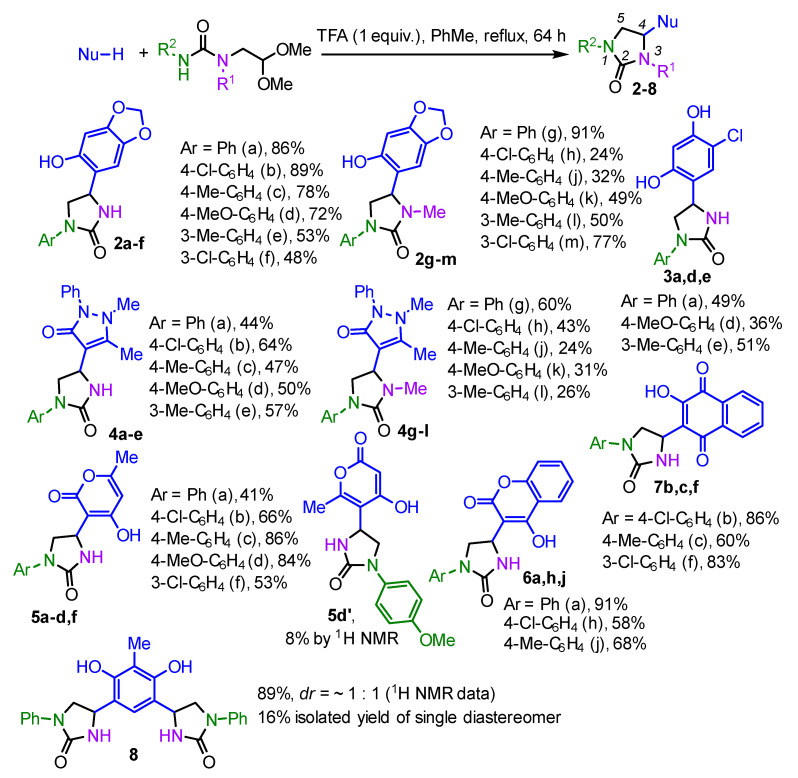
The synthesis of imidazolidine-2-ones 2. The isolated yields are given, unless noted otherwise.

Next, we investigated the reaction of the ureas **1a**–**m** with various electron-rich aromatic and heterocyclic *C*-nucleophiles (Scheme 3). In all cases, the reaction provided the desired imidazolidinones **2** in good to high yield. The previously reported conditions were employed, i.e., refluxing toluene and a triflouroacetic acid (TFA) as catalysts [19]. Notably, the 15-fold excess of catalyst was used in these preliminary experiments. However, the further screening of reaction conditions allowed us to decrease the amount of catalyst significantly without a loss of yield. Moreover, during our preliminary studies, we found that this reaction leads to the mixture of 4- and 5-substituted imidazolidin-2-ones, with 4-substituted regioisomer **2** being the major one. Decreasing the amount of TFA led to the improved regioselectivity, so that only 4-substituted imidazolinones **2** were observed in the reaction mixture. The isolated yields of target compounds varied somewhat. In general, ureas possessing unsubstituted nitrogen atom tend to provide higher yields of imidazolidinones **2**. However, no correlation was observed between the yields and the substituents in the aryl moiety of the starting ureas.

**Figure 2:**
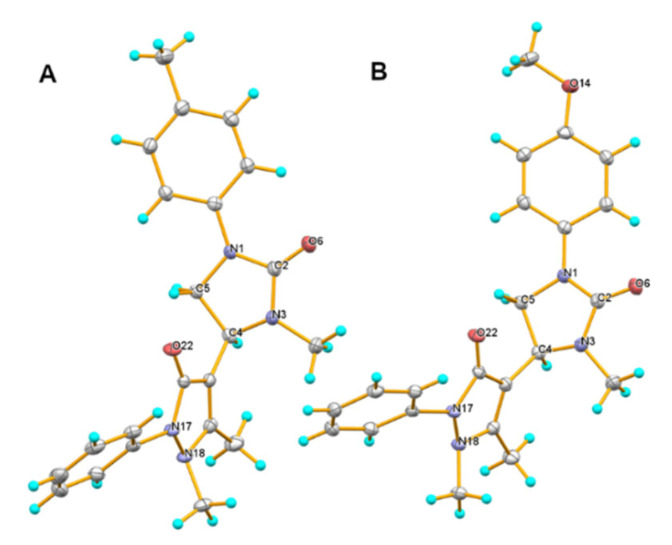
Molecular structure of the compounds **2j** (**A**) and **2k** (**B**). Ellipsoids are given with a 50% probability.

The chemical shifts of methyne and methylene protons for 4- and 5-substituted imidazolidin-2-ones differ notably [19] (see Supplementary Materials, Figure S2). Thus, the substitution site of the imidazolidinone ring for the *N*-methyl substituted compounds **2h**–**m** and **4h**–**l** was determined by the comparison of their NMR spectra with the spectra of previously described 4-substituted imidazolidin-2-ones, **2g** and **4g** [19] (for example, see Supplementary Materials, Figures S3 and S4). Additionally, the structures of the compounds **2j** and **2k** were confirmed by X-ray analysis (Figure 2; for details, see Supplementary Materials, pp. S2–S3 and Figure S1).

**Figure 3:**
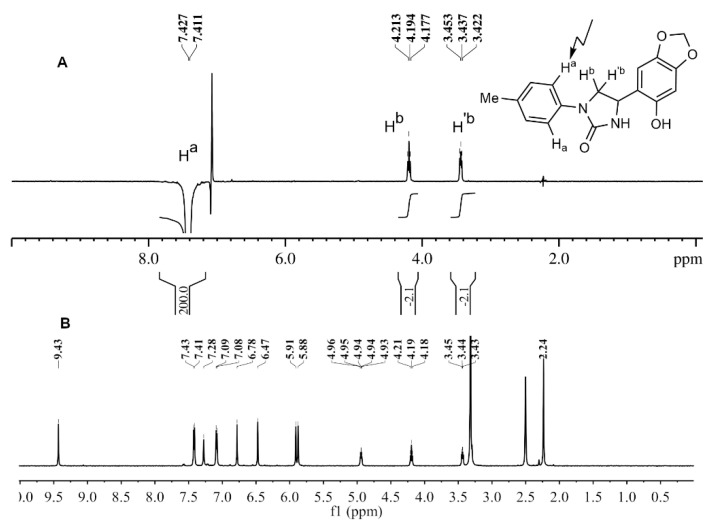
(**A**) 1D NOESY spectrum of compound **2c** (irradiation of *ortho*-aromatic protons resonance) (500 MHz, (CD_3_)_2_SO, 303 K); (**B**) ^1^H NMR spectrum of compound **2c** (400 MHz, (CD_3_)_2_SO, 303 K).

In order to determine the substitution pattern on imidazolidine-2-one ring correctly for other compounds, a complete structure elucidation of the compound **2c** was accomplished by a variety of 1D/2D NMR correlation experiments (^1^H-^1^H COSY, NOESY, ^1^H-^13^C HSQC, ^1^H-^13^C HMBC, ^1^H-^15^N HMBC). First, the signals of protons and carbon atoms were assigned used the set of 2D homo- and heteronuclear correlation experiments. Then, the 1D NOESY experiment was carried out. Selective excitation of *ortho*-protons (H^a^) of aryl fragment provided NOE between these protons and H^b^, H^’b^ protons of the methylene group (H^b^ 2.0%, H^’b^ 2.1%), which indicates the 4-substitution of imidazolidin-2-one ring (Figure 3).

**Figure 4:**
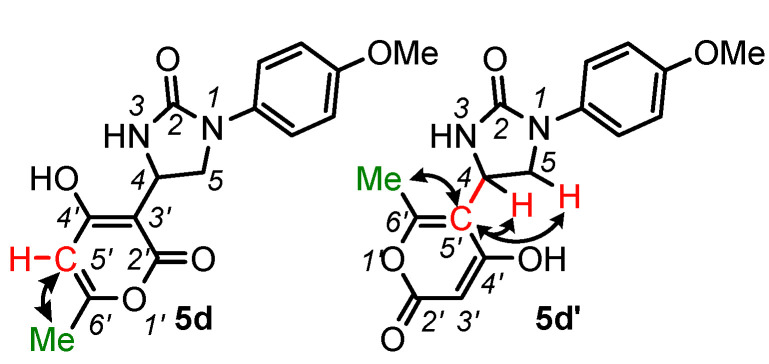
Structure determination of the compounds **5d** and **5d′**. Key cross-peaks in the ^1^H-^13^C HMBC spectra are indicated by arrows.

Interestingly, the reaction of the urea **1d** with 4-hydroxy-6-methylpyran-2-one resulted in the mixture of regioisomeric 4-(2-oxopyran-3-yl)- and 4-(2-oxopyran-5-yl)imidazolidinones **5d** and **5d′** (~10:1 molar ratio according to NMR data). The structure of regioisomers was unequivocally determined by ^1^H-^13^C HMBC experiments (Figure 4). Thus, in compound **5d**, a cross-peak is observed between the protons of the methyl group of the pyran-2-one fragment and 5′-C carbon atom, which is bonded to hydrogen. At the same time, in the compound **5d′**, the cross-peak between the protons of the same methyl group and the substituted 5′-C carbon atom is present (see Supplementary Materials, Figure S98, for the HMBC spectrum). Additionally, there are cross-peaks between the 5′-C carbon atom and protons of the methyne (4-CH) and methylene (5-CH_2_) groups of the imidazolidin-2-one ring.

The reaction of urea **1a** with 2-methylesorcinol possessing two reactive sites resulted in the disubstituted imidazolinone **8**. The compound **8** was obtained as the mixture of the (*R,S*)- and (*S,S*)-, (*R,R*)-diastereomers (*dr* = 1:1 according to NMR data), from which one diastereomer was isolated in the individual form in 16% yield. Unfortunately, we were not able to obtain the crystals suitable for the X-ray analysis; thus, the absolute configuration of the isolated compound could not be determined.

### 2.2. Mechanism Discussion and Quantum Chemistry Calculations

**Scheme 4:**
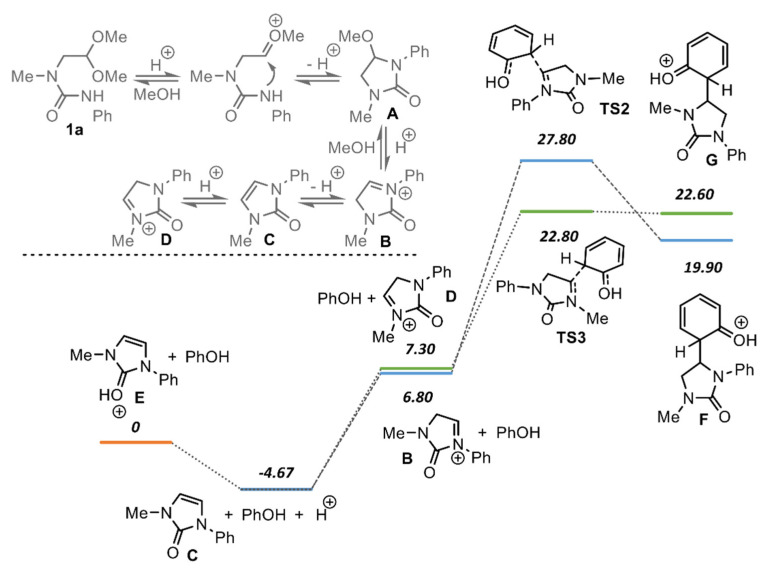
Plausible mechanism for the formation of regioisomeric imidazolidin-2-ones and relative energies of the intermediates (kcal/mol), as obtained from quantum chemistry calculations (B3LYP/6-311++G(d,p), Gaussian16).

The proposed mechanism of the formation of imidazolidine-2-ones from ureas **1** is depicted in Scheme 4 using phenol as the model nucleophile. The first stage of the reaction is the formation of the oxonium cation and its subsequent intramolecular cyclization to give 5-methoxyimidazolidine-2-one **A**. The acid-promoted elimination of the methanol molecule furnishes iminium cation **B**. This intermediate may further undergo two concurrent reactions. The first one is the interaction with phenol molecule, which results in the intermediate **F** via transition state **TS2**. Further deprotonation of this intermediate results in the 5-substituted imidazolidine-2-one. Alternatively, iminium cation **B** may undergo deprotonation-protonation steps to give isomeric cation **D** via imidazoline-2-one **C**. The subsequent reaction of cation **D** with phenol results in the intermediate **G** via transition state **TS3**. Finally, deprotonation of this intermediate furnishes 4-substituted imidazolidine-2-ones **2**.

Quantum chemistry calculations were performed for iminium ions **B** and **D**, as well as for *O*-protonated ion **E** to justify the regioselectivity of the reaction. The relative energies of the intermediates are given in Scheme 4. The oxonium ion **E** appeared to be the most stable, which is in accordance with the preferred protonation of the ureas at the oxygen atom [21]. The energy of the iminium ion **D** is 7.3 kcal/mol higher compared to cation **E**. Surprisingly, the isomeric iminium ion **B** appeared to be somewhat lower in energy (6.8 kcal/mol).

Next, energies of the intermediates **F** and **G**, as well as energies of the appropriate transition states **TS2** and **TS3**, were calculated. *ortho*-Substitution of the phenol was modelled to reflect the presence of the hydroxy group next to the reactive center in the most of the used nucleophiles. Again, the relative energy of the intermediate **F** was lower than that of the intermediate **G**. At the same time, the relative energy of the transition state **TS3** appeared to be lower than the energy of transition state **TS2** by circa 5 kcal/mol. According to the Curtin–Hammett principle [22], the product distribution for the two rapidly interconverting intermediates depends both on their relative energies and energies of the respective transition states. Thus, the observed regioselective formation of 4-substituted imidazolidine-2-ones may be attributed to its lower activation energy.

### 2.3. Biological Studies

Some of the resulting compounds were tested for cytotoxicity against normal and cancer human cell lines at concentrations of 1–100 µM. As seen from the Table 1, most of the obtained compounds exhibited little to no cytotoxicity against both normal and cancer cells. Compounds **2h** and **2k** appeared to be the most active. The selectivity index (SI) was calculated for them as the ratio between the IC_50_ value for normal cells and the IC_50_ value for cancer cells. The cytotoxicity of the compound **2h** against the HuTu 80 cancer cell line was almost two-fold higher than the cytoxicity against normal cells (SI = 1.7). This is better than the selectivity of the reference compound Arglabin (SI = 1.2). Notably, the replacement of the methyl substituent at the nitrogen atom by hydrogen lowers the cytotoxicity against cancer cells, whereas the cytotoxicity against normal cells tends to remain the same or even increases (compare, e.g., **2h** and **2b**, **2k** and **2d** and **2j** and **2c**). Thus, it appears to be one of the key factors influencing the activity of the compounds under study.

## 3. Materials and Methods

### 3.1. Chemistry

IR spectra were recorded on a UR-20 spectrometer in a 400–3600 cm^−1^ range in KBr. ^1^H NMR spectra were recorded on a Bruker MSL 400 spectrometer (400 MHz) with respect to the signals of residual protons of deuterated solvent (CDCl_3_, DMSO-d_6_). ^13^C NMR spectra were recorded on a Bruker Avance 600 (151 MHz) spectrometer relative to signals of residual protons of deuterated solvent (CDCl_3_, DMSO-d_6_). Elemental analysis is performed on a Carlo Erba device EA 1108. The melting points are determined in glass capillaries on a Stuart SMP 10 instrument.

The X-ray diffraction data for the crystals of **2j** and **2k** were collected on a Bruker D8 Venture automatic diffractometer using graphite monochromated radiation. The structures were solved by direct methods and refined by full-matrix least-squares using the SHELXL97 [23] program. All the non-hydrogen atoms were refined with anisotropic atomic displacement parameters. All figures were made using the program OLEX2 [24]. Crystallographic data for the structure reported in this paper have been deposited with the Cambridge Crystallographic Data Center (2068964–2068965).

Crystal data for **2j**: C_22_H_24_N_4_O_2_, M = 376.45, monoclinic, space group P2_1_/n, Z = 4, a = 7.3485(4), b = 21.8110(10), c = 12.3569(6)Å, β = 104.398(2)°, V = 1918.34(17)Å^3^, ρ_calc_ = 1.303 g/cm^3^, μ =0.086 mm^−1^, 15151 reflections collected (±h, ±k, ±l), 3768 independent (Rint 0.0387) and 3013 observed reflections [I ≥ 2\s (I)], 257 refined parameters, R_1_ = 0.0457, wR_2_ = 0.1049, max. residual electron density is 0.267 (−0.231) eÅ^−3^.

Crystal data for **2k**: C_22_H_24_N_4_O_3_, M = 392.45, monoclinic, space group P2_1_/c, Z = 4, a = 7.5009(5), b = 21.8592(14), c = 12.5236(9)Å, β = 107.237(3)°, V = 1961.2(2)Å^3^, ρ_calc_ = 1.329 g/cm^3^, μ =0.091 mm^−1^, 19745 reflections collected (±h, ±k, ±l), 5225 independent (Rint 0.0637) and 3528 observed reflections [I ≥ 2\s (I)], 266 refined parameters, R_1_ = 0.0479, wR_2_ = 0.1170, max. residual electron density is 0.258 (−0.249) eÅ^−3^.

**General method for the synthesis *N*-(2,2-dimethoxyethyl)ureas 1**

To a solution of 2,2-dimethoxyethan-1-amine or 2,2-dimethoxy-N-methylethan-1-amine (11.0 mmol) in benzene (10 mL), aryl isocyanate (11.0 mmol) was added dropwise under cooling (5–8 °C). The reaction mixture was stirred for 6 h at room temperature, the solvent was removed in vacuum (20 mm Hg). The resulting bright fusible solid was dried in vacuum (1 h, 0.01 mm Hg) to a constant weight.

**1-(2,2-Dimethoxyethyl)-3-phenylurea (1a)**. Yield 93%. The spectral characteristics were in agreement with the previously published data [25].

**1-(4-Chlorophenyl)-3-(2,2-dimethoxyethyl)urea (1b)**. Yield 95%; ^1^H-NMR (400 MHz, CHCl_3_, δ ppm) 3.41 (s, 6H, CH_3_), 3.36-3.45 (m, 2H, CH_2_), 4.40 (t, 1H, *J =* 4.7 Hz, CH), 5.68 (br s, 1H, NH), 7.17–7.25 (m, 4H, Ar-H), 7.48 (br s, 1H, NH); ^13^C-NMR (151 MHz, CHCl_3_, δ ppm) 41.87, 54.73, 103.69, 121.24, 128.18, 128.98, 137.52, 156.12.

**1-(2,2-Dimethoxyethyl)-3-(*p*-tolyl)urea (1c)**. Yield 93%. The spectral characteristics were in agreement with the previously published data [26].

**1-(2,2-Dimethoxyethyl)-3-(4-methoxyphenyl)urea (1d)**. Yield 97%; ^1^H-NMR (400 MHz, CHCl_3_, δ ppm) 3.39 (s, 6H, CH_3_), 3.32–3.44 (m, 2H, CH_2_), 3.77 (s, 3H, CH_3_), 4.40 (t, 1H, *J =* 5.0 Hz, CH), 5.44 (br s, 1H, NH), 6.82 (d, 2H, *J =* 9.0 Hz, Ar-H), 7.08 (br s, 1H, NH), 7.19 (d, 2H, *J =* 9.1 Hz, Ar-H); ^13^C-NMR (151 MHz, CHCl_3_, δ ppm) 41.90, 54.53, 55.51, 103.56, 114.45, 123.49, 131.47, 156.43, 156.83.

**1-(2,2-Dimethoxyethyl)-3-(*m*-tolyl)urea (1e)**. Yield 86%; ^1^H-NMR (400 MHz, CHCl_3_, δ ppm) 2.27 (s, 3H, CH_3_), 3.40 (s, 6H, CH_3_), 3.35-3.45 (m, 2H, CH_2_), 4.42 (t, 1H, *J =* 5.2 Hz, CH), 5.82 (br s, 1H, NH), 6.84 (d, 1H, *J =* 7.2 Hz, Ar-H), 7.07–7.18 (m, 3H, Ar-H), 7.51 (br s, 1H, NH); ^13^C-NMR (151 MHz, CHCl_3_, δ ppm) 21.39, 41.84, 54.48, 103.56, 117.37, 120.99, 124.02, 128.84, 138.83, 138.89, 156.49.

**1-(3-Chlorophenyl)-3-(2,2-dimethoxyethyl)urea (1f)**. Yield 82%; ^1^H-NMR (400 MHz, CHCl_3_, δ ppm) 3.42 (s, 6H, CH_3_), 3.37-3.46 (m, 2H, CH_2_), 4.41 (t, 1H, *J =* 4.9 Hz, CH), 5.77 (br s, 1H, NH), 6.96 (d, 1H, *J =* 7.4 Hz, Ar-H), 7.10–7.19 (m, 2H, Ar-H), 7.39 (s, 1H, Ar-H), 7.65 (br s, 1H, NH); ^13^C-NMR (151 MHz, CHCl_3_, δ ppm) 41.86, 54.69, 103.66, 117.59, 119.62, 122.84, 129.92, 134.56, 140.37, 155.98.

**1-(2,2-Dimethoxyethyl)-1-methyl-3-phenylurea (1g)**. Yield 96%. The spectral characteristics were in agreement with the previously published data [27].

**3-(4-Chlorophenyl)-1-(2,2-dimethoxyethyl)-1-methylurea (1h)**. Yield 91%; ^1^H-NMR (400 MHz, CHCl_3_, δ ppm) 3.03 (s, 3H, CH_3_), 3.43 (d, 2H, *J =* 5.0 Hz, CH_2_), 3.51 (s, 6H, CH_3_), 4.48 (t, 1H, *J =* 5.0 Hz, CH), 7.20–7.24 (m, 2H, Ar-H), 7.25–7.30 (m, 2H, Ar-H), 7.69 (br s, 1H, NH); ^13^C-NMR (151 MHz, CHCl_3_, δ ppm) 35.55, 52.29, 55.04, 103.81, 119.95, 126.72, 128.20, 137.83, 155.97.

**1-(2,2-Dimethoxyethyl)-1-methyl-3-(*p*-tolyl)urea (1j)**. Yield 90%; ^1^H-NMR (400 MHz, CHCl_3_, δ ppm) 2.29 (s, 3H, CH_3_), 3.03 (s, 3H, CH_3_), 3.43 (d, 2H, *J =* 5.0 Hz, CH_2_), 3.50 (s, 6H, CH_3_), 4.48 (t, 1H, *J =* 5.0 Hz, CH), 7.07 (d, 2H, *J =* 8.0 Hz, Ar-H), 7.22 (d, 2H, *J =* 8.2 Hz, Ar-H), 7.47 (br s, 1H, NH); ^13^C-NMR (151 MHz, CHCl_3_, δ ppm) 20.72, 36.06, 52.75, 55.45, 104.39, 119.55, 129.30, 131.92, 137.07, 156.76.

**1-(2,2-Dimethoxyethyl)-3-(4-methoxyphenyl)-1-methylurea (1k).** Yield 96%; ^1^H-NMR (400 MHz, CHCl_3_, δ ppm) 3.00 (s, 3H, CH_3_), 3.40 (d, 2H, *J =* 5.0 Hz, CH_2_), 3.47 (s, 6H, CH_3_), 3.75 (s, 3H, CH_3_), 4.46 (t, 1H, *J =* 5.0 Hz, CH), 6.80 (d, 2H, *J =* 9.0 Hz, Ar-H), 7.21 (d, 2H, *J =* 8.9 Hz, Ar-H), 7.36 (br s, 1H, NH); ^13^C-NMR (151 MHz, CHCl_3_, δ ppm) 36.05, 52.61, 55.38, 55.51, 104.34, 114.08, 121.53, 132.78, 155.42, 157.00.

**1-(2,2-Dimethoxyethyl)-1-methyl-3-(*m*-tolyl)urea (1l)**. Yield 79%; ^1^H-NMR (400 MHz, CHCl_3_, δ ppm) 2.32 (s, 3H, CH_3_), 3.03 (s, 3H, CH_3_), 3.43 (d, 2H, *J =* 4.7 Hz, CH_2_), 3.50 (s, 6H, CH_3_), 4.48 (t, 1H, *J =* 4.7 Hz, CH), 6.81 (d, 1H, *J =* 7.4 Hz, Ar-H), 7.02–7.11 (m, 1H, Ar-H), 7.12–7.17 (m, 1H, Ar-H), 7.21 (s, 1H, Ar-H), 7.53 (br s, 1H, NH); ^13^C-NMR (151 MHz, CHCl_3_, δ ppm) 21.50, 36.05, 52.76, 55.47, 104.37, 116.46, 120.10, 123.30, 128.60, 138.60, 139.58, 156.67.

**3-(3-Chlorophenyl)-1-(2,2-dimethoxyethyl)-1-methylurea (1m)**. Yield 80%; ^1^H-NMR (400 MHz, CHCl_3_, δ ppm) 3.03 (s, 3H, CH_3_), 3.42 (d, 2H, *J =* 5.0 Hz, CH_2_), 3.31 (s, 6H, CH_3_), 4.48 (t, 1H, *J =* 4.9 Hz, CH), 6.93–6.98 (m, 1H, Ar-H), 7.14–7.21 (m, 2H, Ar-H), 7.43 (s, 1H, Ar-H), 7.75 (br s, 1H, NH); ^13^C-NMR (151 MHz, CHCl_3_, δ ppm) 36.09, 52.82, 55.59, 104.31, 117.24, 119.28, 122.36, 129.73, 134.42, 140.99, 156.37.

**General method for the synthesis of imidazolidin-2-ones 2**

To a solution of urea **1** (0.40 g, 1.66 mmol) in toluene (10 mL), the appropriate *C*-nucleophile (1.66 mmol) and TFA (0.19 g, 1.66 mmol) were added and the mixture was refluxed for 64 h. Volatiles were removed under vacuum and the residue was washed with acetone, then recrystallized from absolute ethanol and dried under vacuum (10.1 Torr, r.t., 3 h) to give imidazolidin-2-ones **2**.

**4-(6-Hydroxybenzo[*d*][1,3]dioxol-5-yl)-1-phenylimidazolidin-2-one (2a)**. Beige solid, yield 86%, m.p. 225–226 °C; IR (ν, cm^−1^): 1598, 1615, 2929, 3113, 3203; ^1^H-NMR (400 MHz, DMSO-*d*_6_, δ ppm) 3.48 (dd, 1H, *J* = 6.3 Hz, *J* = 9.1 Hz, H_a_3), 4.23 (dd, 1H, *J* = 8.9 Hz, *J* = 9.1 Hz, H_b_3), 4.96 (dd, 1H, *J* = 3.6 Hz, *J* = 8.9 Hz, H4), 5.90 (m, 2H, H9), 6.48 (s, 1H, H11), 6.79 (s, 1H, H7), 6.97 (t, 1H, *J* = 7.9 Hz, H16), 7.28 (t, 2H, *J* = 7.9 Hz, H15), 7.36 (s, 1H, NH5), 7.54 (d, 2H, *J* = 7.9 Hz, H14), 9.44 (s, 1H, OH); ^13^C-NMR (151 MHz, DMSO-*d*_6_, δ ppm) 46.6 (C4), 52.0 (C3), 97.6 (C11), 100.7 (C9), 105.6 (C7), 117.0 (C14), 120.1 (C6), 121.5 (C16), 128.5 (C15), 139.8 (C8), 140.5 (C13), 146.6 (C10), 149.2 (C12), 158.4 (C1); Elemental analysis: calc. for C_16_H_14_N_2_O_4_ (298): C, 64.42; H, 4.73; N, 9.39; found C, 64.64; H, 4.89; N, 9.27.

**1-(4-Chlorophenyl)-4-(6-hydroxybenzo[*d*][1,3]dioxol-5-yl)imidazolidin-2-one (2b)**. Beige solid, yield 89%, m.p. 252 °C; IR (ν, cm^−1^): 1599, 1617, 2906, 3143, 3255; ^1^H-NMR (400 MHz, DMSO-*d*_6_, δ ppm) 3.44-3.51 (m, 1H, H_a_3), 4.18–4.26 (m, 1H, H_b_3), 4.92–4.98 (m, 1H, H4), 5.89 (m, 2H, H9), 6.48 (s, 1H, H11), 6.78 (s, 1H, H7), 7.32 (d, 2H, *J =* 9.0 Hz, H15), 7.46 (s, 1H, NH5), 7.58 (d, 2H, *J =* 9.1 Hz, H16), 9.46 (s, 1H, OH); ^13^C-NMR (151 MHz, DMSO-*d*_6_, δ ppm) 46.56 (C4), 51.87 (C3), 97.61 (C11), 100.67 (C9), 105.64 (C7), 118.47 (C14), 119.87 (C6), 125.17 (C16), 128.26 (C15), 139.46 (C8), 139.76 (C13), 146.62 (C10), 149.19 (C12), 158.17 (C1); Elemental analysis: calc. for C_16_H_13_ClN_2_O_4_ (332.5): C, 57.76; H, 3.94; Cl, 10.65; N, 8.42; found C, 57.91; H, 4.14; Cl, 10.83; N, 8.59.

**4-(6-Hydroxybenzo[*d*][1,3]dioxol-5-yl)-1-(*p*-tolyl)imidazolidin-2-one (2c)**. Beige solid, yield 78%, m.p. 257–258 °C; IR (ν, cm^−1^): 1597, 1627, 2974, 3154, 3275; ^1^H-NMR (400 MHz, DMSO-*d*_6_, δ ppm) 2.24 (s, 3H, H17), 3.45 (dd, 1H, *J* = 6.2 Hz, *J* = 9.0 Hz, H_a_3), 4.20 (dd, 1H, *J* = 8.9 Hz, *J* = 9.0 Hz, H_b_3), 4.95 (dd, 1H, *J* = 3.7 Hz, *J* = 8.9 Hz, H4), 5.90 (d, 2H, *J* = 17.1 Hz, H9), 6.47 (s, 1H, H11), 6.78 (s, 1H, H7), 7.09 (d, 2H, *J* = 8.4 Hz, H15), 7.29 (br s, 1H, NH5), 7.43 (d, 2H, *J* = 8.6 Hz, H14), 9.43 (s, 1H, OH); ^13^C-NMR (151 MHz, DMSO-*d*_6_, δ ppm) 20.20 (C17), 46.6 (C4), 52.1 (C3), 97.6 (C11), 100.6 (C9), 105.6 (C7), 117.1 (C15), 120.1 (C6), 128.9 (C14), 130.3 (C16), 138.1 (C13), 139.8 (C8), 146.5 (C10), 149.1 (C12), 158.5 (C1); Elemental analysis: calc. for C_17_H_16_N_2_O_4_ (312): C, 65.38; H, 5.16; N, 8.97; found C, 65.47; H, 5.26; N, 9.19.

**4-(6-Hydroxybenzo[*d*][1,3]dioxol-5-yl)-1-(4-methoxyphenyl)imidazolidin-2-one (2d)**. Beige solid, yield 72%, m.p. 227–228 °C; IR (ν, cm^−1^): 1597, 1618, 3153, 3284; ^1^H-NMR (400 MHz, DMSO-*d*_6_, δ ppm) 3.39–3.48 (m, 1H, H_a_3), 3.71 (s, 3H, H17), 4.14–4.22 (m, 1H, H_b_3), 4.90–4.99 (m, 1H, H4), 5.90 (d, 2H, *J =* 19.8 Hz, H9), 6.48 (s, 1H, H11), 6.79 (s, 1H, H7), 6.86 (d, 2H, *J =* 9.1 Hz, H15), 7.21 (br s, 1H, NH5), 7.44 (d, 2H, *J =* 9.0 Hz, H14), 9.42 (s, 1H, OH18); ^13^C-NMR (151 MHz, DMSO-*d*_6_, δ ppm) 47.22 (C4), 52.92 (C3), 55.68 (C17), 98.14 (C11), 101.18 (C9), 106.12 (C7), 114.31 (C15), 119.38 (C14), 120.70 (C6), 134.41 (C13), 140.30 (C8), 147.06 (C10), 149.65 (C12), 154.83 (C16), 159.14 (C1); Elemental analysis: calc. for C_17_H_16_N_2_O_5_ (328): C, 62.19; H, 4.91; N, 8.53; found C, 62.35; H, 5.09; N, 8.75.

**4-(6-Hydroxybenzo[*d*][1,3]dioxol-5-yl)-1-(*m*-tolyl)imidazolidin-2-one (2e)**. Beige solid, yield 53%, m.p. 213 °C; IR (ν, cm^−1^): 1595, 1613, 3153, 3205; ^1^H-NMR (400 MHz, DMSO-*d*_6_, δ ppm) 2.27 (s, 3H, H19), 3.42–3.49 (m, 1H, H_a_3), 4.18–4.27 (m, 1H, H_b_3), 4.91–4.99 (m, 1H, H4), 5.90 (d, 2H, *J =* 13.7 Hz, H9), 6.48 (s, 1H, H11), 6.78 (s, 1H, H7), 6.80 (br s, 1H, NH5), 7.11–7.21 (m, 1H, H14), 7.26–7.41 (m, 3H, H16,17,18), 9.44 (s, 1H, OH20); ^13^C-NMR (151 MHz, DMSO-*d*_6_, δ ppm) 21.75 (C19), 47.10 (C4), 52.61 (C3), 98.13 (C11), 101.15 (C9), 106.04 (C7), 114.79 (C14), 118.16 (C16), 120.68 (C18), 122.76 (C17), 128.81 (C15), 138.15 (C8), 140.28 (C13), 147.07 (C10), 149.63 (C12), 158.91 (C1); Elemental analysis: calc. for C_17_H_16_N_2_O_4_ (312): C, 65.38; H, 5.16; N, 8.97; found C, 65.24; H, 5.32; N, 8.86.

**1-(3-Chlorophenyl)-4-(6-hydroxybenzo[*d*][1,3]dioxol-5-yl)imidazolidin-2-one (2f)**. Beige solid, yield 48%, m.p. 201–202 °C; IR (ν, cm^−1^): 1597, 1615, 2943, 3183, 3287; ^1^H-NMR (400 MHz, DMSO-*d*_6_, δ ppm) 3.46–3.56 (m, 1H, H_a_3), 4.18–4.29 (m, 1H, H_b_3), 4.92–5.02 (m, 1H, H4), 5.90 (d, 2H, *J =* 15.8 Hz, H9), 6.48 (s, 1H, Ar-H), 6.78 (s, 1H, Ar-H), 6.97–7.04 (m, 1H, Ar-H), 7.26–7.31 (m, 1H, Ar-H), 7.33–7.39 (m, 1H, H11), 7.54 (s, 1H, H7), 7.81 (br s, 1H, NH5), 9.47 (s, 1H, OH19); ^13^C-NMR (151 MHz, DMSO-*d*_6_, δ ppm) 46.58 (C4), 51.79 (C3), 97.62 (C11), 100.67 (C9), 105.70 (C7), 115.06 (C16), 116.58 (C17), 119.79 (C18), 121.01 (C6), 130.08 (C14), 133.08 (C15), 139.77 (C8), 141.94 (C13), 146.66 (C10), 149.23 (C12), 158.09 (C1); Elemental analysis: calc. for C_16_H_13_ClN_2_O_4_ (332.5): C, 57.76; H, 3.94; Cl, 10.65; N, 8.42; found C, 57.88; H, 4.19; Cl, 10.44; N, 8.42.

**1-Phenyl-4-(6-hydroxybenzo[*d*][1,3]dioxol-5-yl)-3-methylimidazolidin-2-one (2g).** Yield 91%. The spectral characteristics were in agreement with the previously published data [19].

**1-(4-Chlorophenyl)-4-(6-hydroxybenzo[*d*][1,3]dioxol-5-yl)-3-methylimidazolidin-2-one (2h).** Beige solid, yield 24%, m.p. 218–219 °C; IR (ν, cm^−1^): 1596, 1617, 2976, 3181, 3298; ^1^H-NMR (400 MHz, DMSO-*d*_6_, δ ppm) 2.60 (s, 3H, H5), 3.46–3.55 (m, 1H, H_a_3), 4.08–4.17 (m, 1H, H_b_3), 4.86–4.94 (m, 1H, H4), 5.92 (d, 2H, *J =* 11.1 Hz, H9), 6.49 (s, 1H, H11), 6.68 (s, 1H, H7), 7.33 (d, 2H, *J =* 9.0 Hz, H15), 7.61 (d, 2H, *J =* 9.1 Hz, H14), 9.50 (s, 1H, OH17); ^13^C-NMR (151 MHz, DMSO-*d*_6_, δ ppm) 28.93 (C5), 49.43 (C4), 52.40 (C3), 97.82 (C11), 100.81 (C9), 106.34 (C7), 116.52 (C14), 118.29 (C15), 125.18 (C16), 128.32 (C14), 139.57 (C8), 140.17 (C13), 147.15 (C10), 150.33 (C12), 157.06 (C1); Elemental analysis: calc. for C_17_H_15_ClN_2_O_4_ (346.5): C, 58.88; H, 4.36; Cl, 10.22; N, 8.08; found C, 59.00; H, 4.45; Cl, 10.34; N, 8.16.

**4-(6-Hydroxybenzo[*d*][1,3]dioxol-5-yl)-3-methyl-1-(*p*-tolyl)imidazolidin-2-one (2j)**. Beige solid, yield 32%, m.p. 182–183 °C; IR (ν, cm^−1^): 1597, 1618, 2904, 3101, 3189;^1^H-NMR (400 MHz, DMSO-*d*_6_, δ ppm) 2.24 (s, 3H, H17), 2.59 (s, 3H, H5), 3.42–3.48 (m, 1H, H_a_3), 4.05–4.14 (m, 1H, H_b_3), 4.83–4.91 (m, 1H, H4), 5.91 (d, 2H, *J =* 14.5 Hz, H9), 6.49 (s, 1H, H11), 6.67 (s, 1H, H7), 7.10 (d, 2H, *J =* 8.1 Hz, H15), 7.46 (d, 2H, *J =* 8.5 Hz, H14), 9.45 (s, 1H, OH18); ^13^C-NMR (151 MHz, DMSO-*d*_6_, δ ppm) 20.75 (C17), 29.62 (C5), 50.20 (C4), 53.01 (C3), 98.37 (C11), 101.33 (C9), 106.64 (C7), 117.35 (C6), 117.54 (C15), 129.48 (C14), 130.94 (C16), 138.73 (C13), 140.73 (C8), 147.59 (C10), 150.79 (C12), 157.96 (C1); Elemental analysis: calc. for C_18_H_18_N_2_O_4_ (326): C, 66.25; H, 5.56; N, 8.58; found C, 66.39; H, 5.67; N, 8.47.

**4-(6-Hydroxybenzo[*d*][1,3]dioxol-5-yl)-1-(4-methoxyphenyl)-3-methylimidazolidin-2-one (2k)**. Beige solid, yield 49%, m.p. 186–187 °C; IR (ν, cm^−1^): 1597, 1617, 2978, 3104, 3257; ^1^H-NMR (400 MHz, DMSO-*d*_6_, δ ppm) 2.59 (s, 3H, H5), 3.43–3.48 (m, 1H, H_a_3), 3.71 (s, 3H, H17), 4.05–4.12 (m, 1H, H_b_3), 4.80–4.90 (m, 1H, H4), 5.91 (d, 2H, *J =* 14.7 Hz, H9), 6.49 (s, 1H, H11), 6.68 (s, 1H, H7), 6.88 (d, 2H, *J =* 9.0 Hz, H15), 7.47 (d, 2H, *J =* 8.9 Hz, H14), 9.46 (s, 1H, OH18); ^13^C-NMR (151 MHz, DMSO-*d*_6_, δ ppm) 29.69 (C5), 50.53 (C4), 53.07 (C3), 55.69 (C17), 98.37 (C11), 101.32 (C9), 106.62 (C7), 114.37 (C15), 117.35 (C6), 119.31 (C14), 134.52 (C16), 140.73 (C13), 147.57 (C8), 150.79 (C10), 154.90 (C12), 158.16 (C1); Elemental analysis: calc. for C_18_H_18_N_2_O_5_ (342): C, 63.15; H, 5.30; N, 8.18; found C, 63.37; H, 5.41; N, 8.21.

**4-(6-Hydroxybenzo[*d*][1,3]dioxol-5-yl)-3-methyl-1-(*m*-tolyl)imidazolidin-2-one (2l)**. Beige solid, yield 50%, m.p. 180 °C; IR (ν, cm^−1^): 1595, 1615, 2893, 3101, 3179; ^1^H-NMR (400 MHz, DMSO-*d*_6_, δ ppm) 2.27 (s, 3H, H19), 2.60 (s, 3H, H5), 3.46–3.54 (m, 1H, H_a_3), 4.07–4.15 (m, 1H, H_b_3), 4.85–4.91 (m, 1H, H4), 5.91 (d, 2H, *J =* 11.5 Hz, H9), 6.50 (s, 1H, H11), 6.66 (s, 1H, H7), 6.76–6.82 (m, 1H, Ar-H), 7.13–7.20 (m, 1H, Ar-H), 7.36–7.43 (m, 2H, Ar-H), 9.47 (s, 1H, OH20); ^13^C-NMR (151 MHz, DMSO-*d*_6_, δ ppm) 21.79 (C19), 29.56 (C5), 50.20 (C3), 52.99 (C4), 98.38 (C11), 101.33 (C9), 106.64 (C7), 114.71 (C14), 117.34 (C6), 118.05 (C18), 122.83 (C16), 128.88 (C17), 138.23 (C15), 140.72 (C13), 141.11 (C8), 147.61 (C10), 150.80 (C12), 157.87 (C1); Elemental analysis: calc. for C_18_H_18_N_2_O_4_ (326): C, 66.25; H, 5.56; N, 8.58; found C, 66.16; H, 5.70; N, 8.68.

**1-(3-Chlorophenyl)-4-(6-hydroxybenzo[*d*][1,3]dioxol-5-yl)-3-methylimidazolidin-2-one (2m)**. Beige solid, yield 77%, m.p. 210 °C; IR (ν, cm^−1^): 1596, 1617, 3134, 3233; ^1^H-NMR (400 MHz, DMSO-*d*_6_, δ ppm) 2.60 (s, 3H, H5), 3.49–3.57 (m, 1H, H_a_3), 4.09–4.18 (m, 1H, H_b_3), 4.86–4.94 (m, 1H, H4), 5.92 (d, 2H, *J =* 9.4 Hz, H9), 6.49 (s, 1H, H11), 6.70 (s, 1H, H7), 6.98–7.05 (m, 1H, Ar-H), 7.27–7.37 (m, 1H, Ar-H), 7.38–7.46 (m, 1H, Ar-H), 7.79–7.86 (m, 1H, Ar-H), 9.49 (s, 1H, OH19); ^13^C-NMR (151 MHz, DMSO-*d*_6_, δ ppm) 28.83 (C5), 49.35 (C3), 52.43 (C4), 97.82 (C11), 100.81 (C9), 106.45 (C7), 114.93 (C18), 116.37 (C17), 116.42 (C16), 121.02 (C6), 130.11 (C14), 133.14 (C15), 140.16 (C13), 142.04 (C8), 147.18 (C10), 150.37 (C12), 156.92 (C1); Elemental analysis: calc. for C_17_H_15_ClN_2_O_4_ (346.5): C, 59.88; H, 4.36; Cl, 10.22; N, 8.08; found C, 58.99; H, 4.49; Cl, 10.46; N, 8.20.

**4-(5-Chloro-2,4-dihydroxyphenyl)-1-phenylimidazolidin-2-one (3a)**. Beige solid, yield 49%, m.p. 213–214 °C; IR (ν, cm^−1^): 1597, 1625, 2902, 3182, 3248, 3348; ^1^H-NMR (400 MHz, DMSO-*d*_6_, δ ppm) 3.46–3.54 (m, 1H, H_a_3), 4.18–4.26 (m, 1H, H_b_3), 4.83–4.92 (m, 1H, H4), 6.56 (s, 1H, H10), 6.97 (t, 1H, *J =* 7.3 Hz, H17), 7.11 (s, 1H, H7), 7.28 (t, 2H, *J =* 7.4 Hz, H16), 7.37 (br s, 1H, NH5), 7.54 (d, 2H, *J =* 7.8 Hz, H15), 9.80 (s, 1H, OH12), 9.94 (s, 1H, OH13); ^13^C-NMR (151 MHz, DMSO-*d*_6_, δ ppm) 46.32 (C4), 51.78 (C3), 103.70 (C10), 109.32 (C8), 117.00 (C15), 120.62 (C6), 121.48 (C7), 126.60 (C17), 128.47 (C16), 140.48 (C4), 152.71 (C11), 154.02 (C9), 158.29 (C1); Elemental analysis: calc. for C_15_H_13_ClN_2_O_3_ (304.5): C, 59.12; H, 4.30; Cl, 11.63; N, 9.19; found C, 59.37; H, 4.54; Cl, 11.81; N, 9.37.

**4-(5-Chloro-2,4-dihydroxyphenyl)-1-(4-methoxyphenyl)imidazolidin-2-one (3d)**. Beige solid, yield 36%, m.p. 225–226 °C; IR (ν, cm^−1^): 1594, 1626, 3182, 3249, 3345; ^1^H-NMR (400 MHz, DMSO-*d*_6_, δ ppm) 3.42–3.50 (m, 1H, H_a_3), 3.47 (s, 3H, H18), 4.15–4.23 (m, 1H, H_b_3), 4.83-4.91 (m, 1H, H4), 6.56 (s, 1H, H10), 6.86 (d, 2H, *J =* 9.2 Hz, H16), 7.12 (s, 1H, H7), 7.24 (br s, 1H, NH5), 7.44 (d, 2H, *J =* 9.1 Hz, H15), 9.81 (s, 1H, OH12), 9.95 (s, 1H, OH13); ^13^C-NMR (151 MHz, DMSO-*d*_6_, δ ppm) 46.40 (C4), 52.21 (C3), 55.12 (C18), 103.69 (C10), 109.34 (C8), 113.77 (C16), 118.82 (C15), 120.70 (C6), 126.58 (C7), 133.83 (C14), 152.67 (C11), 154.00 (C17), 154.29 (C9), 158.52 (C1); Elemental analysis: calc. for C_16_H_15_ClN_2_O_4_ (334.5): C, 57.41; H, 4.52; Cl, 10.59; N, 8.37; found C, 57.50; H, 4.61; Cl, 10.78; N, 8.36.

**4-(5-Chloro-2,4-dihydroxyphenyl)-1-(*m*-tolyl)imidazolidin-2-one (3e)**. Beige solid, yield 51%, m.p. 201–202 °C; IR (ν, cm^−1^): 1591, 1628, 2932, 3141, 3209, 3323; ^1^H-NMR (400 MHz, DMSO-*d*_6_, δ ppm) 3.27 (s, 3H, H20), 3.42–3.52 (m, 1H, H_a_3), 4.15–4.24 (m, 1H, H_a_3), 4.81–4.92 (m, 1H, H4), 6.56 (s, 1H, H10), 6.76–6.81 (m, 1H, Ar-H), 7.10 (s, 1H, H7), 7.25–7.53 (m, 4H, Ar-H, NH), 9.80 (s, 1H, OH12), 9.94 (s, 1H, OH13); ^13^C-NMR (151 MHz, DMSO-*d*_6_, δ ppm) 21.24 (C20), 46.31 (C4), 51.90 (C3), 103.70 (C10), 109.32 (C8), 114.25 (C17), 117.60 (C19), 120.69 (C6), 122.25 (C15), 126.55 (C7), 128.30 (C18), 137.64 (C16), 140.44 (C14), 152.70 (C11), 154.00 (C9), 158.32 (C1); Elemental analysis: calc. for C_16_H_15_ClN_2_O_3_ (318.5): C, 60.29; H, 4.74; Cl, 11.12; N, 8.79; found C, 60.56; H, 4.80; Cl, 11.26; N, 8.99.

**1,5-Dimethyl-4-(2-oxo-1-phenylimidazolidin-4-yl)-2-phenyl-1,2-dihydro-3*H*-pyrazol-3-one (4a).** Beige solid, yield 44%, m.p. 199–200 °C; IR (ν, cm^−1^): 1598, 1613, 2969, 3316, 3379; ^1^H-NMR (400 MHz, DMSO-*d*_6_, δ ppm) 2.29 (s, 3H, H9), 3.08 (s, 3H, H10), 3.95–4.10 (m, 2H, H3), 4.70–4.79 (m, 1H, H4), 6.97 (t, 1H, *J =* 7.3 Hz, H14), 7.13 (br s, 1H, NH5), 7.27–7.35 (m, 5H, Ar-H), 7.48 (t, 2H, *J =* 7.8 Hz, Ar-H), 7.58 (d, 2H, *J =* 8.4 Hz, Ar-H); ^13^C-NMR (151 MHz, DMSO-*d*_6_, δ ppm) 10.63 (C9), 35.36 (C10), 42.61 (C4), 48.74 (C3), 106.39 (C6), 116.85 (C16), 121.29 (C14), 123.50 (C12), 126.12 (C18), 128.45 (C13), 128.94 (C17), 134.99 (C11), 140.61 (C15), 154.84 (C7), 158.10 (C1), 163.78 (C8); Elemental analysis: calc. for C_20_H_20_N_4_O_2_ (348): C, 68.95; H, 5.79; N, 16.08; found C, 69.11; H, 5.96; N, 16.22.

**4-(1-(4-Chlorophenyl)-2-oxoimidazolidin-4-yl)-1,5-dimethyl-2-phenyl-1,2-dihydro-3*H*-pyrazol-3-one (4b)**. Beige solid, yield 64%, m.p. 203–204 °C; IR (ν, cm^−1^): 1597, 1612, 2954, 3101, 3322; ^1^H-NMR (400 MHz, DMSO-*d*_6_, δ ppm) 2.28 (s, 3H, H9), 3.07 (s, 3H, H10), 3.92–4.00 (m, 1H, H3), 4.02–4.09 (m, 1H, H3), 4.72–4.79 (m, 1H, H4), 7.24 (br s, 1H, NH5), 7.27–7.38 (m, 5H, Ar-H), 7.46–7.51 (m, 2H, Ar-H), 7.61 (d, 2H, *J =* 9.0 Hz, Ar-H); ^13^C-NMR (151 MHz, DMSO-*d*_6_, δ ppm) 10.59 (C9), 35.35 (C10), 42.51 (C4), 48.62 (C3), 106.22 (C6), 118.26 (C16), 123.51 (C12), 124.96 (C18), 126.14 (C14), 128.25 (C13), 128.94 (C17), 134.95 (C11), 139.56 (C15), 154.80 (C7), 157.87 (C1), 163.72 (C8); Elemental analysis: calc. for C_20_H_19_ClN_4_O_2_ (382.5): C, 62.75; H, 5.00; Cl, 9.26; N, 14.63; found C, 62.98; H, 5.21; Cl, 9.29; N, 14.49.

**1,5-Dimethyl-4-(2-oxo-1-(p-tolyl)imidazolidin-4-yl)-2-phenyl-1,2-dihydro-3*H*-pyrazol-3-one (4c).** Beige solid, yield 47%, m.p. 213–214 °C; IR (ν, cm^−1^): 1595, 1615, 3310, 3328; ^1^H-NMR (400 MHz, DMSO-*d*_6_, δ ppm) 2.24 (s, 3H, H19), 2.28 (s, 3H, H9), 3.07 (s, 3H, H10), 3.92–3.98 (m, 1H, H3), 3.99–4.05 (m, 1H, H3), 4.69–4.77 (m, 1H, H4), 7.05 (br s, 1H, NH5), 7.10 (d, 2H, *J =* 8.3 Hz, Ar-H), 7.28–7.34 (m, 3H, Ar-H), 7.44–7.52 (m, 4H, Ar-H); ^13^C-NMR (151 MHz, DMSO-*d*_6_, δ ppm) 10.64 (C9), 20.21 (C19), 35.37 (C10), 42.66 (C4), 48.85 (C3), 106.44 (C6), 116.94 (C16), 123.49 (C12), 126.11 (C14), 128.86 (C13), 128.94 (C17), 130.11 (C18), 135.00 (C11), 138.18 (C15), 154.84 (C7), 158.18 (C1), 163.79 (C8); Elemental analysis: calc. for C_21_H_22_N_4_O_2_ (362): C, 69.59; H, 6.12; N, 15.46; found C, 69.77; H, 6.27; N, 15.55.

**4-(1-(4-Methoxyphenyl)-2-oxoimidazolidin-4-yl)-1,5-dimethyl-2-phenyl-1,2-dihydro-3*H*-pyrazol-3-one (4d).** Beige solid, yield 50%, m.p. 155 °C; IR (ν, cm^−1^): 1598, 1613, 2969, 3316, 3379; ^1^H-NMR (400 MHz, DMSO-*d*_6_, δ ppm) 2.29 (s, 3H, H9), 3.07 (s, 3H, H10), 3.72 (s, 3H, H19), 3.92–4.07 (m, 2H, H3), 4.68–4.76 (m, 1H, H4), 6.88 (d, 2H, *J =* 9.0 Hz, Ar-H), 6.99 (br s, 1H, NH5), 7.29–7.35 (m, 3H, Ar-H), 7.46–7.51 (m, 4H, Ar-H); ^13^C-NMR (151 MHz, DMSO-*d*_6_, δ ppm) 10.65 (C9), 35.37 (C10), 42.73 (C4), 49.18 (C3), 55.15 (C19), 106.48 (C6), 113.76 (C16), 118.65 (C12), 123.49 (C13), 126.10 (C14), 128.94 (C17), 134.00 (C11), 135.01 (C15), 154.17 (C18), 154.85 (C7), 158.32 (C1), 163.81 (C8); Elemental analysis: calc. for C_21_H_22_N_4_O_3_ (378): C, 66.65; H, 5.86; N, 14.81; found C, 66.41; H, 5.88; N, 14.87.

**1,5-Dimethyl-4-(2-oxo-1-(*m*-tolyl)imidazolidin-4-yl)-2-phenyl-1,2-dihydro-3*H*-pyrazol-3-one (4e)**. Beige solid, yield 57%, m.p. 185–186 °C; IR (ν, cm^−1^): 1595, 1617, 2969, 3319; ^1^H-NMR (400 MHz, DMSO-*d*_6_, δ ppm) 2.28 (s, 3H,H21), 2.29 (s, 3H, H9), 3.08 (s, 3H, H10), 3.93–4.09 (m, 2H, H3), 4.69–4.77 (m, 1H, H4), 6.79 (d, 1H, *J =* 7.5 Hz, Ar-H), 7.10 (br s, 1H, NH), 7.17 (t, 1H, *J =* 7.8 Hz, Ar-H), 7.29–7.34 (m, 3H, Ar-H), 7.36–7.41 (m, 2H, Ar-H), 7.47–7.50 (m, 2H, Ar-H); ^13^C-NMR (151 MHz, DMSO-*d*_6_, δ ppm) 10.63 (C9), 21.30 (C21), 35.37 (C10), 42.60 (C4), 48.86 (C3), 106.46 (C6), 114.11 (C16), 117.44 (C20), 122.05 (C18), 123.49 (C12), 126.11 (C19), 128.28 (C14), 128.94 (C13), 135.00 (C17), 137.57 (C11), 140.57 (C15), 154.84 (C7), 158.12 (C1), 163.79 (C8); Elemental analysis: calc. for C_21_H_22_N_4_O_2_ (362): C, 69.59; H, 6.12; N, 15.46; found C, 69.72; H, 6.26; N, 15.29.

**4-(1-phenyl-3-methyl-2-oxoimidazolidin-4-yl)-1,5-dimethyl-2-phenyl-1,2-dihydro-3*H*-pyrazol-3-one (4g).** Yield 60%. The spectral characteristics were in agreement with the previously published data [19].

**4-(1-(4-Chlorophenyl)-3-methyl-2-oxoimidazolidin-4-yl)-1,5-dimethyl-2-phenyl-1,2-dihydro-3*H*-pyrazol-3-one (4h)**. Beige solid, yield 43%, m.p. 245–247 °C; IR (ν, cm^−1^): 1595, 1613; ^1^H-NMR (400 MHz, DMSO-*d*_6_, δ ppm) 2.31 (s, 3H, H9), 2.62 (s, 3H, H10), 3.13 (s, 3H, H5), 3.91–4.06 (m, 2H, H3), 4.62–4.70 (m, 1H, H4), 7.30–7.37 (m, 5H, Ar-H), 7.46–7.53 (m, 2H, Ar-H), 7.59–7.66 (m, 2H, Ar-H); ^13^C-NMR (151 MHz, DMSO-*d*_6_, δ ppm) 10.87 (C9), 28.73 (C5), 35.81 (C10), 46.63 (C3), 48.97 (C4), 102.99 (C6), 118.62 (C16), 124.32 (C12), 125.56 (C18), 126.86 (C14), 128.85 (C13), 129.49 (C17), 135.44 (C11), 140.13 (C15), 156.25 (C7), 157.10 (C1), 164.26 (C8); Elemental analysis: calc. for C_21_H_21_ClN_4_O_2_ (396.5): C, 63.55; H, 5.33; Cl, 8.93; N, 14.12; found C, 63.69; H, 5.20; Cl, 9.06; N, 14.12.

**1,5-Dimethyl-4-(3-methyl-2-oxo-1-(*p*-tolyl)imidazolidin-4-yl)-2-phenyl-1,2-dihydro-3*H*-pyrazol-3-one (4j)**. Beige solid, yield 24%, m.p. 248 °C; IR (ν, cm^−1^): 1592, 1614; ^1^H-NMR (400 MHz, DMSO-*d*_6_, δ ppm) 2.24 (s, 3H, H9), 2.30 (s, 3H, H19), 2.60 (s, 3H, H10), 3.12 (s, 3H, H5), 3.90–3.98 (m, 2H, H3), 4.59–4.65 (m, 1H, H4), 7.10 (d, 2H, *J =* 8.1 Hz, Ar-H), 7.29–7.35 (m, 3H, Ar-H), 7.44–7.51 (m, 4H, Ar-H); ^13^C-NMR (151 MHz, DMSO-*d*_6_, δ ppm) 10.93 (C9), 20.75 (C19), 28.91 (C5), 35.86 (C10), 46.77 (C3), 49.22 (C4), 103.21 (C6), 117.34 (C12), 124.30 (C16), 126.84 (C14), 129.48 (C13), 129.51 (C17), 130.78 (C18), 135.50 (C11), 138.80 (C15), 156.31 (C7), 157.49 (C1), 164.38 (C8); Elemental analysis: calc. for C_22_H_24_N_4_O_2_ (376): C, 70.19; H, 6.43; N, 14.88; found C, 70.23; H, 6.49; N, 14.88.

**4-(1-(4-Methoxyphenyl)-3-methyl-2-oxoimidazolidin-4-yl)-1,5-dimethyl-2-phenyl-1,2-dihydro-3*H*-pyrazol-3-one (4k)**. Beige solid, yield 31%, m.p. 209–210 °C; IR (ν, cm^−1^): 1595, 1613; ^1^H-NMR (400 MHz, DMSO-*d*_6_, δ ppm) 2.31 (s, 3H, H9), 2.61 (s, 3H, H10), 3.12 (s, 3H, H5), 3.72 (s, 3H, H19), 3.90–3.99 (m, 2H, H3), 4.56–4.65 (m, 1H, H4), 6.90 (d, 2H, *J =* 9.1 Hz, Ar-H), 7.32–7.36 (m, 3H, Ar-H), 7.42–7.52 (m, 4H, Ar-H); ^13^C-NMR (151 MHz, DMSO-*d*_6_, δ ppm) 28.95 (C9), 31.13 (C5), 35.82 (C10), 47.06 (C3), 49.29 (C4), 55.66 (C19), 103.18 (C6), 114.35 (C12), 119.04 (C16), 124.29 (C13), 126.82 (C14), 129.49 (C17), 134.58 (C11), 135.45 (C15), 154.76 (C19), 156.25 (C7), 157.65 (C1), 164.37 (C8); Elemental analysis: calc. for C_22_H_24_N_4_O_3_ (392): C, 67.33; H, 6.16; N, 14.28; found C, 67.51; H, 6.29; N, 14.40.

**1,5-Dimethyl-4-(3-methyl-2-oxo-1-(*m*-tolyl)imidazolidin-4-yl)-2-phenyl-1,2-dihydro-3*H*-pyrazol-3-one (4l)**. Beige solid, yield 26%, m.p. 215–216 °C; IR (ν, cm^−1^): 1592, 1613; ^1^H-NMR (400 MHz, DMSO-*d*_6_, δ ppm) 2.28 (s, 3H, H9), 2.30 (s, 3H, H21), 2.61 (s, 3H, H10), 3.12 (s, 3H, H5), 3.91–4.01 (m, 2H, H3), 4.58–4.67 (m, 1H, H4), 6.80 (d, 1H, *J =* 6.9 Hz, Ar-H), 7.17 (t, 1H, *J =* 7.5 Hz, Ar-H), 7.30– 7.36 (m, 3H, Ar-H), 7.38–7.43 (m, 2H, Ar-H), 7.48 (t, 2H, *J =* 7.4 Hz, Ar-H); ^13^C-NMR (151 MHz, DMSO-*d*_6_, δ ppm) 10.92 (C9), 21.83 (C21), 28.87 (C5), 35.86 (C10), 46.78 (C3), 49.12 (C4), 103.23 (C6), 114.52 (C16), 117.82 (C18), 122.68 (C20), 124.30 (C12), 126.84 (C14), 128.89 (C19), 129.51 (C13), 135.50 (C17), 138.19 (C11), 141.18 (C15), 156.31 (C7), 157.40 (C1), 164.37 (C8); Elemental analysis: calc. for C_22_H_24_N_4_O_2_ (376): C, 70.19; H, 6.43; N, 14.88; found C, 70.37; H, 6.61; N, 14.71.

**4-(4-Hydroxy-6-methyl-2-oxo-2*H*-pyran-3-yl)-1-phenylimidazolidin-2-one (5a).** Beige solid, yield 41%, m.p. 182–183 °C; IR (ν, cm^−1^): 1598, 1613, 3145, 3271; ^1^H-NMR (400 MHz, DMSO-*d*_6_, δ ppm) 2.17 (s, 3H, H11), 3.75–3.82 (m, 1H, H_a_3), 3.99–4.07 (m, 1H, H_b_3), 4.97–5.05 (m, 1H, H4), 6.01 (s, 1H, H8), 6.92 (br s, 1H, NH5), 6.94 (t, 1H, *J =* 7.2 Hz, H15), 7.27 (t, 2H, *J =* 7.7 Hz, H14), 7.57 (d, 2H, *J =* 8.4 Hz, H13), 11.75 (s, 1H, OH16); ^13^C-NMR (151 MHz, DMSO-*d*_6_, δ ppm) 19.87 (C11), 42.57 (C4), 48.84 (C3), 100.41 (C6), 100.79 (C8), 117.04 (C14), 121.38 (C15), 128.92 (C13), 141.44 (C12), 158.81 (C1), 162.54 (C9), 163.44 (C7), 167.55 (C10); Elemental analysis: calc. for C_15_H_14_N_2_O_4_ (286): C, 62.93; H, 4.93; N, 9.79; found C, 63.12; H, 5.13; N, 9.91.

**1-(4-Chlorophenyl)-4-(4-hydroxy-6-methyl-2-oxo-2*H*-pyran-3-yl)imidazolidin-2-one (5b)**. Beige solid, yield 66%, m.p. 190–191 °C; IR (ν, cm^−1^): 1592, 1613, 3102, 3228; ^1^H-NMR (400 MHz, DMSO-*d*_6_, δ ppm) 2.18 (s, 3H, H11), 3.77–3.80 (m, 1H, H_a_3), 4.00–4.10 (m, 1H, H_b_3), 4.96–5.05 (m, 1H, H4), 6.02 (s, 1H, H8), 7.05 (br s, 1H, NH5), 7.32 (d, 2H, *J =* 9.1 Hz, H14), 7.61 (d, 2H, *J =* 9.0 Hz, H13), 11.80 (s, 1H, OH16); ^13^C-NMR (151 MHz, DMSO-*d*_6_, δ ppm) 19.84 (C11), 42.46 (C4), 48.80 (C3), 100.38 (C6), 100.69 (C8), 118.45 (C14), 125.03 (C15), 128.70 (C13), 140.36 (C12), 158.59 (C1), 162.57 (C9), 163.39 (C7), 167.55 (C10); Elemental analysis: calc. for C_15_H_13_ClN_2_O_4_ (320.5): C, 56.17; H, 4.09; Cl, 11.05; N, 8.73; found C, 55.98; H, 3.95; Cl, 10.87; N, 8.68.

**4-(4-Hydroxy-6-methyl-2-oxo-2*H*-pyran-3-yl)-1-(*p*-tolyl)imidazolidin-2-one (5c)**. Beige solid, yield 86%, m.p. 184–185 °C; IR (ν, cm^−1^): 1593, 1620, 3132, 3288; ^1^H-NMR (400 MHz, DMSO-*d*_6_, δ ppm) 2.17 (s, 3H, H11), 2.24 (s, 3H, H16), 3.74–3.82 (m, 1H, H_a_3), 3.96–4.05 (m, 1H, H_b_3), 4.95–5.02 (m, 1H, H4), 6.01 (s, 1H, H8), 6.84 (br s, 1H, NH5), 7.08 (d, 2H, *J =* 8.4 Hz, H14), 7.45 (d, 2H, *J =* 8.5 Hz, H13), 11.74 (s, 1H, OH17); ^13^C-NMR (151 MHz, DMSO-*d*_6_, δ ppm) 19.32 (C11), 20.19 (C16), 42.08 (C4), 48.36 (C3), 99.88 (C6), 100.25 (C8), 116.57 (C14), 128.79 (C13), 129.59 (C15), 138.49 (C12), 158.34 (C1), 161.96 (C9), 162.89 (C7), 167.00 (C10); Elemental analysis: calc. for C_16_H_16_N_2_O_4_ (300): C, 63.99; H, 5.37; N, 9.33; found C, 64.09; H, 5.47; N, 9.49.

**4-(4-Hydroxy-6-methyl-2-oxo-2*H*-pyran-3-yl)-1-(4-methoxyphenyl)imidazolidin-2-one (5d)**. Beige solid, yield 84%, m.p. 184–185 °C; IR (ν, cm^−1^): 1593, 1614, 3218; ^1^H-NMR (400 MHz, DMSO-*d*_6_, δ ppm) 2.17 (s, 3H, H11), 3.71 (s, 3H, H16), 3.76–3.78 (m, 1H, H_a_3), 3.96–4.02 (m, 1H, H_b_3), 4.94–5.00 (m, 1H, H4), 6.01 (s, 1H, H8), 6.78 (br s, 1H, NH5), 6.86 (d, 2H, *J =* 9.2 Hz, H14), 7.47 (d, 2H, *J =* 9.1 Hz, H13), 11.74 (s, 1H, OH17); ^13^C-NMR (151 MHz, DMSO-*d*_6_, δ ppm) 19.32 (C11), 42.11 (C4), 48.62 (C3), 55.14 (C16), 99.90 (C6), 100.26 (C8), 113.71 (C14), 118.22 (C13), 134.37 (C12), 153.84 (C15), 158.44 (C1), 161.93 (C9), 162.90 (C7), 167.01 (C10); Elemental analysis: calc. for C_16_H_16_N_2_O_5_ (316): C, 60.76; H, 5.10; N, 8.86; found C, 60.87; H, 5.26; N, 8.99.

**1-(3-Chlorophenyl)-4-(4-hydroxy-6-methyl-2-oxo-2*H*-pyran-3-yl)imidazolidin-2-one (5f)**. Beige solid, yield 53%, m.p. 194 °C; IR (ν, cm^−1^): 1598, 1613, 3125, 3221; ^1^H-NMR (400 MHz, DMSO-*d*_6_, δ ppm) 2.17 (s, 3H, H11), 3.74–3.80 (m, 1H, H_a_3), 4.02–4.10 (m, 1H, H_b_3), 4.93–5.04 (m, 1H, H4), 6.01 (s, 1H, H8), 6.95–7.00 (m, 1H, Ar-H), 7.12 (br s, 1H, NH5), 7.27–7.32 (m, 1H, Ar-H), 7.33–7.37 (m, 1H, Ar-H), 7.86 (s, 1H, H13), 11.79 (s, 1H, OH18); ^13^C-NMR (151 MHz, DMSO-*d*_6_, δ ppm) 19.34 (H11), 41.92 (H4), 48.27 (H3), 99.86 (H6), 100.18 (H8), 114.55 (H17), 116.00 (H15), 120.37 (H16), 130.05 (H13), 133.06 (H14), 142.27 (H12), 158.00 (H1), 162.08 (H9), 162.89 (H7), 167.05 (H10); Elemental analysis: calc. for C_15_H_13_ClN_2_O_4_ (320): C, 56.17; H, 4.09; Cl, 11.05; N, 8.73; found C, 56.15; H, 4.14; Cl, 11.05; N, 8.81.

**4-(4-Hydroxy-2-oxo-2*H*-chromen-3-yl)-1-phenylimidazolidin-2-one (6a)**. Beige solid, yield 91%, m.p. 193–194 °C; IR (ν, cm^−1^): 1596, 1614, 3131, 3203; ^1^H-NMR (400 MHz, DMSO-*d*_6_, δ ppm) 3.87–3.95 (m, 1H, H_a_3), 4.08–4.17 (m, 1H, H_b_3), 5.21–5.29 (m, 1H, H4), 6.96 (t, 1H, *J =* 7.3 Hz, Ar-H), 7.04 (br s, 1H, NH5), 7.27–7.33 (m, 2H, Ar-H), 7.36–7.42 (m, 2H, Ar-H), 7.61 (d, 2H, *J =* 7.8 Hz, Ar-H), 7.63–7.67 (m, 1H, Ar-H), 8.04 (dd, 1H, *J =* 7.8, *J =* 1.6, Hz, Ar-H); ^13^C-NMR (151 MHz, DMSO-*d*_6_, δ ppm) 42.77 (C4), 48.47 (C3), 104.51 (C6), 116.10 (C8), 116.29 (C12), 116.53 (C16), 120.97 (C9), 123.51 (C10), 123.92 (C18), 128.43 (C17), 132.51 (C11), 140.80 (C15), 152.32 (C13), 158.34 (C1), 161.03 (C7), 162.09 (C14); Elemental analysis: calc. for C_18_H_14_N_2_O_4_ (322): C, 67.08; H, 4.38; N, 8.69; found C, 67.19; H, 4.50; N, 8.75.

**1-(4-Chlorophenyl)-4-(4-hydroxy-2-oxo-2*H*-chromen-3-yl)imidazolidin-2-one (6h)**. Beige solid, yield 58%, m.p. 199–200 °C; IR (ν, cm^−1^): 1597, 1616, 3284; ^1^H-NMR (400 MHz, DMSO-*d*_6_, δ ppm) 3.85–3.93 (m, 1H, H_a_3), 4.08–4.16 (m, 1H, H_b_3), 5.23–5.31 (m, 1H, H4), 7.15 (br s, 1H, NH5), 7.32–7.35 (m, 2H, Ar-H), 7.36–7.42 (m, 2H, Ar-H), 7.62–7.67 (m, 3H, Ar-H), 8.03 (dd, 1H, *J =* 8.0, *J =* 1.5, Hz, Ar-H); ^13^C-NMR (151 MHz, DMSO-*d*_6_, δ ppm) 42.67 (C4), 48.44 (C3), 104.41 (C6), 116.09 (C8), 116.30 (C12), 117.96 (C17), 123.53 (C9), 123.93 (C10), 124.66 (C18), 128.24 (C16), 132.53 (C11), 139.74 (C15), 152.33 (C13), 158.17 (C1), 161.05 (C7), 162.17 (C14); Elemental analysis: calc. for C_18_H_13_ClN_2_O_4_ (356.5): C, 60.60; H, 3.67; Cl, 9.94; N, 7.85; found C, 60.65; H, 3.64; Cl, 9.91; N, 7.94.

**4-(4-Hydroxy-2-oxo-2*H*-chromen-3-yl)-1-(*p*-tolyl)imidazolidin-2-one (6j)**. Beige solid, yield 68%, m.p. 194–196 °C; IR (ν, cm^−1^): 1598, 1612, 3124, 3244; ^1^H-NMR (400 MHz, DMSO-*d*_6_, δ ppm) 2.25 (s, 3H, H19), 3.84–3.94 (m, 1H, H_a_3), 4.03–4.14 (m, 1H, H_b_3), 5.19–5.29 (m, 1H, H)4, 6.96 (br s, 1H, NH5), 7.10 (d, 2H, *J =* 8.1 Hz, Ar-H), 7.33–7.42 (m, 2H, Ar-H), 7.49 (d, 2H, *J =* 8.3 Hz, Ar-H), 7.65 (t, 1H, *J =* 7.1 Hz, Ar-H), 8.00–8.07 (m, 1H, Ar-H); ^13^C-NMR (151 MHz, DMSO-*d*_6_, δ ppm) 20.75 (C19), 43.41 (C4), 49.12 (C3), 105.03 (C6), 116.66 (C8), 116.82 (C12), 117.18 (C17), 124.04 (C9), 124.45 (C10), 129.38 (C16), 130.31 (C18), 133.02 (C11), 138.94 (C15), 152.87 (C13), 158.94 (C1), 161.55 (C7), 162.61 (C14); Elemental analysis: calc. for C_19_H_16_N_2_O_4_ (336): C, 67.85; H, 4.80; N, 8.33; found C, 68.08; H, 4.96; N, 8.45.

**2-(1-(4-Chlorophenyl)-2-oxoimidazolidin-4-yl)-3-hydroxynaphthalene-1,4-dione (7b)**. Beige solid, yield 86%, m.p. 234–235 °C; IR (ν, cm^−1^): 1598, 1600, 1613, 3154, 3221; ^1^H-NMR (400 MHz, DMSO-*d*_6_, δ ppm) 3.82–3.91 (m, 1H, H_a_3), 4.12–4.21 (m, 1H, H_b_3), 5.15–5.24 (m, 1H, H4), 7.13 (br s, 1H, NH5), 7.34 (d, 2H, *J =* 9.0 Hz, H18), 7.63 (d, 2H, *J =* 9.0 Hz, H17), 7.79–7.88 (m, 2H, Ar-H), 7.98–8.02 (m, 2H, Ar-H); ^13^C-NMR (151 MHz, DMSO-*d*_6_, δ ppm) 41.76 (C4), 48.58 (C3), 117.96 (C18), 120.97 (C6), 124.66 (C13), 125.72 (C9), 125.81 (C12), 128.26 (C17), 129.77 (C8), 132.02 (C19), 133.24 (C11), 134.88 (C10), 139.74 (C16), 156.86 (C15), 158.12 (C1), 181.19 (C14), 183.39 (C7); Elemental analysis: calc. for C_19_H_13_ClN_2_O_4_ (368.5): C, 61.88; H, 3.55; Cl, 9.61; N, 7.60; found C, 62.09; H, 3.77; Cl, 9.57; N, 7.47.

**2-Hydroxy-3-(2-oxo-1-(*p*-tolyl)imidazolidin-4-yl)naphthalene-1,4-dione (7c)**. Beige solid, yield 60%, m.p. 223–224 °C; IR (ν, cm^−1^): 1597, 1600, 1622, 3133, 3287; ^1^H-NMR (400 MHz, DMSO-*d*_6_, δ ppm) 2.25 (s, 3H, H20), 3.82–3.90 (m, 1H, H_a_3), 4.08–4.19 (m, 1H, H_b_3), 5.15–5.23 (m, 1H, H4), 6.94 (br s, 1H, NH5), 7.10 (d, 2H, *J =* 8.3 Hz, H18), 7.48 (d, 2H, *J =* 8.6 Hz, H17), 7.79–7.83 (m, 1H, Ar-H), 7.85–7.90 (m, 1H, Ar-H), 7.97–8.04 (m, 2H, Ar-H); ^13^C-NMR (151 MHz, DMSO-*d*_6_, δ ppm) 20.22 (C20), 41.89 (C4), 48.71 (C3), 116.63 (C18), 120.51 (C13), 121.18 (C6), 125.70 (C9), 125.81 (C12), 128.85 (C17), 129.78 (C8), 132.02 (C11), 133.23 (C10), 134.88 (C19), 138.39 (C16), 156.74 (C15), 158.37 (C1), 181.21 (C14), 183.44 (C7); Elemental analysis: calc. for C_20_H_16_N_2_O_4_ (348): C, 68.96; H, 4.63; N, 8.04; found C, 69.15; H, 4.73; N, 8.13.

**1-(3-Chlorophenyl)-4-(4-hydroxy-2-oxo-2*H*-chromen-3-yl)imidazolidin-2-one (7f)**. Beige solid, yield 83%, m.p. 199 °C; IR (ν, cm^−1^): 1594, 1618, 3176, 3293; ^1^H-NMR (400 MHz, DMSO-*d*_6_, δ ppm) 3.85–3.95 (m, 1H, H_a_3), 4.08–4.19 (m, 1H, H_b_3), 5.22–5.30 (m, 1H, H4), 6.98–7.02 (m, 1H, Ar-H), 7.22 (br s, 1H, NH5), 7.29–7.33 (m, 1H, Ar-H), 7.36–7.42 (m, 3H, Ar-H), 7.62–7.67 (m, 1H, Ar-H), 7.89 (s, 1H, Ar-H), 8.01–8.06 (m, 1H, Ar-H); ^13^C-NMR (151 MHz, DMSO-*d*_6_, δ ppm) 42.65 (C4), 48.42 (C3), 104.40 (C6), 114.61 (C19), 116.05 (C8), 116.10 (C12), 116.31 (C18), 120.50 (C20), 123.54 (C9), 123.93 (C10), 130.09 (C17), 132.54 (C11), 133.10 (C16), 142.19 (C15), 152.33 (C13), 158.10 (C1), 161.05 (C7), 162.19 (C14); Elemental analysis: calc. for C_18_H_13_ClN_2_O_4_ (356.5): C, 60.60; H, 3.67; Cl, 9.94; N, 7.85; found C, 60.78; H, 3.81; Cl, 10.09; N, 7.86.

**4,4′-(4,6-Dihydroxy-5-methyl-1,3-phenylene)bis(1-(4-chlorophenyl)imidazolidin-2-one) (8).** Beige solid, yield 16%, m.p. > 250 °C; IR (ν, cm^−1^): 1594, 1627, 3089, 3171, 3276; ^1^H-NMR (400 MHz, DMSO-*d*_6_, δ ppm) 2.10 (s, 3H, H10), 3.42–3.51 (m, 2H, H_a_3), 4.21–4.30 (m, 2H, H_b_3), 5.00–5.08 (m, 2H, H4), 7.16 (s, 1H, H9), 7.32 (d, 4H, *J =* 8.9 Hz, H13), 7.52 (br s, 2H, NH5), 7.59 (d, 4H, *J =* 8.9 Hz, H12), 7.50 (s, 2H, OH15); ^13^C-NMR (151 MHz, DMSO-*d*_6_, δ ppm) 10.20 (C10), 47.62 (C4), 52.76 (C3), 113.55 (C8), 118.87 (C13), 120.73 (C6), 121.45 (C14), 125.61 (C9), 128.82 (C12), 140.09 (C11), 152.68 (C7), 158.73 (C1); Elemental analysis: calc. for C_25_H_22_Cl_2_N_4_O_4_ (513): C, 58.49; H, 4.32; Cl, 13.81; N, 10.91; found C, 58.78; H, 4.58; Cl, 13.99; N, 11.09.

### 3.2. Biological Studies

Cytotoxic effects of the test compounds on human cancer and normal cells were estimated by means of the multifunctional Cytell Cell Imaging system (GE Health Care Life Science, Sweden) using the Cell Viability Bio App, which precisely counts the number of cells and evaluates their viability from fluorescence intensity [28]. DAPI and propidium iodide purchased from Sigma were used to detect cell viability. IC_50_ was calculated using an online tool: “Quest Graph™ IC50 Calculator.” AAT Bioquest, Inc, https://www.aatbio.com/tools/ic50-calculator, accessed on 6 April 2021. The M-HeLa clone 11 human, epithelioid cervical carcinoma, strain of HeLa, clone of M-HeLa; human duodenal cancer cell line (HuTu 80) from the Type Culture Collection of the Institute of Cytology (Russian Academy of Sciences) and Chang liver cell line (Human liver cells) from the N. F. Gamaleya Research Center of Epidemiology and Microbiology were used in the experiments. The cells were cultured in a standard Eagle’s nutrient medium manufactured at the Chumakov Institute of Poliomyelitis and Virus Encephalitis (PanEco company) and supplemented with 10% fetal calf serum and 1% nonessential amino acids. The cells were plated into a 96-well plate (Nanc) at a concentration of 1 × 10^5^ cells/mL, 150 μL of medium per well, and cultured in a CO_2_ incubator at 37 °C. Twenty-four hours after seeding the cells into wells, the compound under study was added at a preset dilution, 150 μL to each well. The dilutions of the compounds were prepared immediately in nutrient media; 5% DMSO, which does not induce inhibition of cells at this concentration, was added for better solubility. The experiments were repeated three times. Intact cells cultured in parallel with experimental cells were used as a control.

### 3.3. Quantum Chemistry Calculations

All calculations have been performed with the Gaussian 16 package [29]. The initial structures were fully optimized at the B3LYP/6-311++G(d,p) theory level. All optimizations were followed by frequency calculations at the same level of theory in order to check that optimized structures really correspond to true minima.

## 4. Conclusions

In conclusion, a series of novel 4-(het) arylimidazolidines were obtained via trifluoroacetic acid catalyzed intramolecular cyclization/Mannich-type reaction of *N*-(2,2-diethoxyethyl) ureas with high regioselectivities. The plausible mechanism was discussed using quantum chemistry calculations and the choice of the solvent and catalyst amount was found to have a crucial effect on the regioselectivity. The anti-cancer activities of the obtained compounds were tested in vitro, and the substitution of the nitrogen atom was identified as one the key factors influencing the cytotoxicity against normal and cancer human cell lines.

## Figures and Tables

**Table 1:** Cytotoxic effects of imidazodin-2-ones **2** on the cancer and normal human cell lines ^1^.

Test Compounds	Cancer Cell Lines				Normal Cell Line
	M-HeLa		HuTu 80		Chang Liver
	IC_50_ (µM)	SI	IC_50_ (µM)	SI	IC_50_ (µM)
**2h**	93.6 ± 8.2	1	56b ± 5	1.7	98.7 ± 8.8
**2j**	>100	–	>100	–	>100
**2k**	59.5 ± 4.8	1.1	56.8 ± 4.5	1.1	63.2 ± 5.6
**2l**	96.3 ± 8.2	–	>100	–	>100
**2a**	>100	–	>100	–	95.6 ± 8.2
**2b**	>100	–	>100	–	87.7 ± 7.2
**2c**	>100	–	>100	–	58.4 ± 4.7
**2d**	>100	–	>100	–	>100
**2e**	>100	–	>100	–	>100
**4h**	98.7 ± 8.7	–	>100	–	>100
**4j**	90.7 ± 7.8	–	>100	–	>100
**4k**	94.7 ± 8.8	–	>100	–	85.8 ± 6.9
**4l**	97 ± 8.6	–	>100	–	>100
**4c**	>100	–	>100	–	>100
**4d**	>100	–	>100	–	>100
**5b**	>100	–	>100	–	>100
**5c**	>100	–	>100	–	>100
**5d**	>100	–	>100	–	>100
**6j**	>100	–	>100	–	>100
**7c**	>100	–	>100	–	>100
**Tamoxifen**	28.0 ± 2.5	1.6	NA	–	46.2 ± 3.5
**Arglabin**	41.6 ± 3.2	0.6	22.7 ± 1.8	1.2	26.3 ± 2.1

^1^ Three independent experiments were carried out—means no selectivity; NA means no activity.

## Data Availability

Not applicable.

## Supplementary Materials

The following are available online. Figure S1: Bifurcate CH…O interaction in the crystals of the studied compounds, Table S1: CH…O interactions in crystals of investigated compounds, Figures S2–S98: copies of the NMR spectra of all synthesized compounds.

## Sample Availability

Samples of the compounds **2**–**7** are available from the authors.
