# Supplementary material for: The Highly Regioselective Synthesis of Novel Imidazolidin-2-Ones via the Intramolecular Cyclization/Electrophilic Substitution of Urea Derivatives and the Evaluation of Their Anticancer Activity
Source: Molecules. 2021 Jul 22;26(15):4432. doi: 10.3390/molecules26154432 (PMC8347191; doi:10.3390/molecules26154432)
Supplement: Supplementary file 1 [file molecules-26-04432-s001.zip › molecules-1297956-supplementary.pdf]

SUPPLEMENTARY MATERIALS

The highly regioselective synthesis of novel imidazolidin-2-ones via the intramolecular cyclization / electrophilic substitution of urea derivatives and evaluation of their anticancer activity

Almir S. Gazizov,[a]* Andrey V. Smolobochkin,[a] Dinara S. Abdullaeva,[b] Alexander R. Burilov,[a] Michail A. Pudovik,[a] Alexandra D. Voloshina,[a] Victor V. Syakaev,[a] Anna P. Lyubina,[a] Syumbelya K. Amerhanova[a] and Julia K. Voronina[c,d]

[a] *Arbuzov Institute of Organic and Physical Chemistry, FRC Kazan Scientific Center, Russian Academy of Sciences, 420088, Arbuzova str., 8, Kazan, Russian Federation*
[b] *Kazan Federal University, 420008, Kremlyovskaya str., 18, Kazan, Russian Federation*
[c] *N.S. Kurnakov Institute of General and Inorganic Chemistry, Russian Academy of Sciences, Leninsky Ave., 31, 119991 Moscow, Russian Federation*
[d] *G.V. Plekhanov Russian University of Economics, Stremyanny Per., 36, 117997 Moscow, Russian Federation*

Contents

X-ray studies 2
Copies of NMR spectra 4

X-ray studies

Experimental. The X-ray diffraction data for the crystals of **2j** and **2k** were collected on a Bruker D8 Venture automatic diffractometer using graphite monochromated radiation. The structures were solved by direct methods and refined by full-matrix least-squares using the SHELXL97[1] program. All the non-hydrogen atoms were refined with anisotropic atomic displacement parameters. All figures were made using the program OLEX2[2]. Crystallographic data for the structure reported in this paper have been deposited with the Cambridge Crystallographic Data Center (2068964-2068965).

Crystal data for **2j**: $C_{22}H_{24}N_4O_2$, M = 376.45, monoclinic, space group $P2_1/n$, Z = 4, a = 7.3485(4), b = 21.8110(10), c = 12.3569(6)Å, β = 104.398(2)° , V = 1918.34(17)$Å^3$, ρ_{calc} = 1.303 g/cm^3, μ =0.086 mm^{-1}, 15151 reflections collected (±h, ±k, ±l), 3768 independent (R_{int} 0.0387) and 3013 observed reflections [I ≥ 2\s (I)], 257 refined parameters, R_1 =0.0457, wR_2 = 0.1049, max. residual electron density is 0.267 (-0.231) e$Å^{-3}$.

Crystal data for **2k**: $C_{22}H_{24}N_4O_3$, M = 392.45, monoclinic, space group $P2_1/c$, Z = 4, a = 7.5009(5), b = 21.8592(14), c = 12.5236(9)Å, β = 107.237(3)° , V = 1961.2(2)$Å^3$, ρ_{calc} = 1.329 g/cm^3, μ =0.091 mm^{-1}, 19745 reflections collected (±h, ±k, ±l), 5225 independent (R_{int} 0.0637) and 3528 observed reflections [I ≥ 2\s (I)], 266refined parameters, R_1 =0.0479, wR_2 = 0.1170, max. residual electron density is 0.258 (-0.249) e$Å^{-3}$.

The crystals of compounds **2j** and **2k** were obtained by recrystallization from dimethylsulfoxide. X-ray investigations revealed that the bond lengths, valence and torsion angles in molecules of the studied compound have values that are within standard for each type of chemical bond. Moreover, the geometries of the molecules of the studied compounds are identical within the experimental error. sp^3-Hybridization of C4 and C5 atoms results in the twisted geometry of molecules.

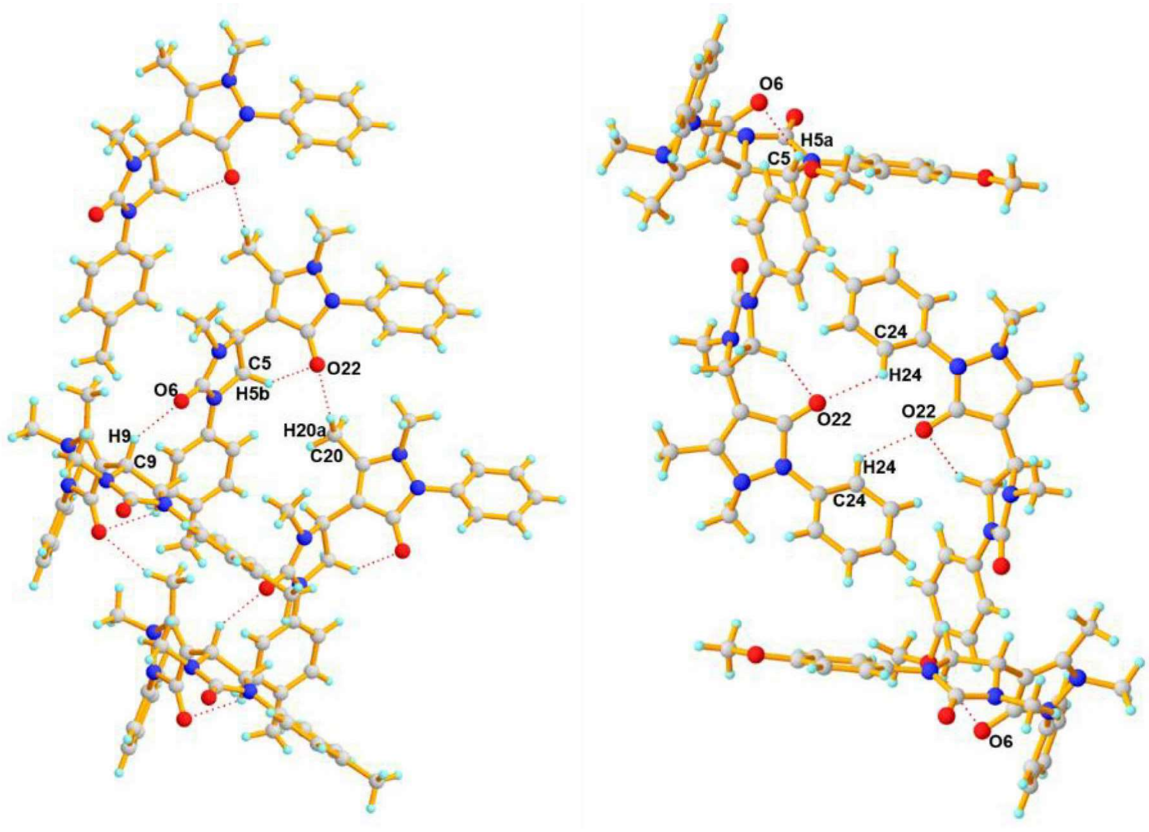

Figure S 1 Bifurcate CH...O interaction in the crystals of the studied compounds

The absence of a classical H-donors in the molecules of investigated compounds leads to the fact that the crystal packing is formed only by CH...O (Table S 1) and CH...π interactions. Interestingly, the appearance of an additional oxygen atom in the **2j** molecule does not significantly change the packing only slightly increasing

its density. Large number of CH-donors and a limited number of acceptors leads to the implementation of bifurcate CH...O interactions (Figure S 1). Crystals of investigated compounds consists of dimers, bound in infinite layers parallel to the 0a axis.

Table S 1 CH...O interactions in crystals of investigated compounds

H-bond	D-H	H...A	D...A	D-H...A
1095 **2j**				
C5-H5B...O22	0.99	2.42	3.149(2)	130
C9-H9...O6	0.95	2.33	2.906(2)	118
C20-H20A...O22	0.98	2.28	3.211(2)	158
C28-H28...O22	0.95	2.43	3.160(2)	134
1054 **2k**				
C5-H5B...O22	0.99	2.40	3.1321(19)	130
C9-H9...O6	0.95	2.39	2.917(2)	114
C20-H20A...O22’	0.98	2.47	3.388(2)	157
C20-H20C...O14	0.98	2.51	3.432(2)	157
C24-H24...O22	0.95	2.59	2.9702(19)	104
C24-H24...O22”	0.95	2.45	3.090(2)	125
C25-H25...O22”	0.95	2.60	3.162(2)	119

Copies of NMR spectra

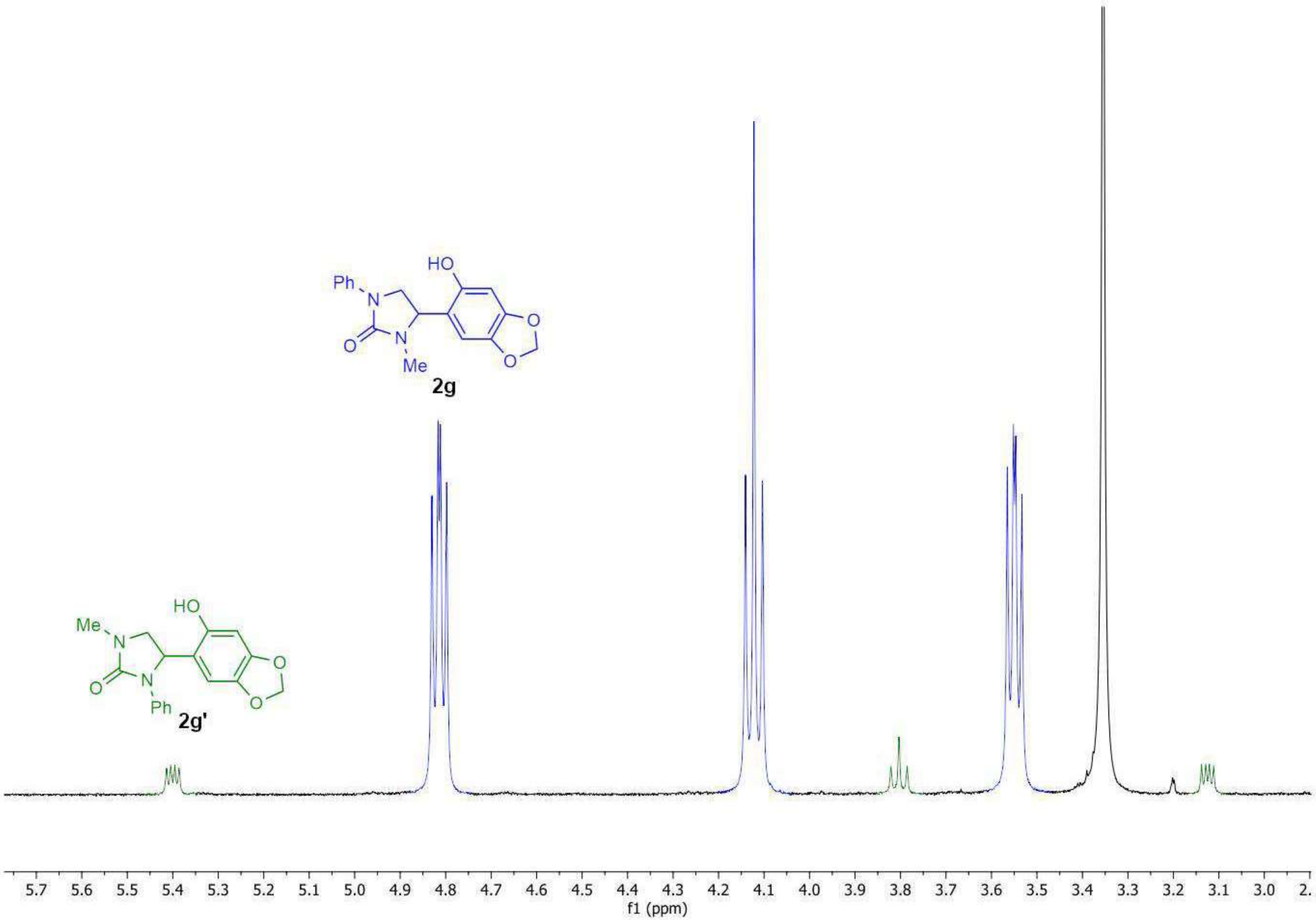

Figure S 2 Fragment of the ^{1}H NMR spectrum (DMSO-d_6, 400 MHz, 303K) of the mixture of regioisomers **2g** and **2g'** (see Smolobochkin A.V., Gazizov A.S., Otegen N.K., Voronina J.K., Strelnik A.G., Samigullina A.I., Burilov A.R., Pudovik M.A. *Synthesis*, **2020**, 52, 3263–3271, DOI: 10.1055/s-0040-1707864) for detailed structure elucidation)

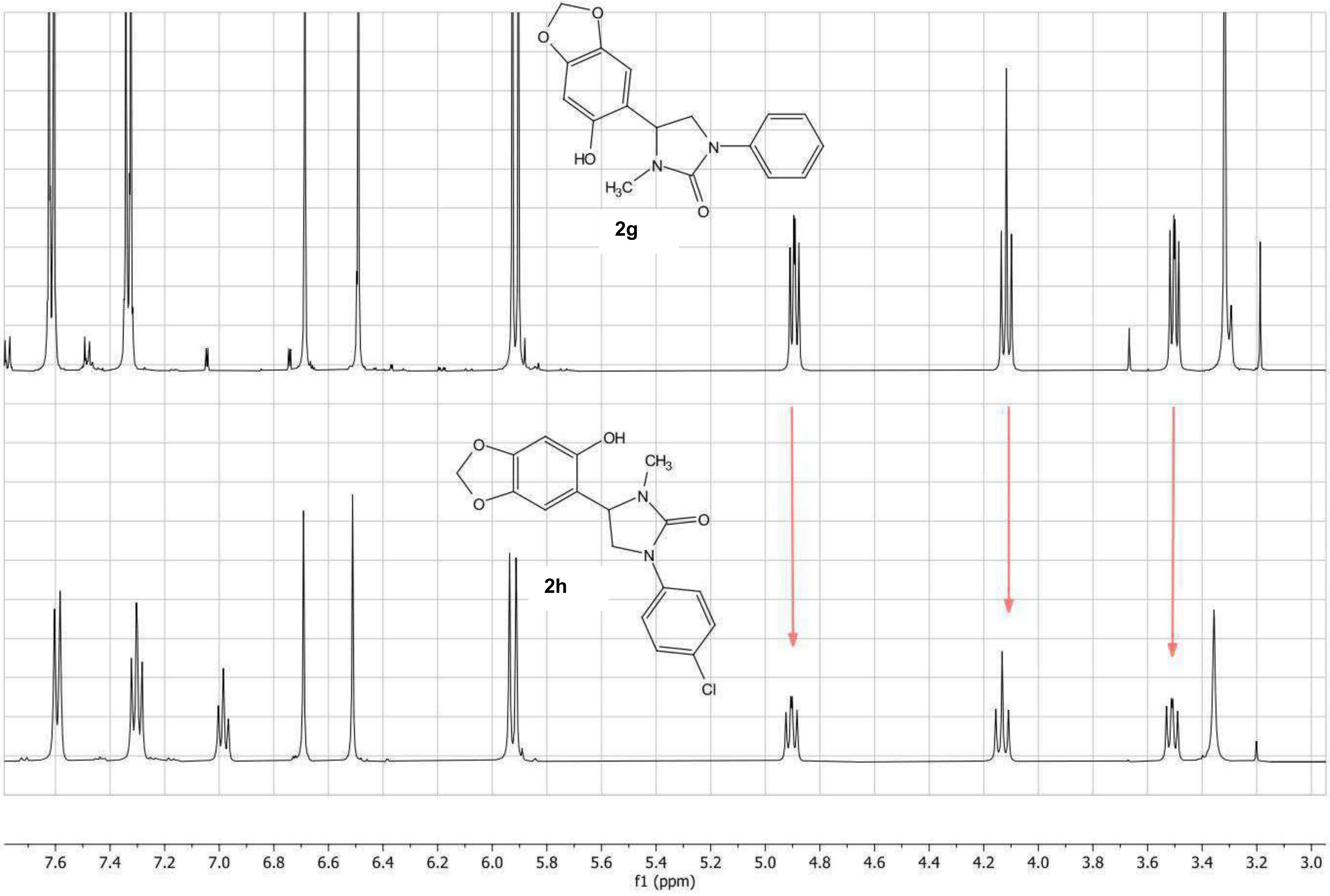

Figure S 3 Comparison of the ^{1}H NMR spectra of the compounds **2g** and **2h** (DMSO-d_6, 400 MHz, 303K)

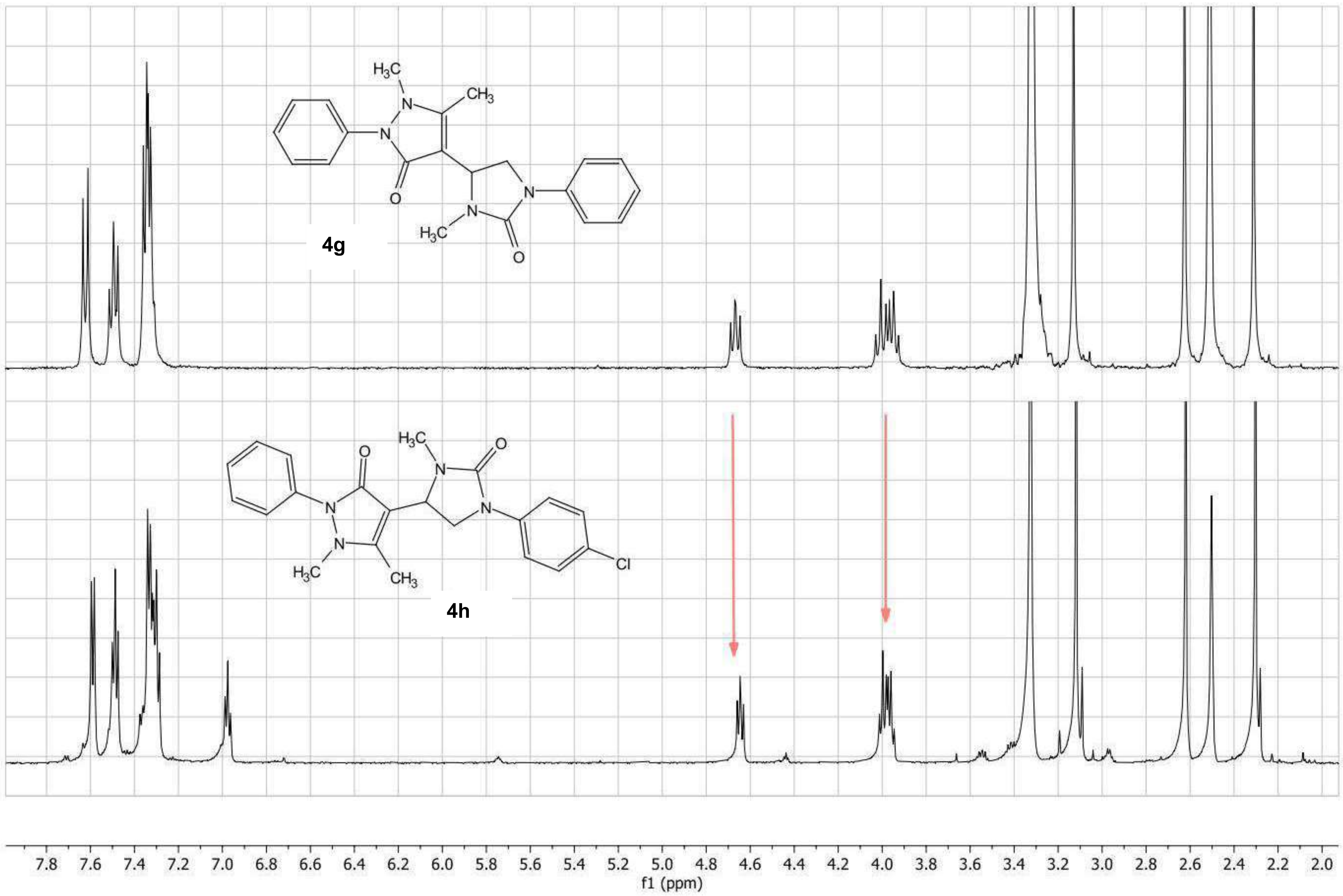

Figure S 4 Comparison of the ^{1}H NMR spectra of the compounds **4g** and **4h** (DMSO-d_6, 400 MHz, 303K)

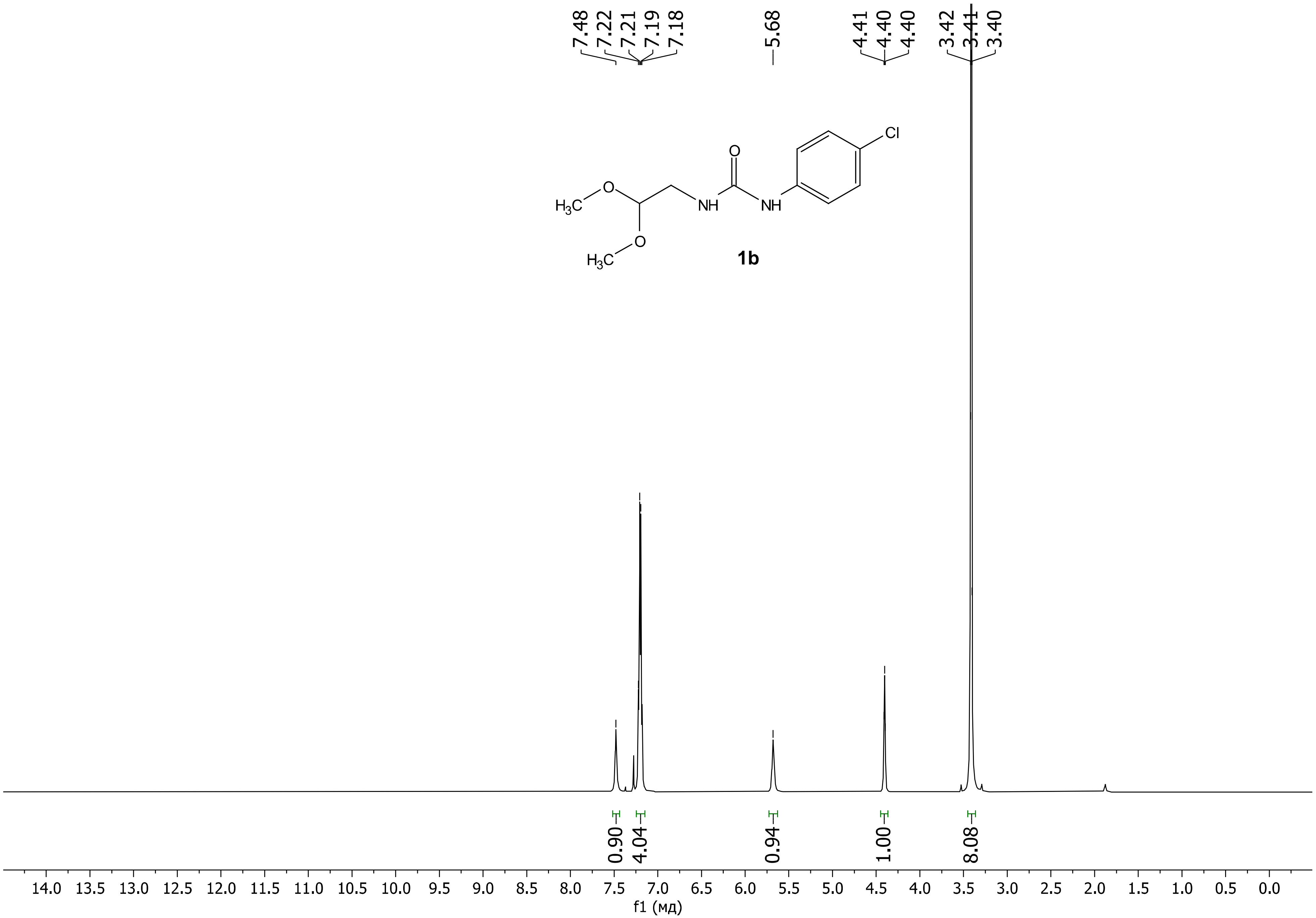

Figure S5. ^{1}H NMR spectrum ($CDCl_3$, 400MHz, 303K) of the compound **1b**

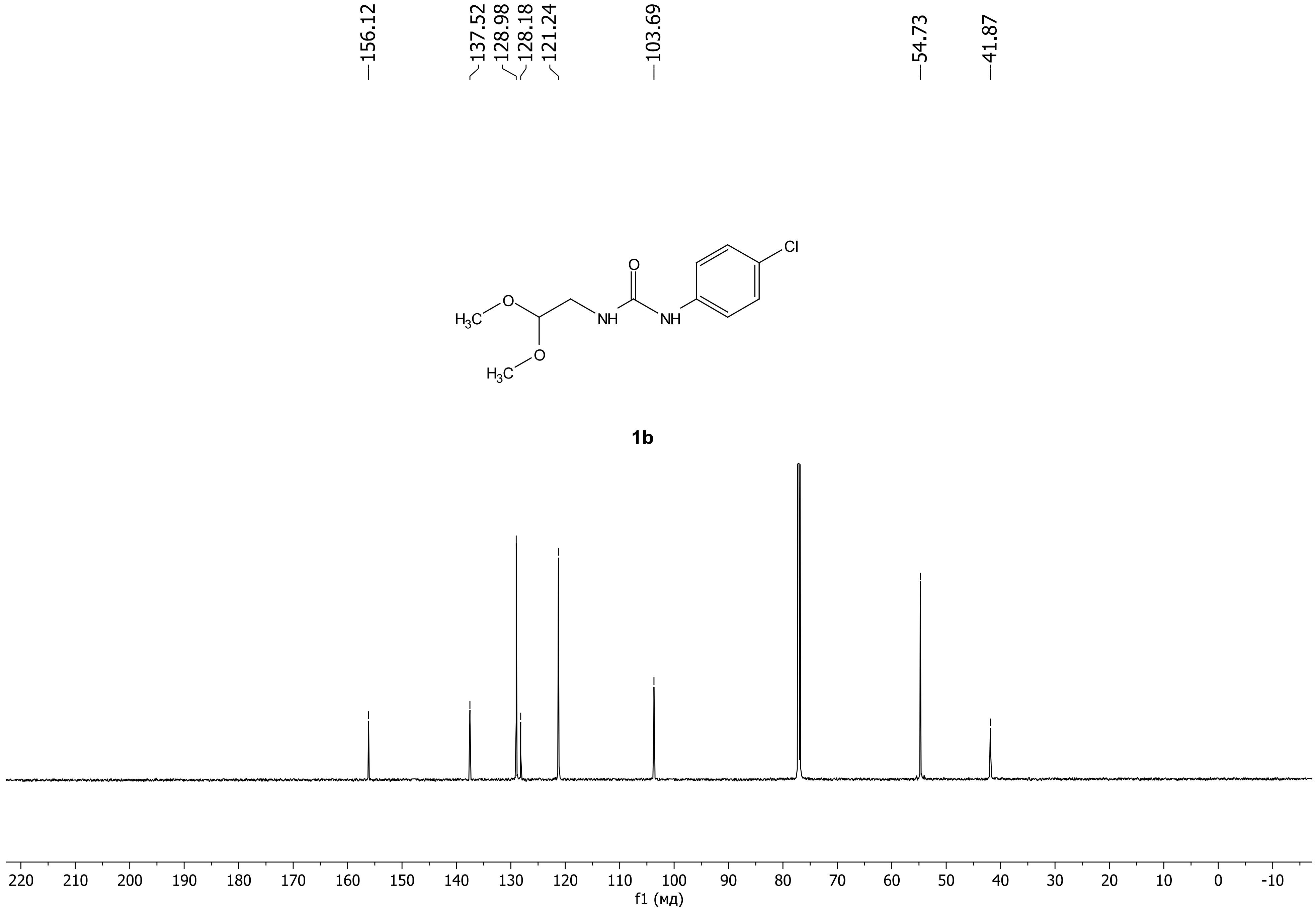

1b

Figure S6. ^{13}C NMR spectrum ($CDCl_3$, 151MHz, 303K) of the compound **1b**

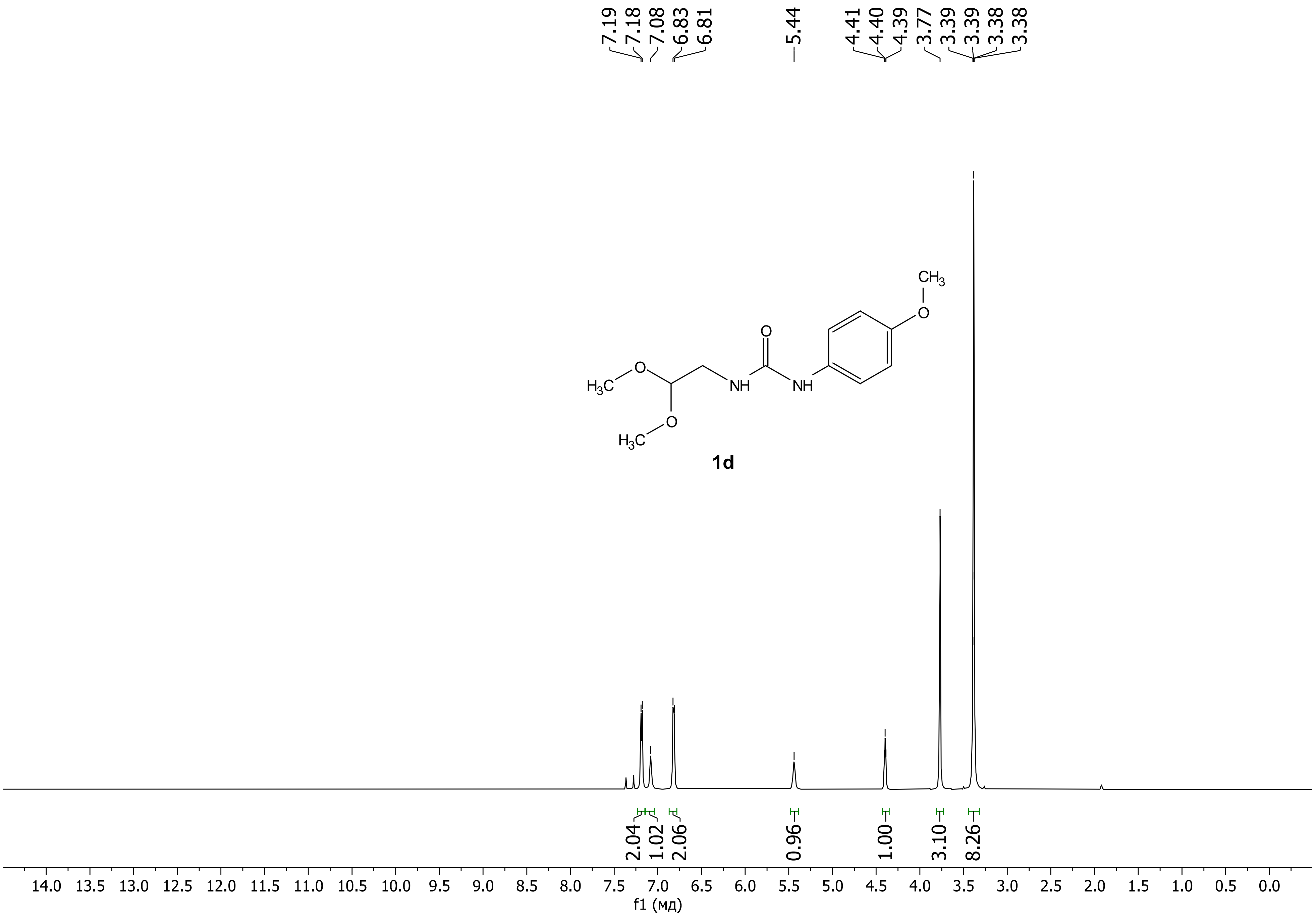

Figure S7. ^{1}H NMR spectrum ($CDCl_3$, 400MHz, 303K) of the compound **1d**

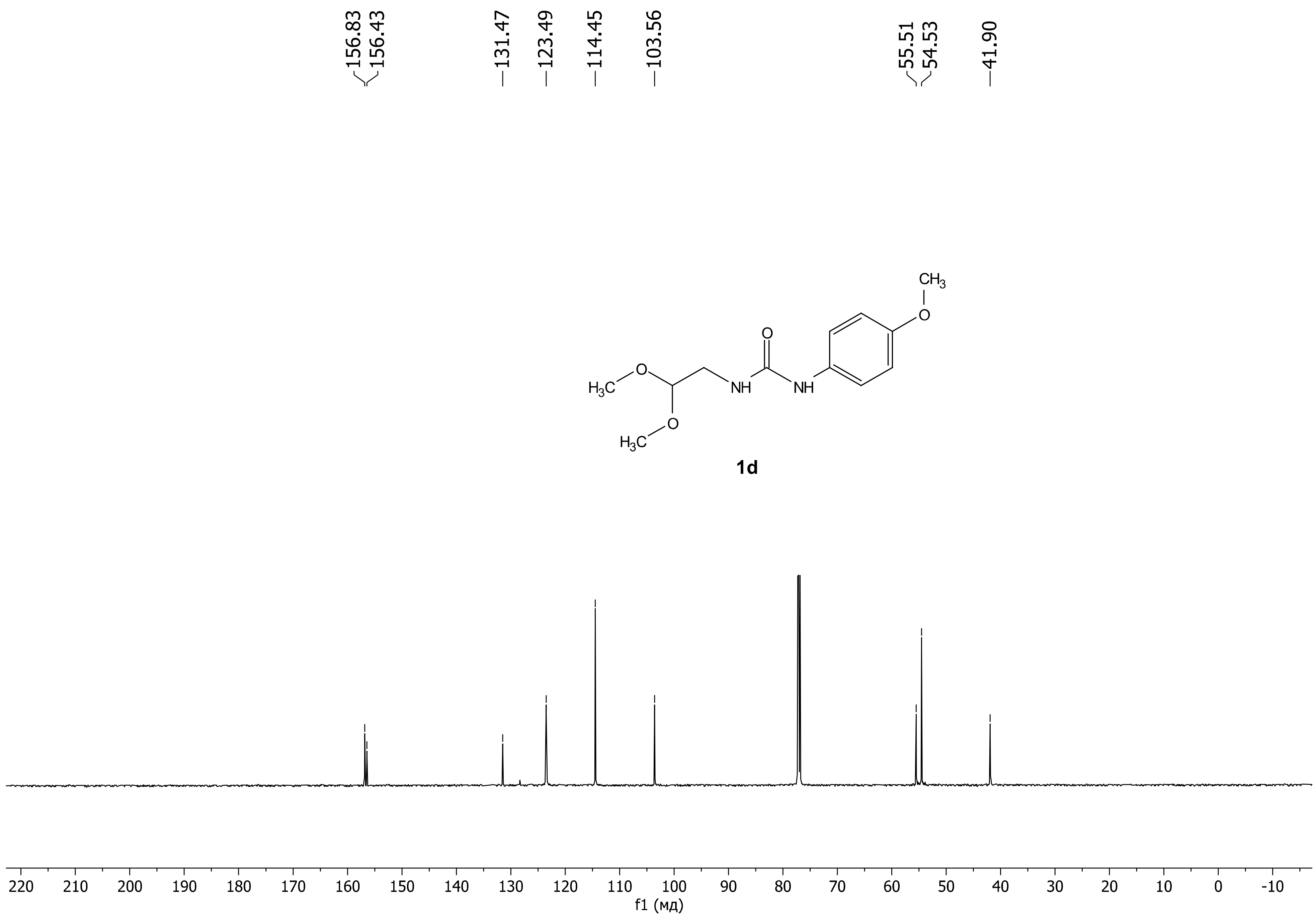

Figure S8. ^{13}C NMR spectrum ($CDCl_3$, 151MHz, 303K) of the compound **1d**

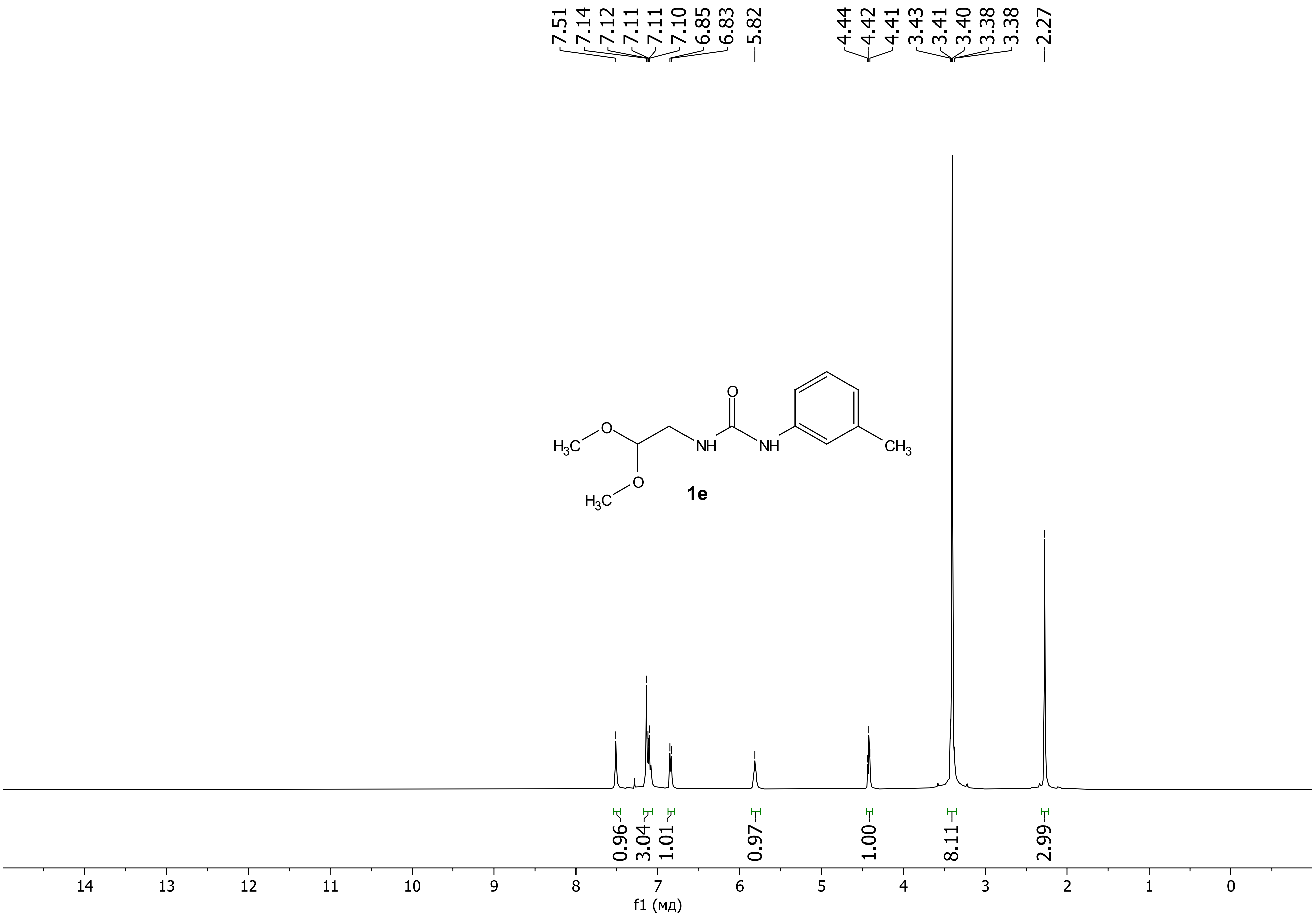

Figure S9. ^{1}H NMR spectrum ($CDCl_3$, 400MHz, 303K) of the compound **1e**

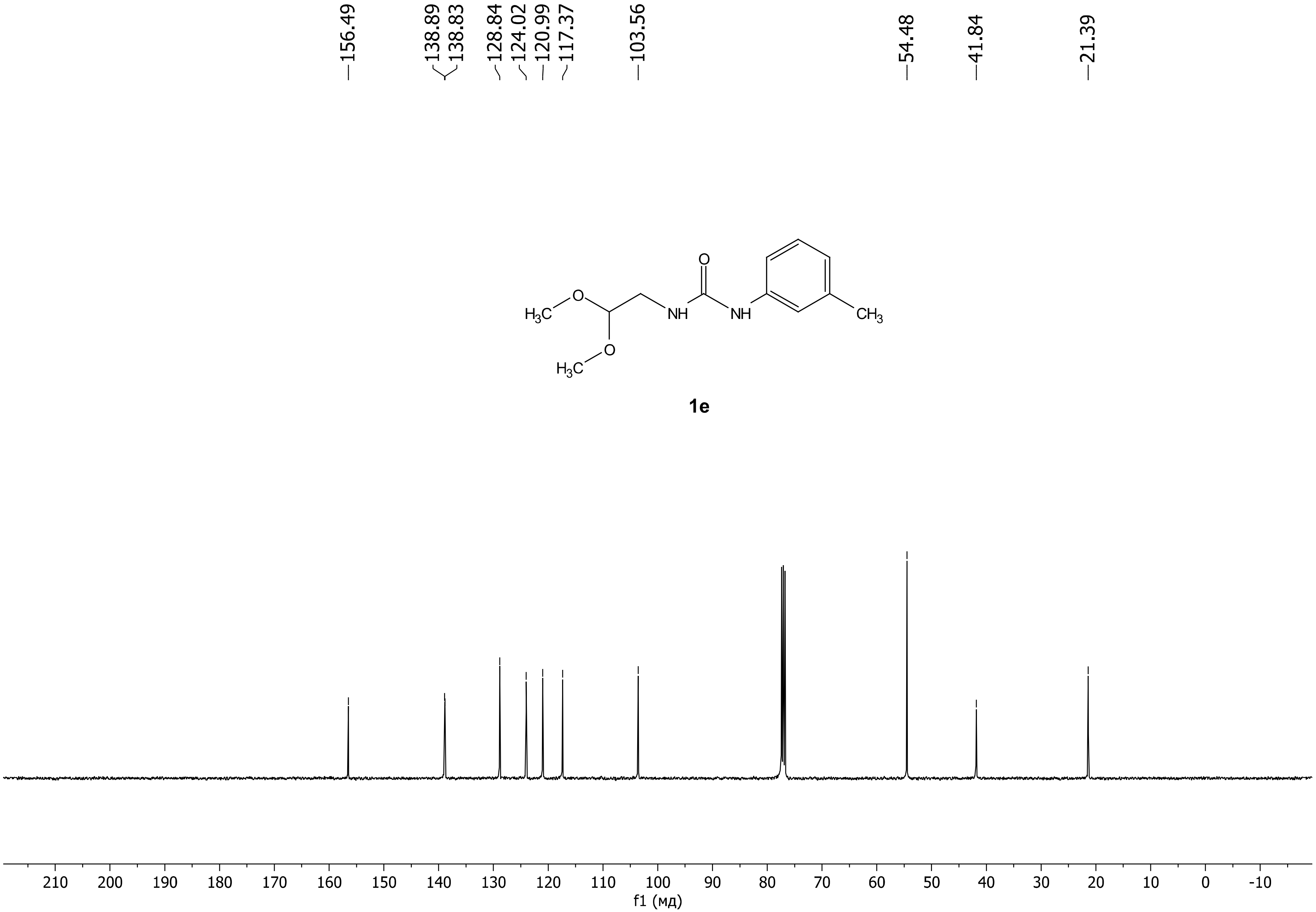

Figure S10. ^{13}C NMR spectrum ($CDCl_3$, 151MHz, 303K) of the compound **1e**

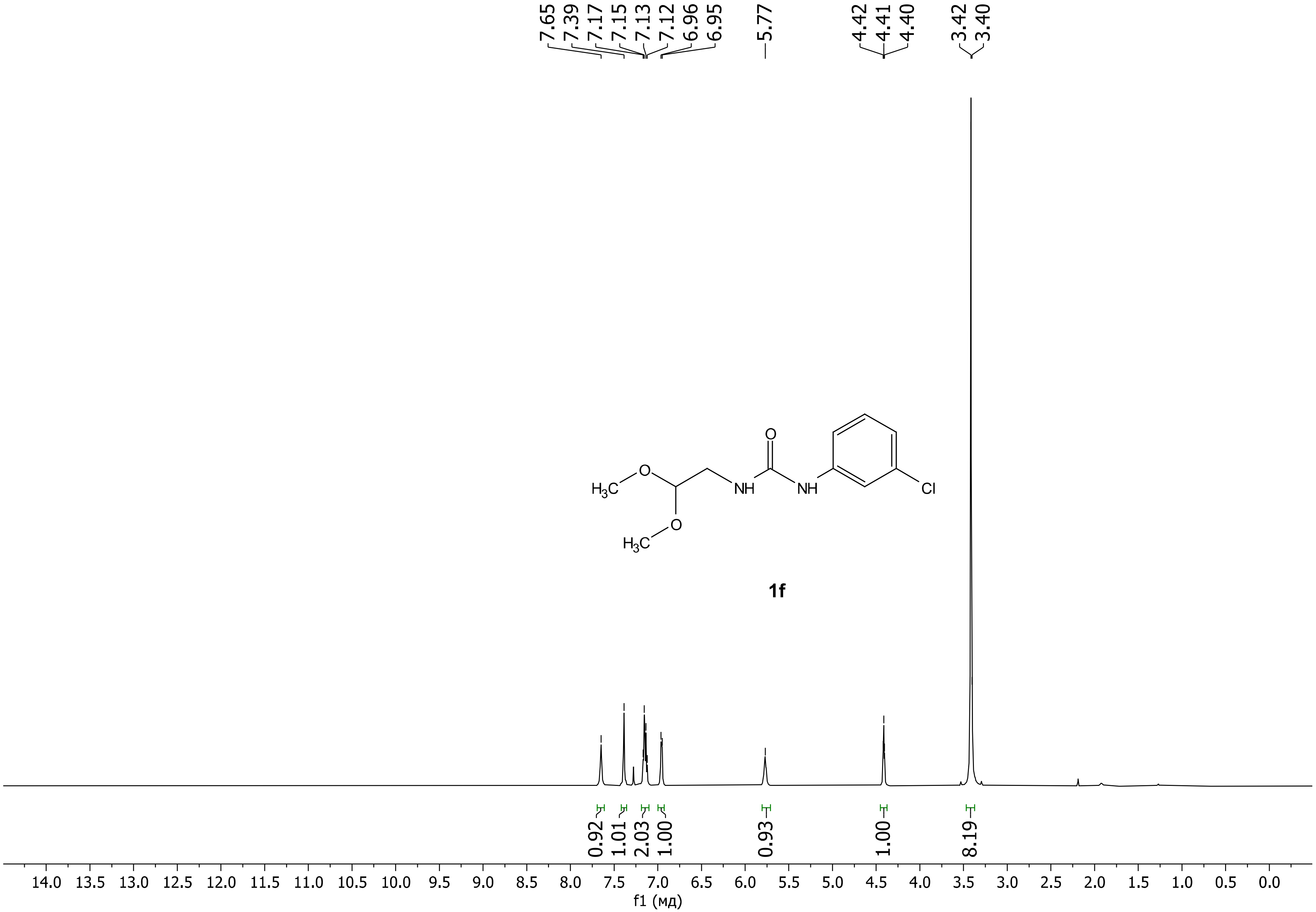

Figure S11. ^{1}H NMR spectrum ($CDCl_3$, 400MHz, 303K) of the compound **1f**

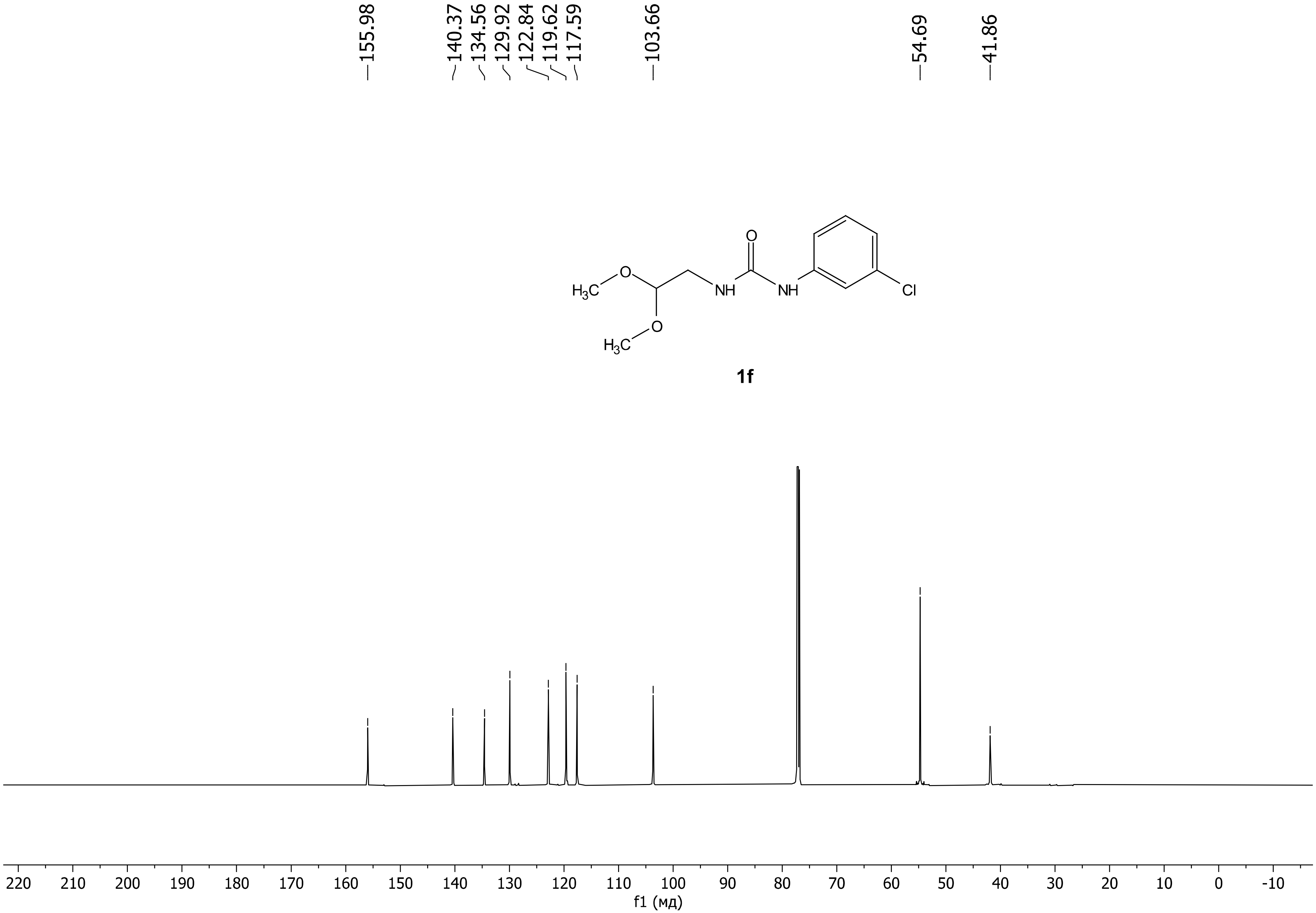

1f

Figure S12. ^{13}C NMR spectrum ($CDCl_3$, 151MHz, 303K) of the compound **1f**

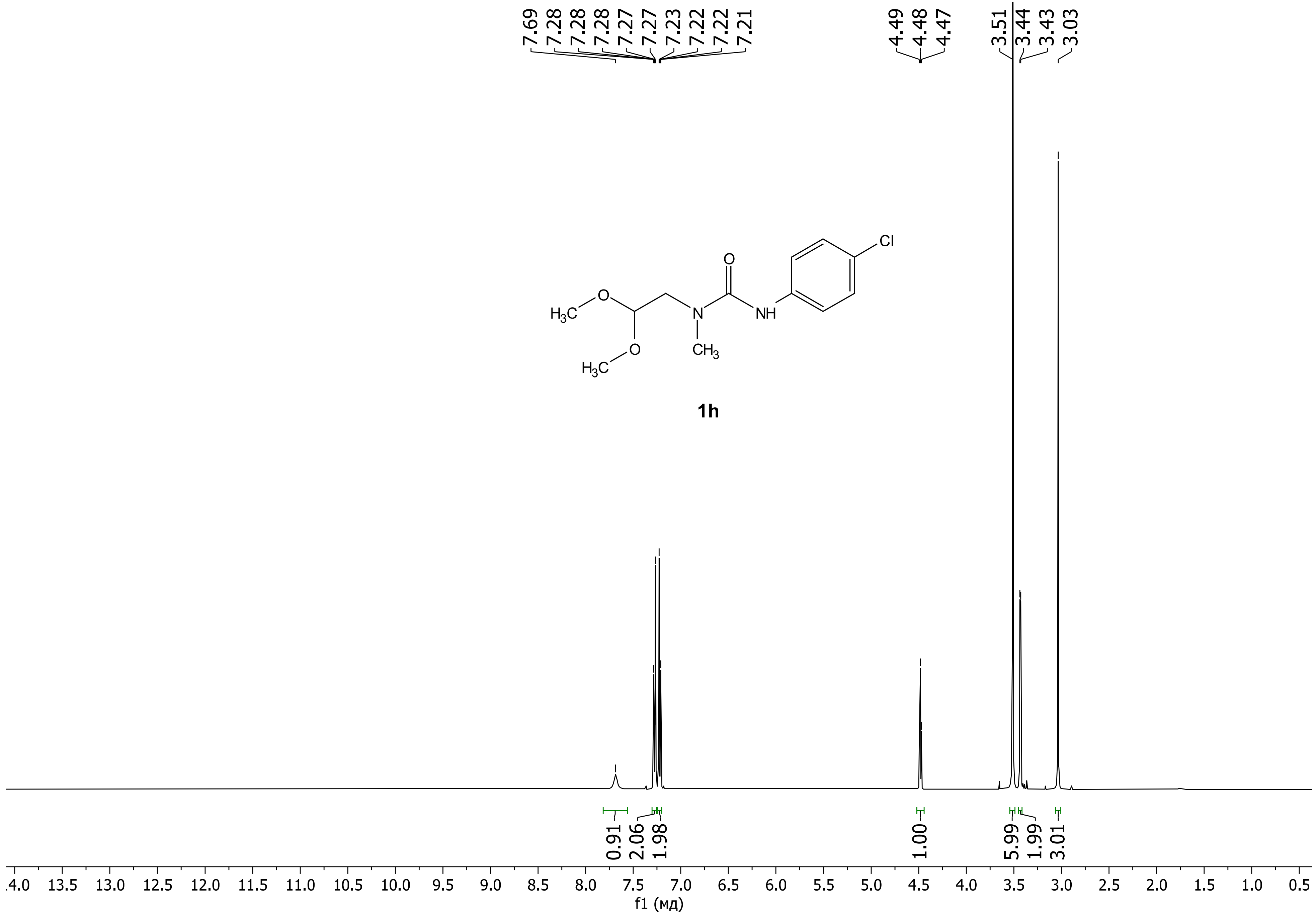

Figure S13. ^{1}H NMR spectrum ($CDCl_3$, 400MHz, 303K) of the compound **1h**

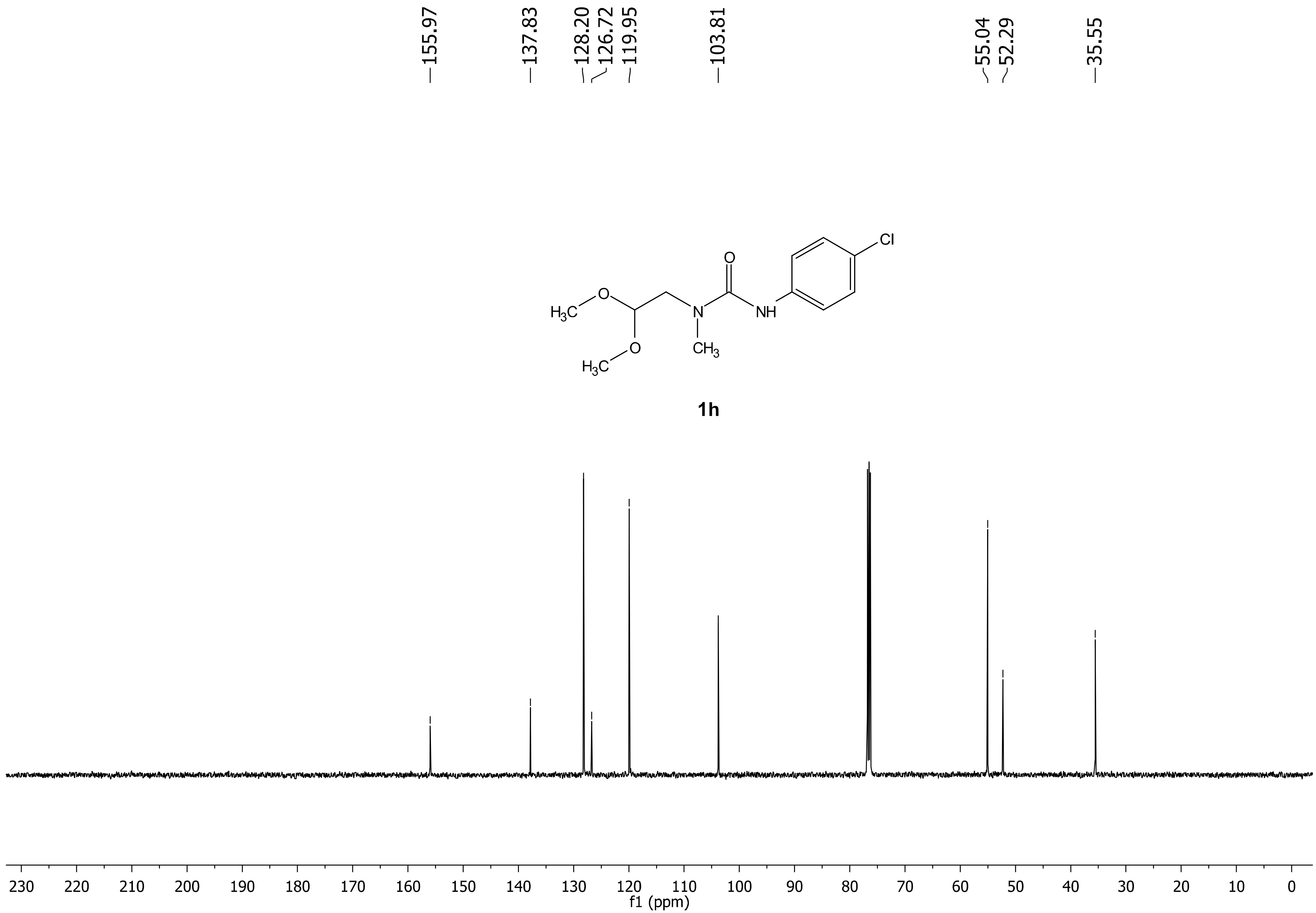

Figure S14. ${}^{13}C$ NMR spectrum ($CDCl_3$, 151MHz, 303K) of the compound **1h**

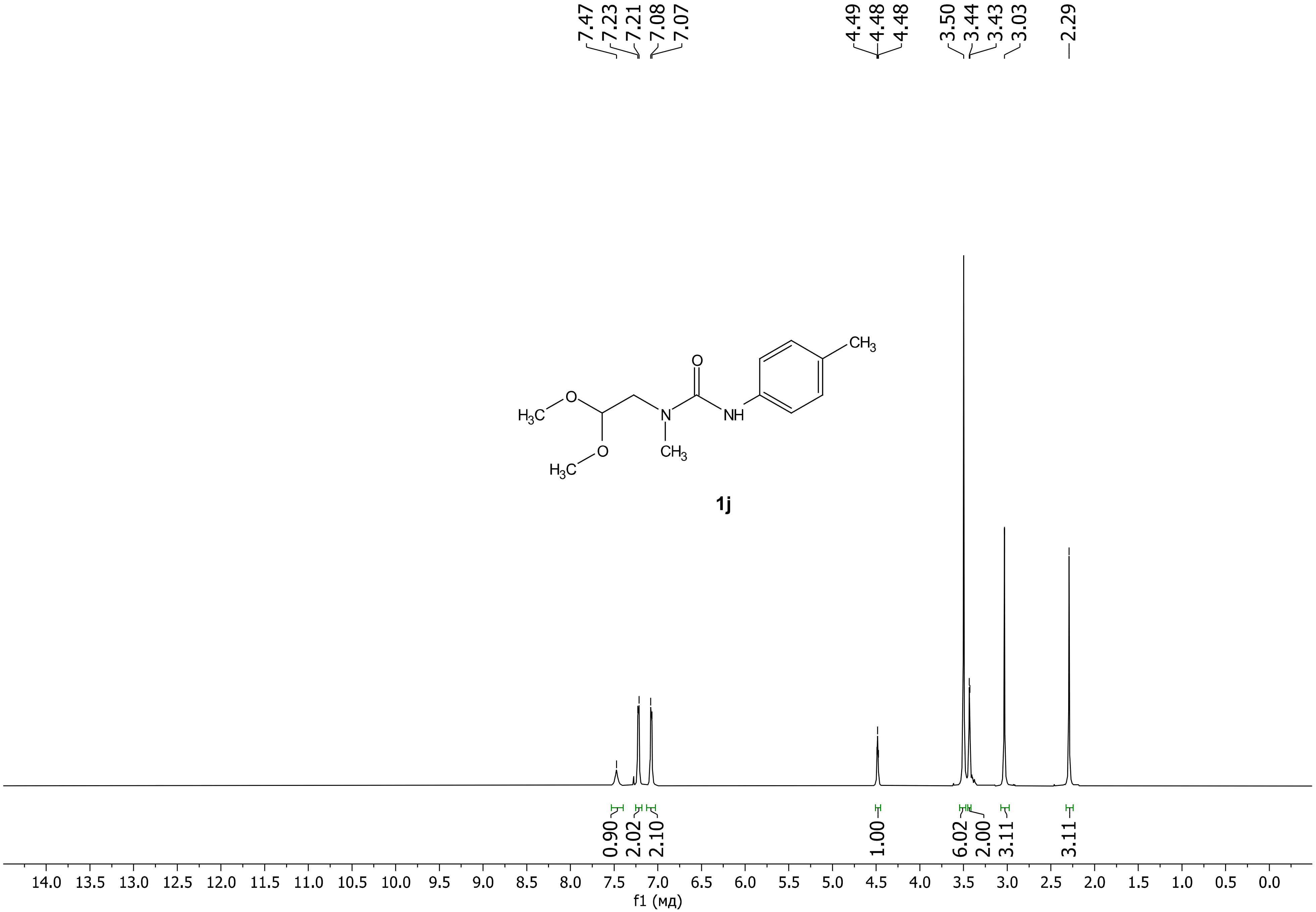

Figure S15. ^{1}H NMR spectrum ($CDCl_3$, 400MHz, 303K) of the compound **1j**

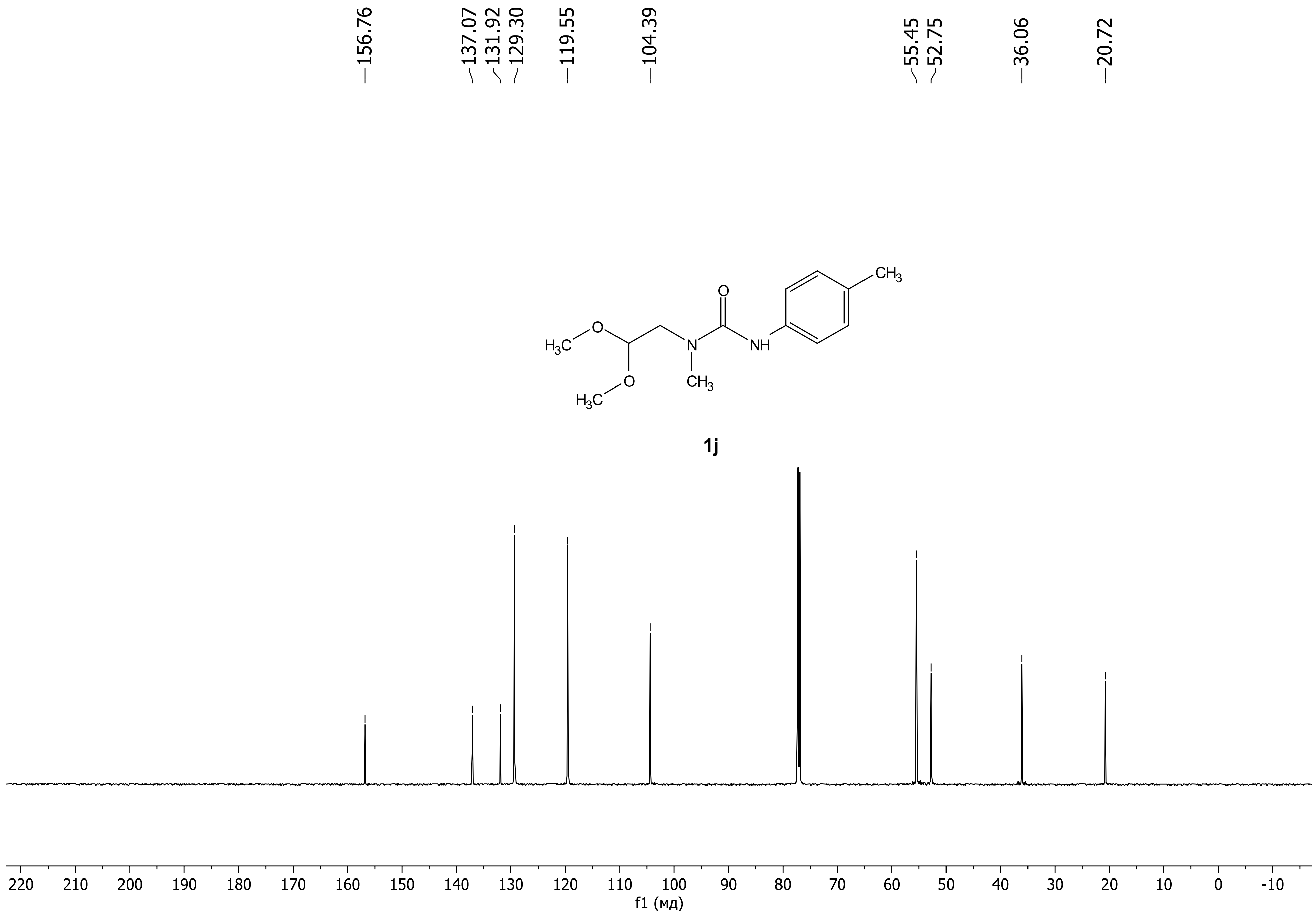

Figure S16. ^{13}C NMR spectrum ($CDCl_3$, 151MHz, 303K) of the compound **1j**

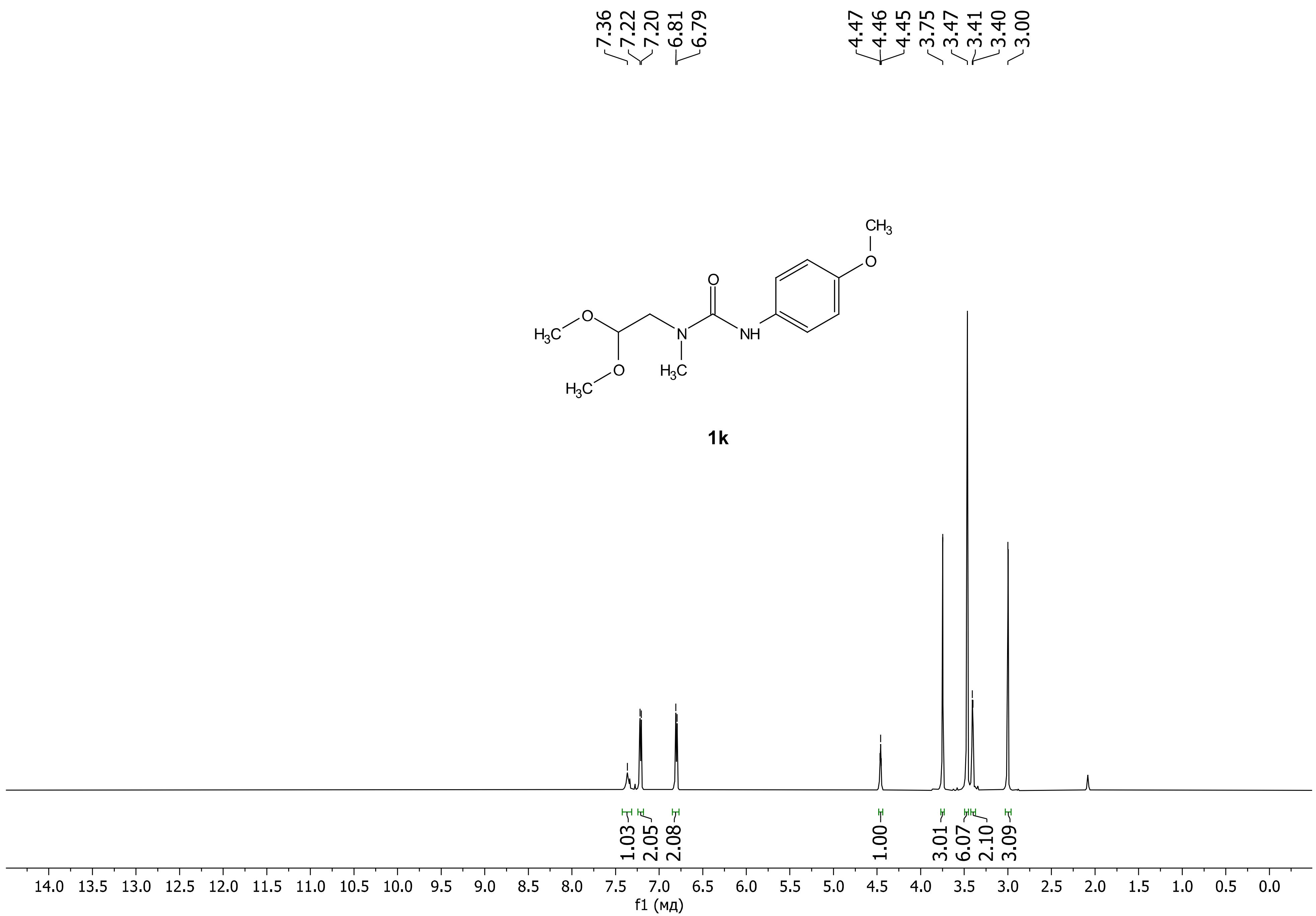

Figure S17. ^{1}H NMR spectrum ($CDCl_3$, 400MHz, 303K) of the compound **1k**

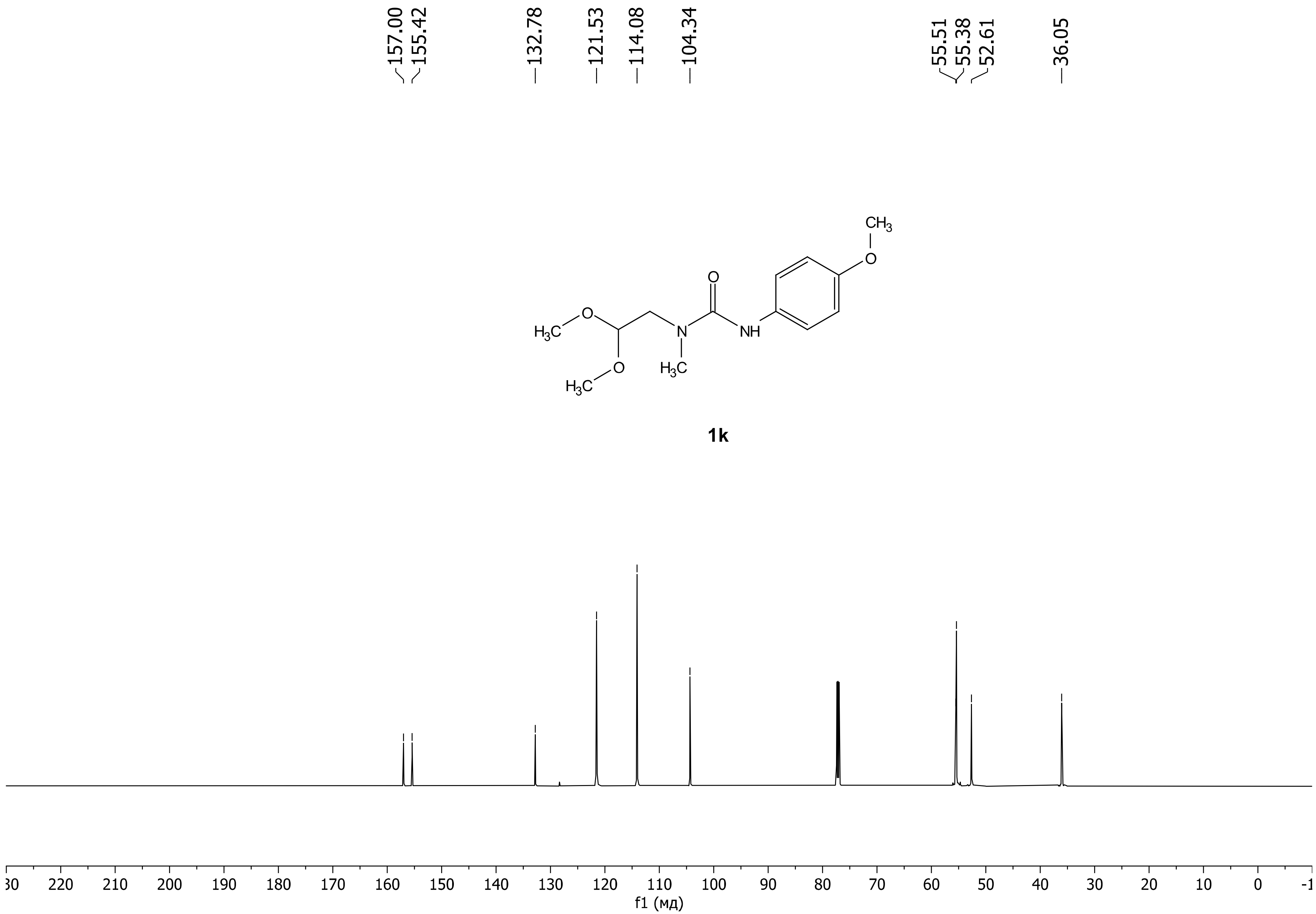

Figure S18. ^{13}C NMR spectrum ($CDCl_3$, 151MHz, 303K) of the compound **1k**

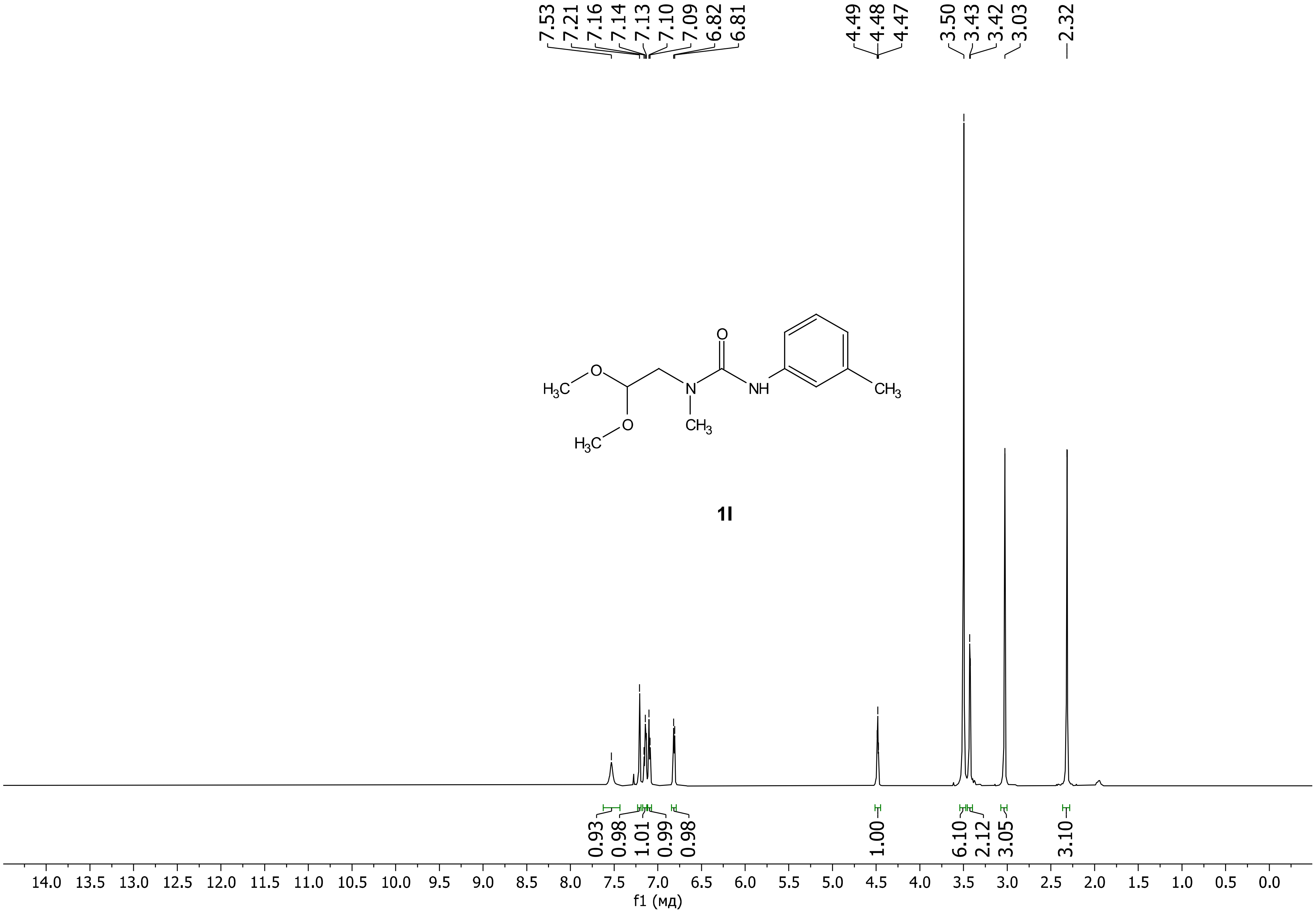

1l

Figure S19. ^{1}H NMR spectrum ($CDCl_3$, 400MHz, 303K) of the compound **1l**

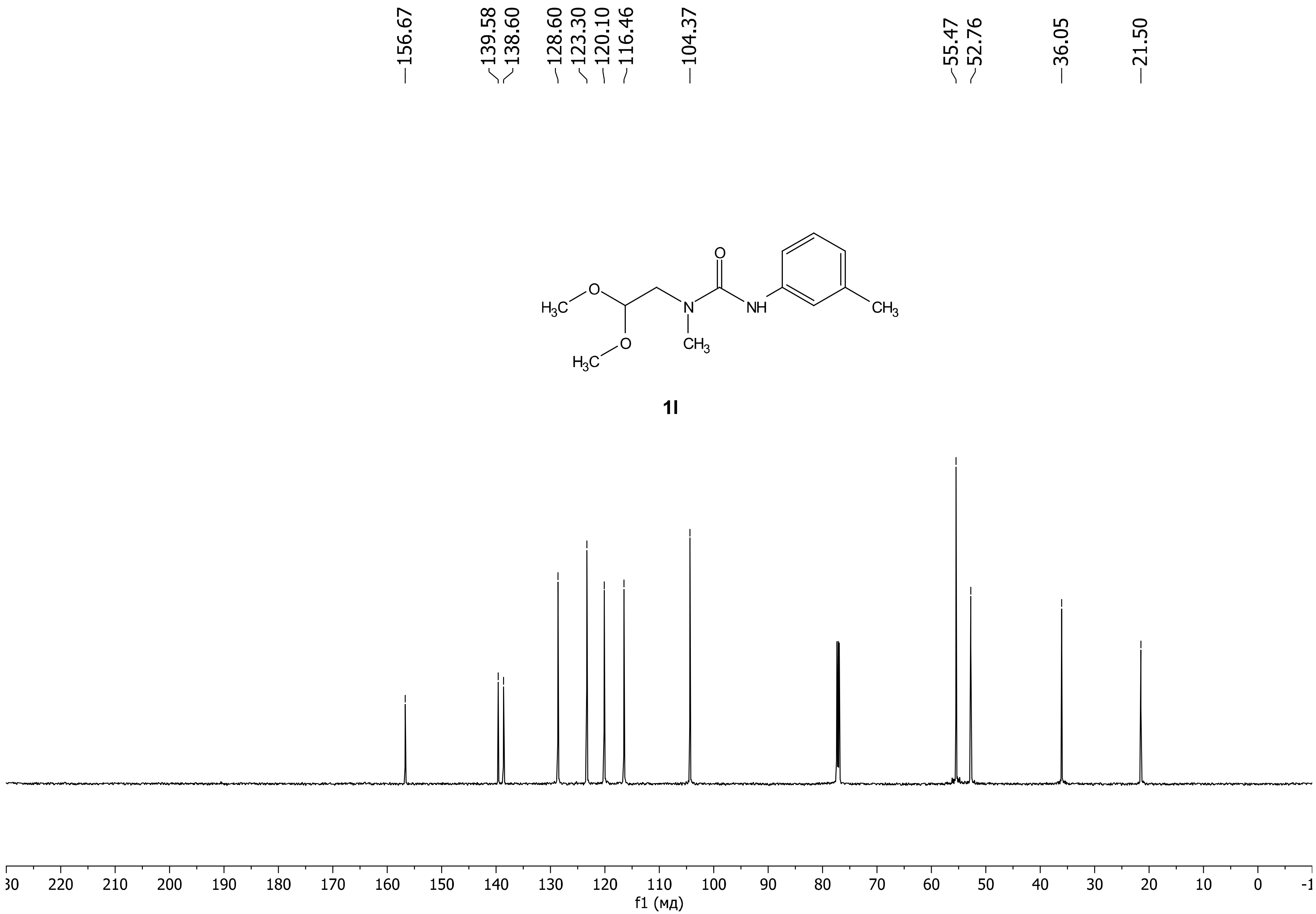

Figure S20. ^{13}C NMR spectrum ($CDCl_3$, 151MHz, 303K) of the compound **1l**

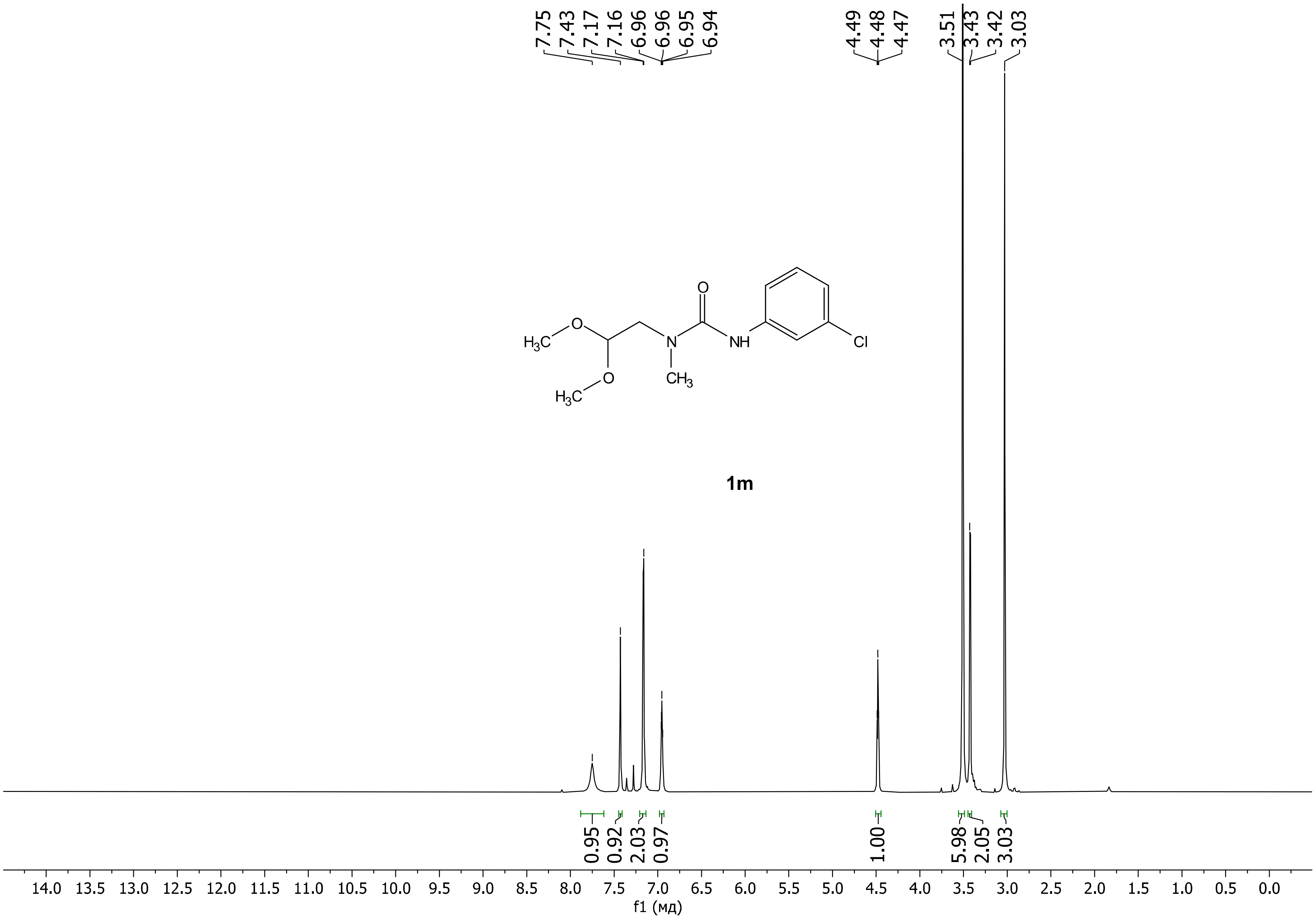

Figure S21. ^{1}H NMR spectrum ($CDCl_3$, 400MHz, 303K) of the compound **1m**

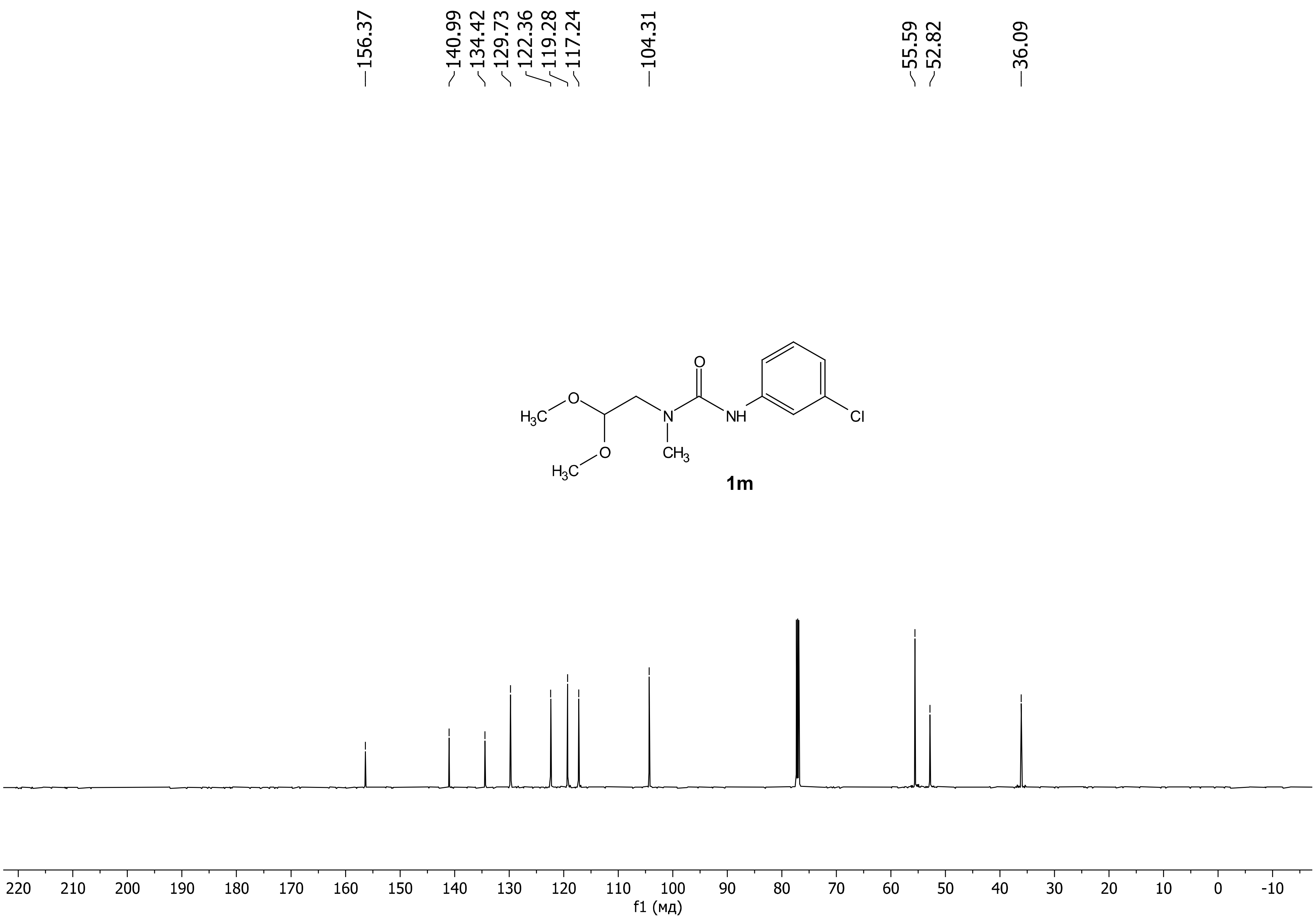

Figure S22. ^{13}C NMR spectrum ($CDCl_3$, 151MHz, 303K) of the compound **1m**

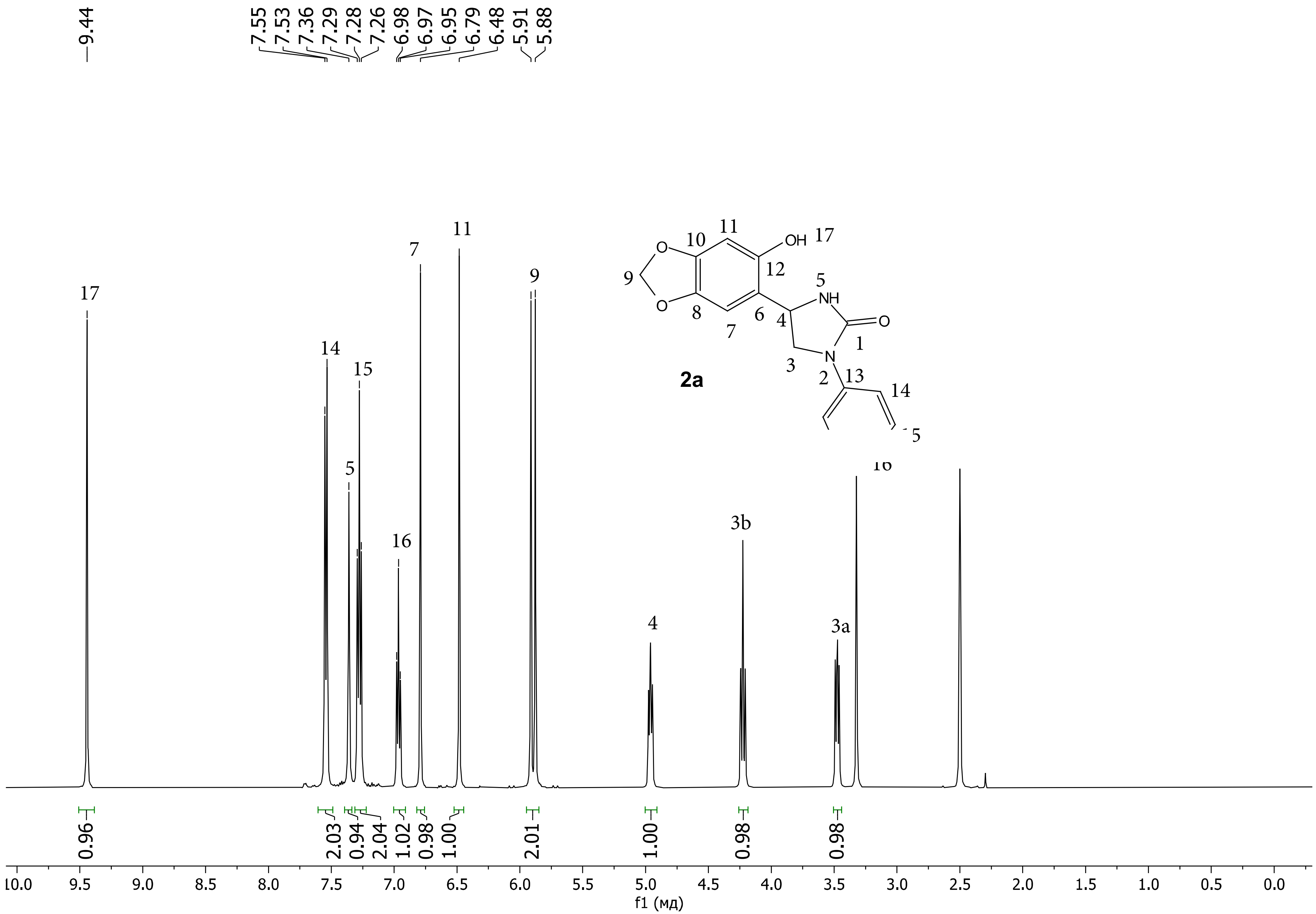

Figure S23. ^{1}H NMR spectrum (DMSO-d_6, 400MHz, 303K) of the compound **2a**

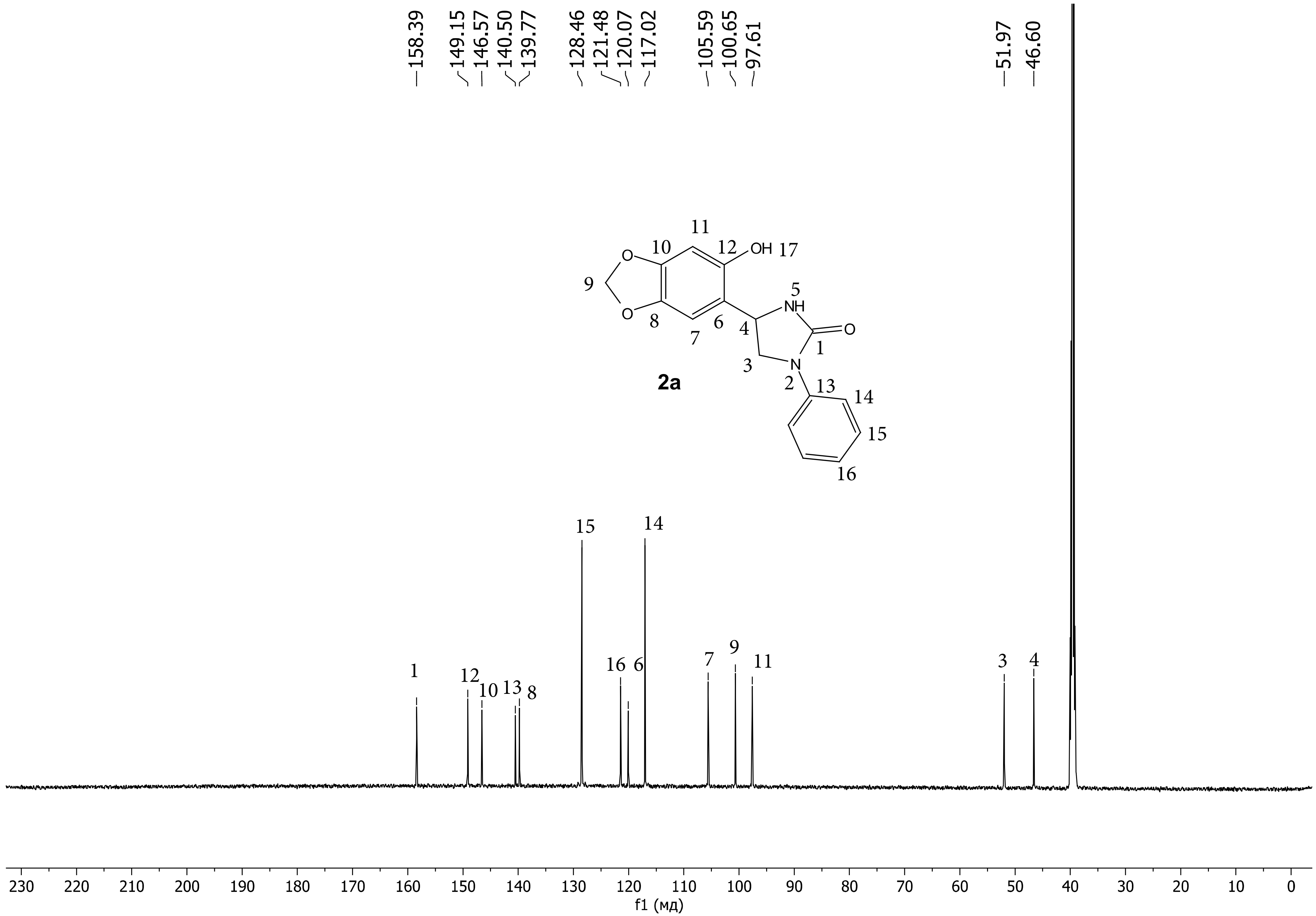

Figure S24. ^{13}C NMR spectrum (DMSO-d_6, 151MHz, 303K) of the compound **2a**

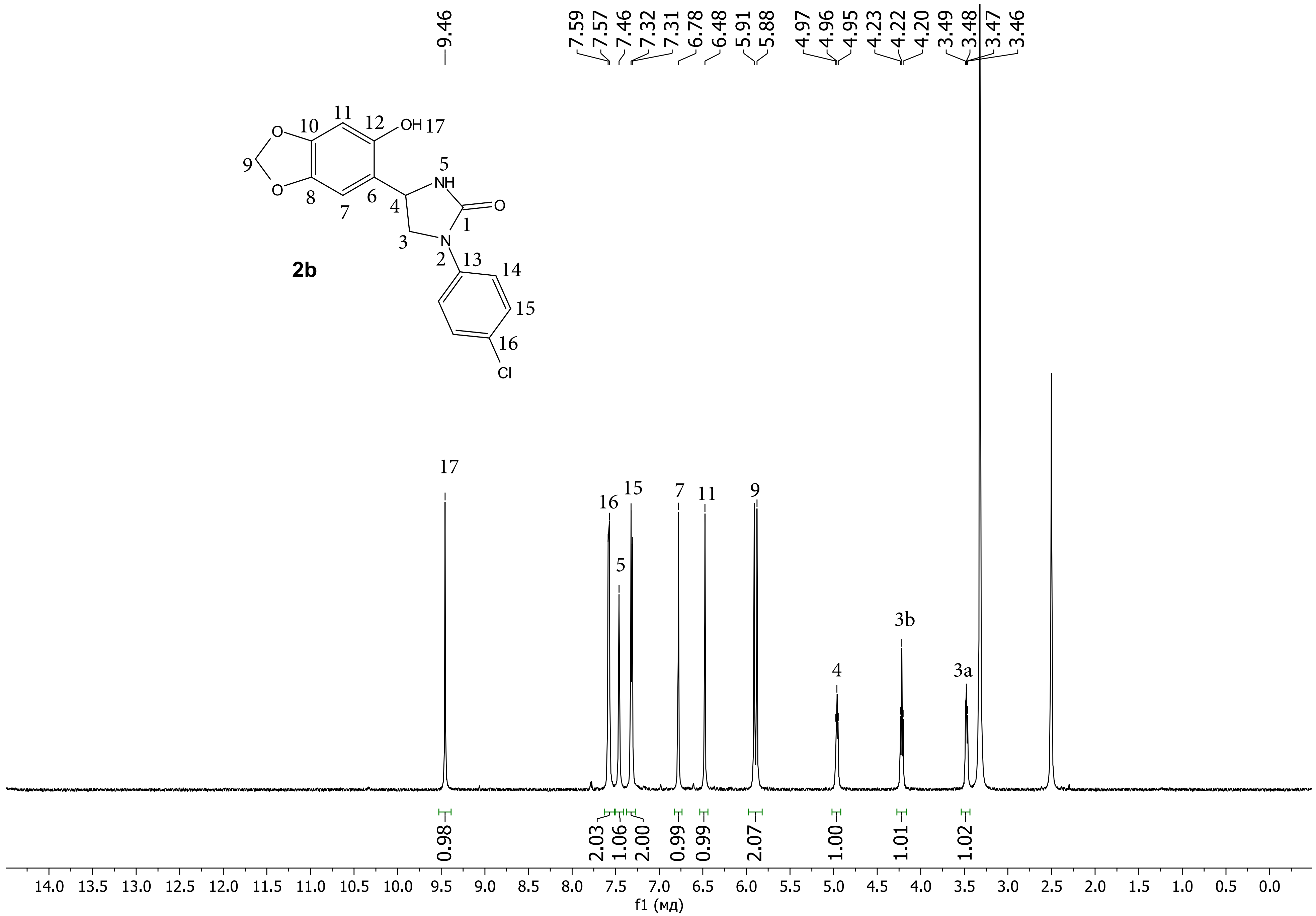

Figure S25. ^{1}H NMR spectrum (DMSO-d_6, 400MHz, 303K) of the compound **2b**

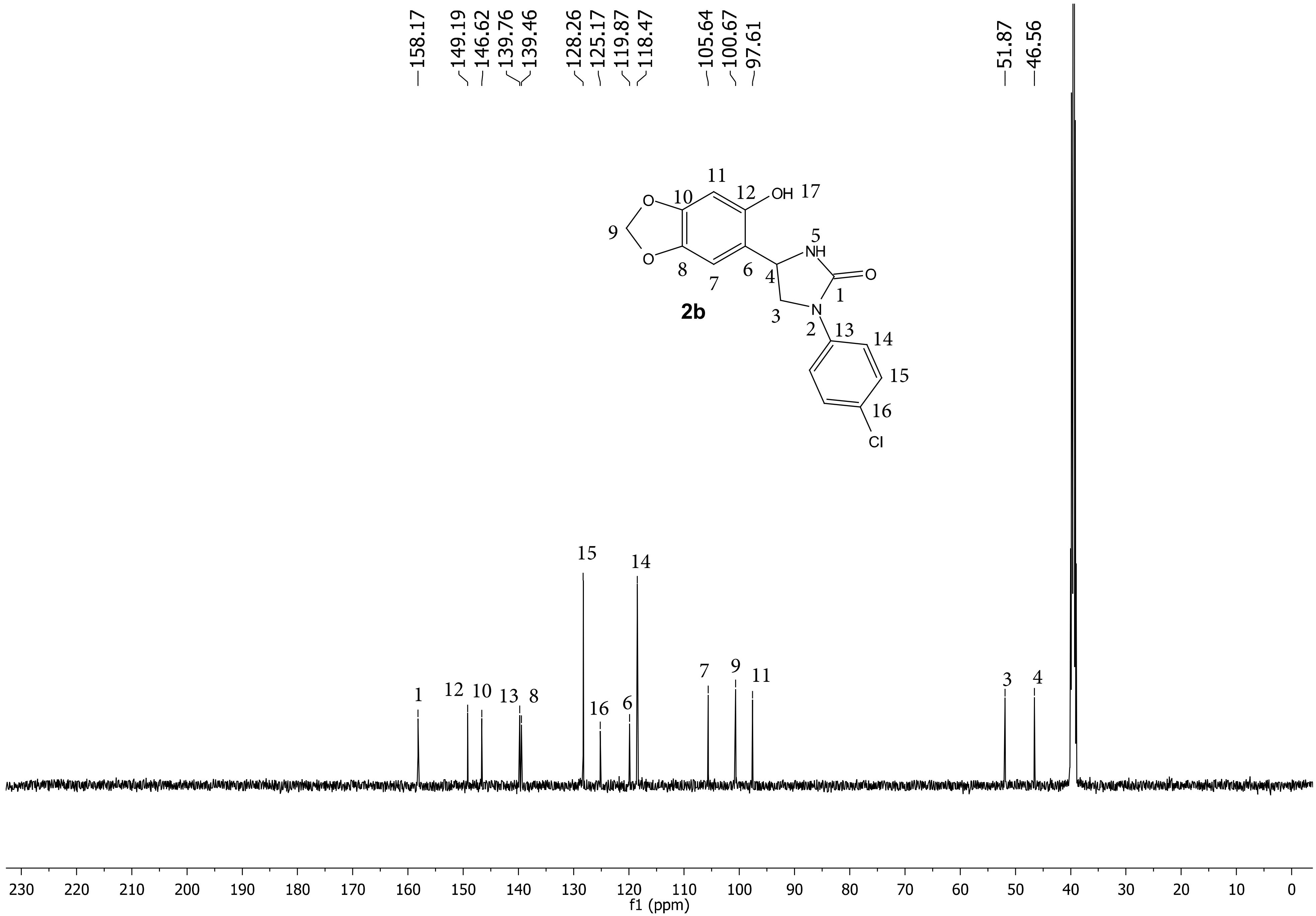

Figure S26. ^{13}C NMR spectrum (DMSO-d_6, 151MHz, 303K) of the compound **2b**

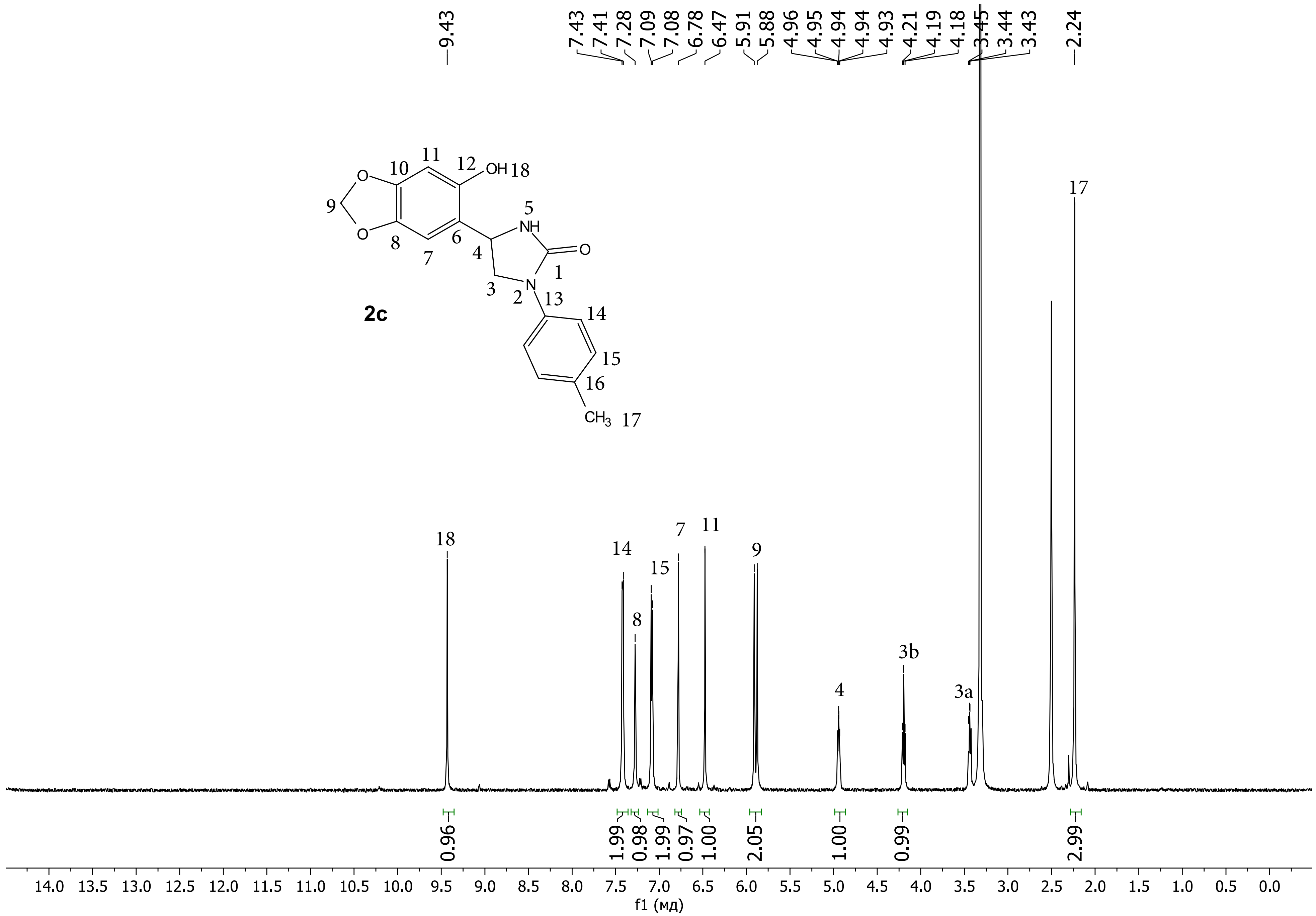

Figure S27. ^{1}H NMR spectrum (DMSO-d_6, 400MHz, 303K) of the compound **2c**

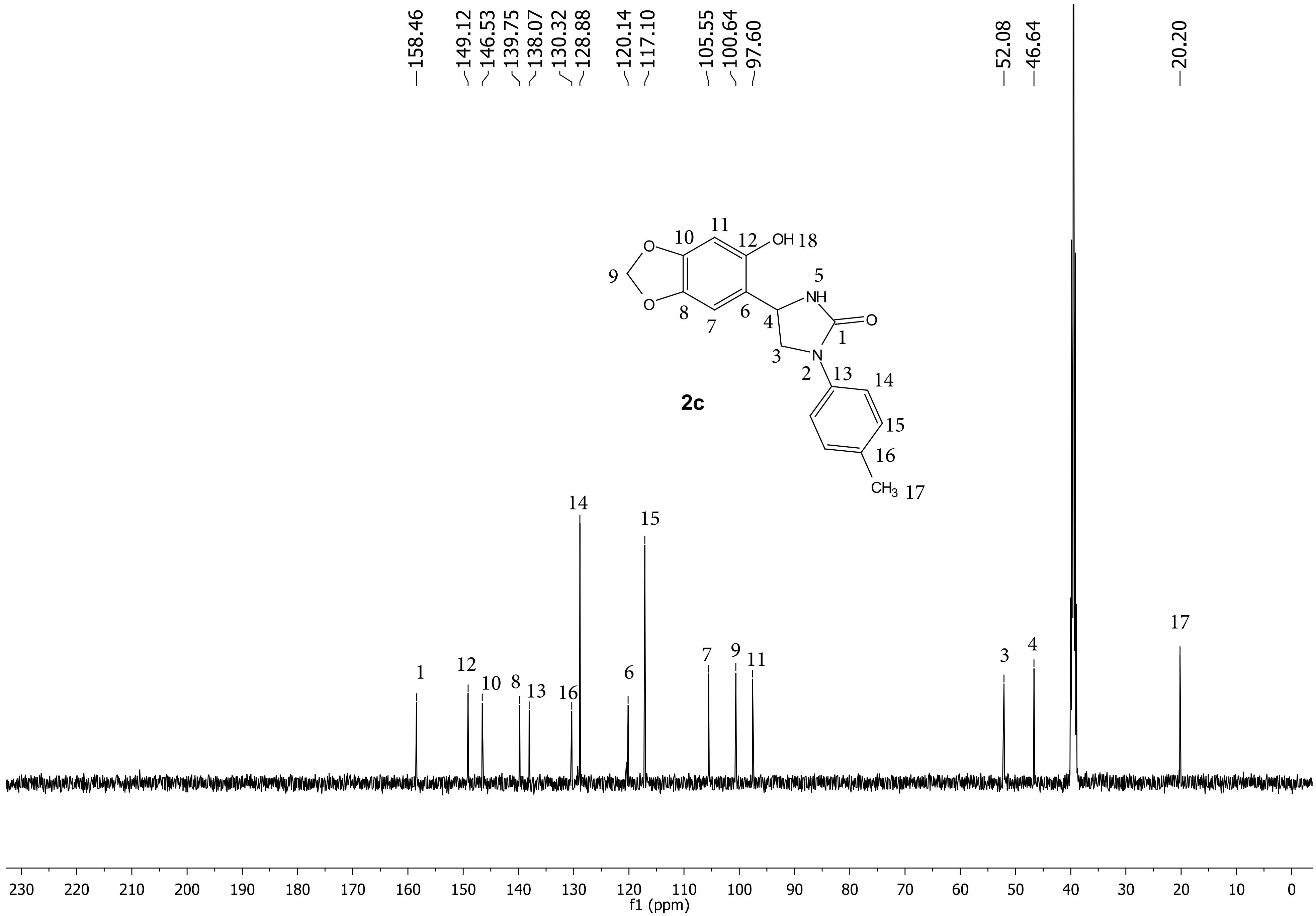

Figure S28. ^{13}C NMR spectrum (DMSO-d_6, 151MHz, 303K) of the compound **2c**

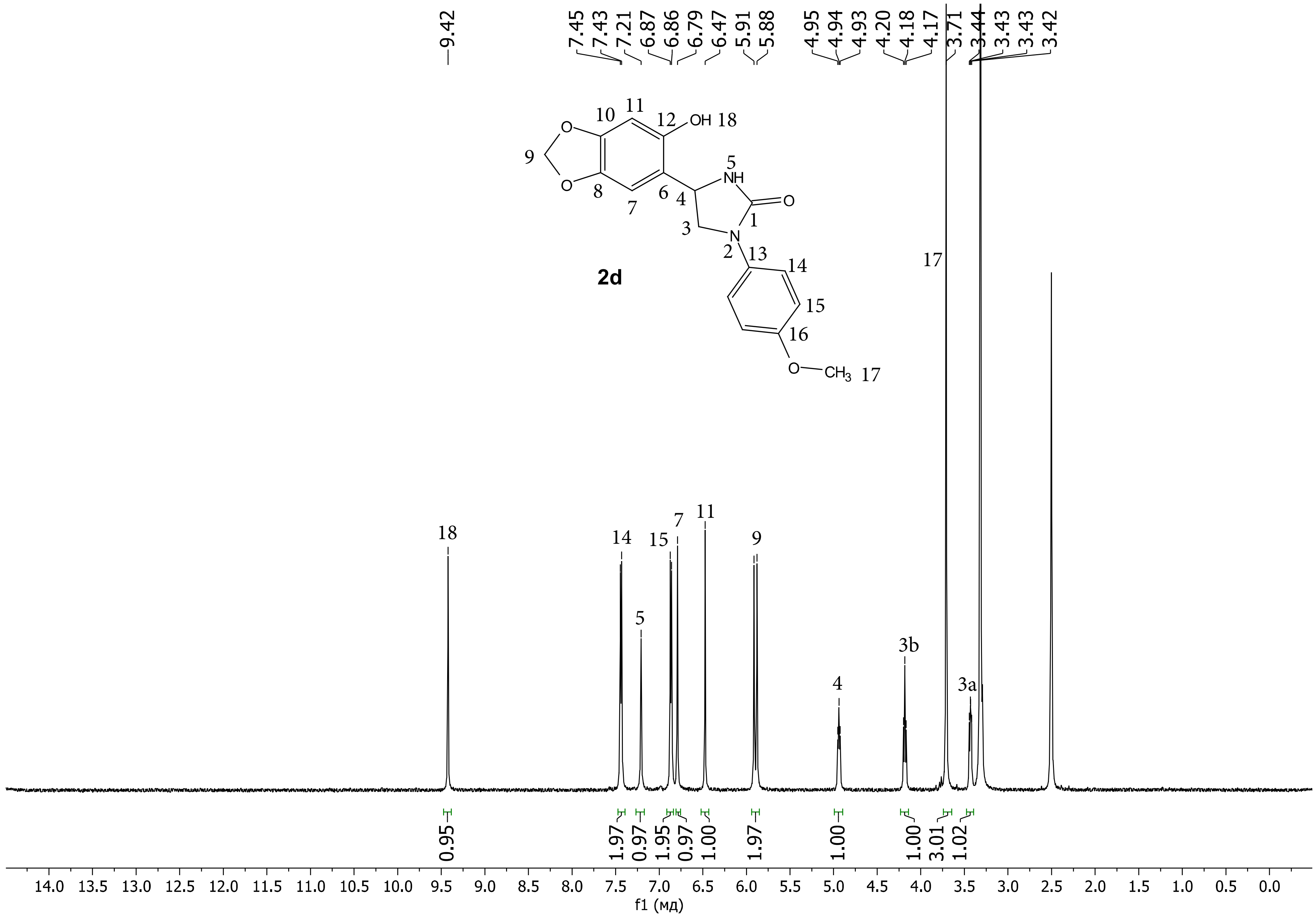

Figure S29. ^{1}H NMR spectrum (DMSO-d_6, 400MHz, 303K) of the compound **2d**

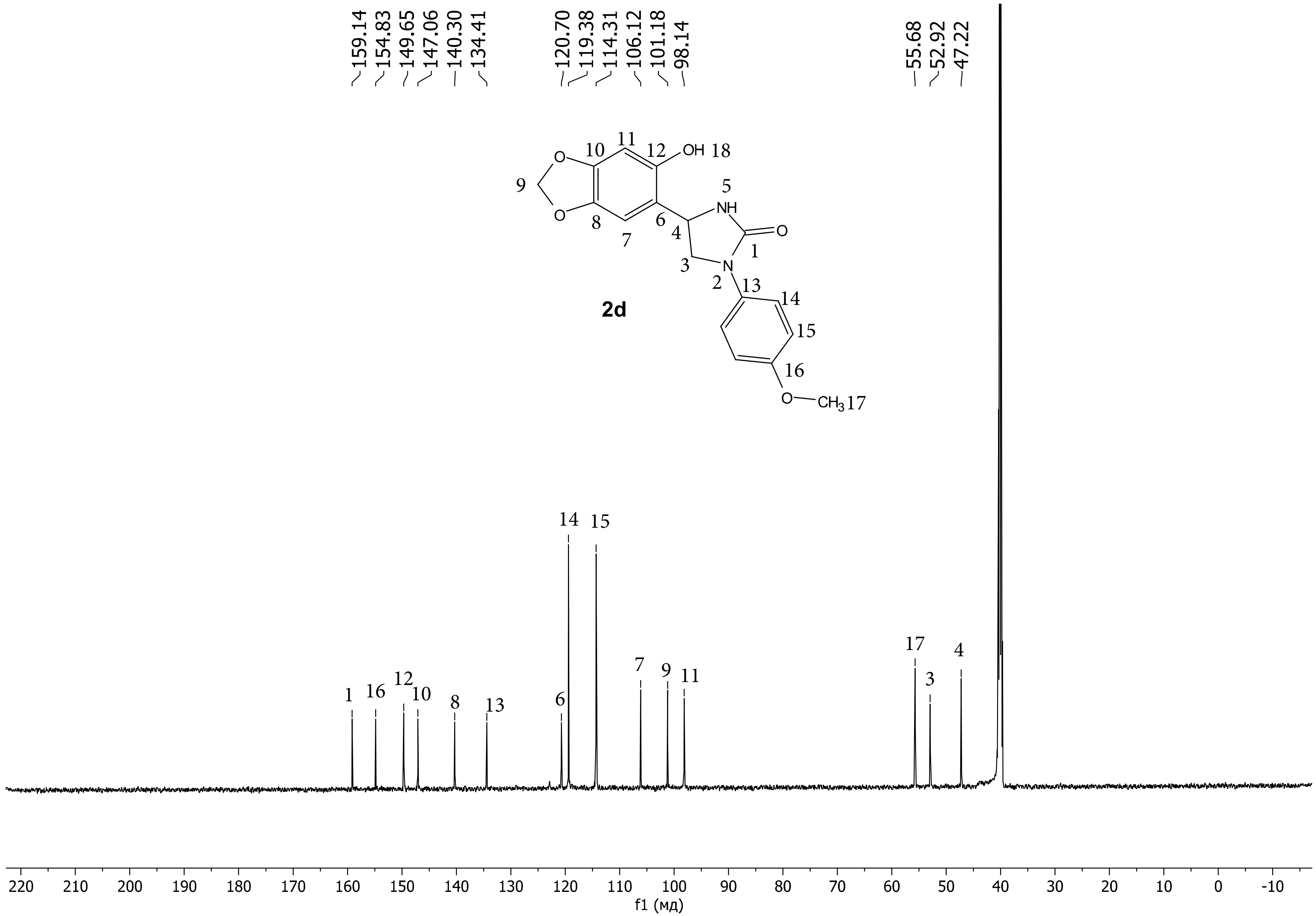

Figure S30. ^{13}C NMR spectrum (DMSO-d_6, 151MHz, 303K) of the compound **2d**

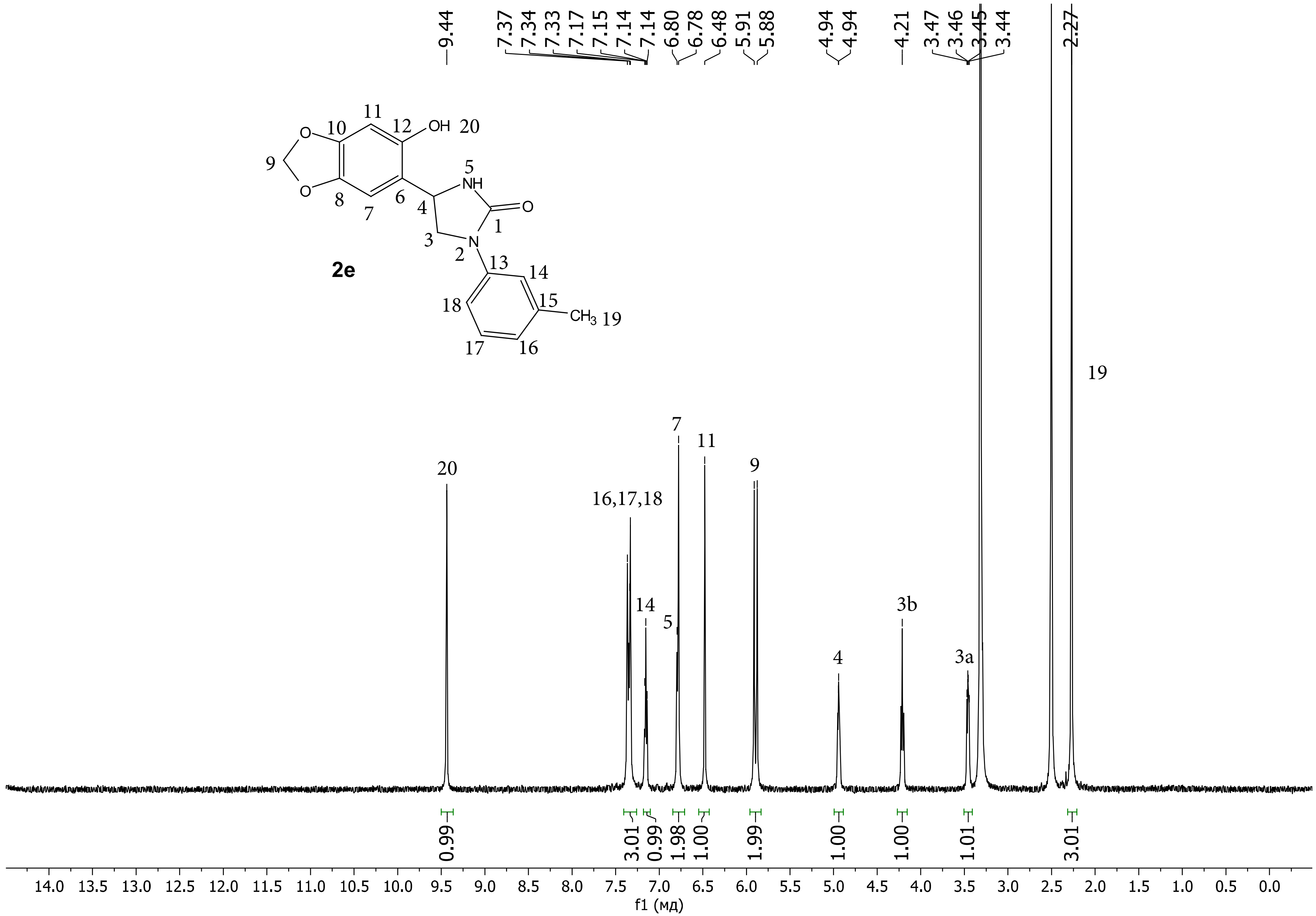

Figure S31. ^{1}H NMR spectrum (DMSO-d_6, 400MHz, 303K) of the compound **2e**

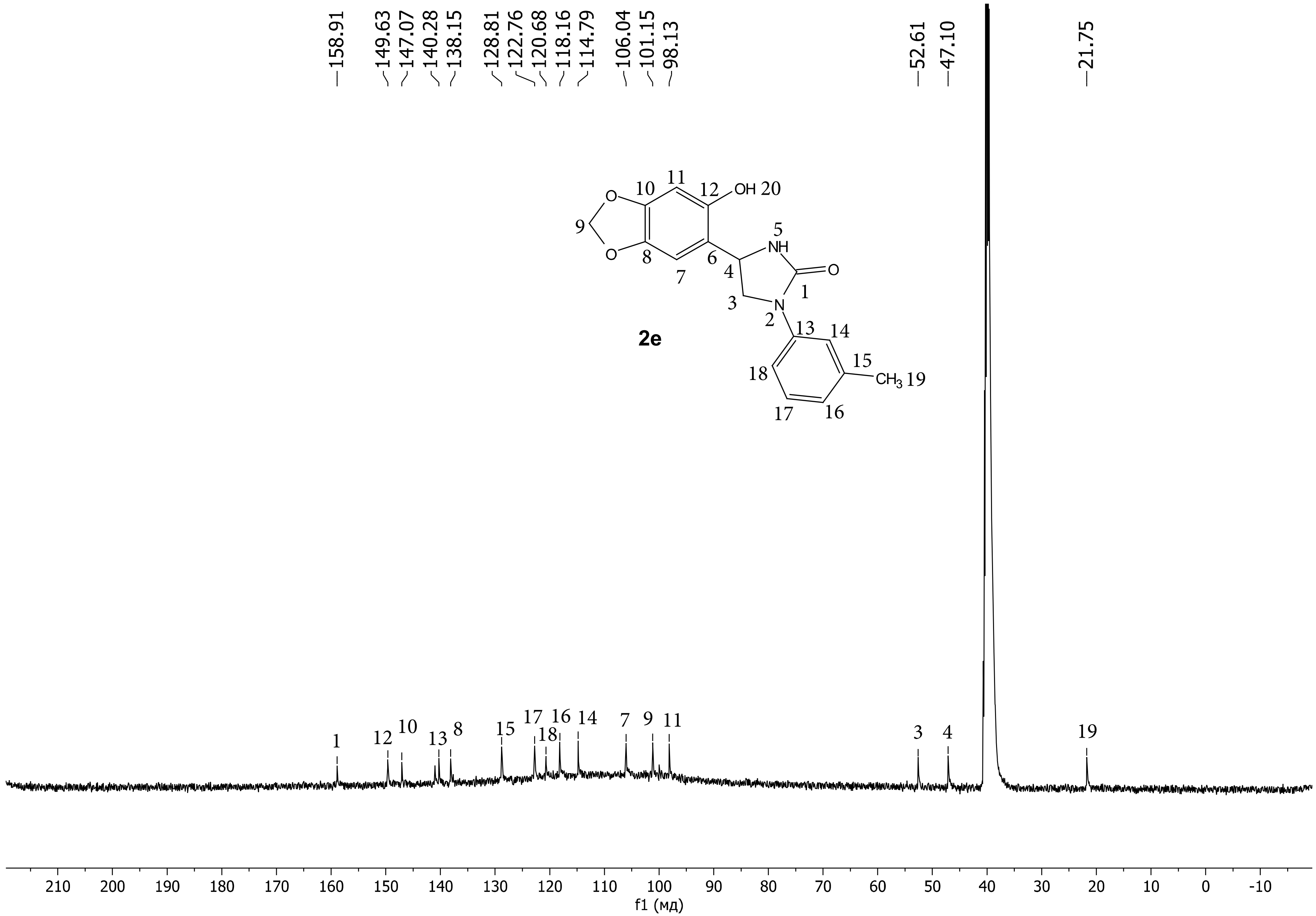

Figure S32. ^{13}C NMR spectrum (DMSO-d_6, 151MHz, 303K) of the compound **2e**

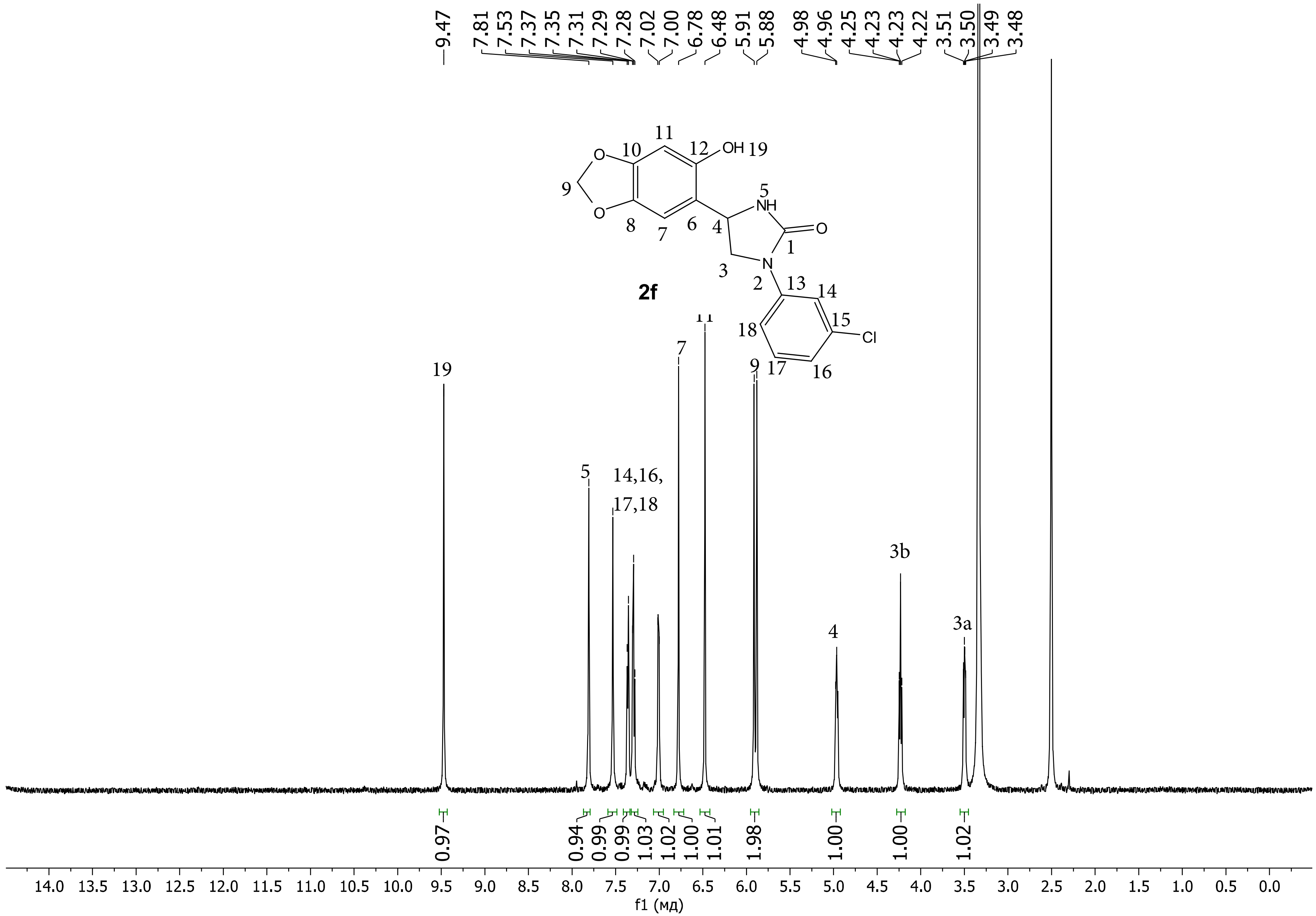

Figure S33. ^{1}H NMR spectrum (DMSO-d_6, 400MHz, 303K) of the compound **2f**

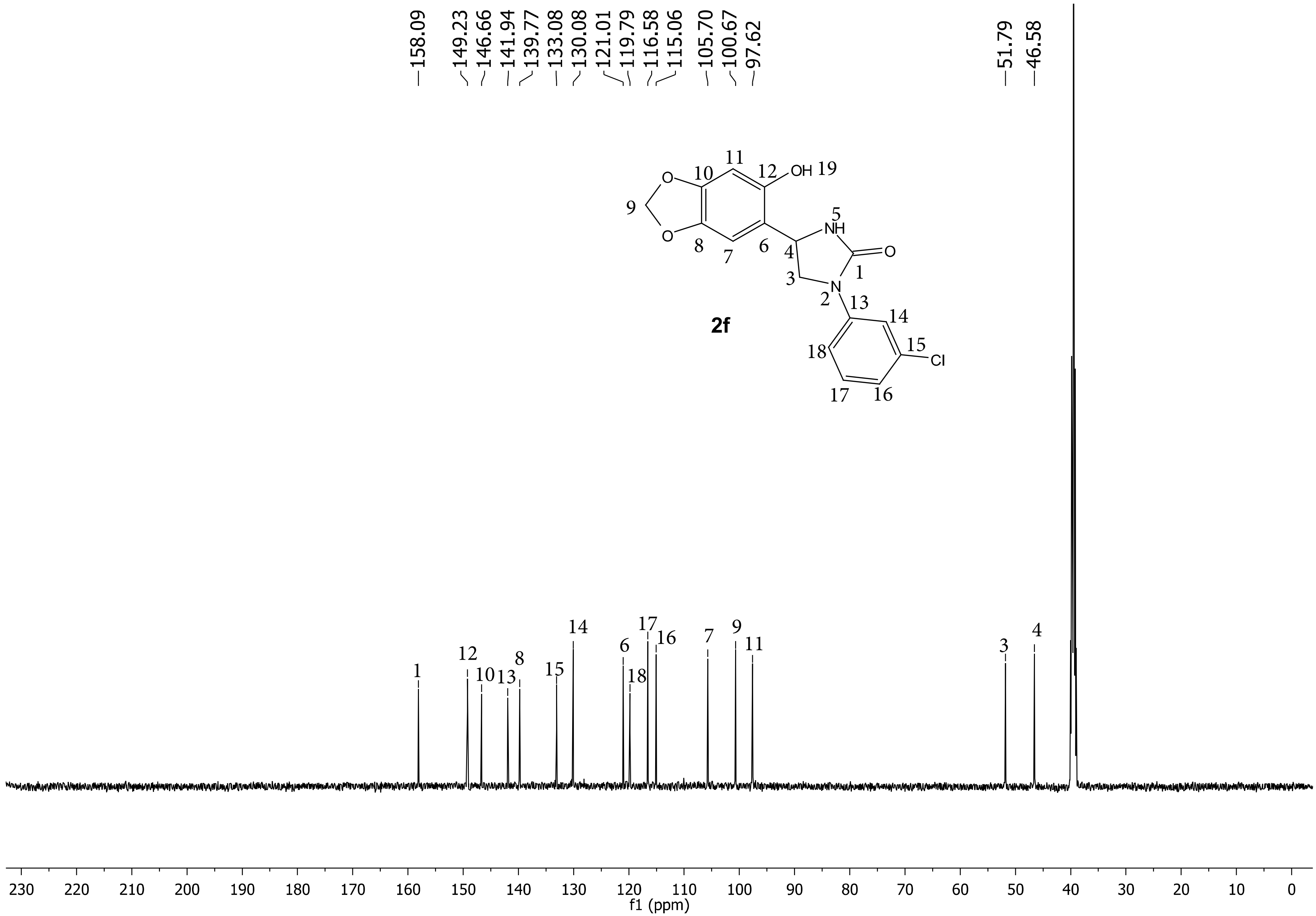

Figure S34. ^{13}C NMR spectrum (DMSO-d_6, 151MHz, 303K) of the compound **2f**

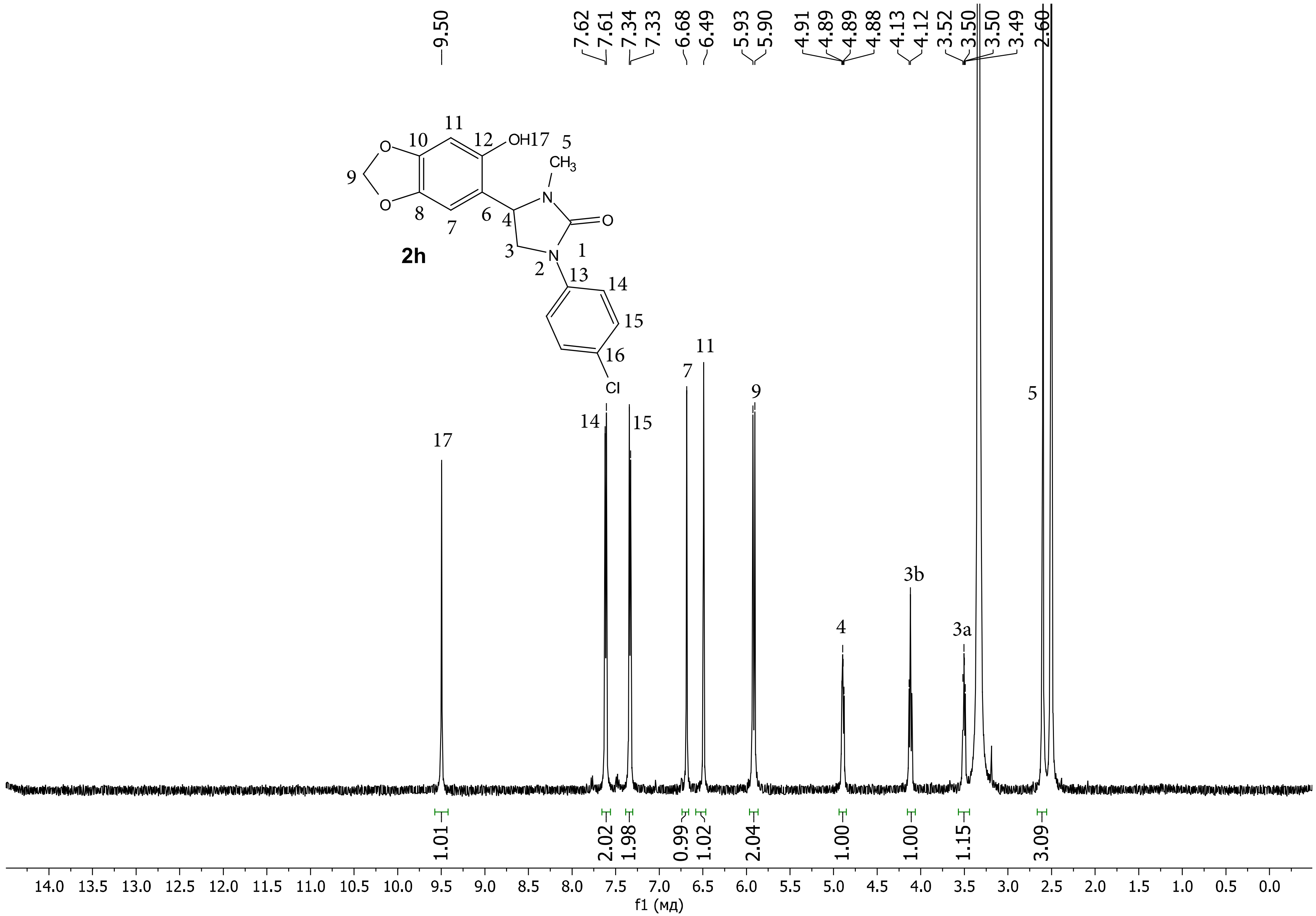

Figure S35. ^{1}H NMR spectrum (DMSO-d_6, 400MHz, 303K) of the compound **2h**

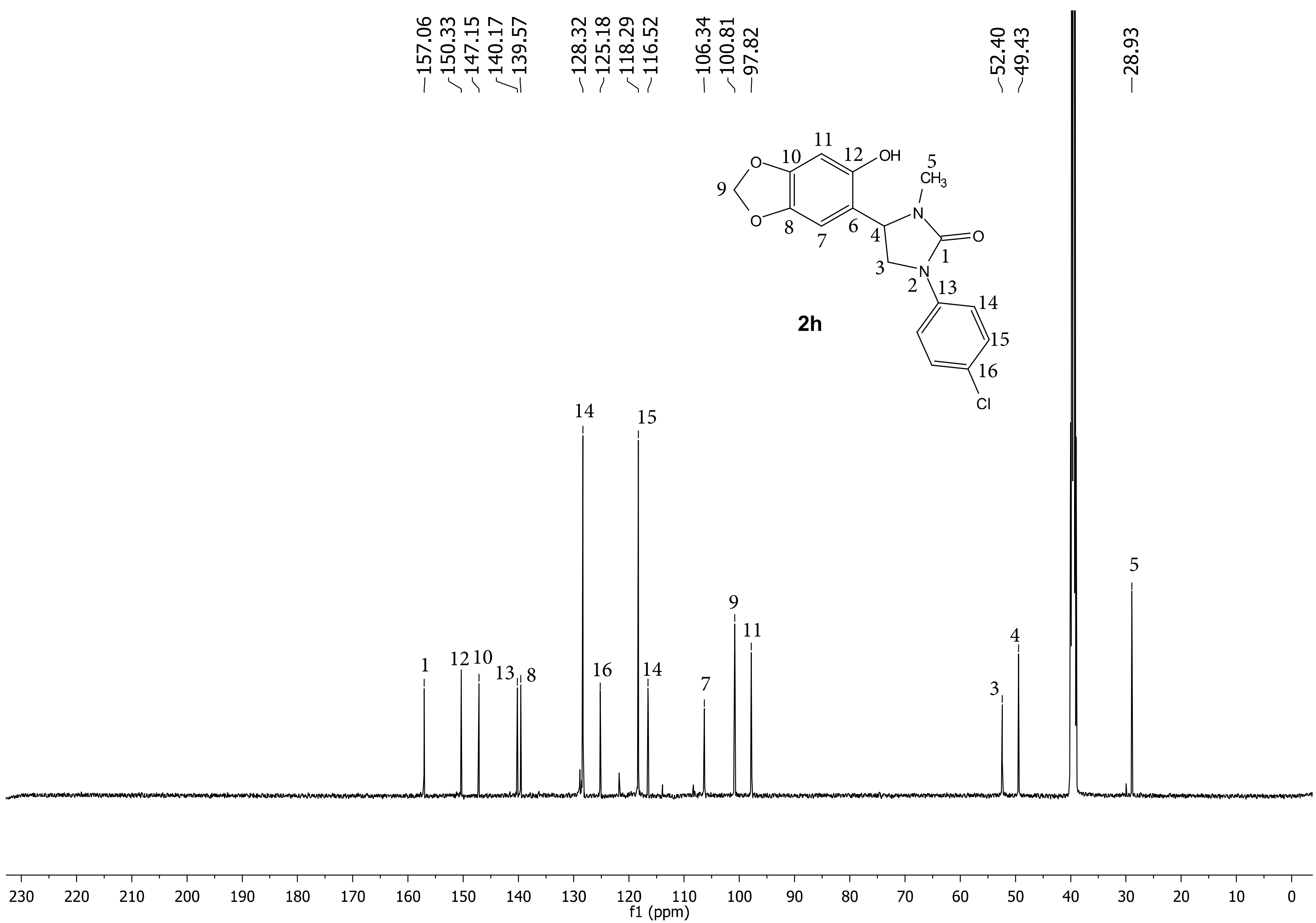

Figure S36. ^{13}C NMR spectrum (DMSO-d_6, 151MHz, 303K) of the compound **2h**

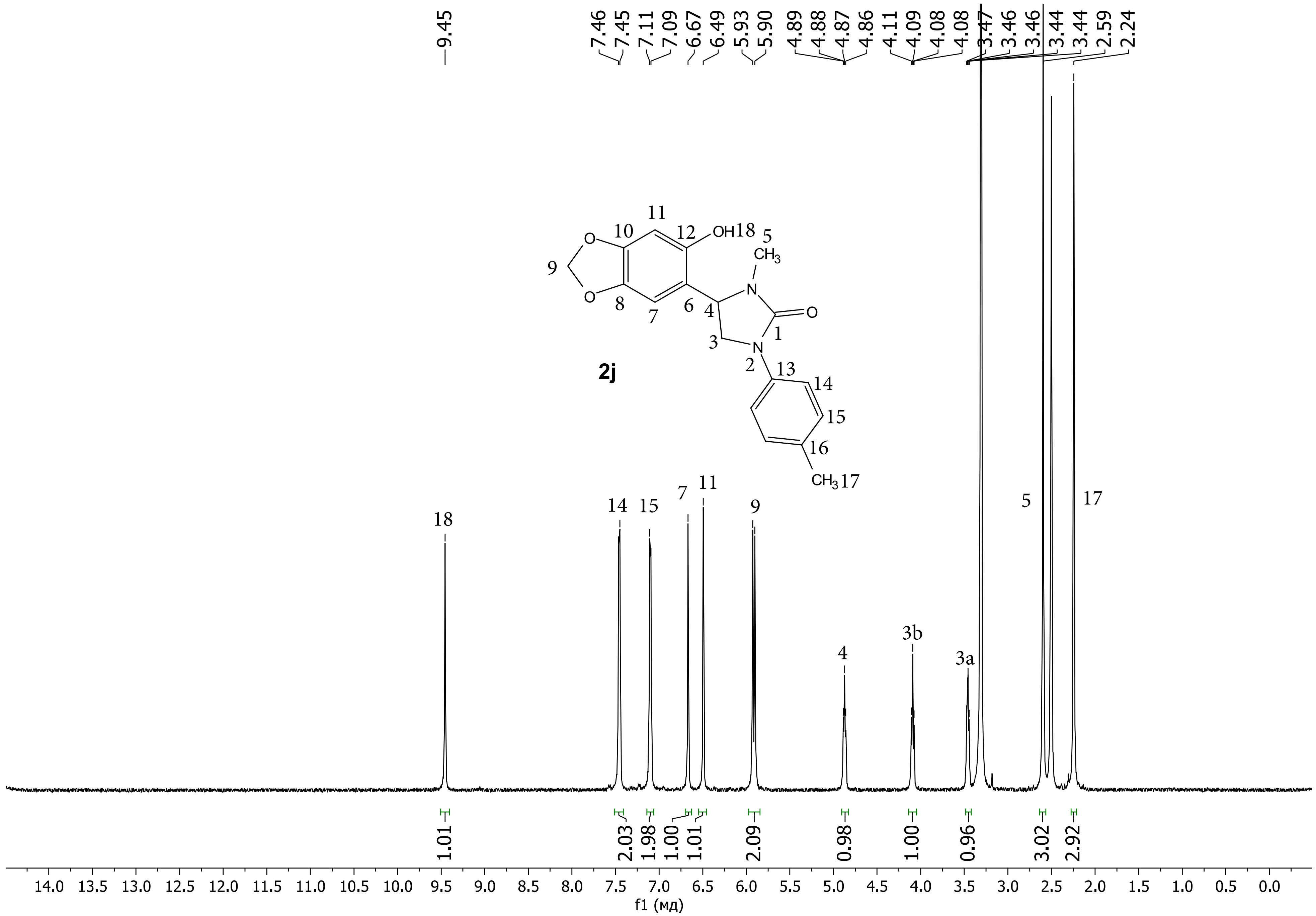

Figure S37. ^{1}H NMR spectrum (DMSO-d_6, 400MHz, 303K) of the compound **2j**

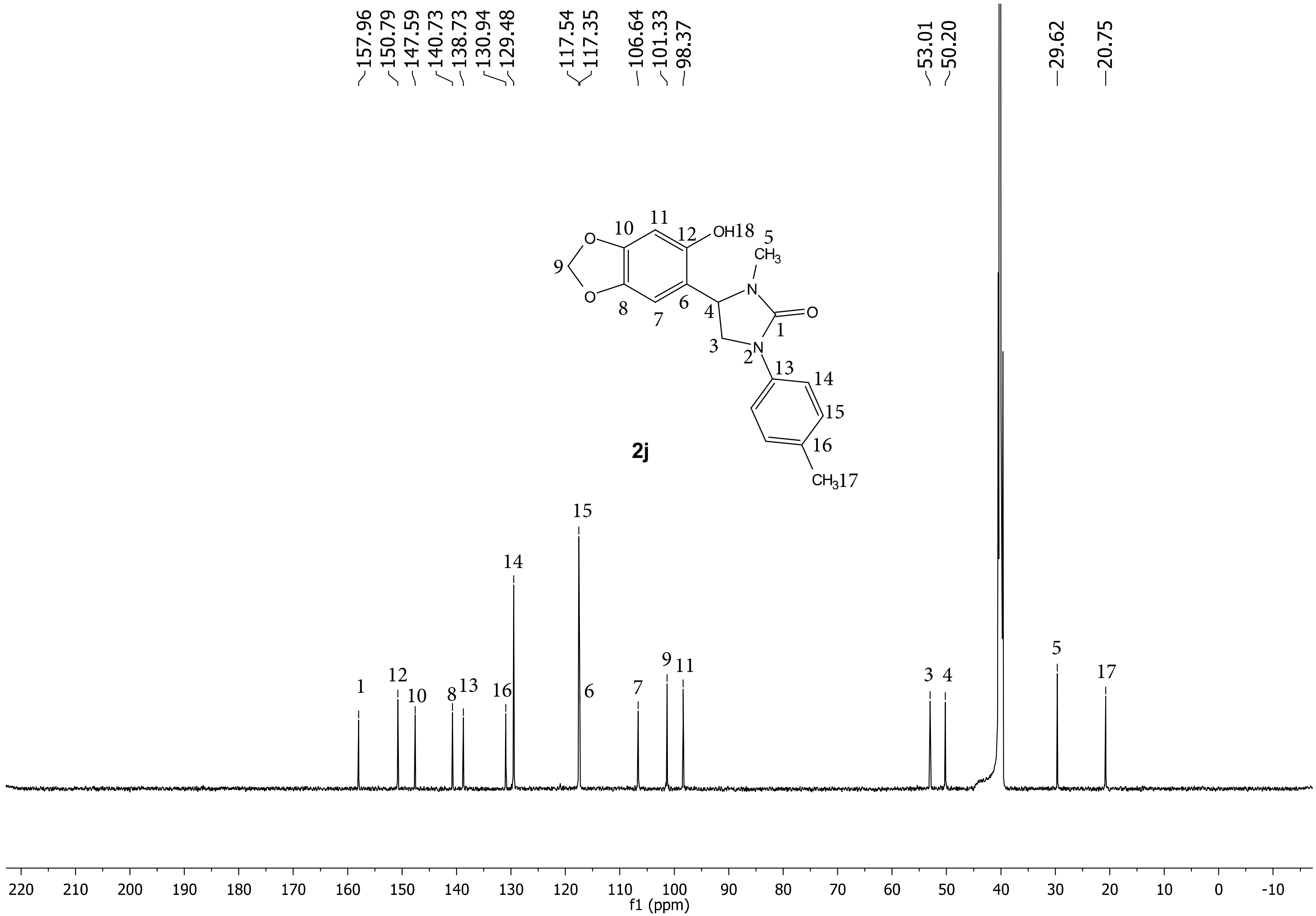

Figure S38. ^{13}C NMR spectrum (DMSO-d_6, 151MHz, 303K) of the compound **2j**

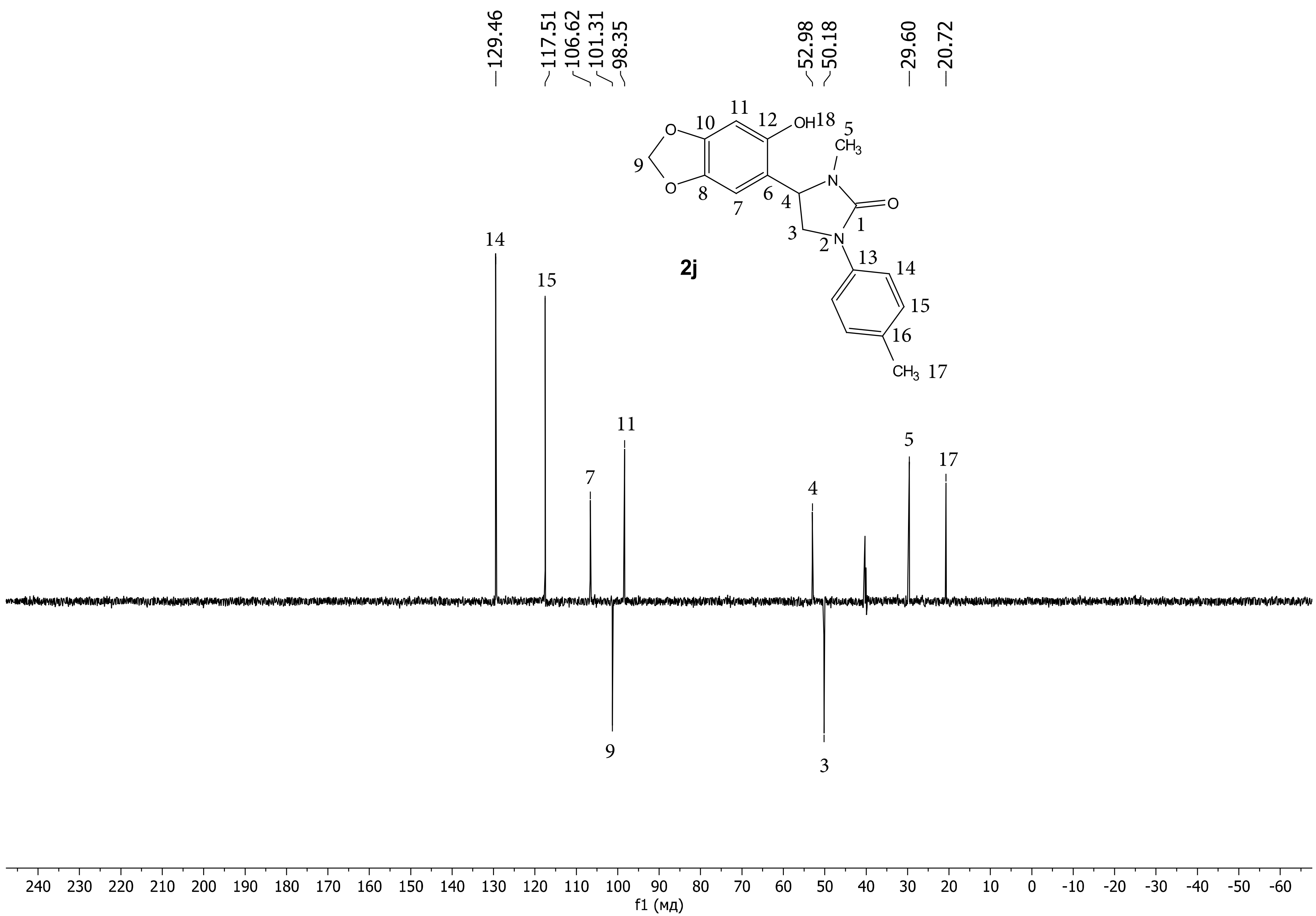

Figure S39. ^{13}C DEPT NMR spectrum (DMSO-d_6, 151MHz, 135° pulse, 303K) of the compound **2j**

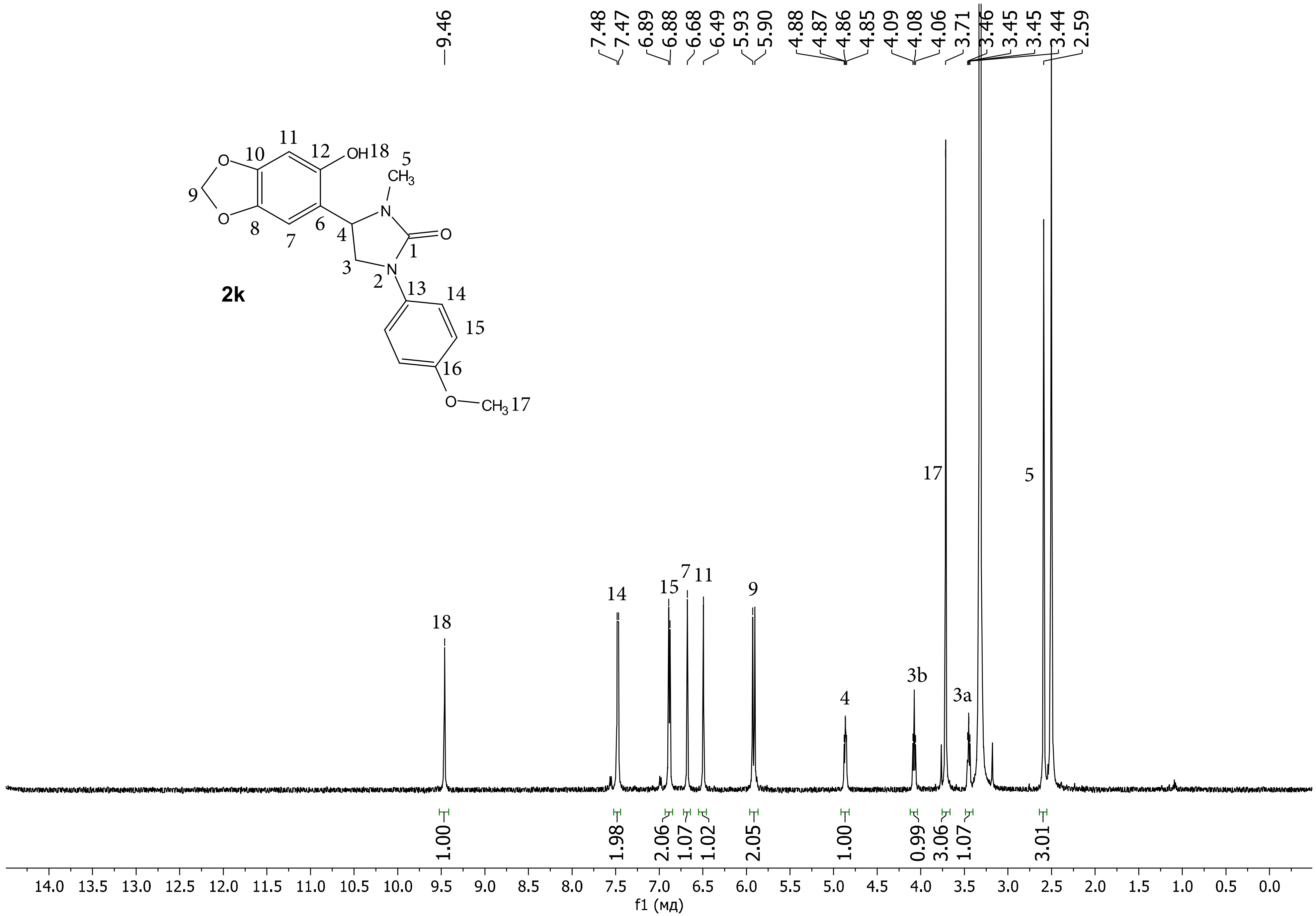

Figure S40. ^{1}H NMR spectrum (DMSO-d_6, 400MHz, 303K) of the compound **2k**

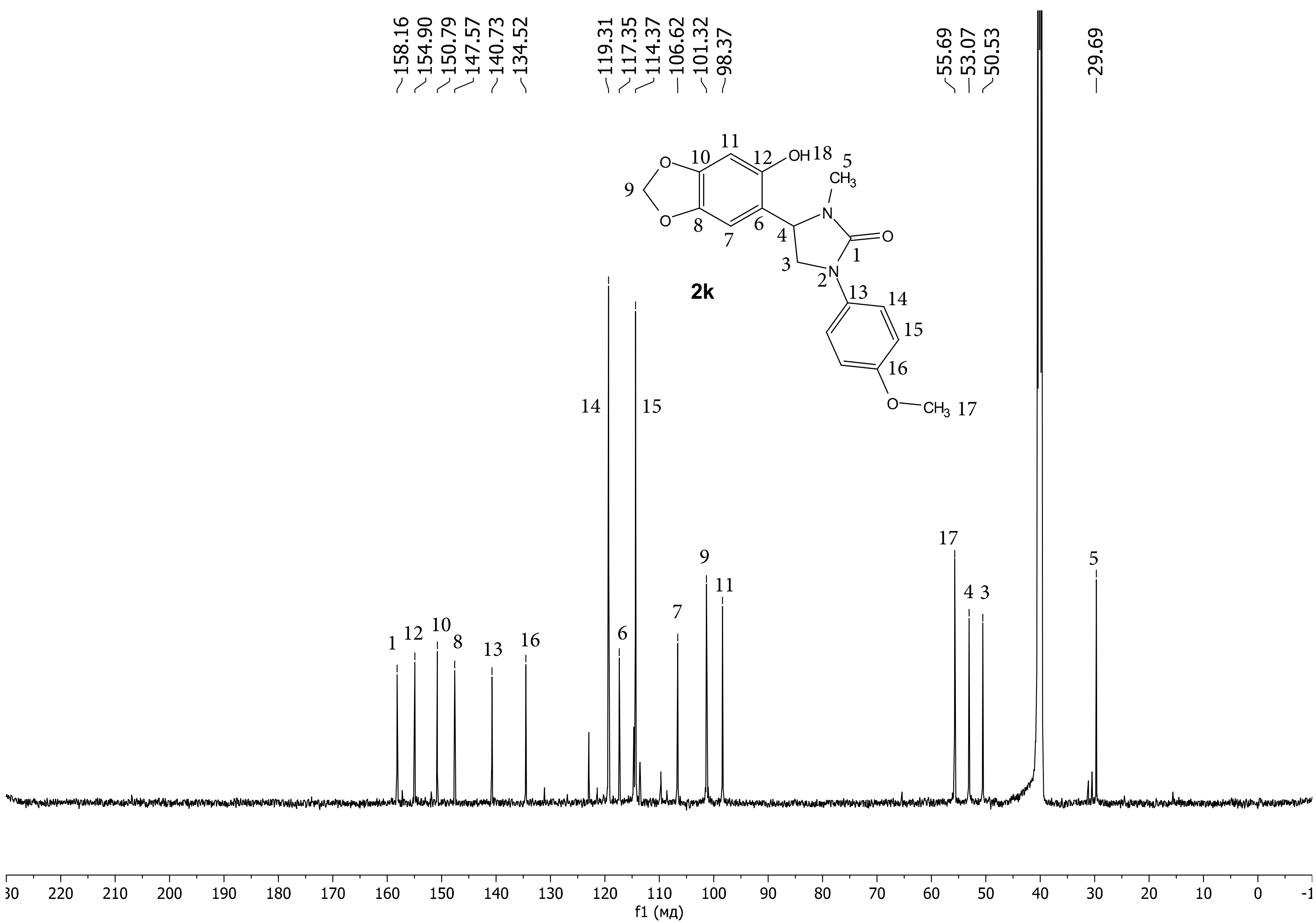

Figure S41. ^{13}C NMR spectrum (DMSO-d_6, 151MHz, 303K) of the compound **2k**

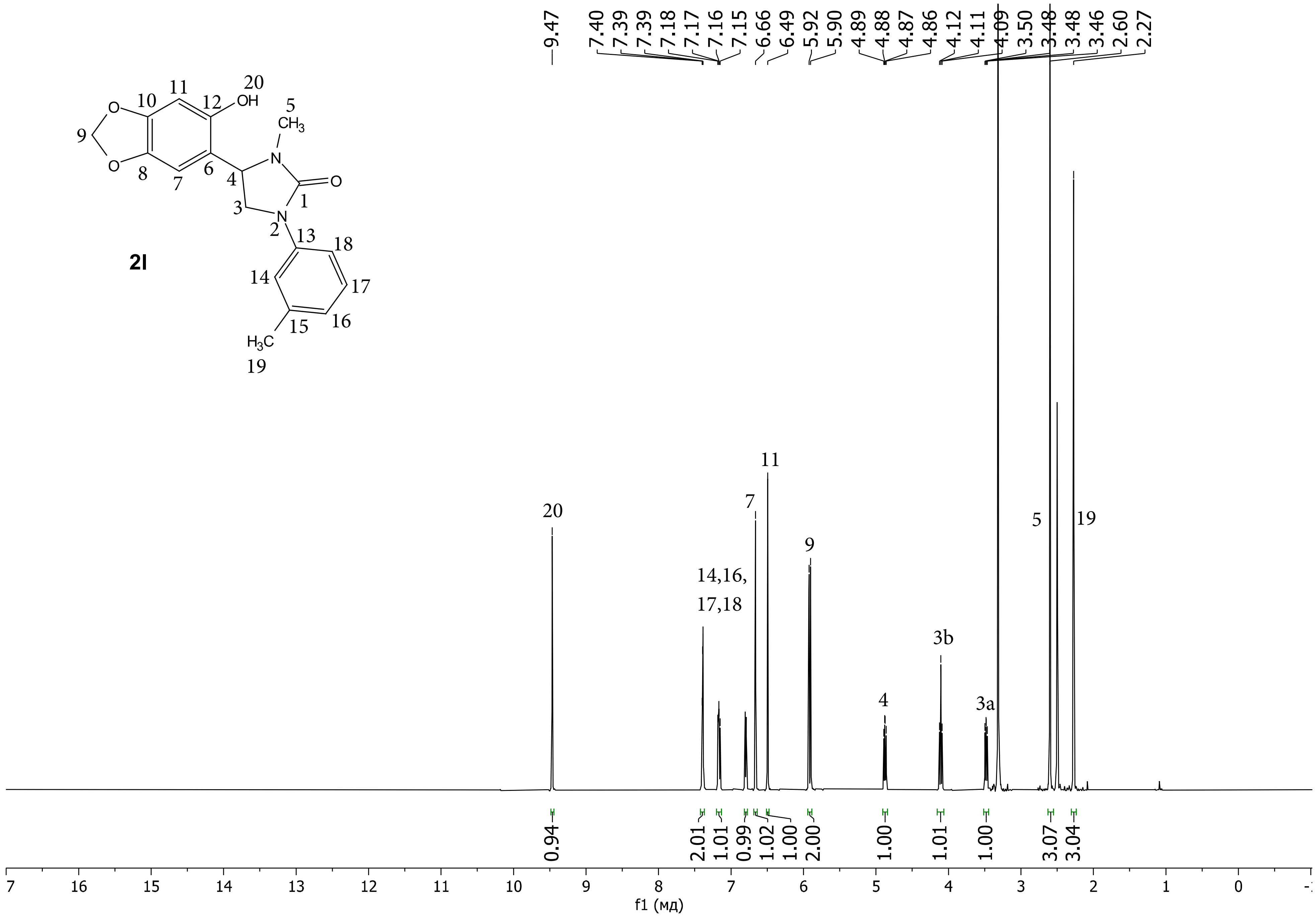

Figure S42. ^{1}H NMR spectrum (DMSO-d_6, 400MHz, 303K) of the compound **2l**

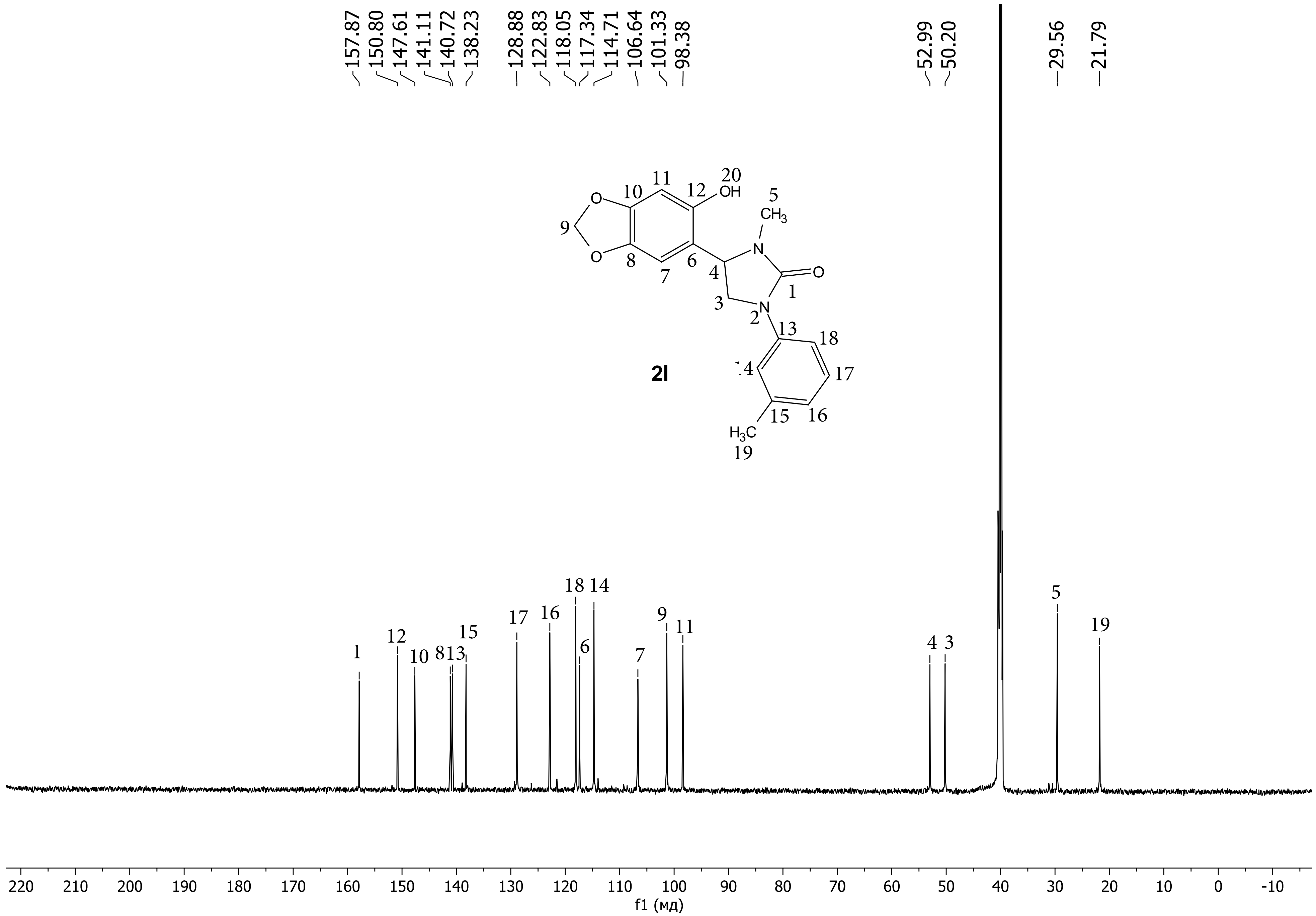

Figure S43. ^{13}C NMR spectrum (DMSO-d_6, 151MHz, 303K) of the compound **2l**

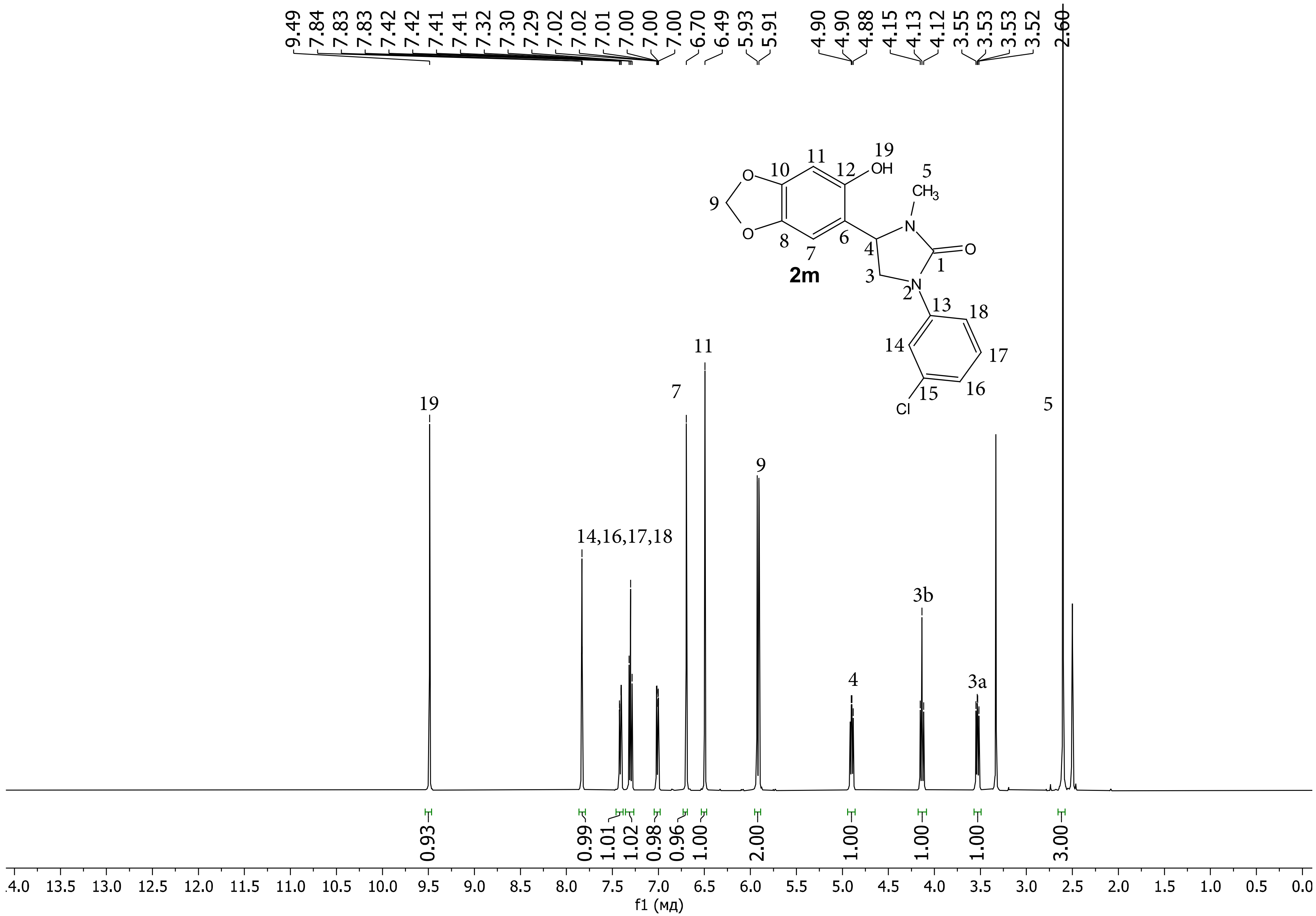

Figure S44. ^{1}H NMR spectrum (DMSO-d_6, 400MHz, 303K) of the compound **2m**

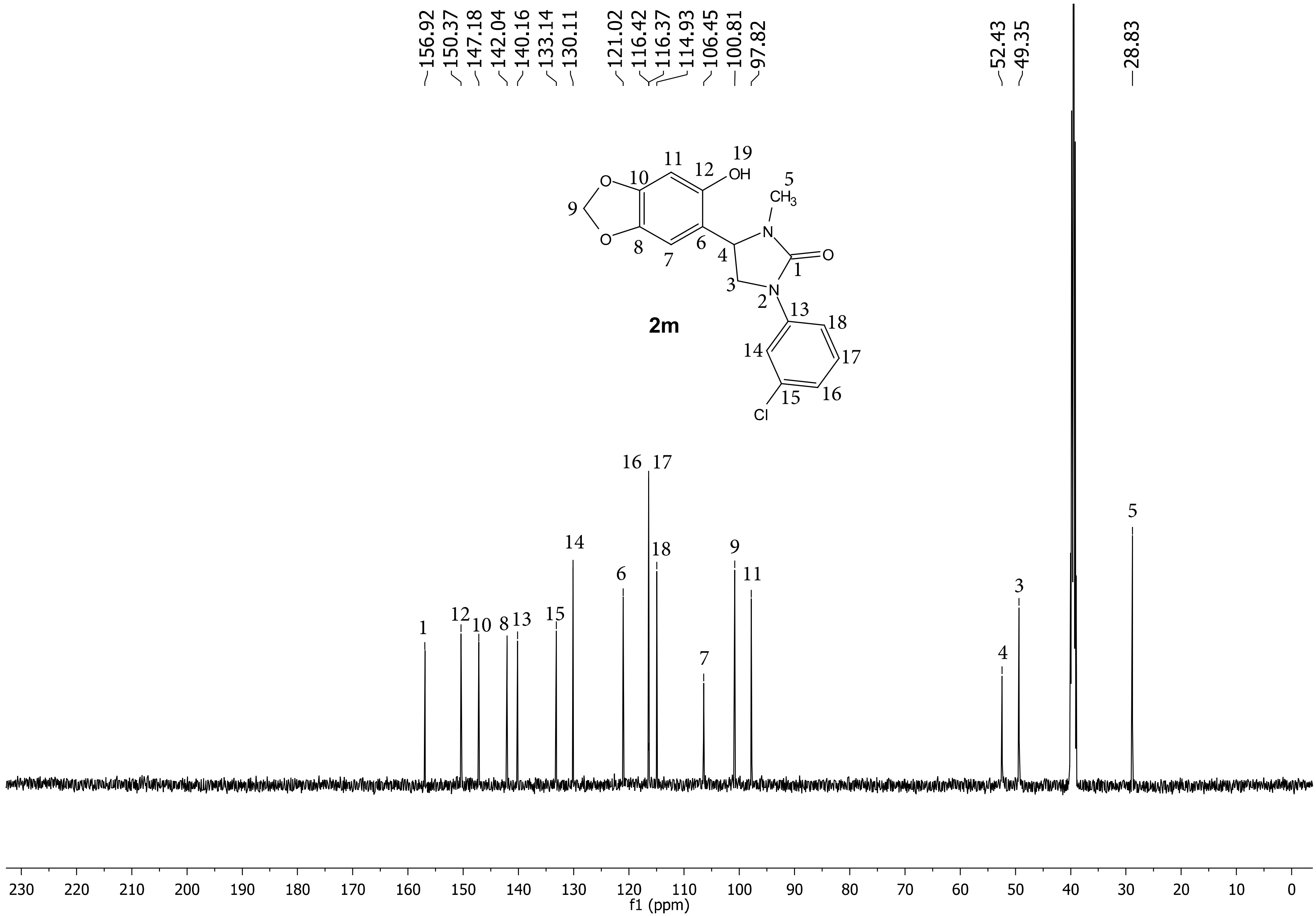

Figure S45. ^{13}C NMR spectrum (DMSO-d_6, 151MHz, 303K) of the compound **2m**

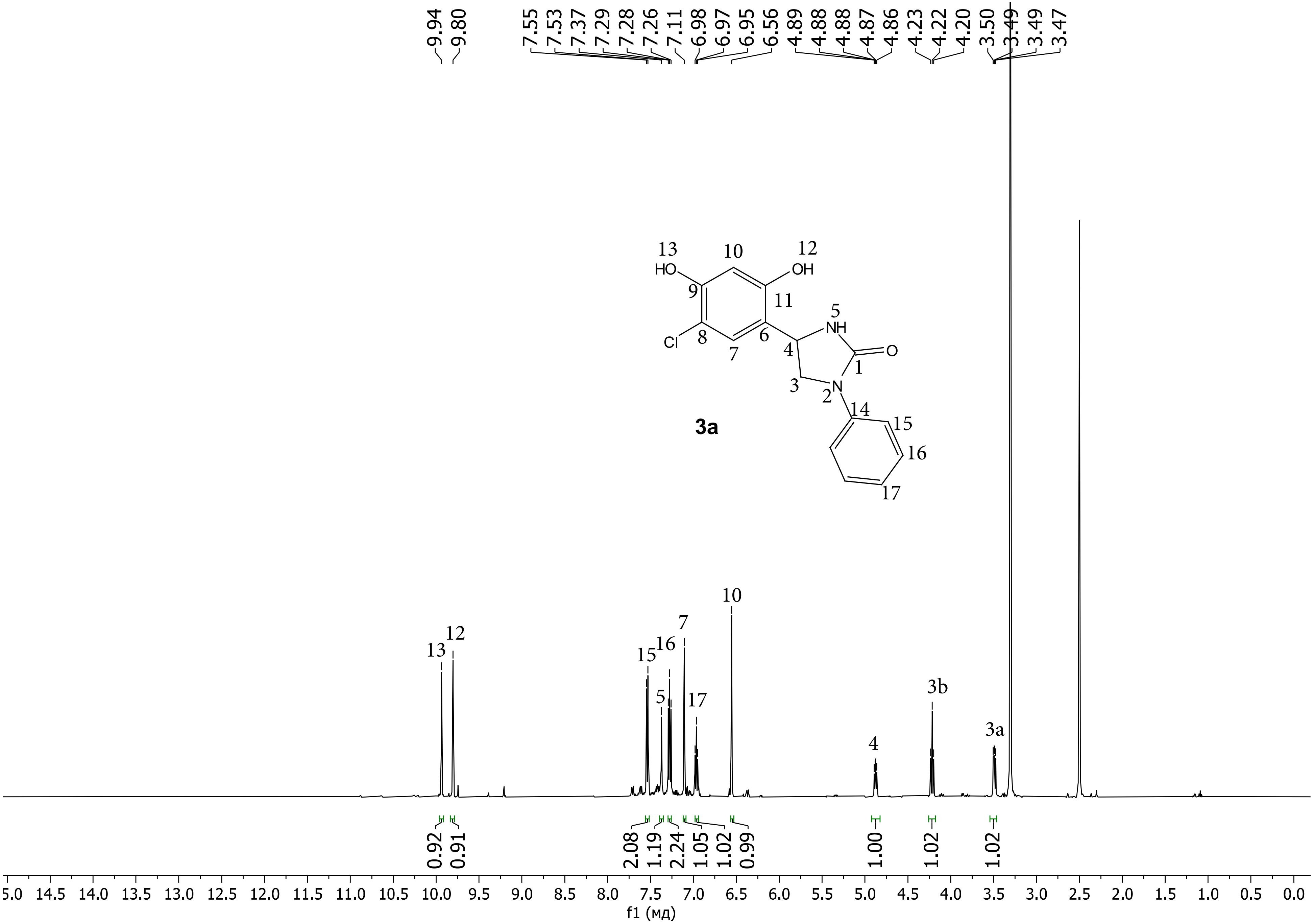

Figure S46. ^{1}H NMR spectrum (DMSO-d_6, 400MHz, 303K) of the compound **3a**

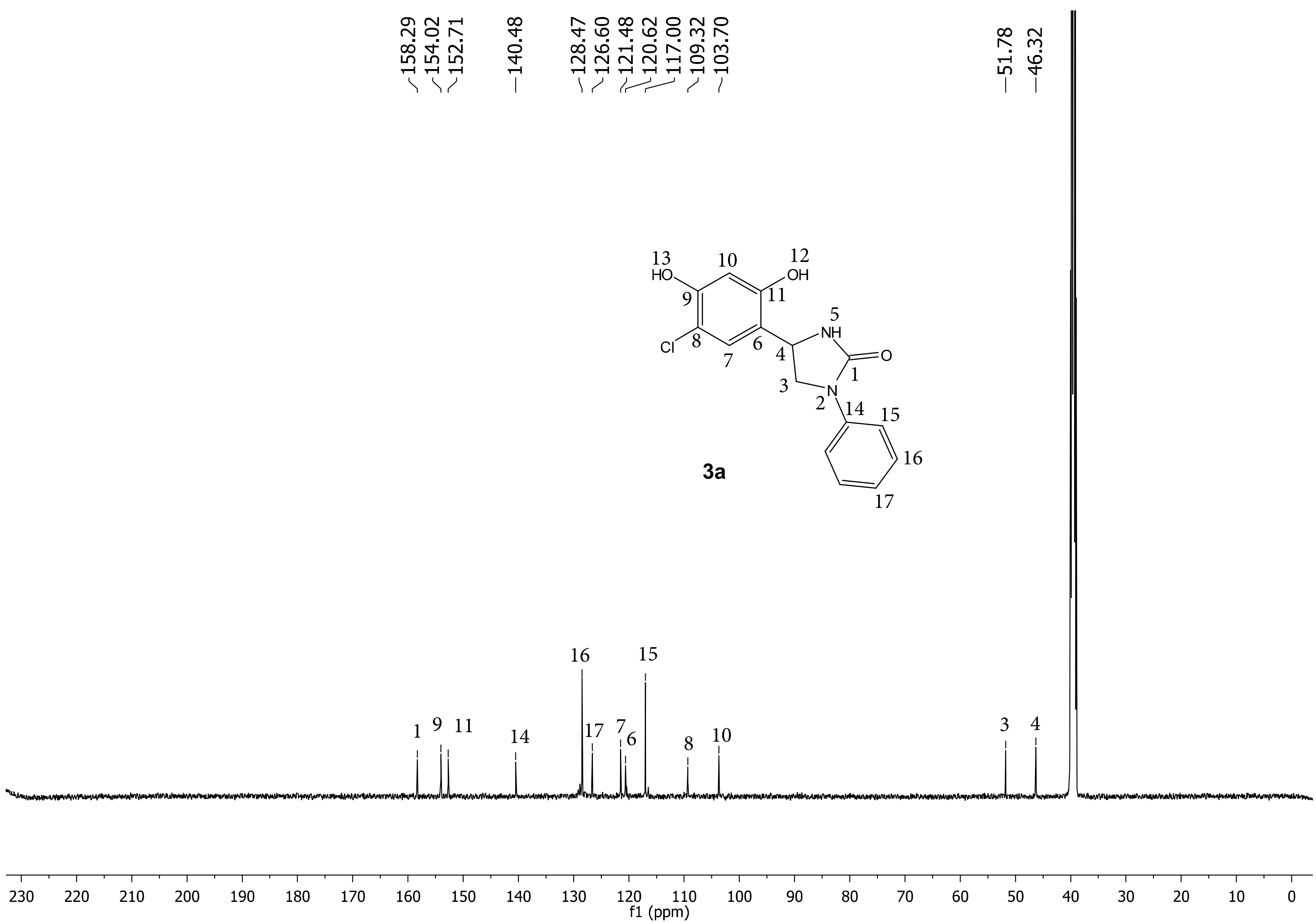

Figure S47. ^{13}C NMR spectrum (DMSO-d_6, 151MHz, 303K) of the compound **3a**

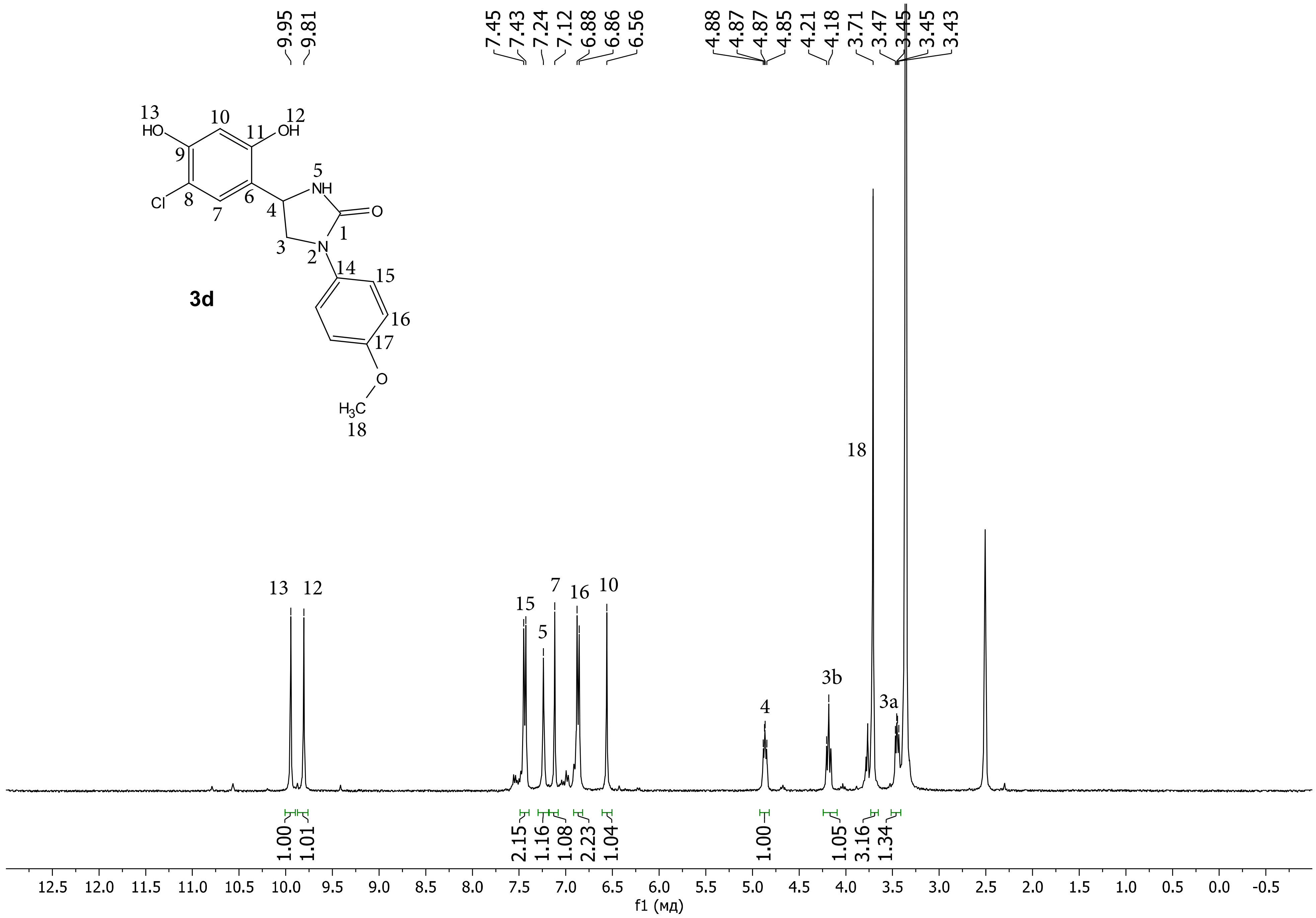

Figure S48. ^{1}H NMR spectrum (DMSO-d_6, 400MHz, 303K) of the compound **3d**

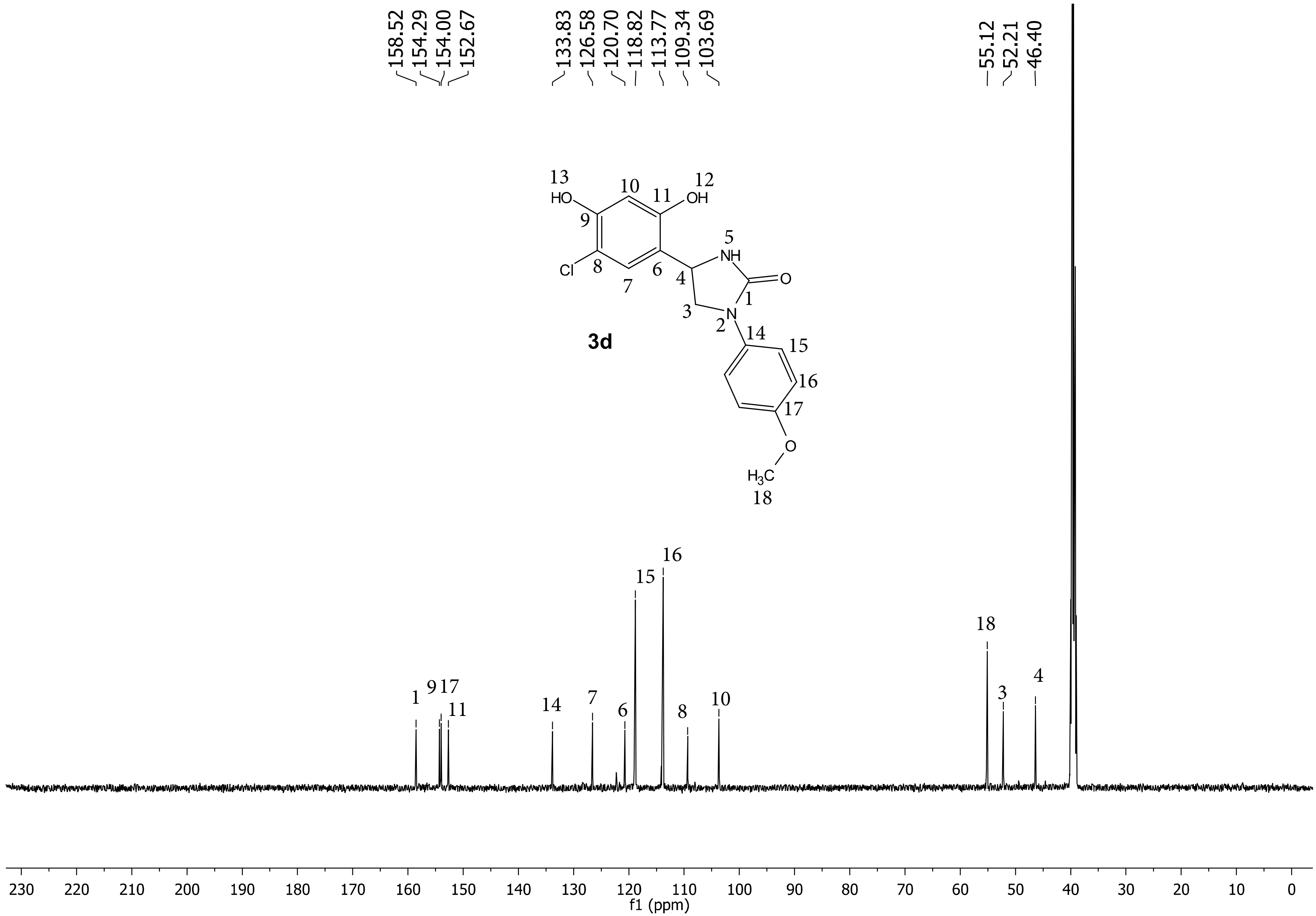

Figure S49. ^{13}C NMR spectrum (DMSO-d_6, 151MHz, 303K) of the compound **3d**

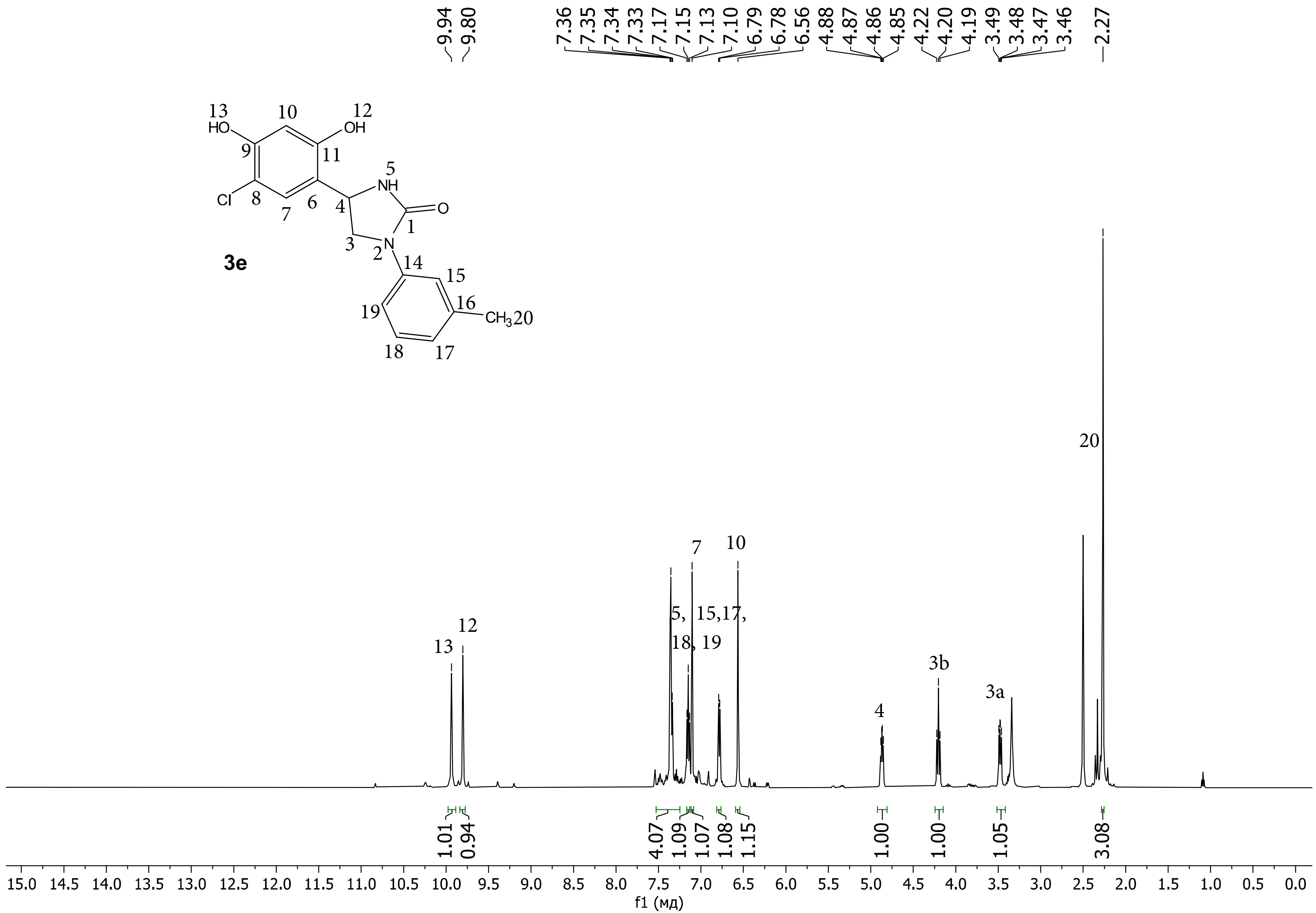

Figure S50. ^{1}H NMR spectrum (DMSO-d_6, 400MHz, 303K) of the compound **3e**

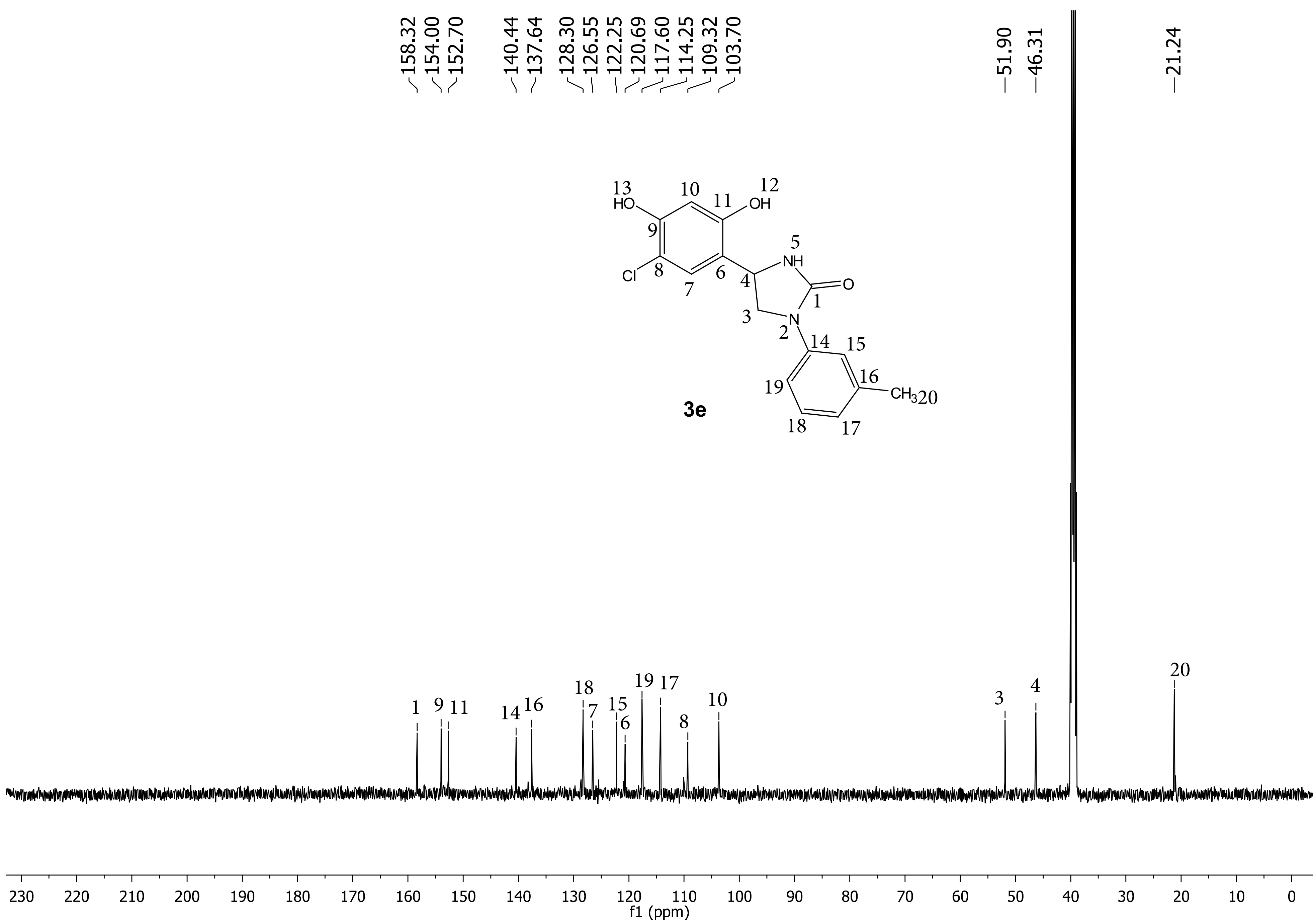

Figure S51. ^{13}C NMR spectrum (DMSO-d_6, 151MHz, 303K) of the compound **3e**

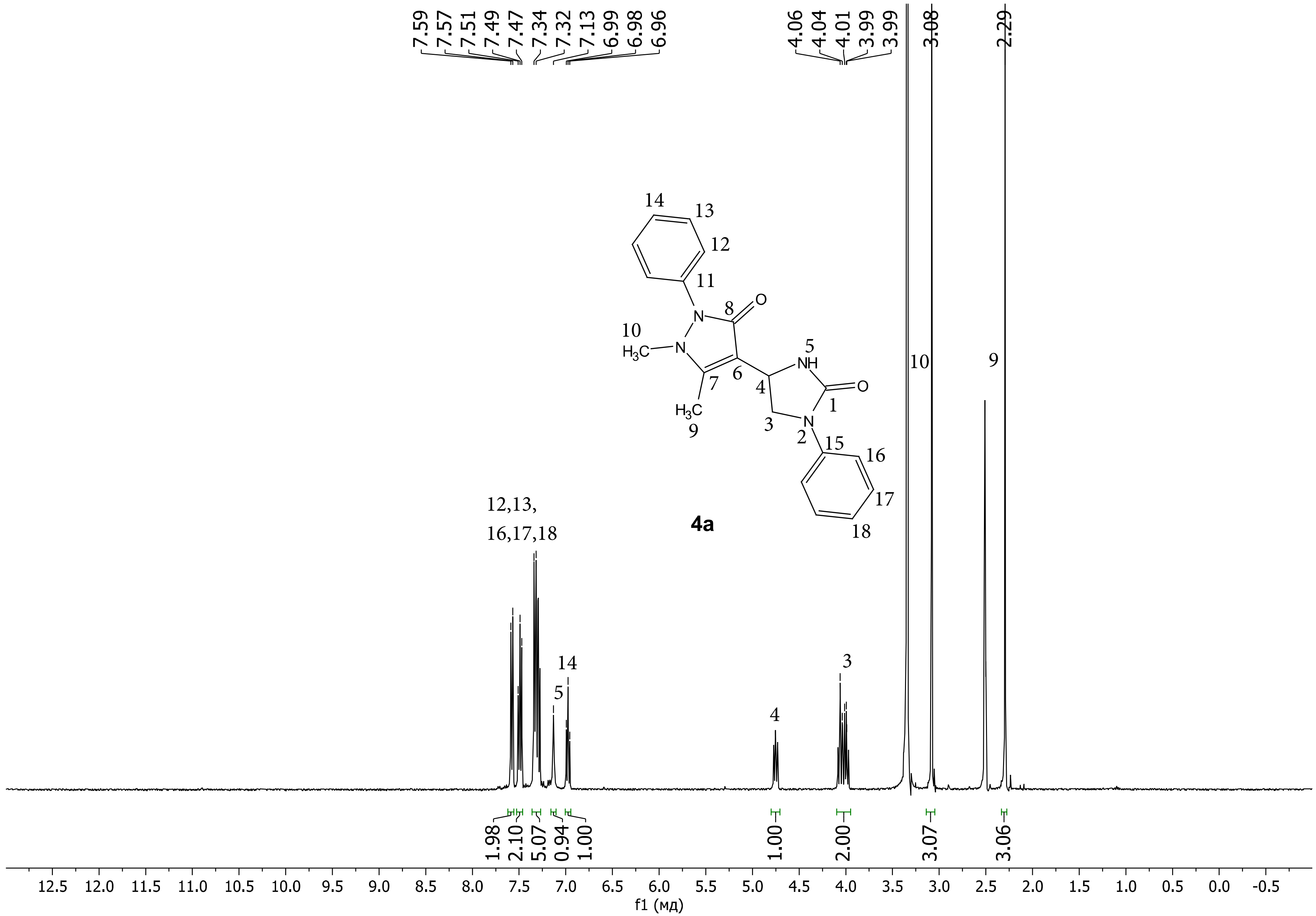

Figure S52. ^1H NMR spectrum (DMSO-d_6, 400MHz, 303K) of the compound **4a**

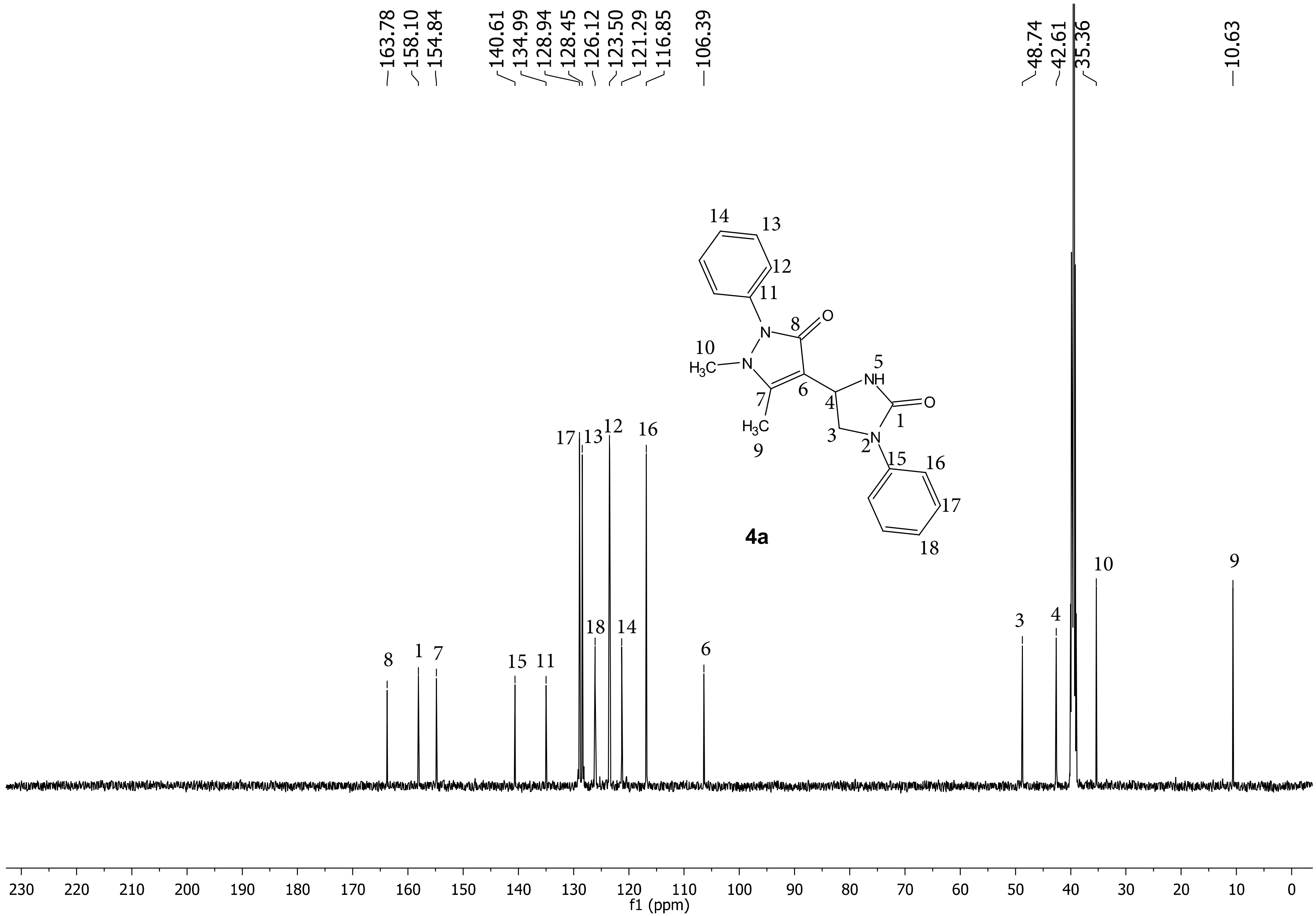

Figure S53. ^{13}C NMR spectrum (DMSO-d_6, 151MHz, 303K) of the compound **4a**

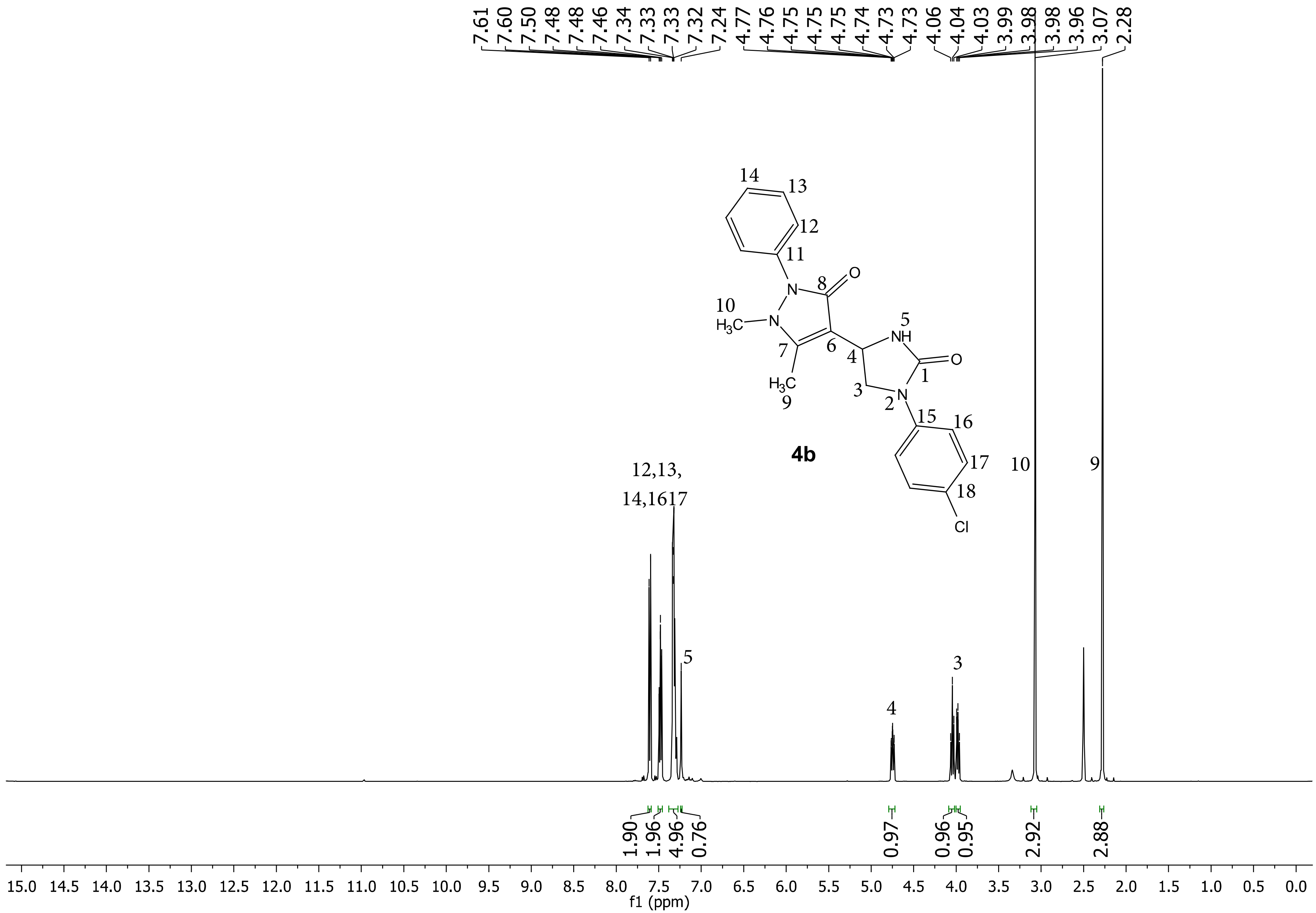

Figure S54. ^{1}H NMR spectrum (DMSO-d_6, 400MHz, 303K) of the compound **4b**

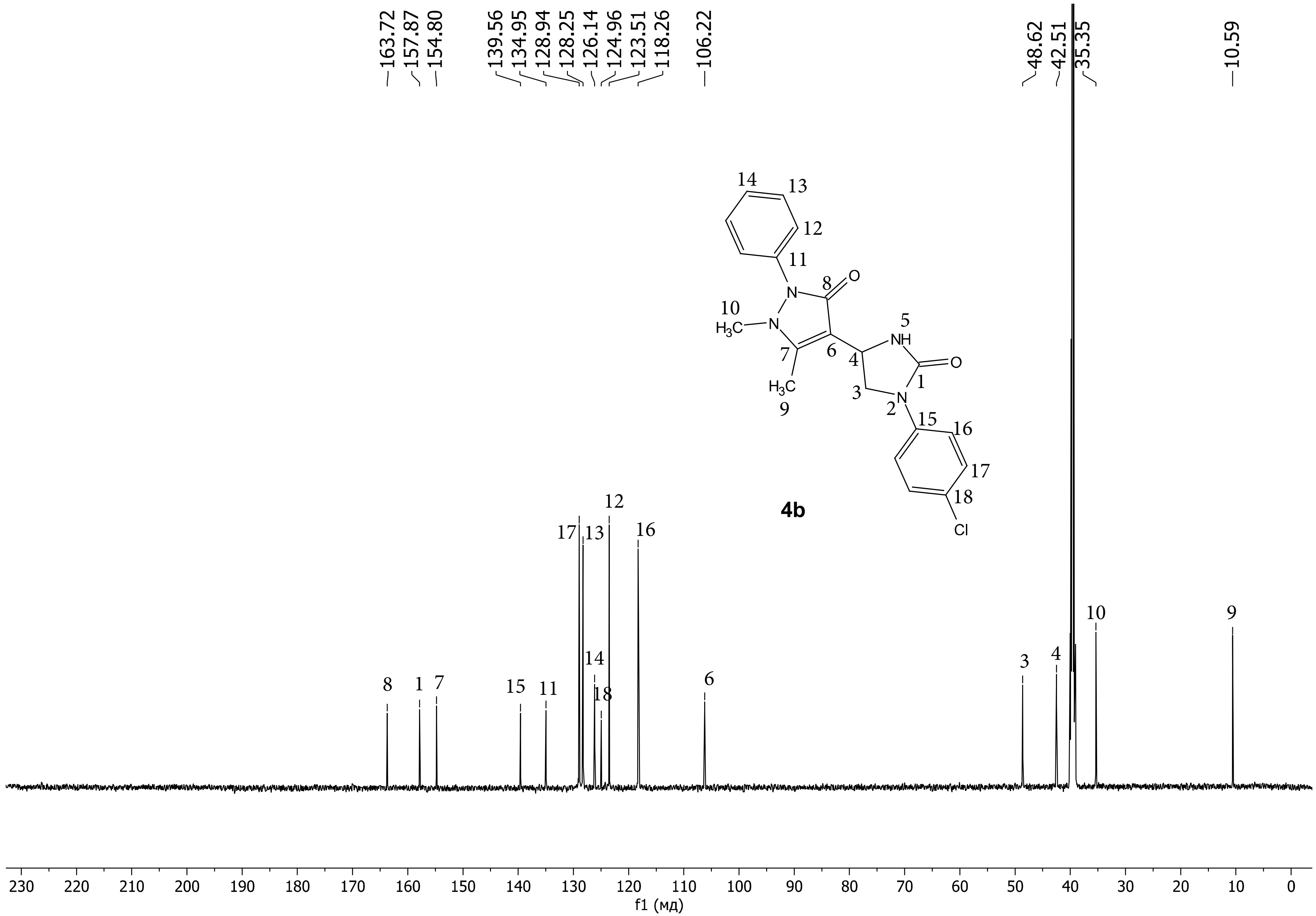

Figure S55. ^{13}C NMR spectrum (DMSO-d_6, 151MHz, 303K) of the compound **4b**

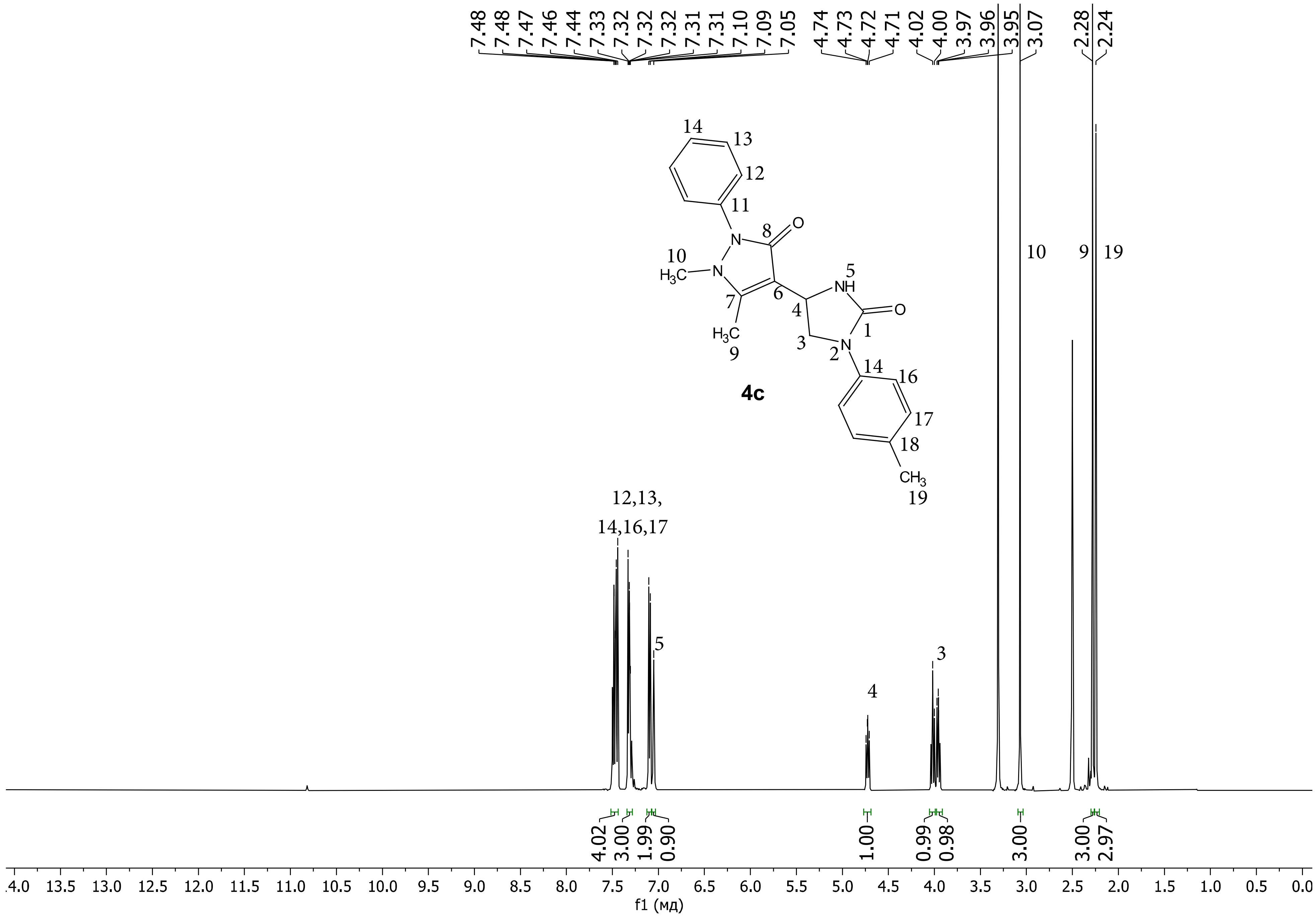

Figure S56. ^{1}H NMR spectrum (DMSO-d_6, 400MHz, 303K) of the compound **4c**

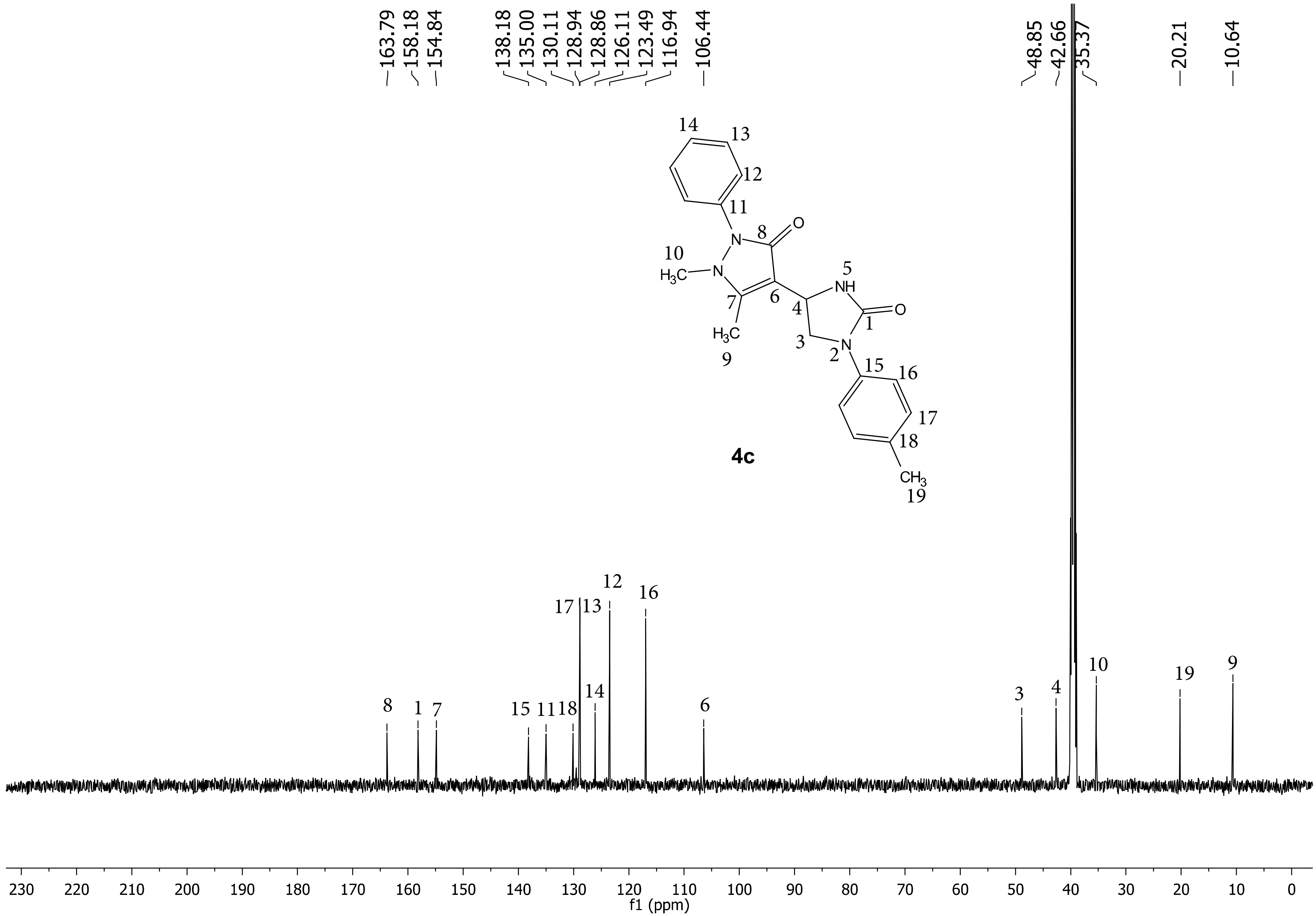

Figure S57. ^{13}C NMR spectrum (DMSO-d_6, 151MHz, 303K) of the compound **4c**

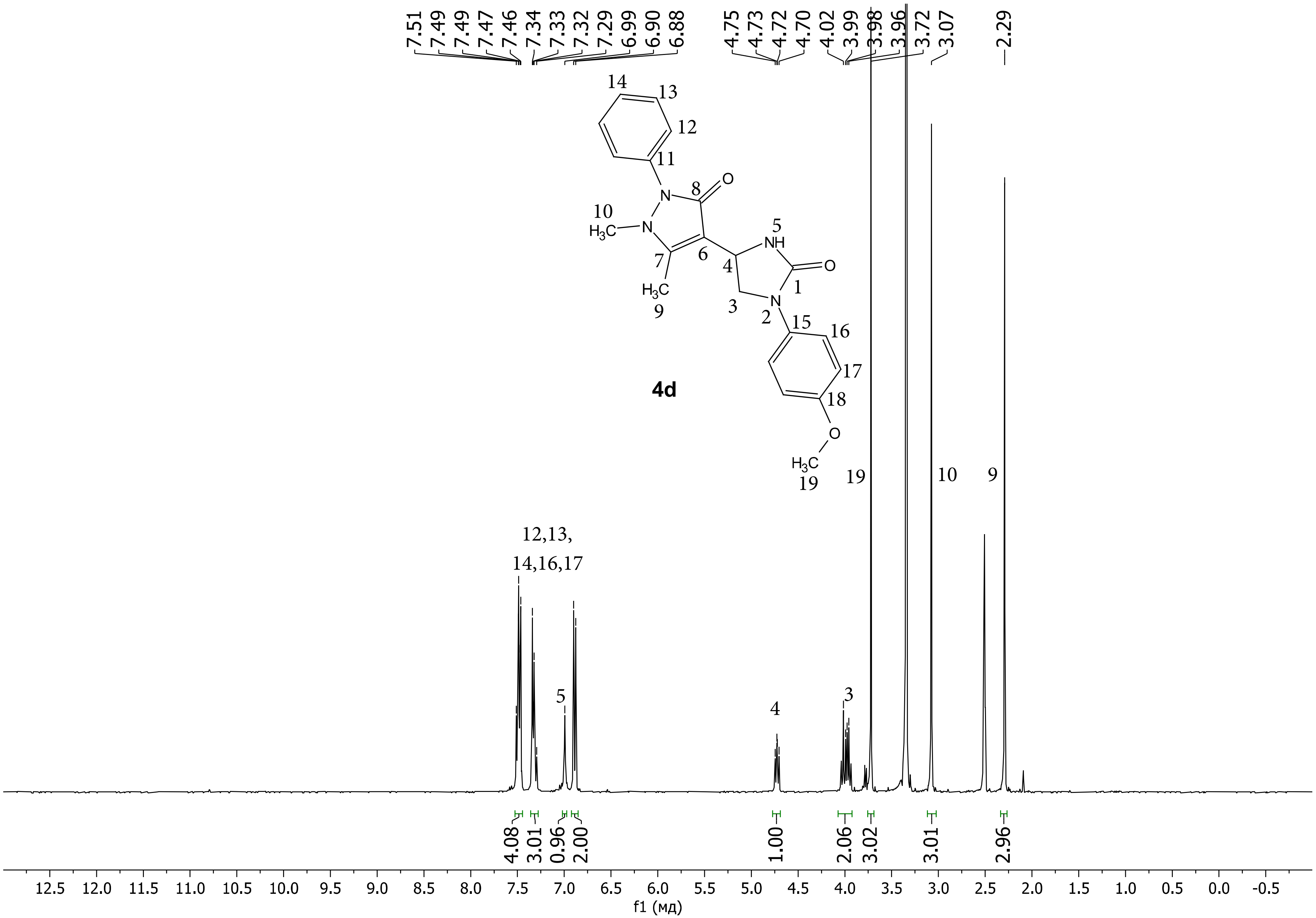

Figure S58. ^{1}H NMR spectrum (DMSO-d_6, 400MHz, 303K) of the compound **4d**

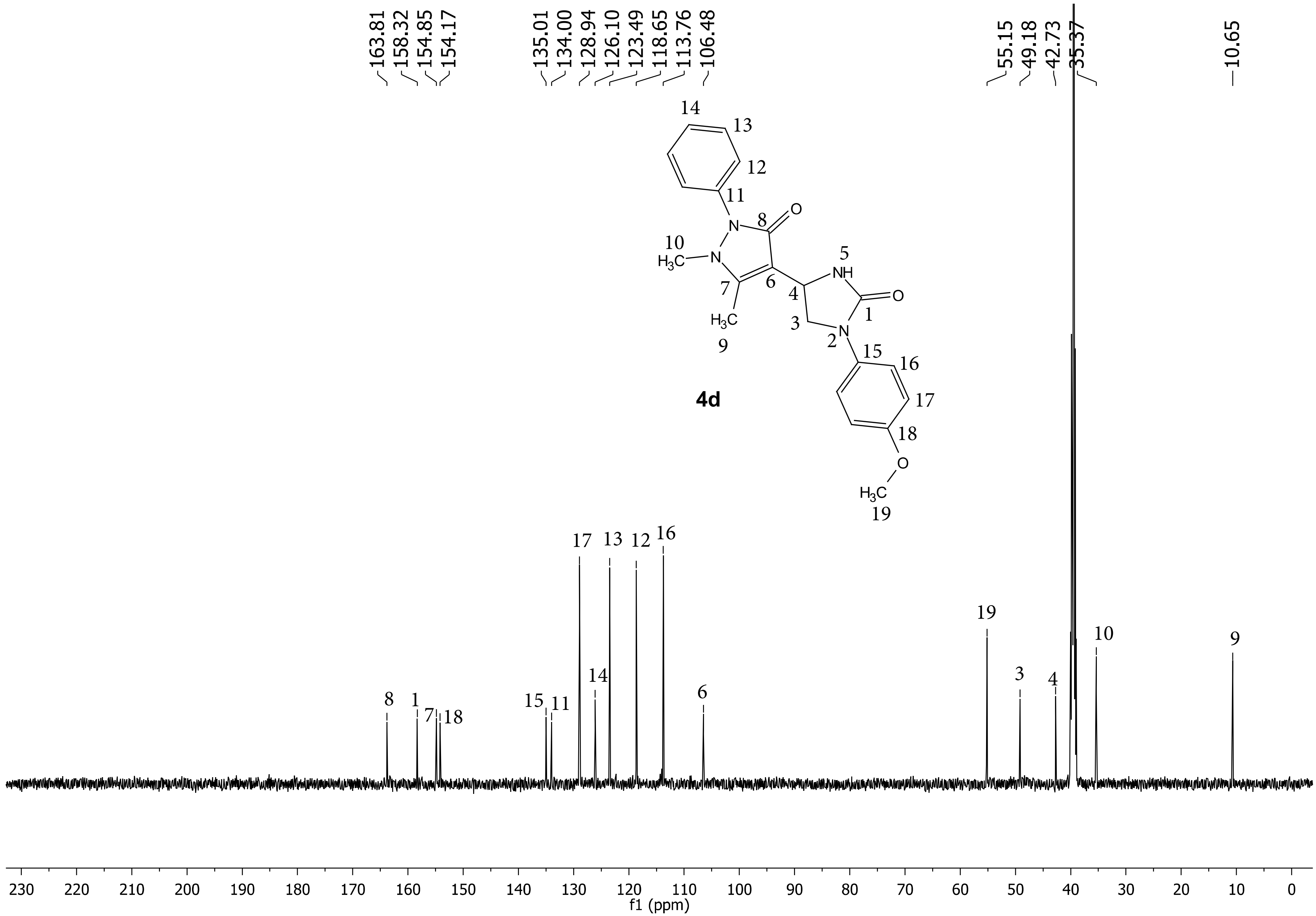

Figure S59. ^{13}C NMR spectrum (DMSO-d_6, 151MHz, 303K) of the compound **4d**

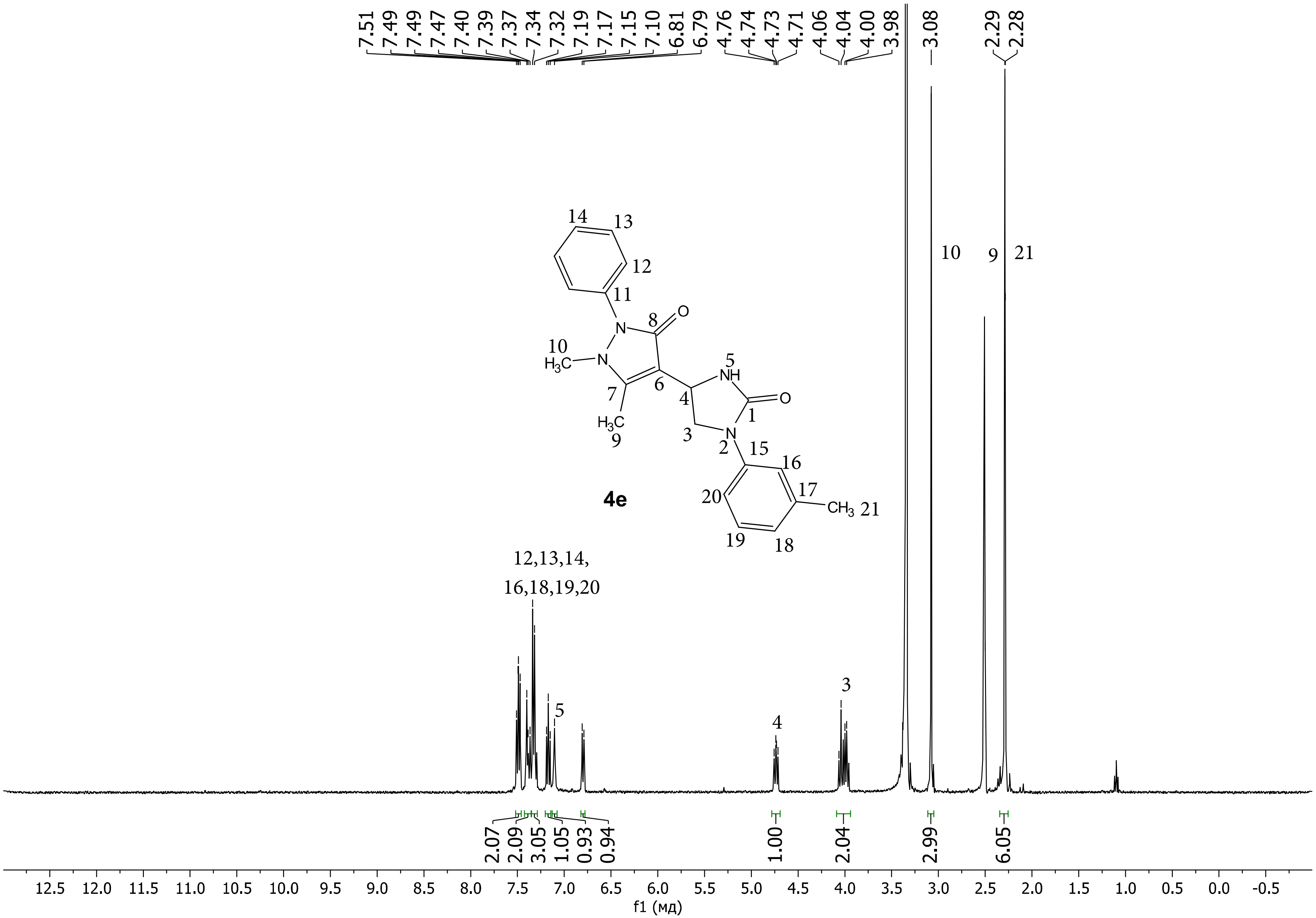

Figure S60. ^{1}H NMR spectrum (DMSO-d_6, 400MHz, 303K) of the compound **4e**

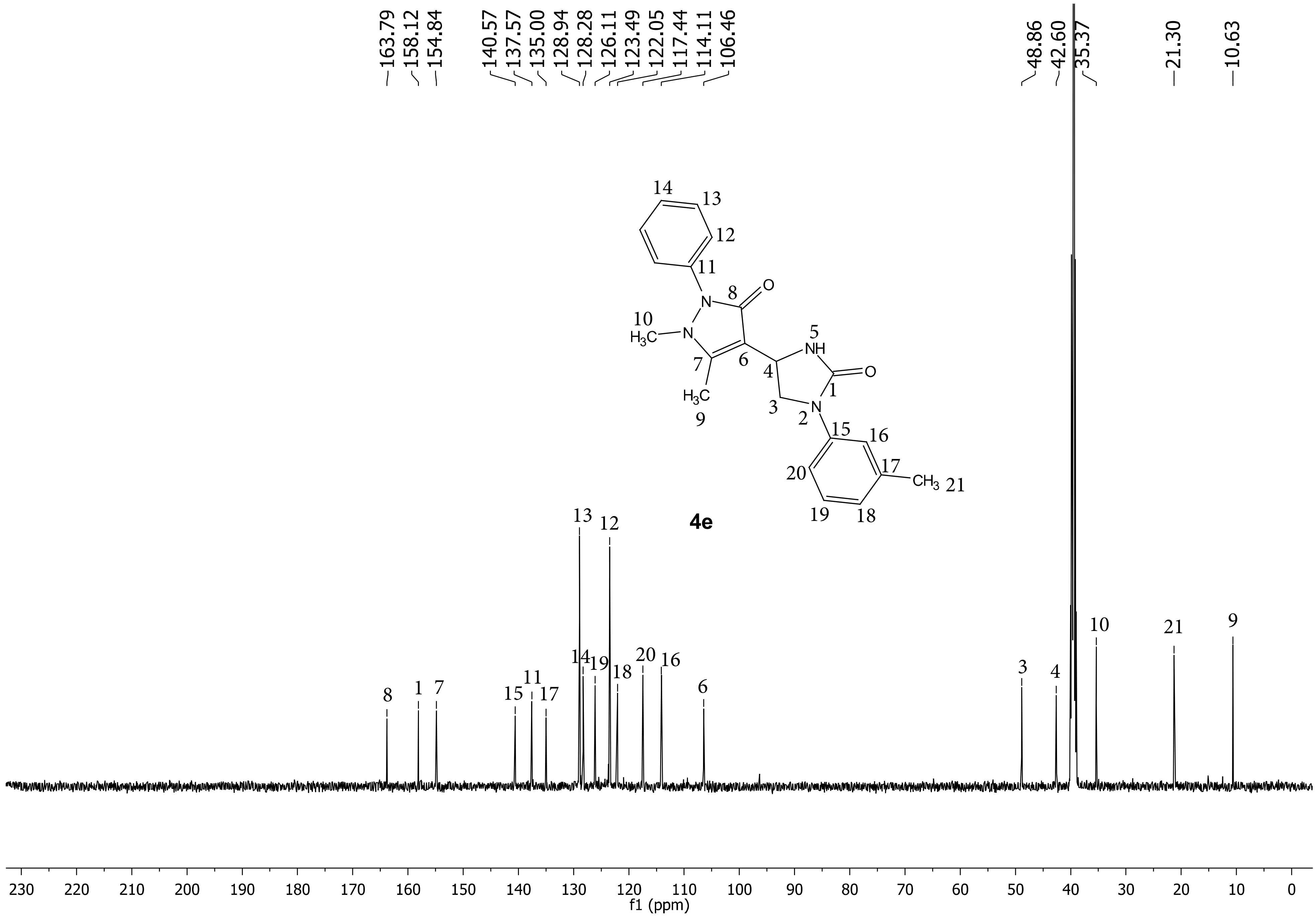

Figure S61. ^{13}C NMR spectrum (DMSO-d_6, 151MHz, 303K) of the compound **4e**

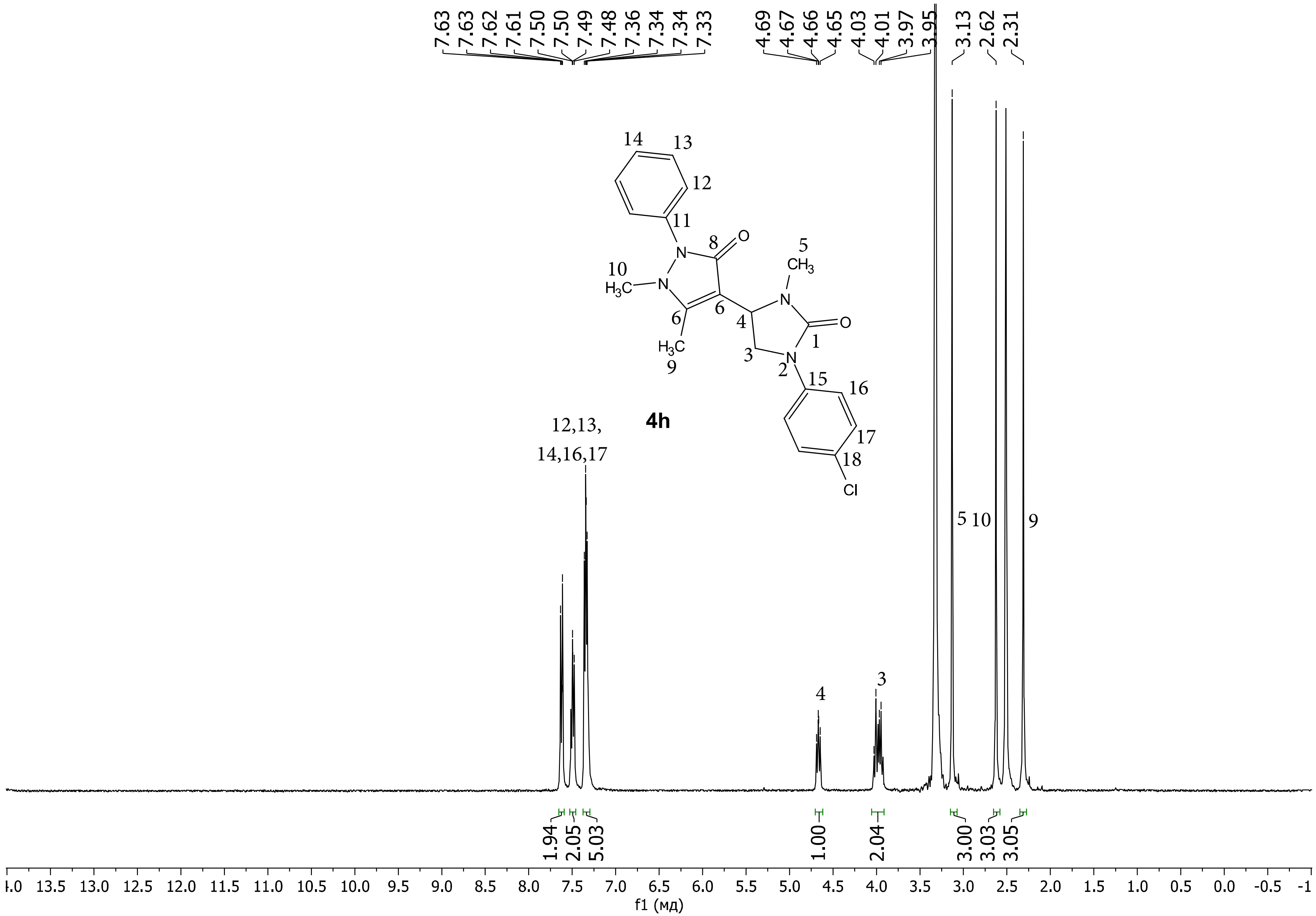

Figure S62. ^{1}H NMR spectrum (DMSO-d_6, 400MHz, 303K) of the compound **4h**

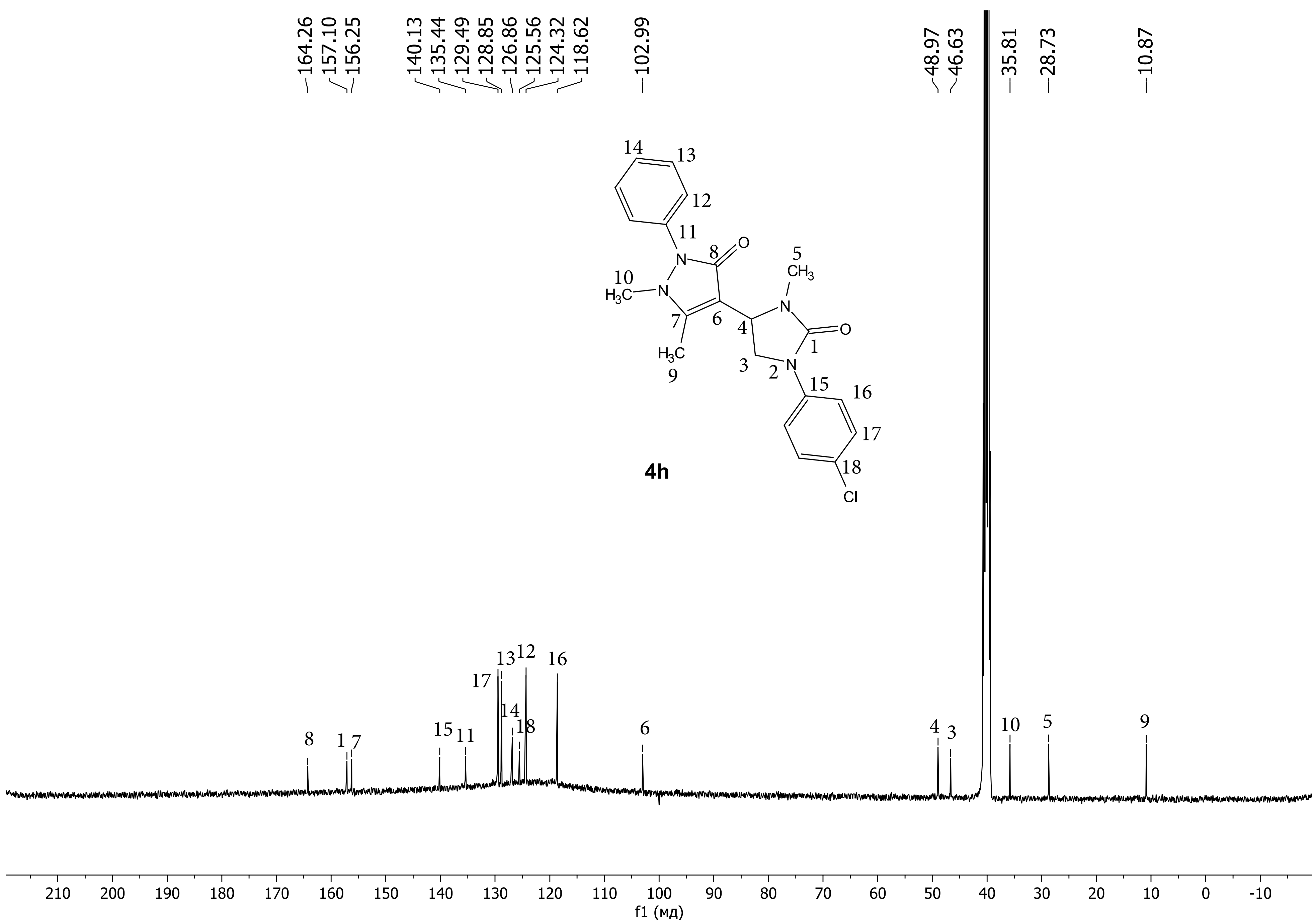

Figure S63. ^{13}C NMR spectrum (DMSO-d_6, 151MHz, 303K) of the compound **4h**

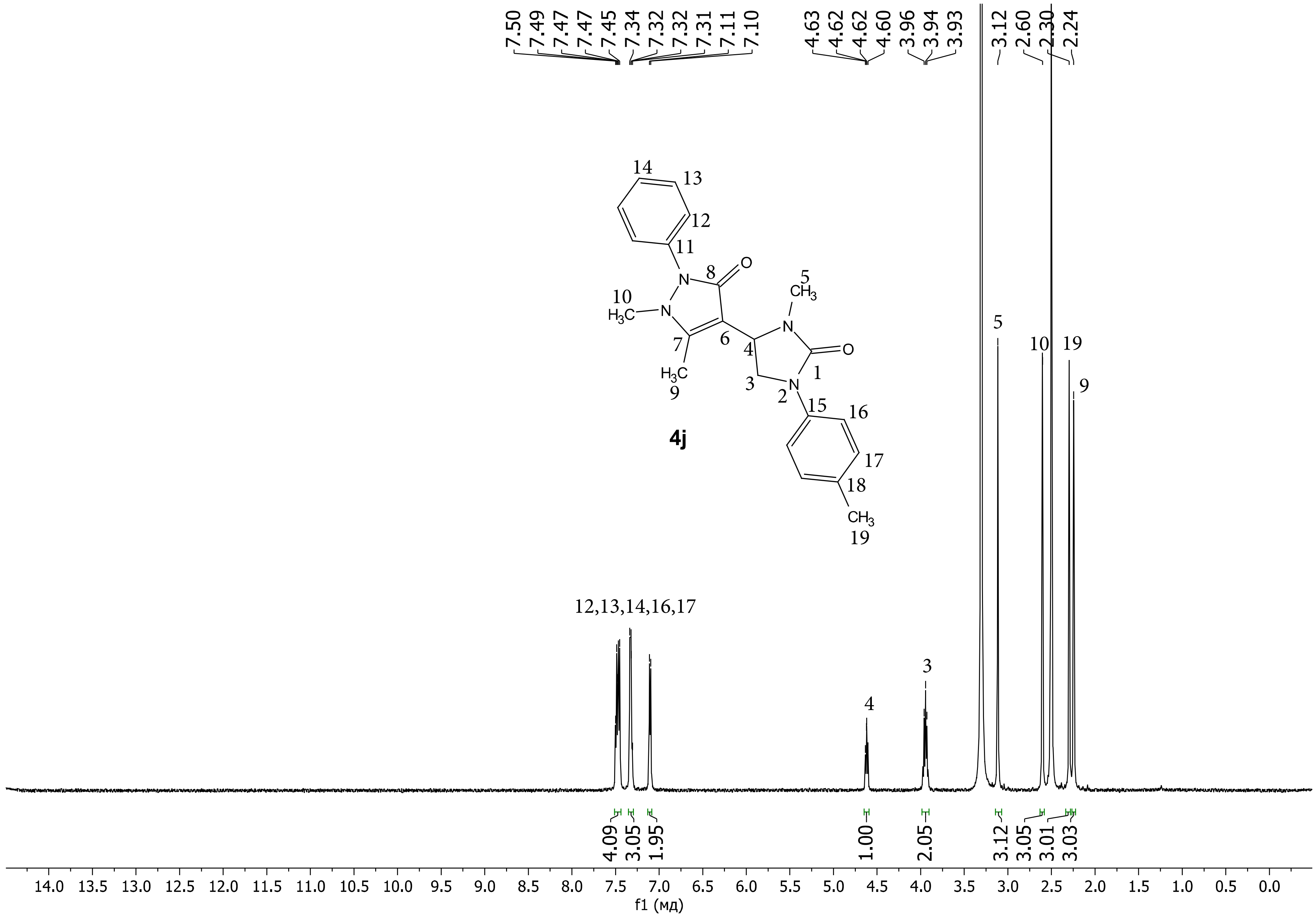

Figure S64. ^{1}H NMR spectrum (DMSO-d_6, 400MHz, 303K) of the compound **4j**

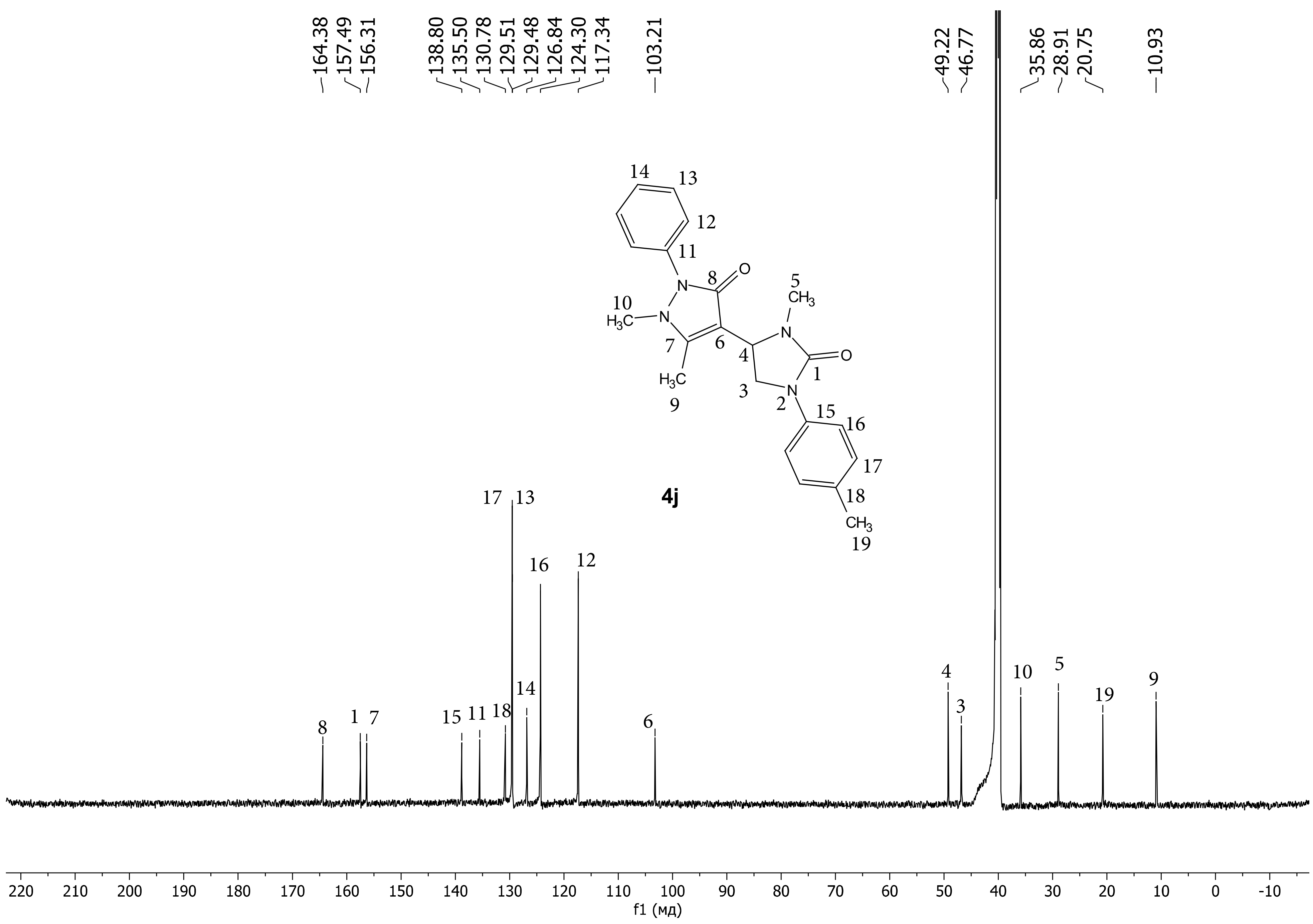

Figure S65. ^{13}C NMR spectrum (DMSO-d_6, 151MHz, 303K) of the compound **4j**

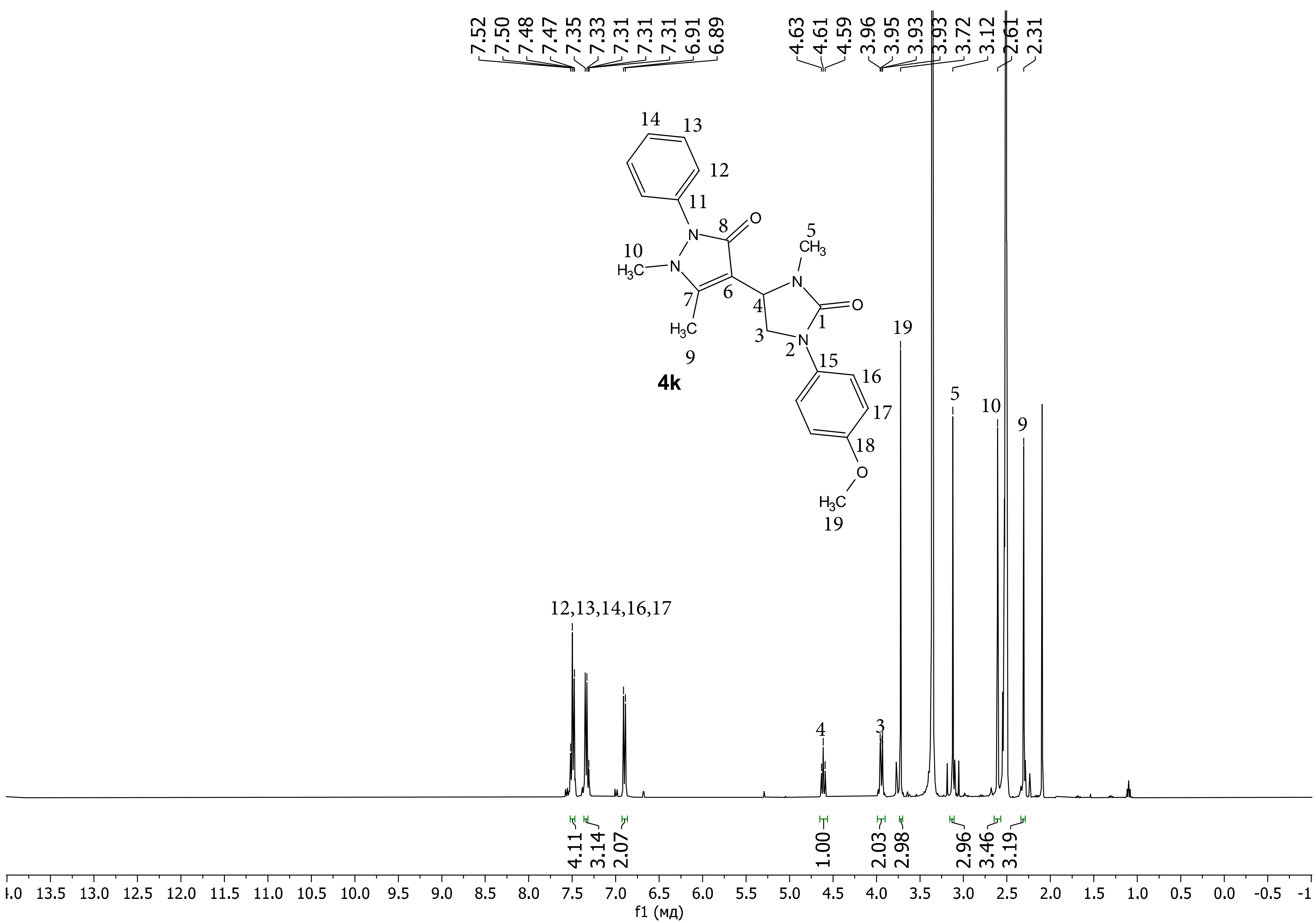

Figure S66. ^{1}H NMR spectrum (DMSO-d_6, 400MHz, 303K) of the compound **4k**

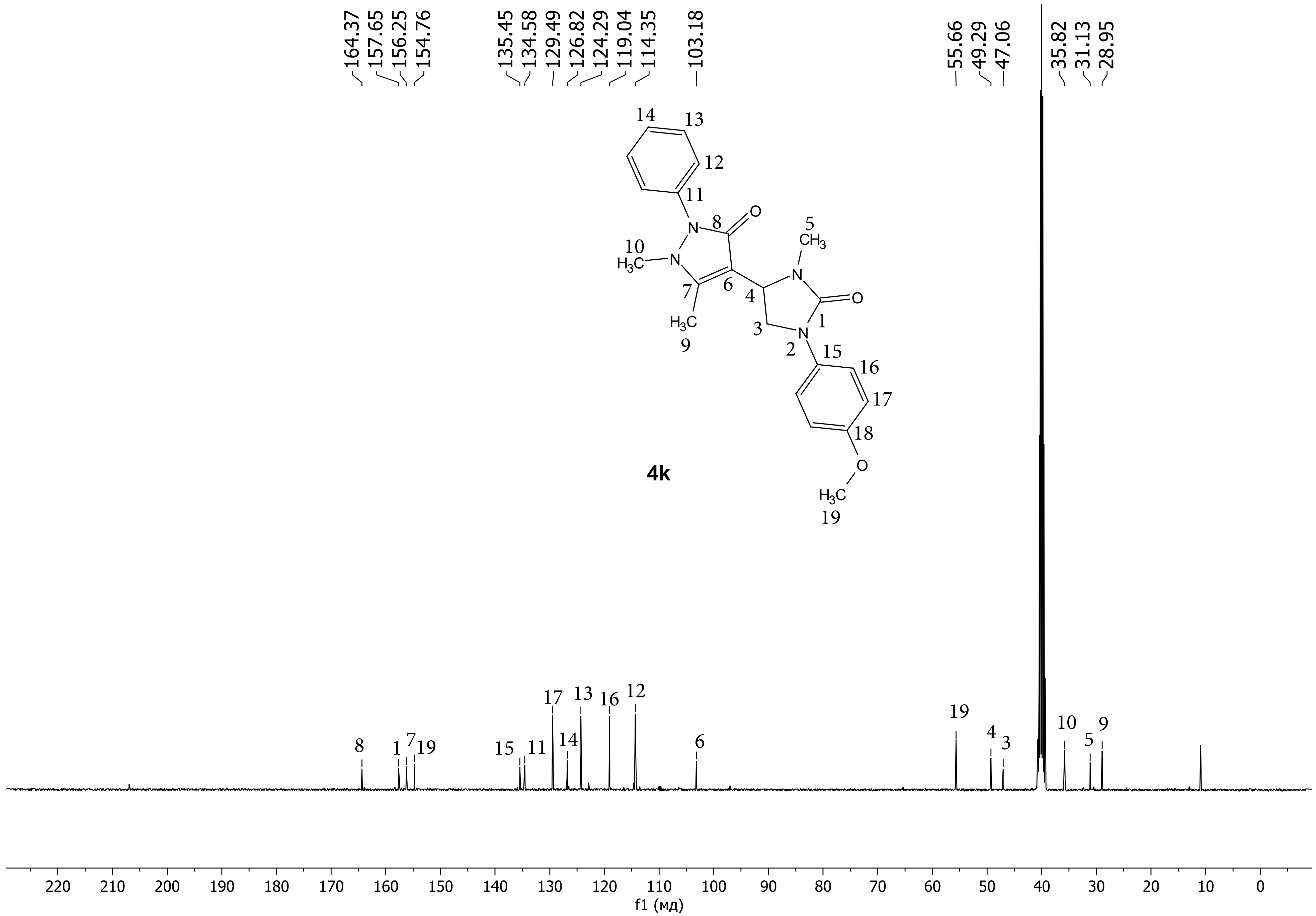

Figure S67. ^{13}C NMR spectrum (DMSO-d_6, 151MHz, 303K) of the compound **4k**

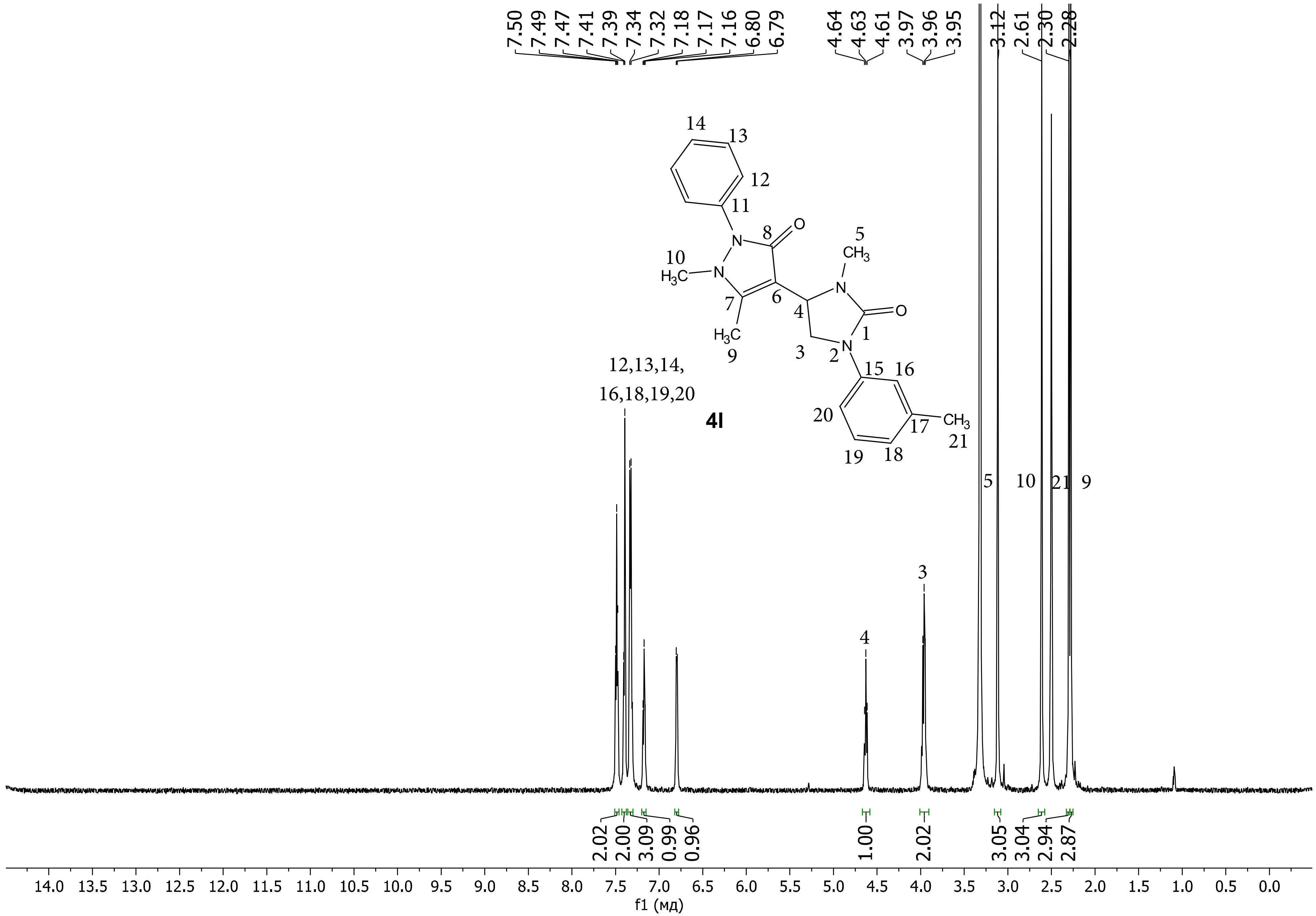

Figure S68. ^{1}H NMR spectrum (DMSO-d_6, 400MHz, 303K) of the compound **4l**

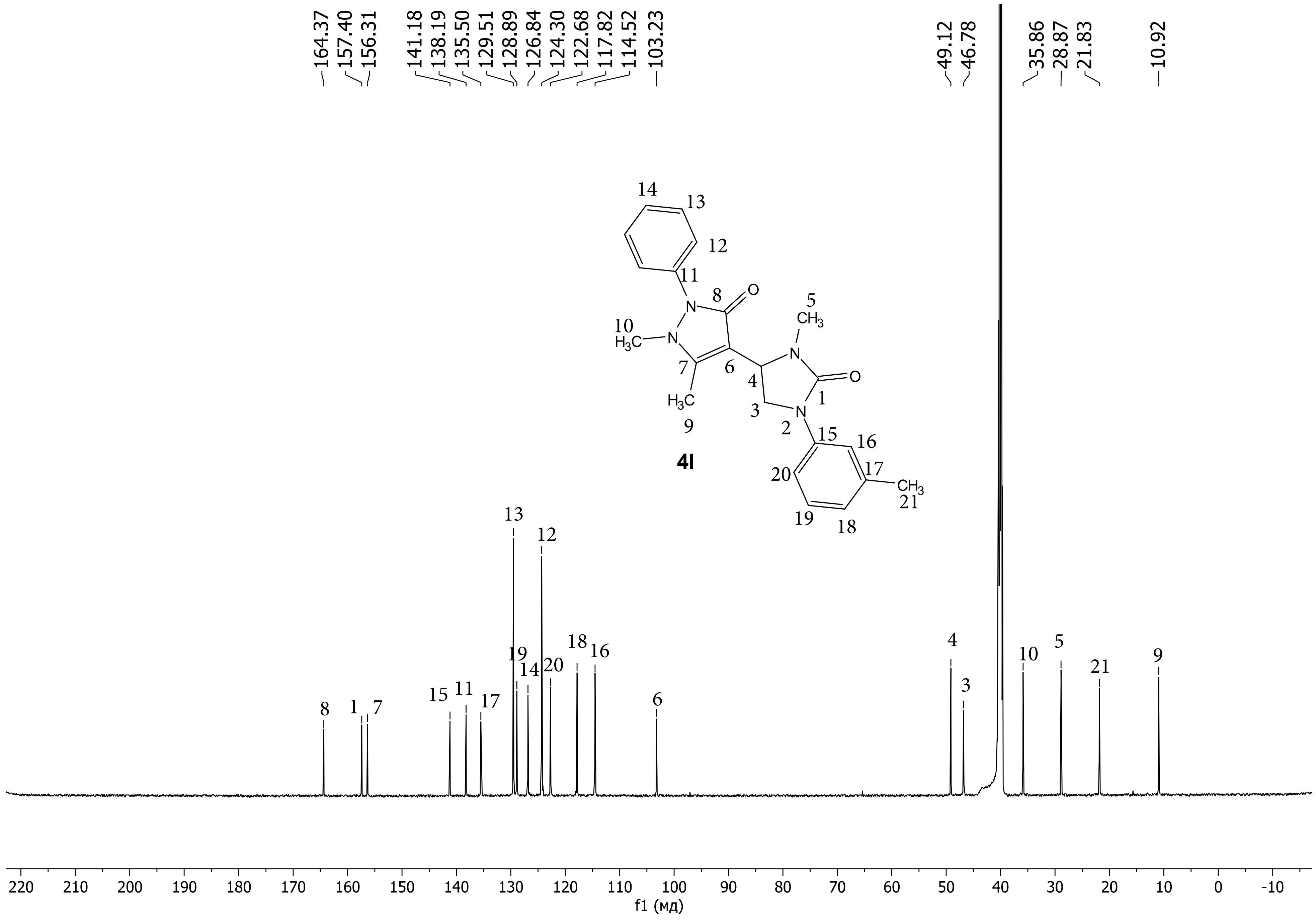

Figure S69. ^{13}C NMR spectrum (DMSO-d_6, 151MHz, 303K) of the compound **4l**

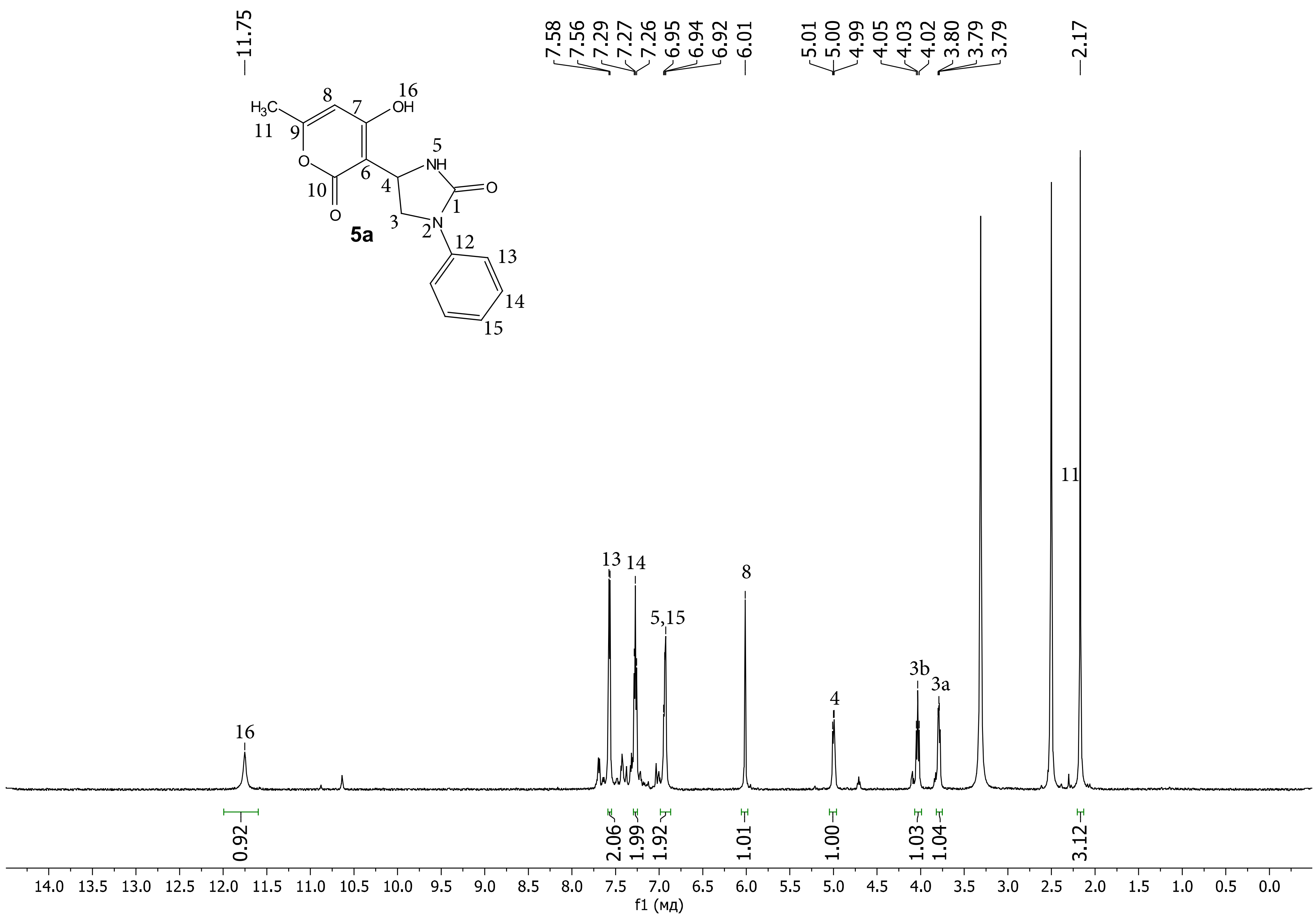

Figure S70. ^{1}H NMR spectrum (DMSO-d_6, 400MHz, 303K) of the compound **5a**

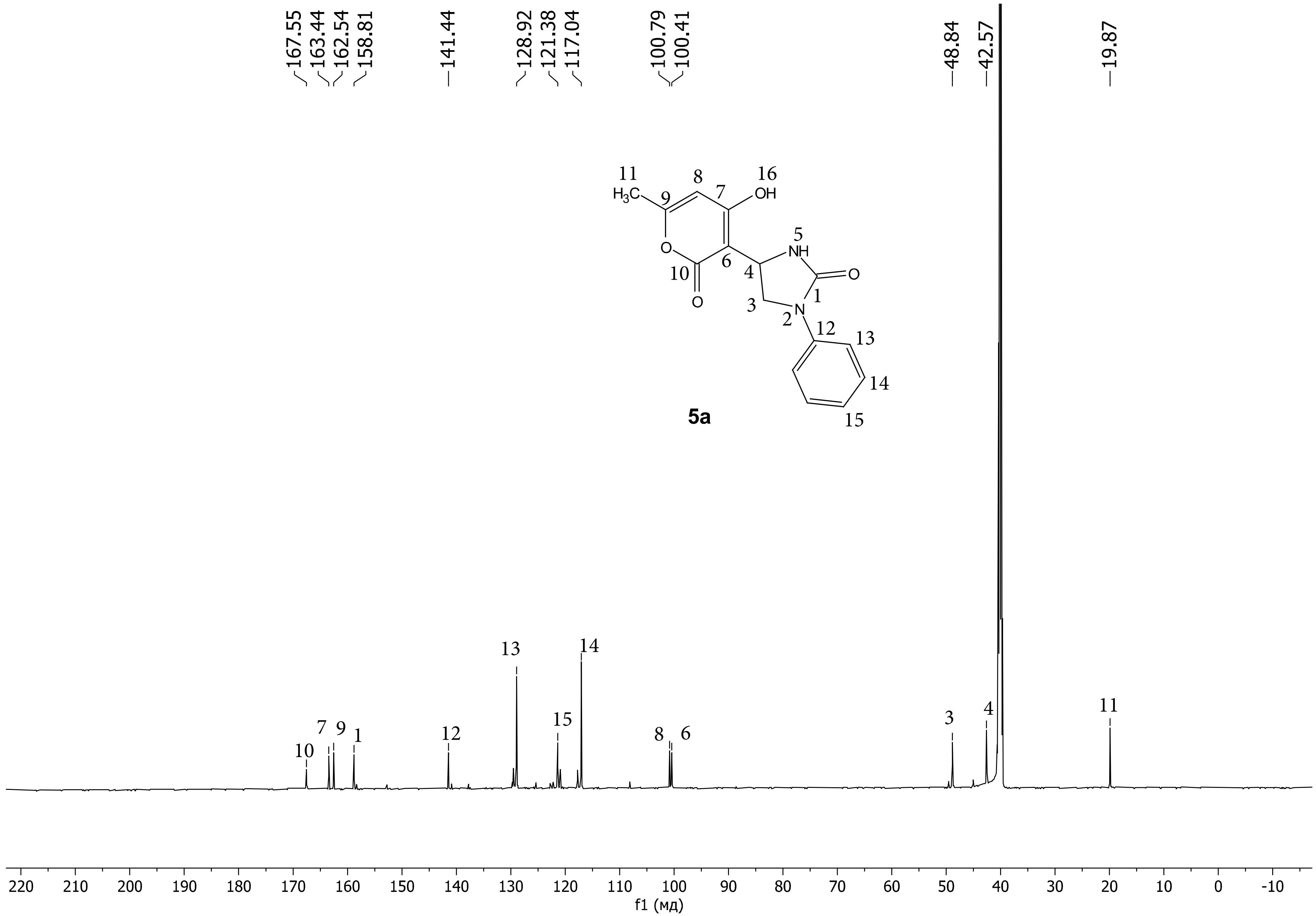

Figure S71. ^{13}C NMR spectrum (DMSO-d_6, 151MHz, 303K) of the compound **5a**

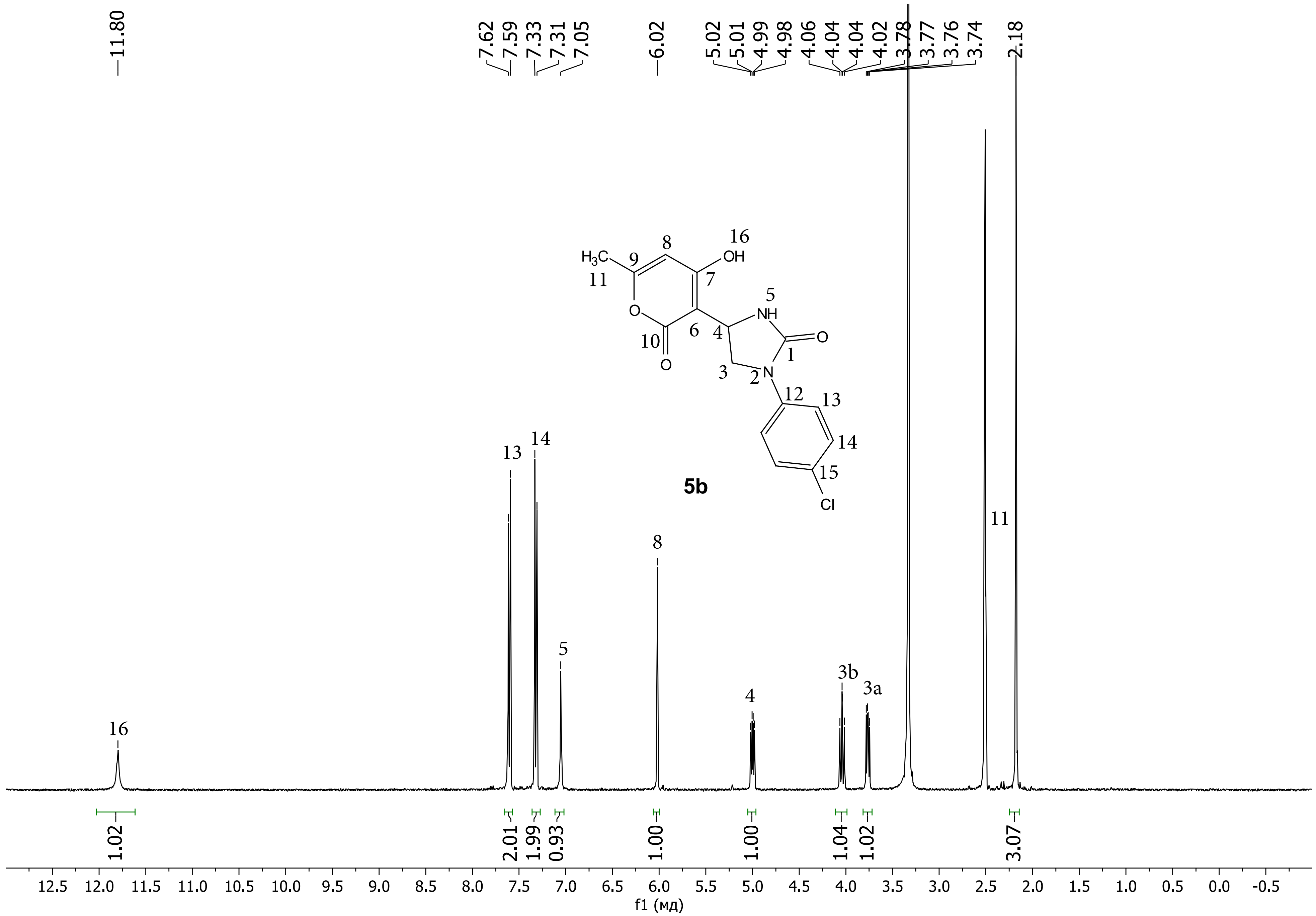

S74

Figure S72. ^{1}H NMR spectrum (DMSO-d_6, 400MHz, 303K) of the compound **5b**

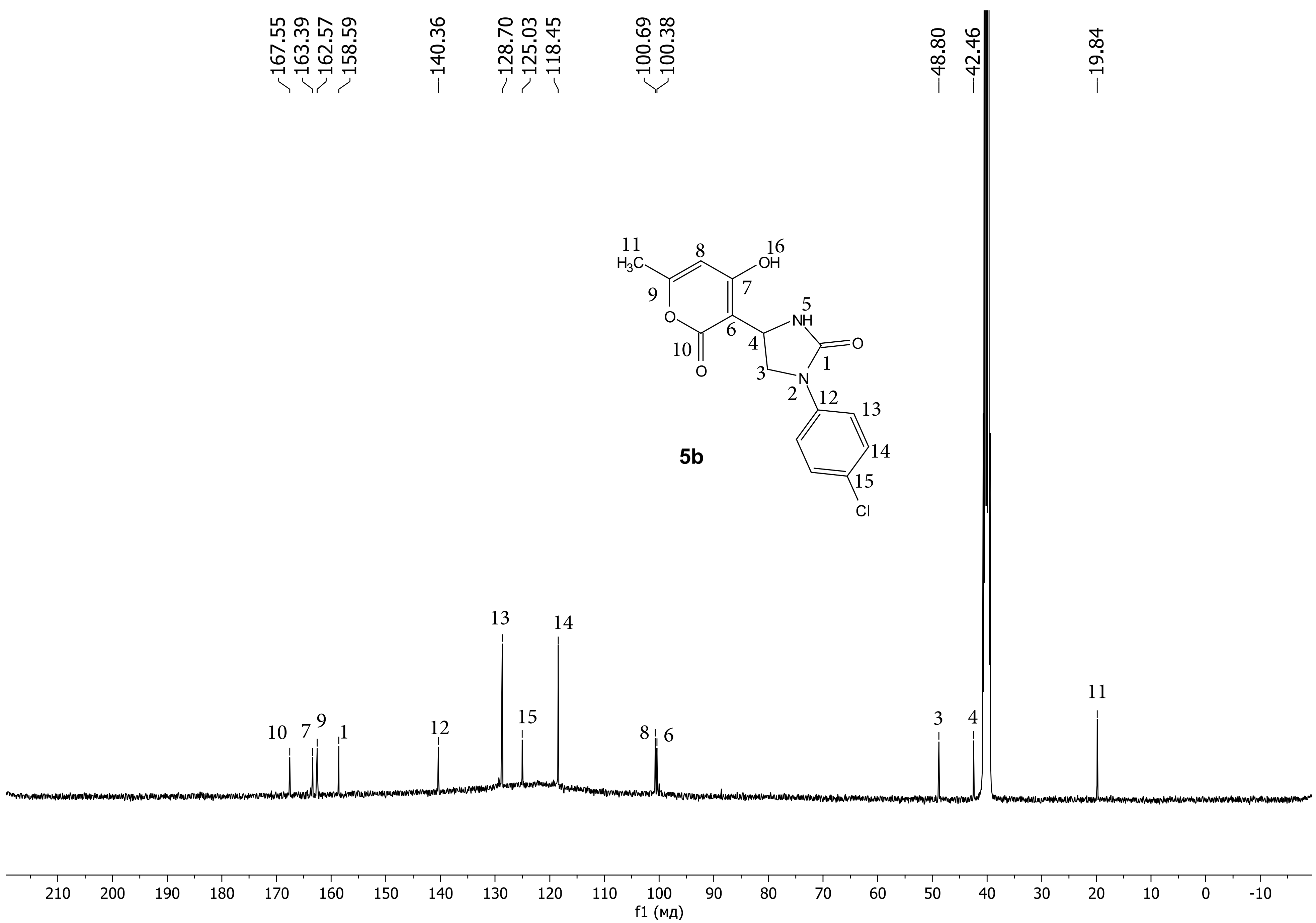

Figure S73. ^{13}C NMR spectrum (DMSO-d_6, 151MHz, 303K) of the compound **5b**

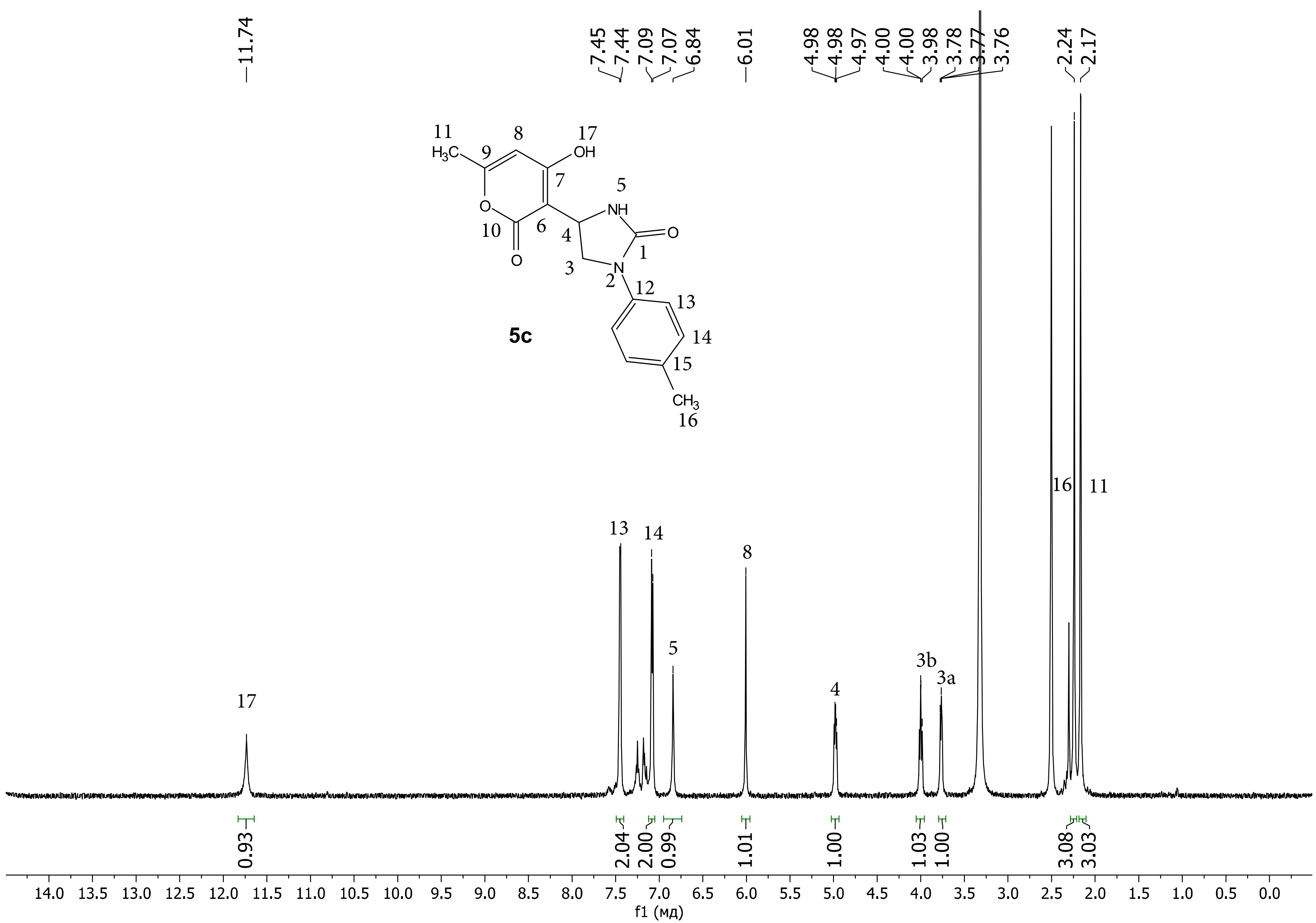

Figure S74. ^{1}H NMR spectrum (DMSO-d_6, 400MHz, 303K) of the compound **5c**

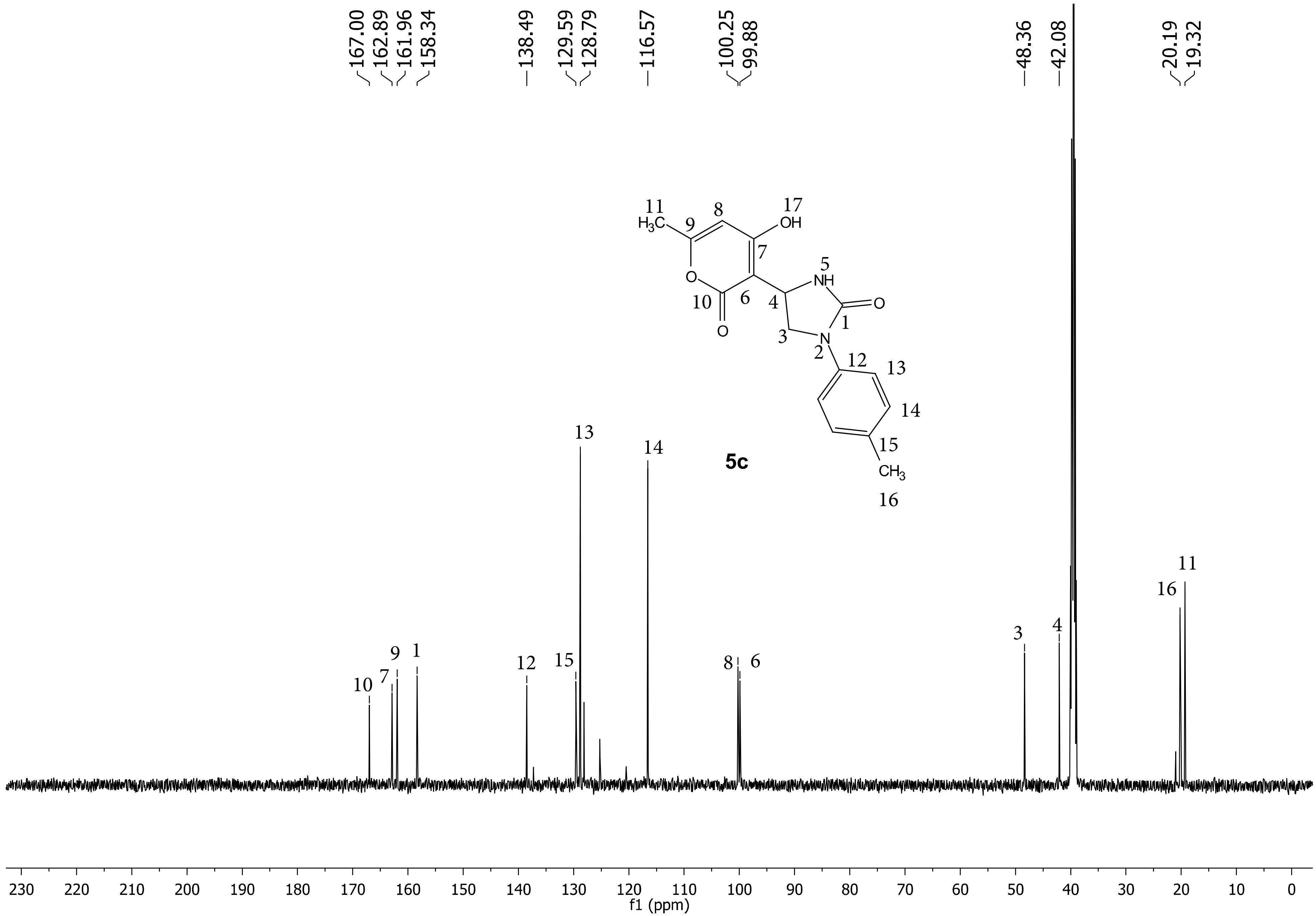

Figure S75. ^{13}C NMR spectrum (DMSO-d_6, 151MHz, 303K) of the compound **5c**

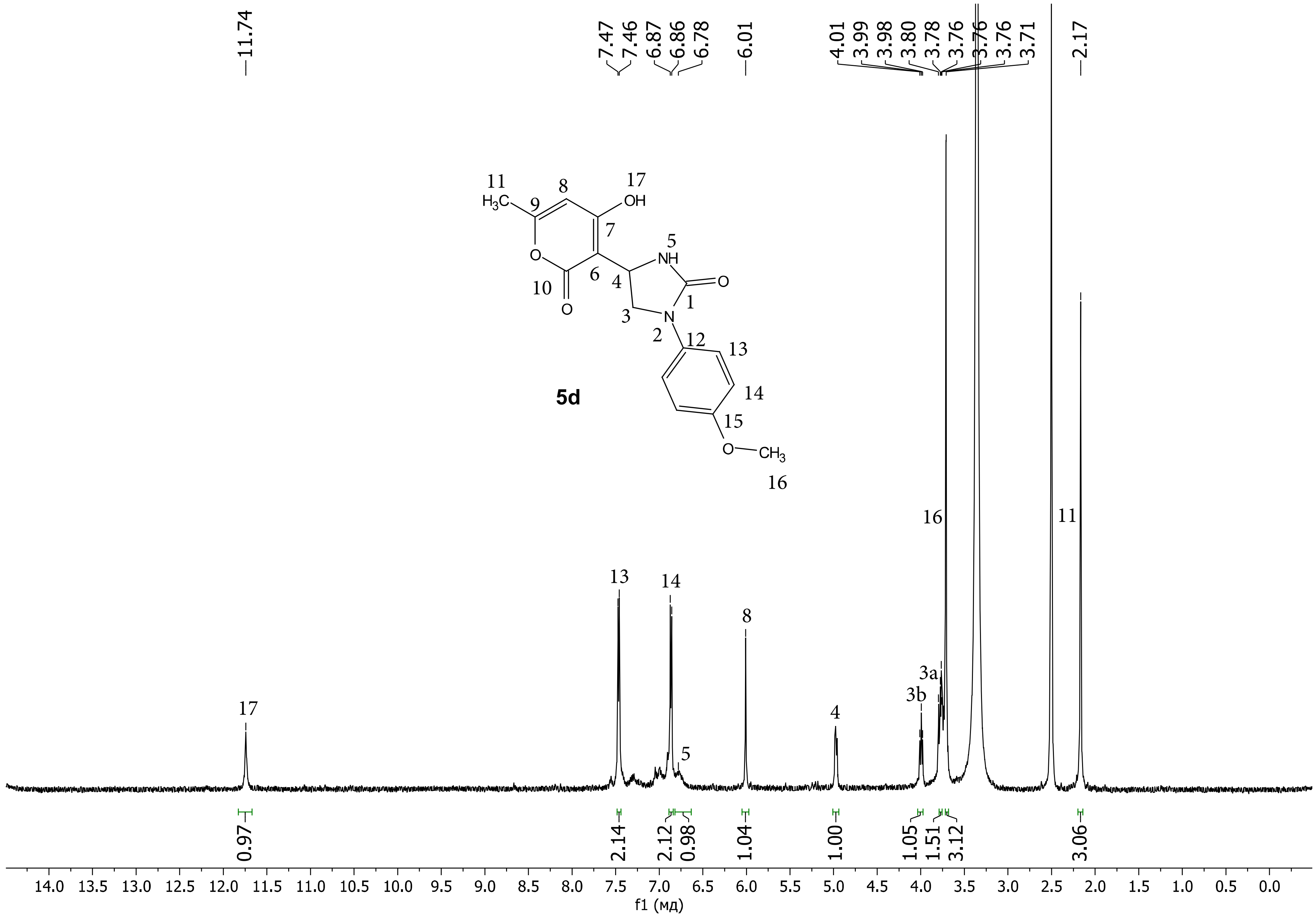

Figure S76. ^{1}H NMR spectrum (DMSO-d_6, 400MHz, 303K) of the compound **5d**

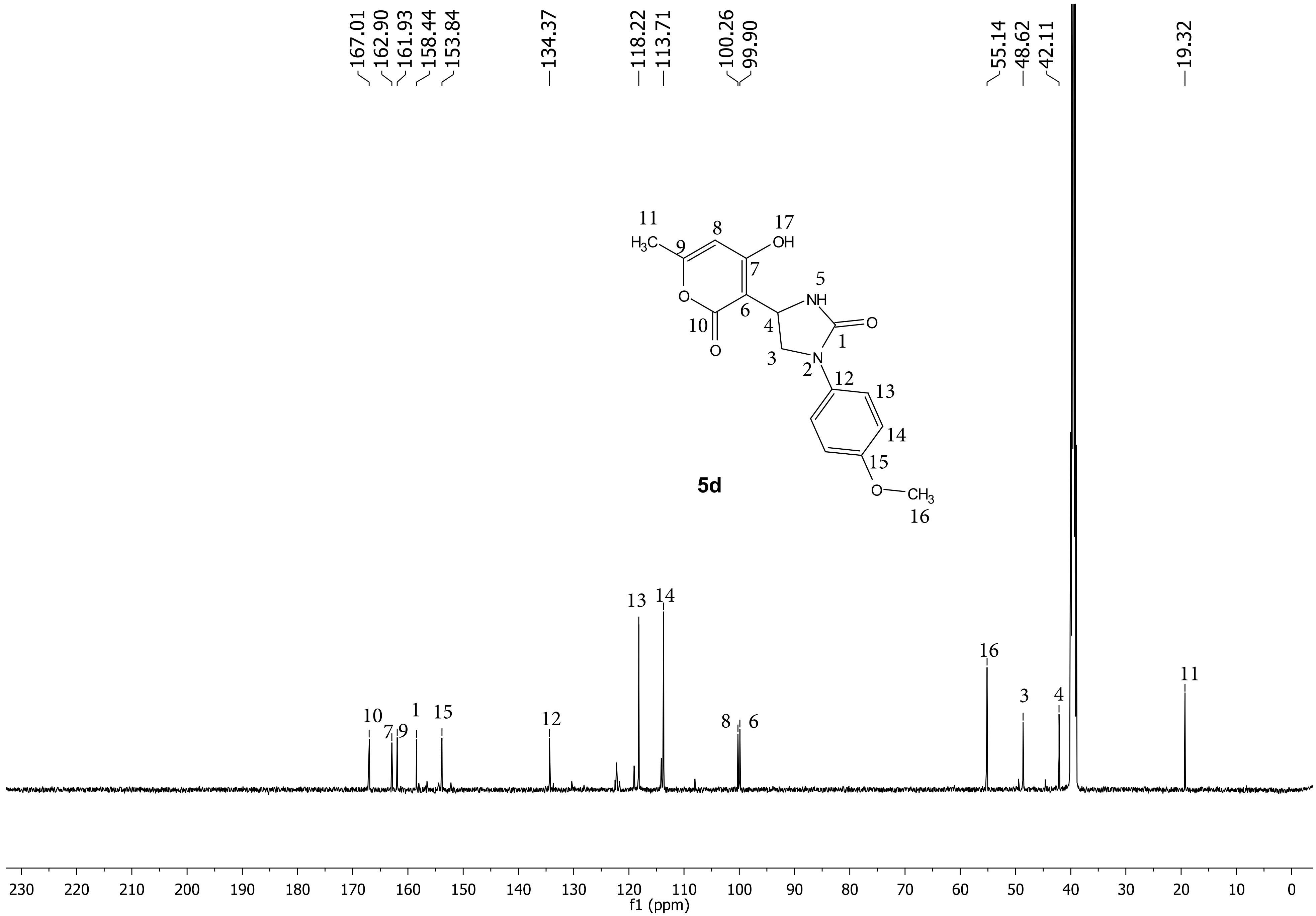

Figure S77. ^{13}C NMR spectrum (DMSO-d_6, 151MHz, 303K) of the compound **5d**

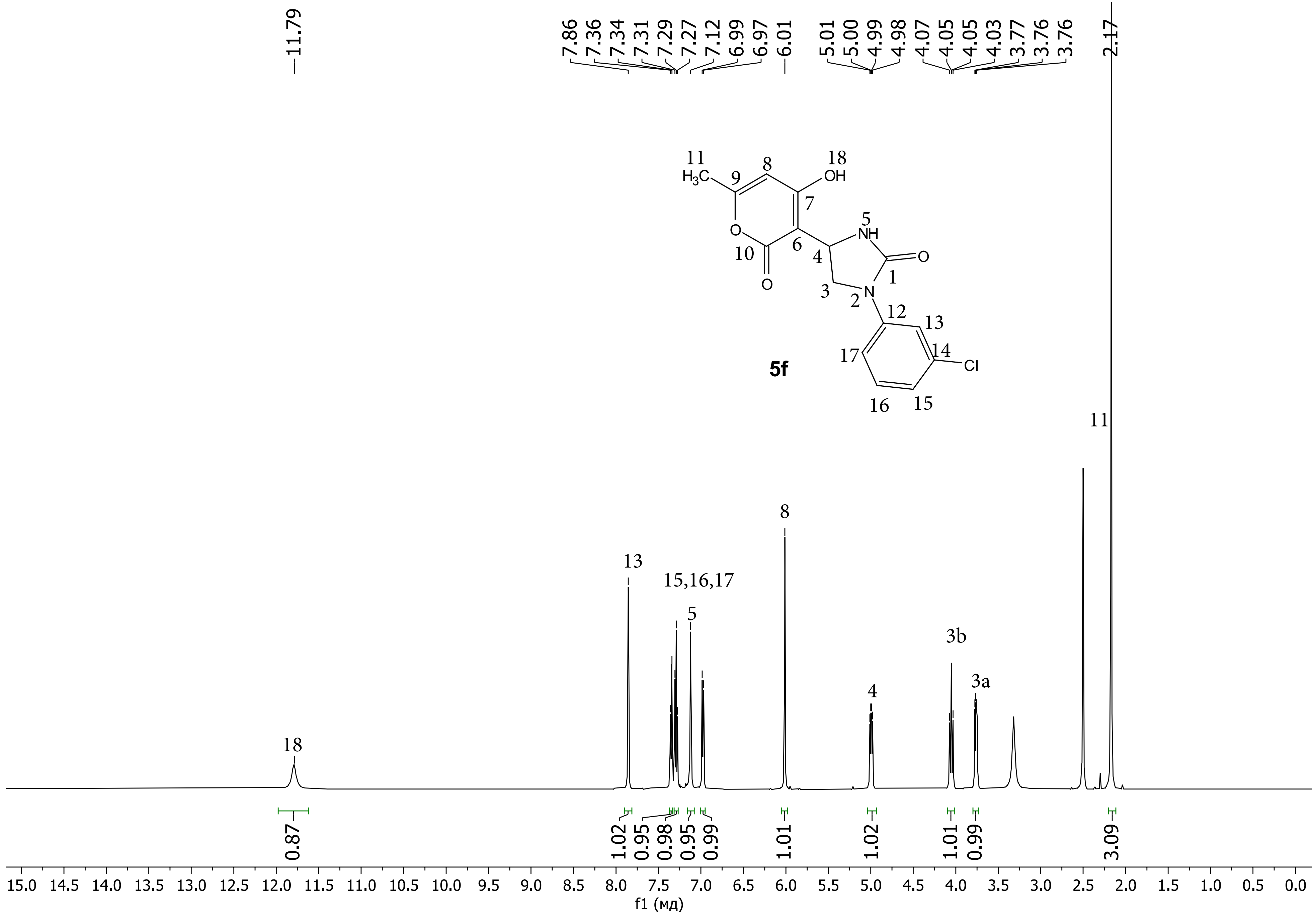

Figure S78. ^{1}H NMR spectrum (DMSO-d_6, 400MHz, 303K) of the compound **5f**

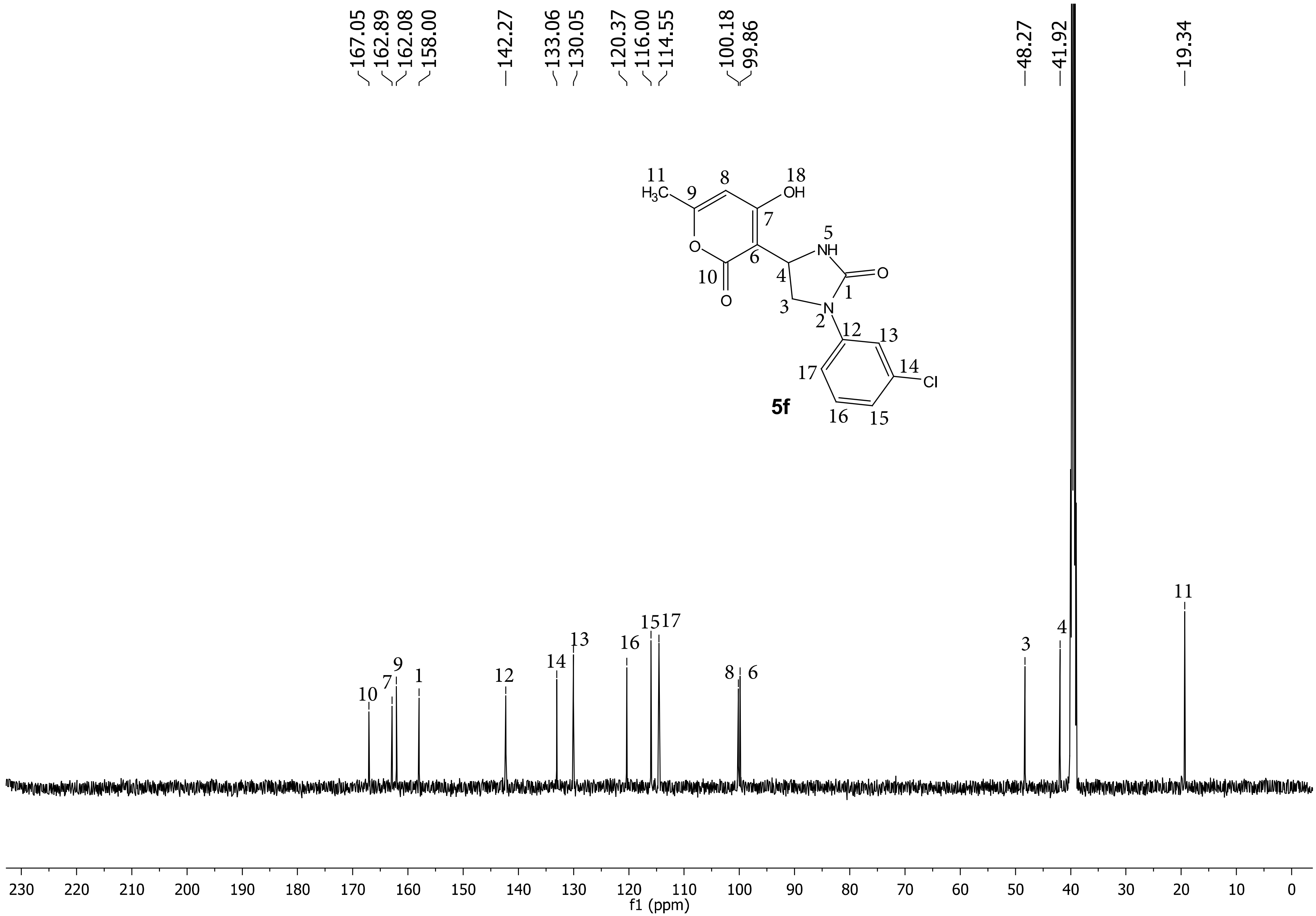

Figure S79. ^{13}C NMR spectrum (DMSO-d_6, 151MHz, 303K) of the compound **5f**

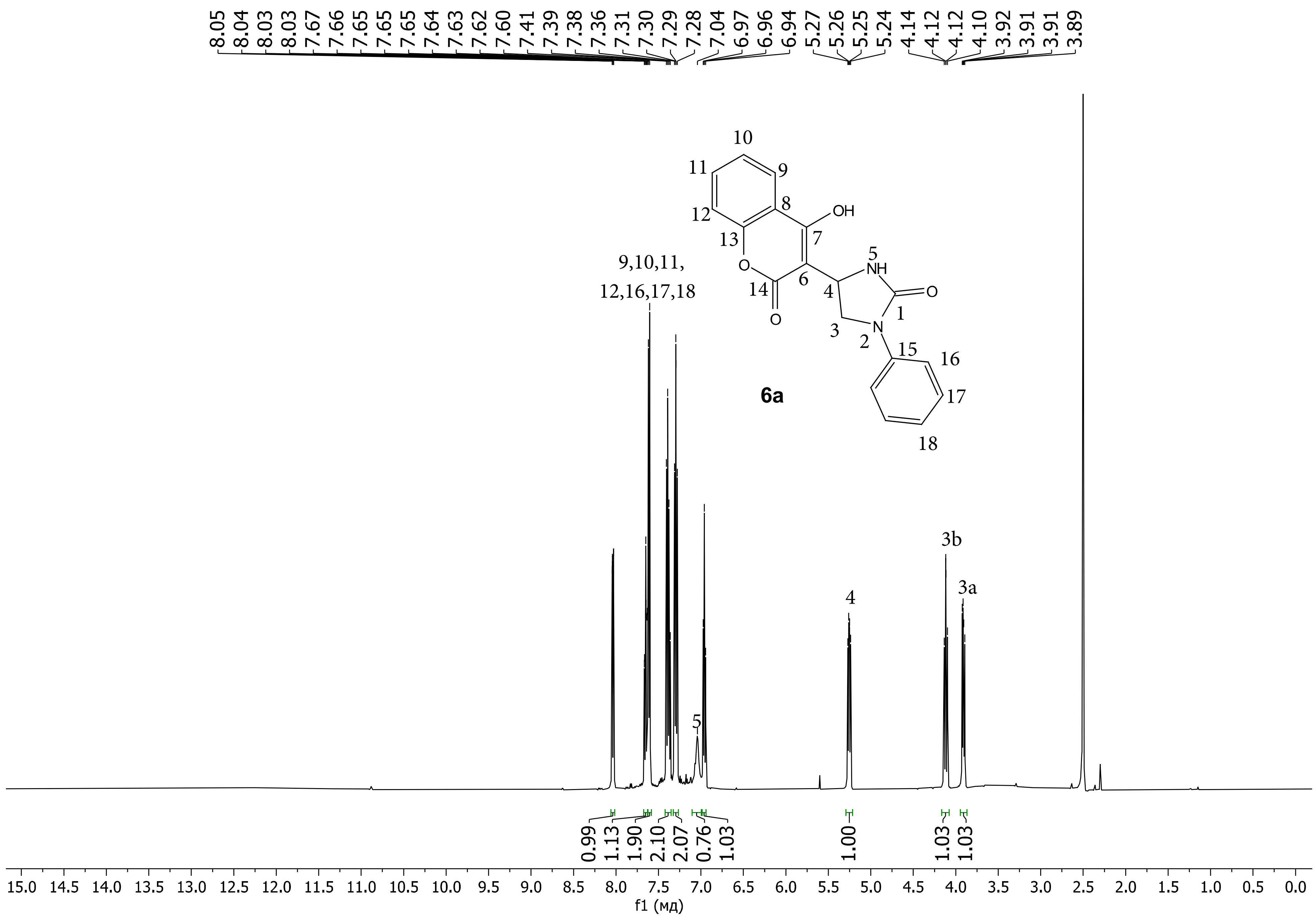

Figure S80. ^{1}H NMR spectrum (DMSO-d_6, 400MHz, 303K) of the compound **6a**

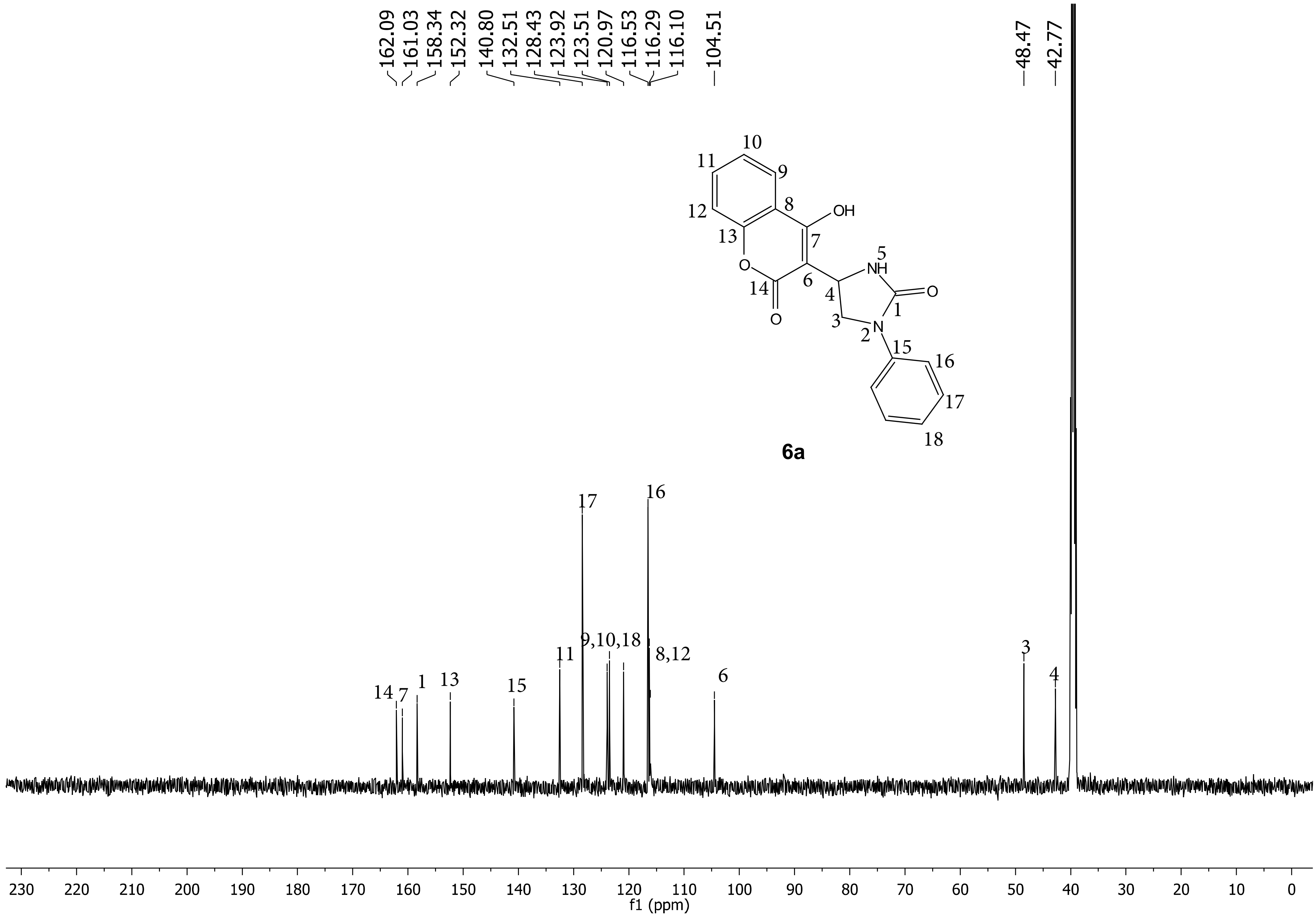

Figure S81. ^{13}C NMR spectrum (DMSO-d_6, 151MHz, 303K) of the compound **6a**

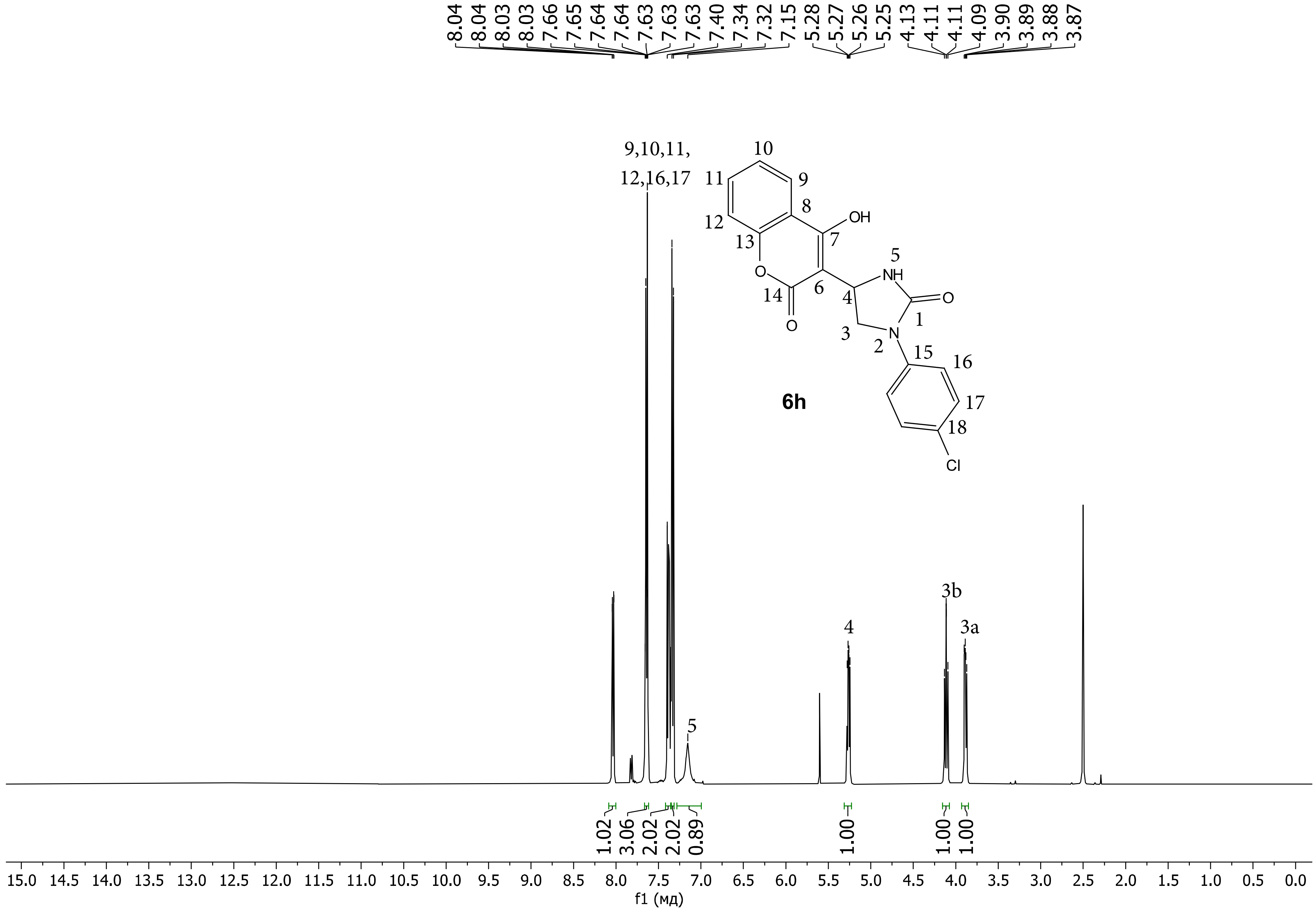

Figure S82. ^{1}H NMR spectrum (DMSO-d_6, 400MHz, 303K) of the compound **6h**

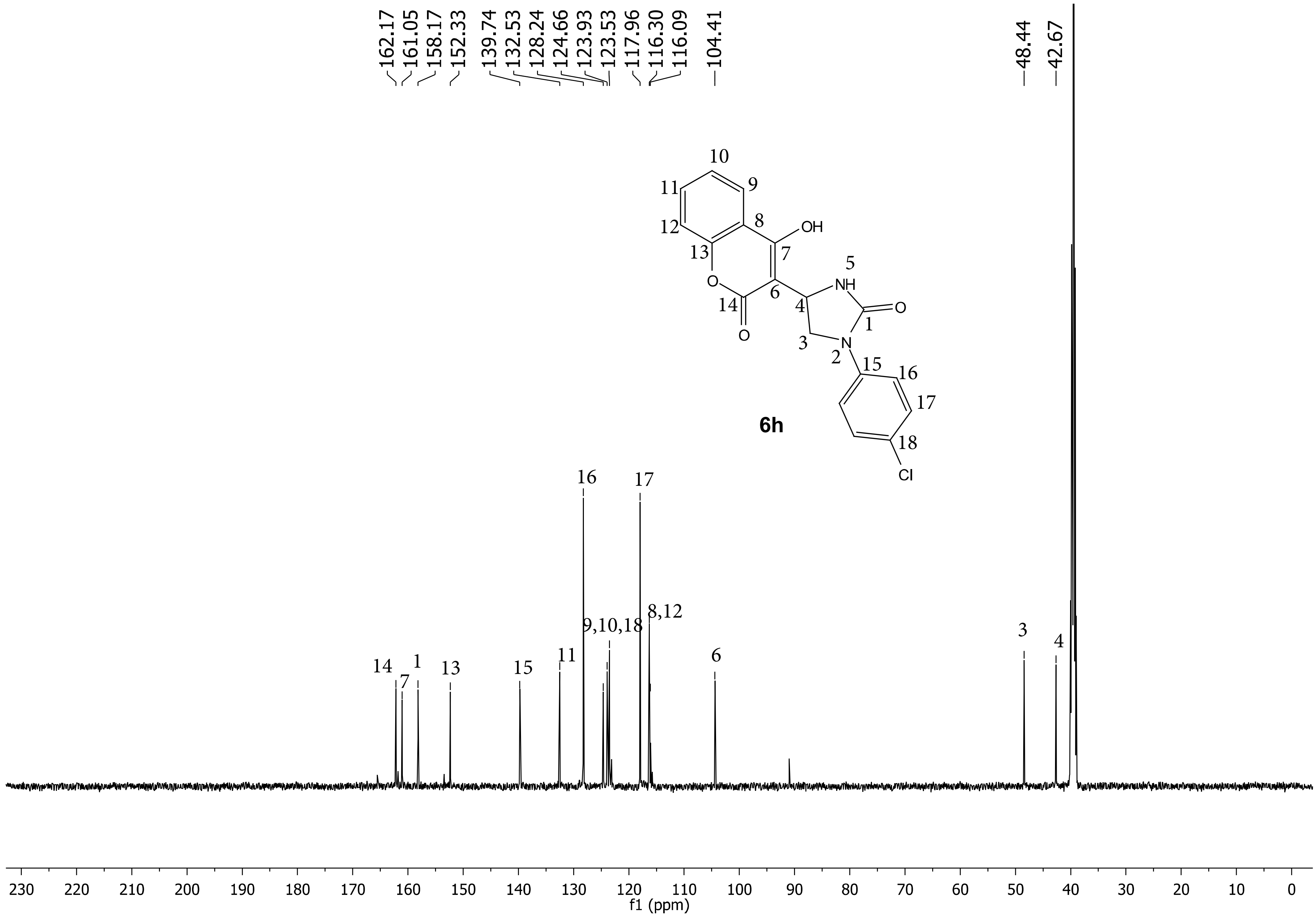

Figure S83. ^{13}C NMR spectrum (DMSO-d_6, 151MHz, 303K) of the compound **6h**

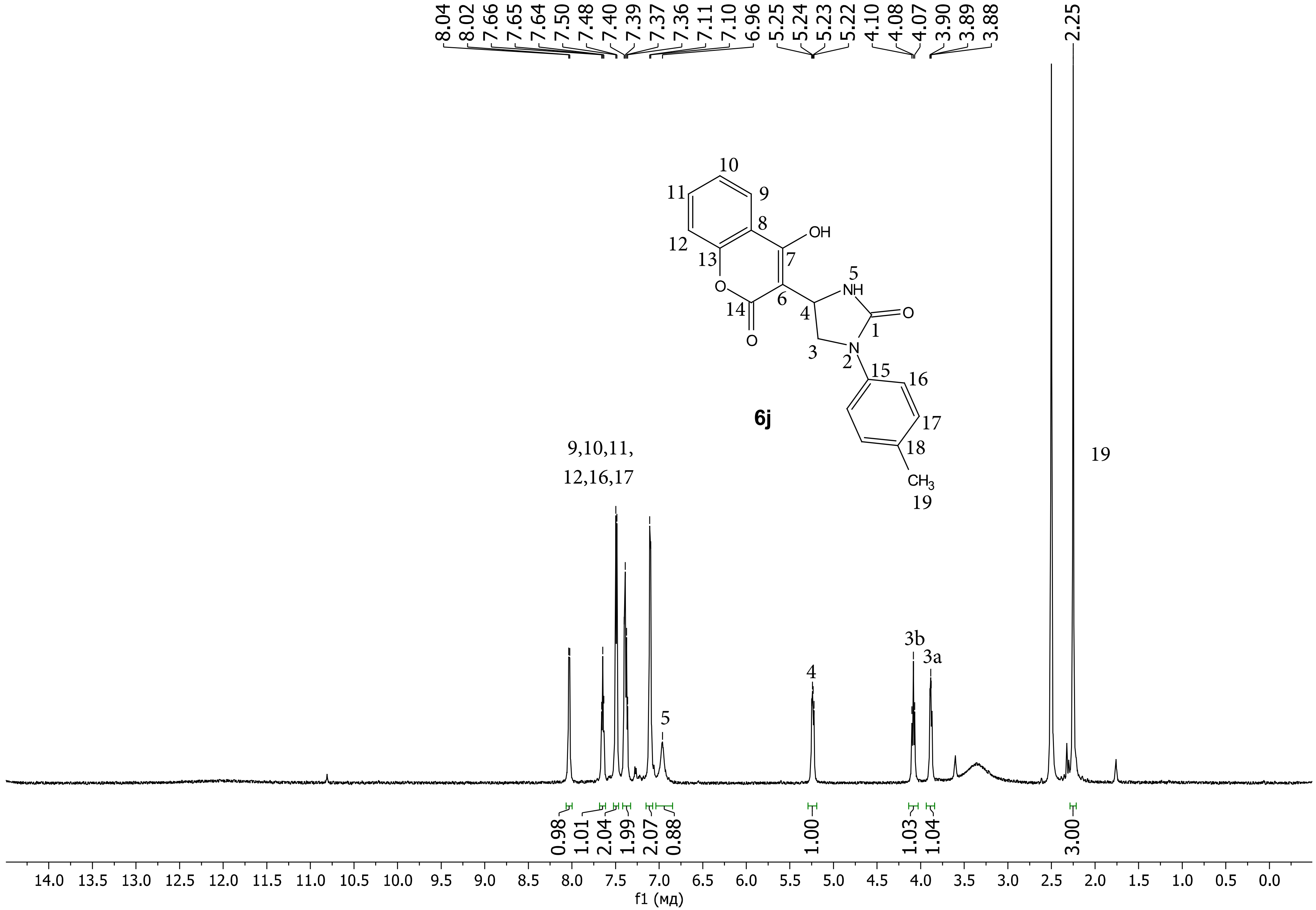

Figure S84. ^{1}H NMR spectrum (DMSO-d_6, 400MHz, 303K) of the compound **6j**

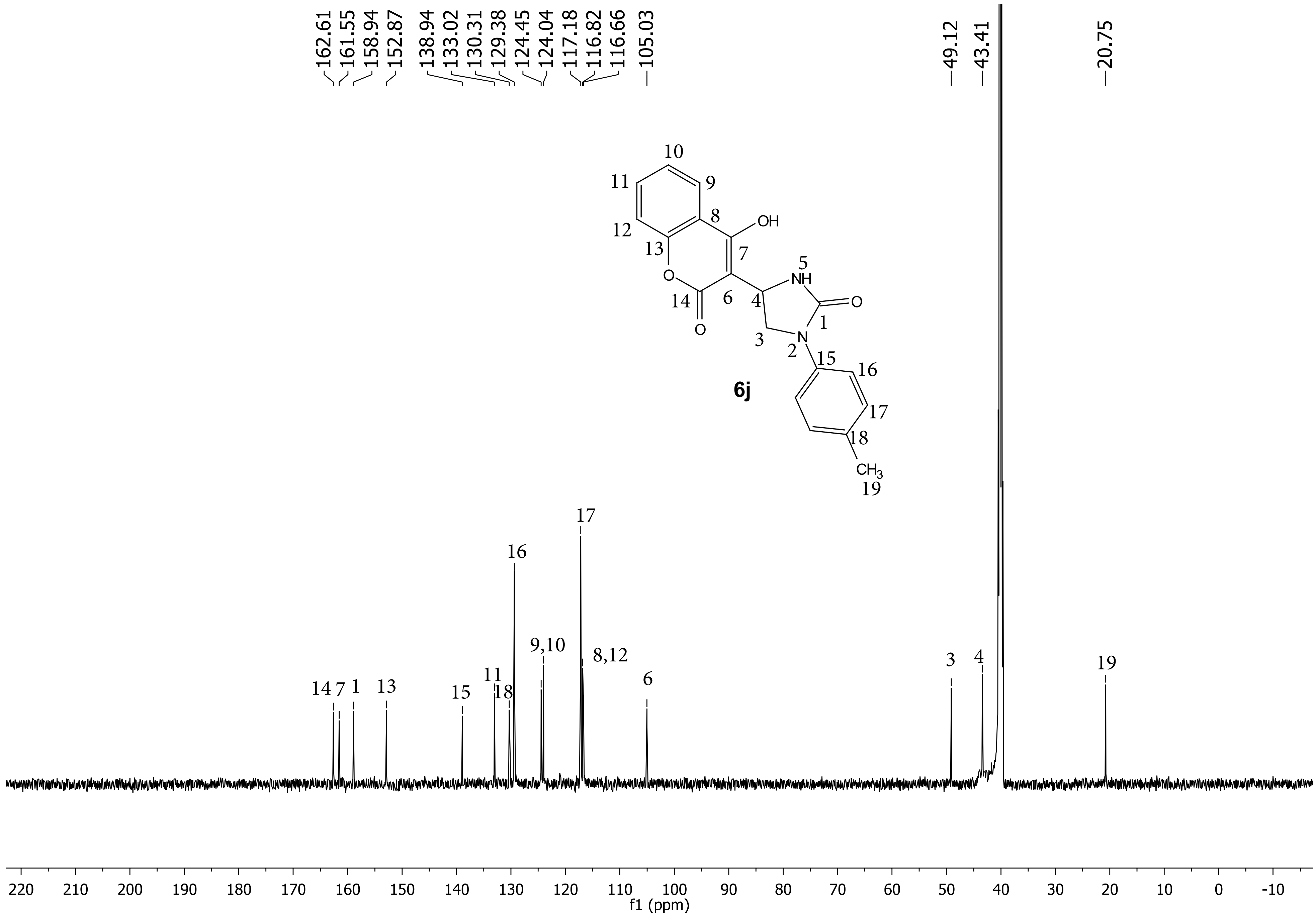

Figure S85. ^{13}C NMR spectrum (DMSO-d_6, 151MHz, 303K) of the compound **6j**

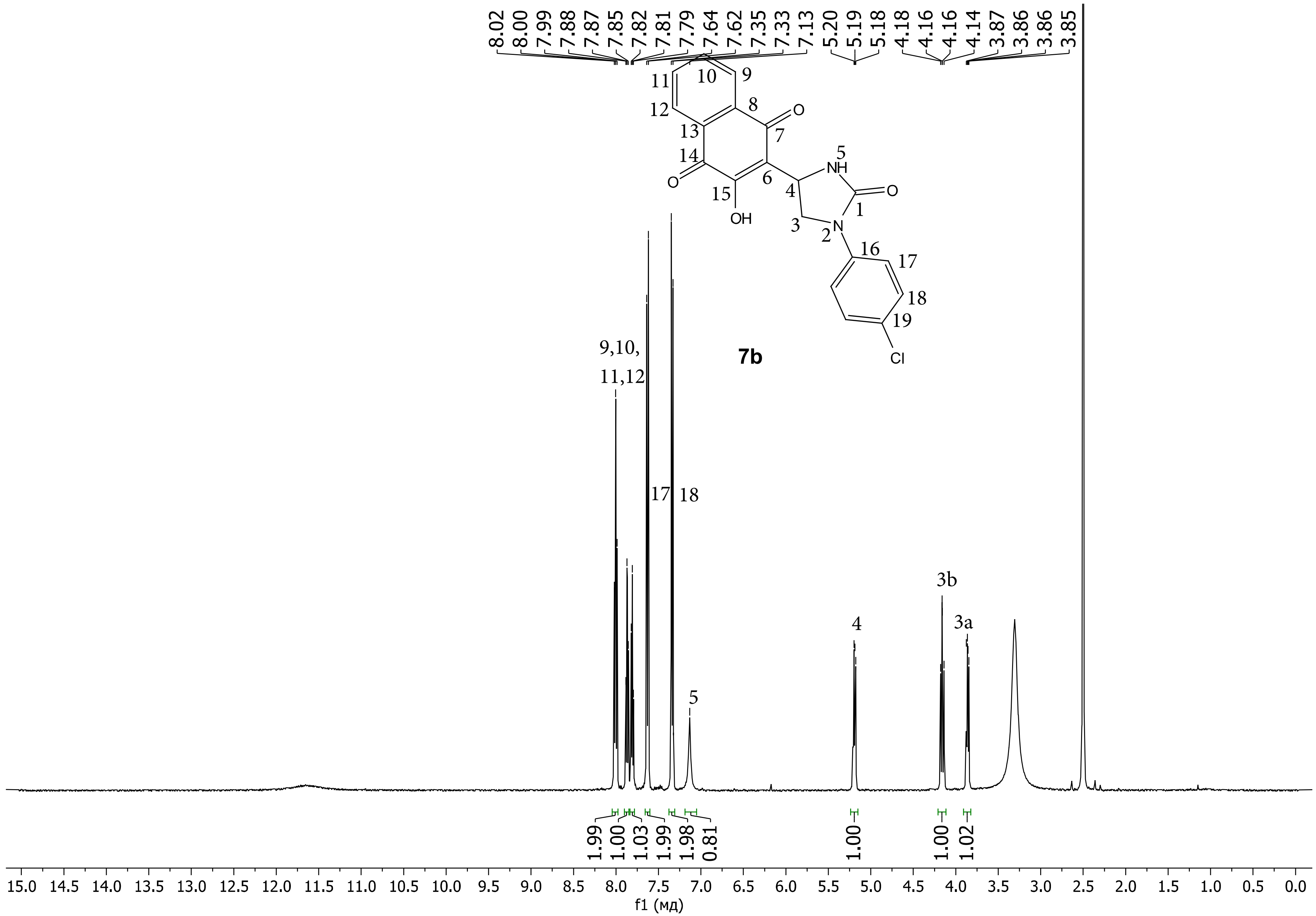

Figure S86. ^{1}H NMR spectrum (DMSO-d_6, 400MHz, 303K) of the compound **7b**

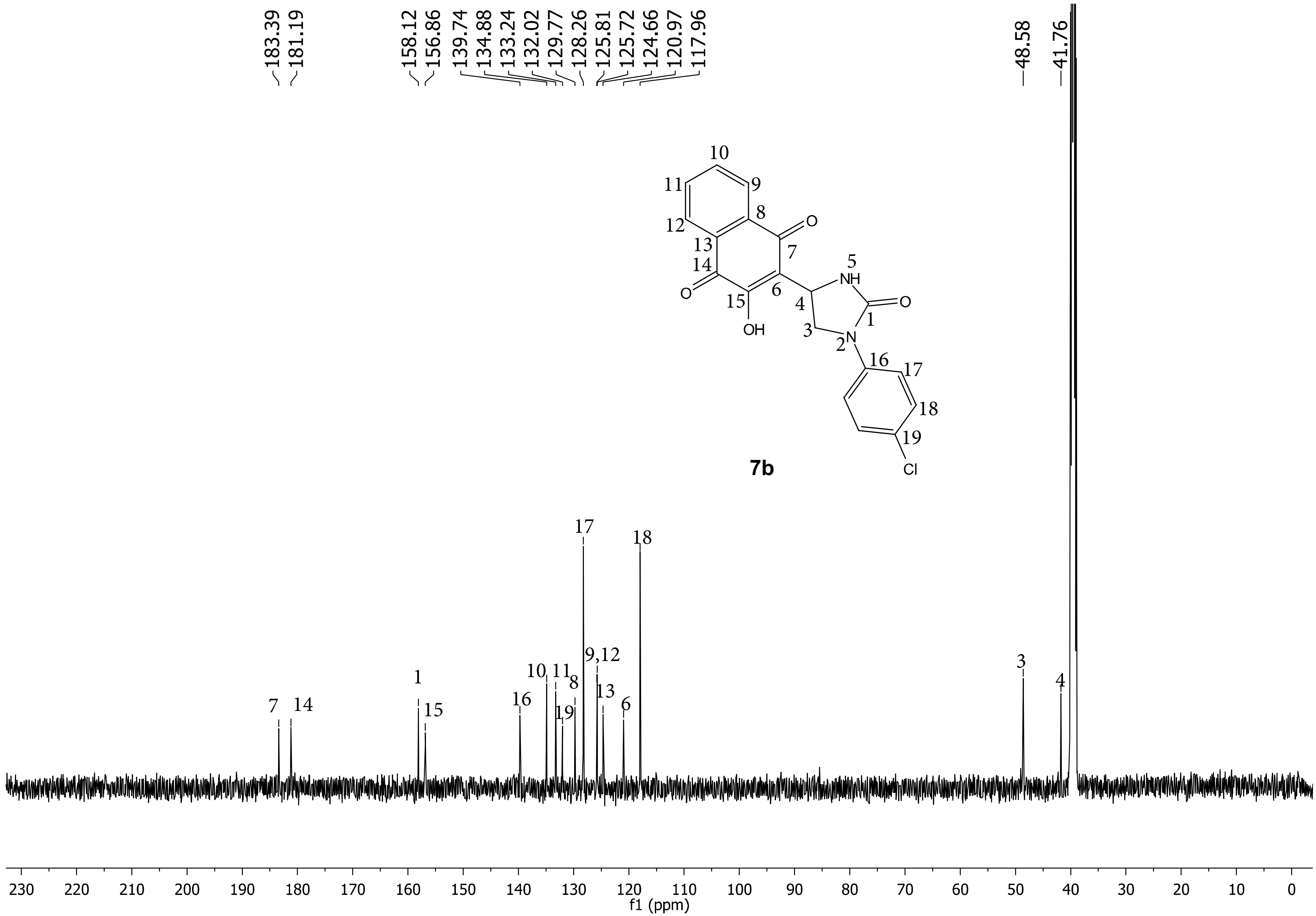

Figure S87. ^{13}C NMR spectrum (DMSO-d_6, 151MHz, 303K) of the compound **7b**

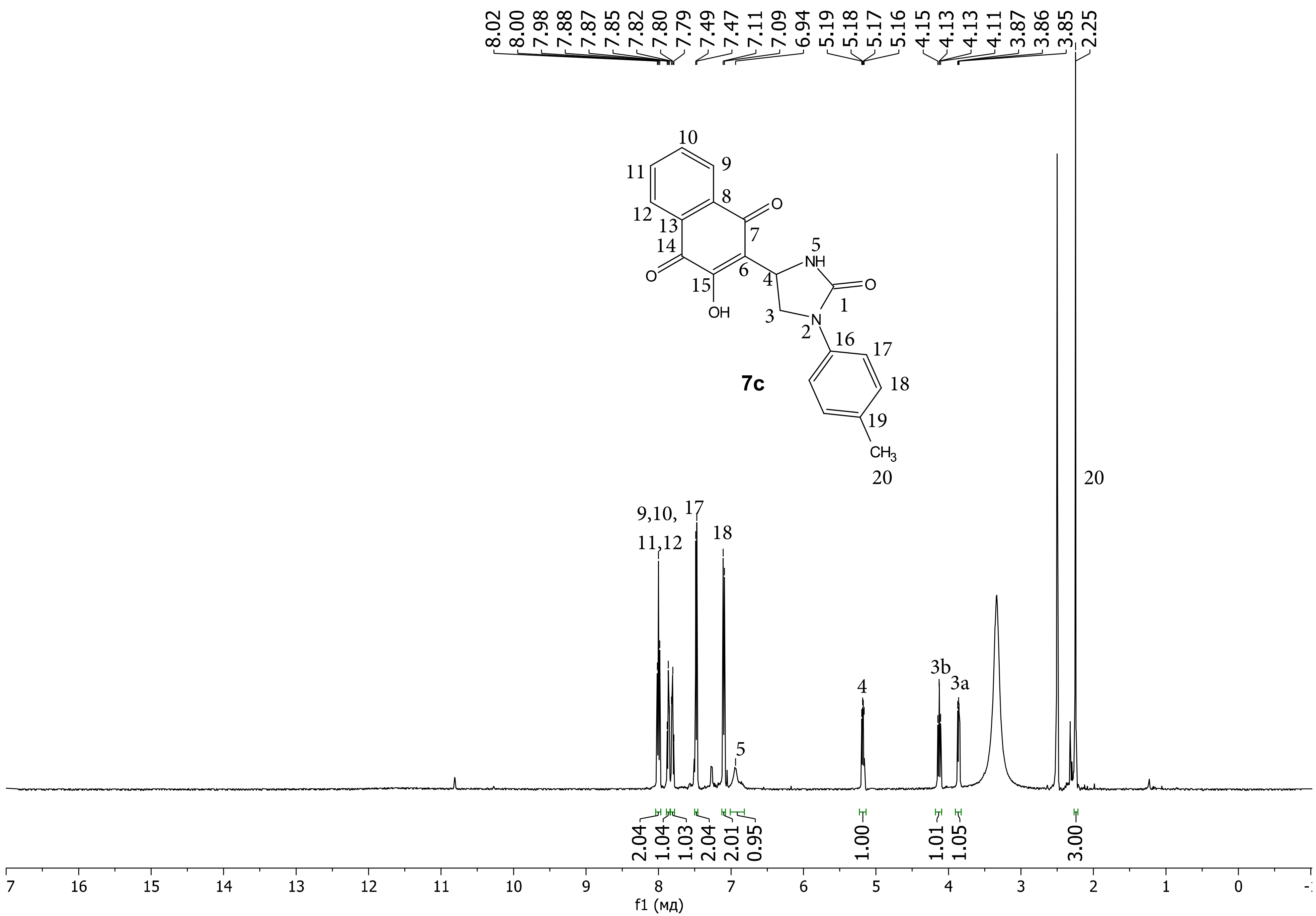

Figure S88. ^{1}H NMR spectrum (DMSO-d_6, 400MHz, 303K) of the compound **7c**

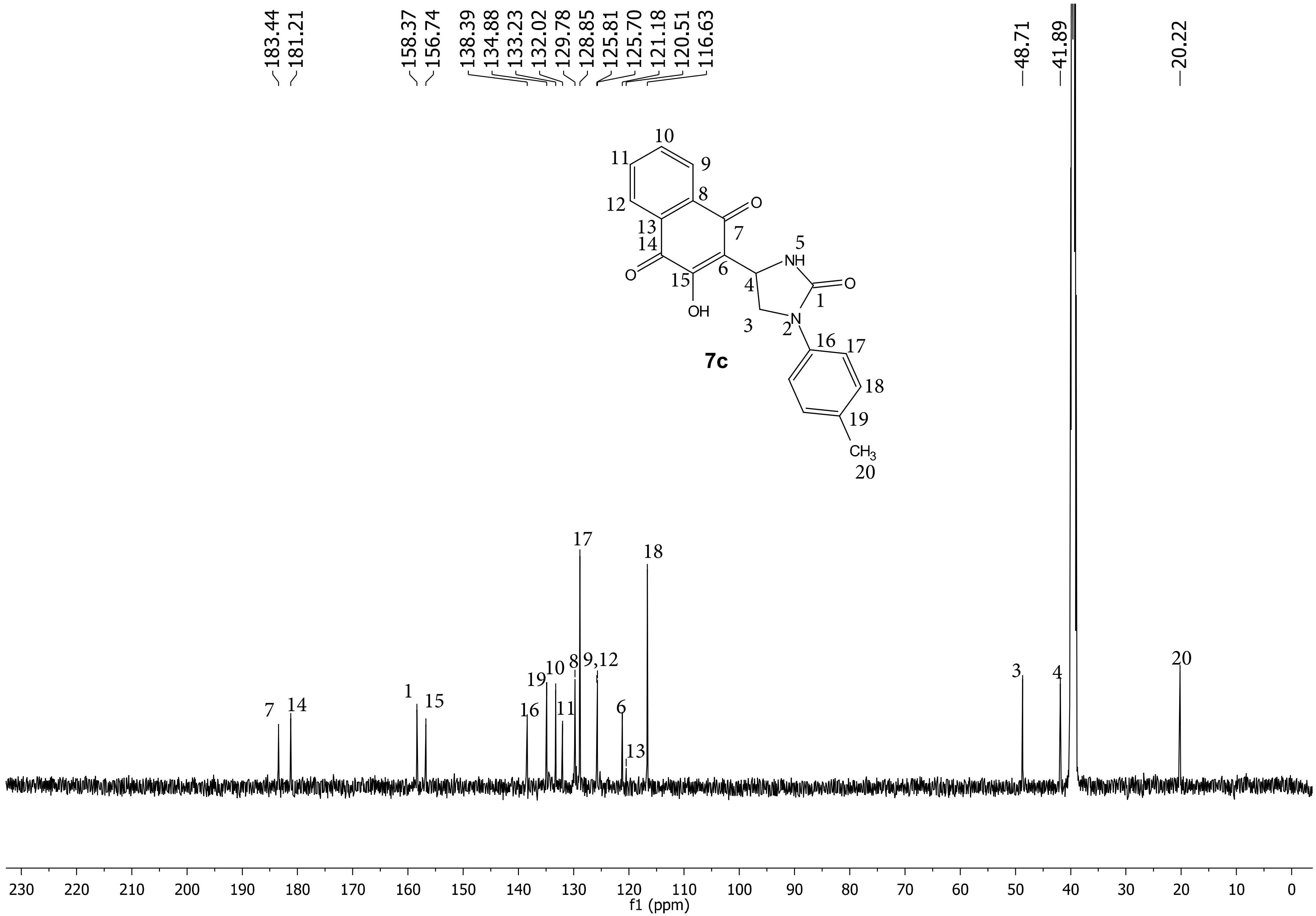

Figure S89. ^{13}C NMR spectrum (DMSO-d_6, 151MHz, 303K) of the compound **7c**

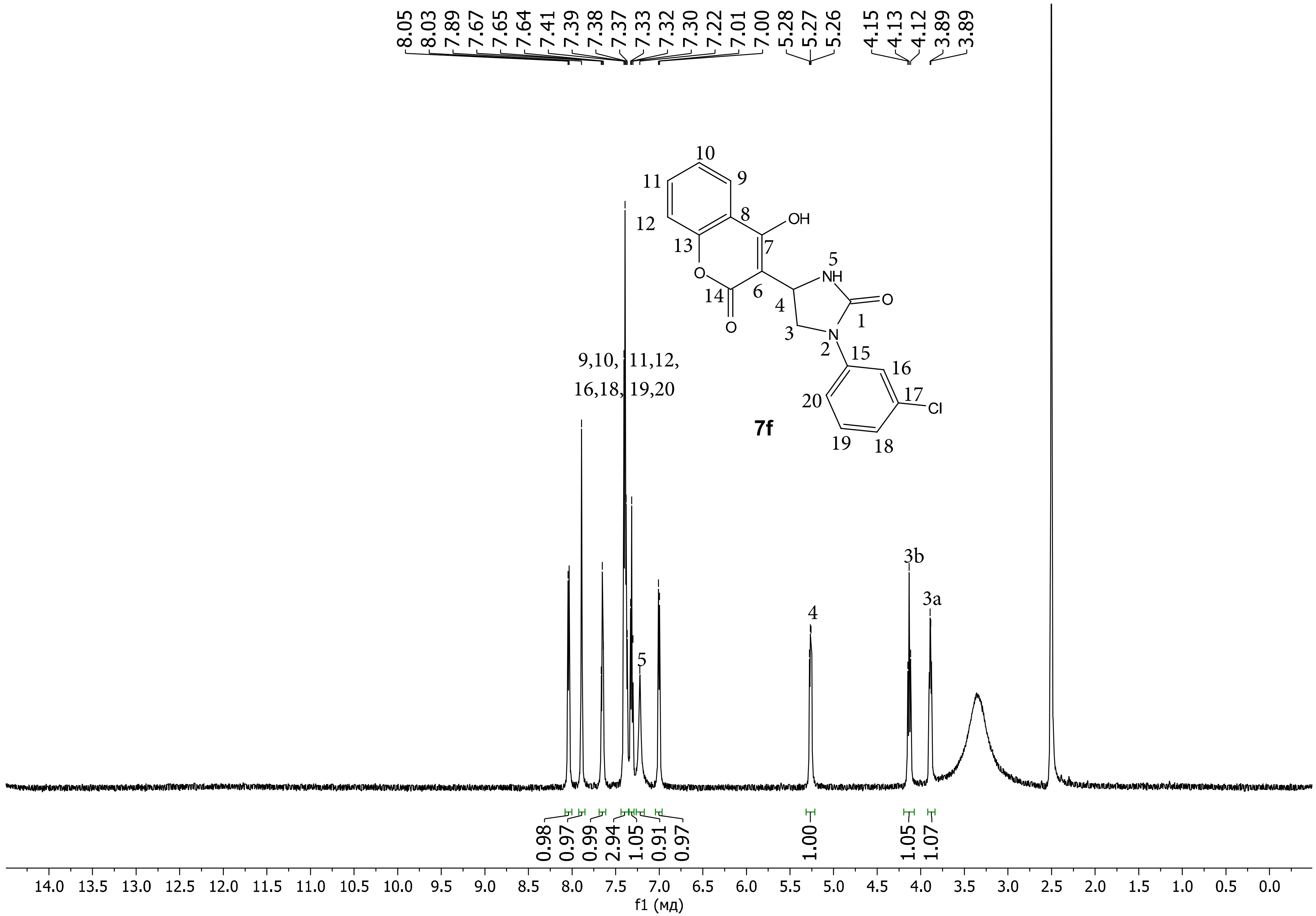

Figure S90. ^{1}H NMR spectrum (DMSO-d_6, 400MHz, 303K) of the compound **7f**

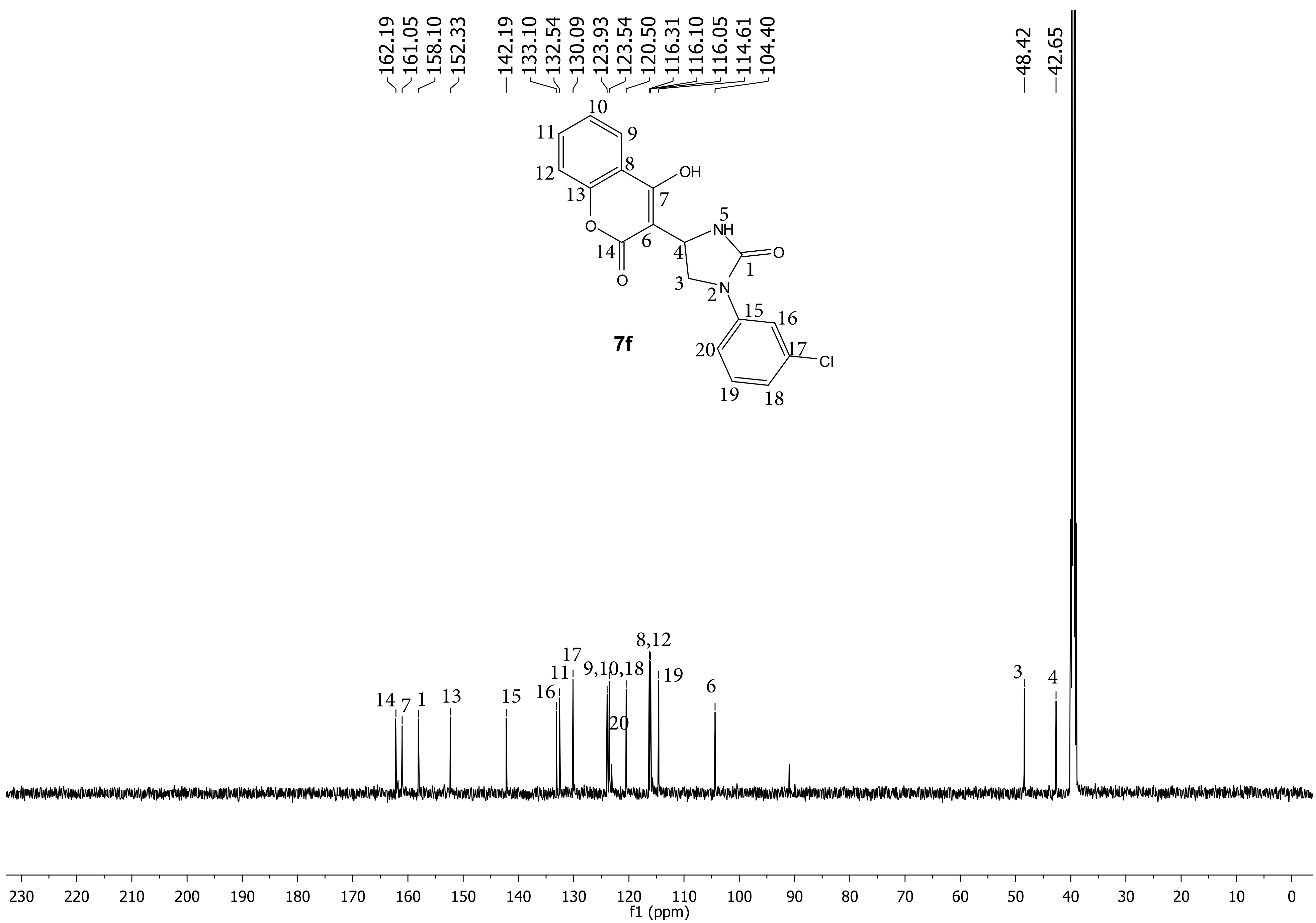

Figure S91. ^{13}C NMR spectrum (DMSO-d_6, 151MHz, 303K) of the compound **7f**

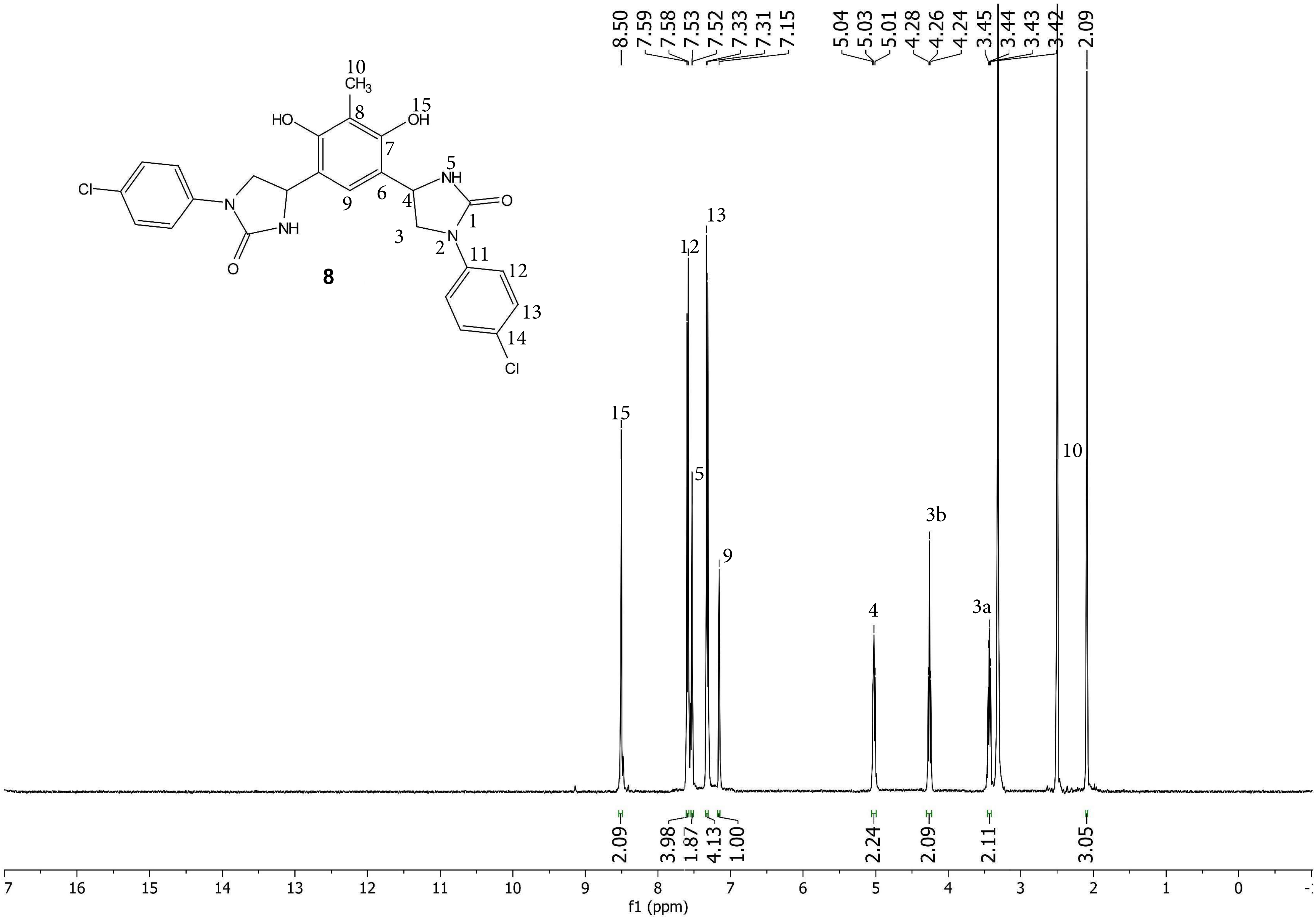

Figure S92. ^{1}H NMR spectrum (DMSO-d_6, 400MHz, 303K) of the compound **8**

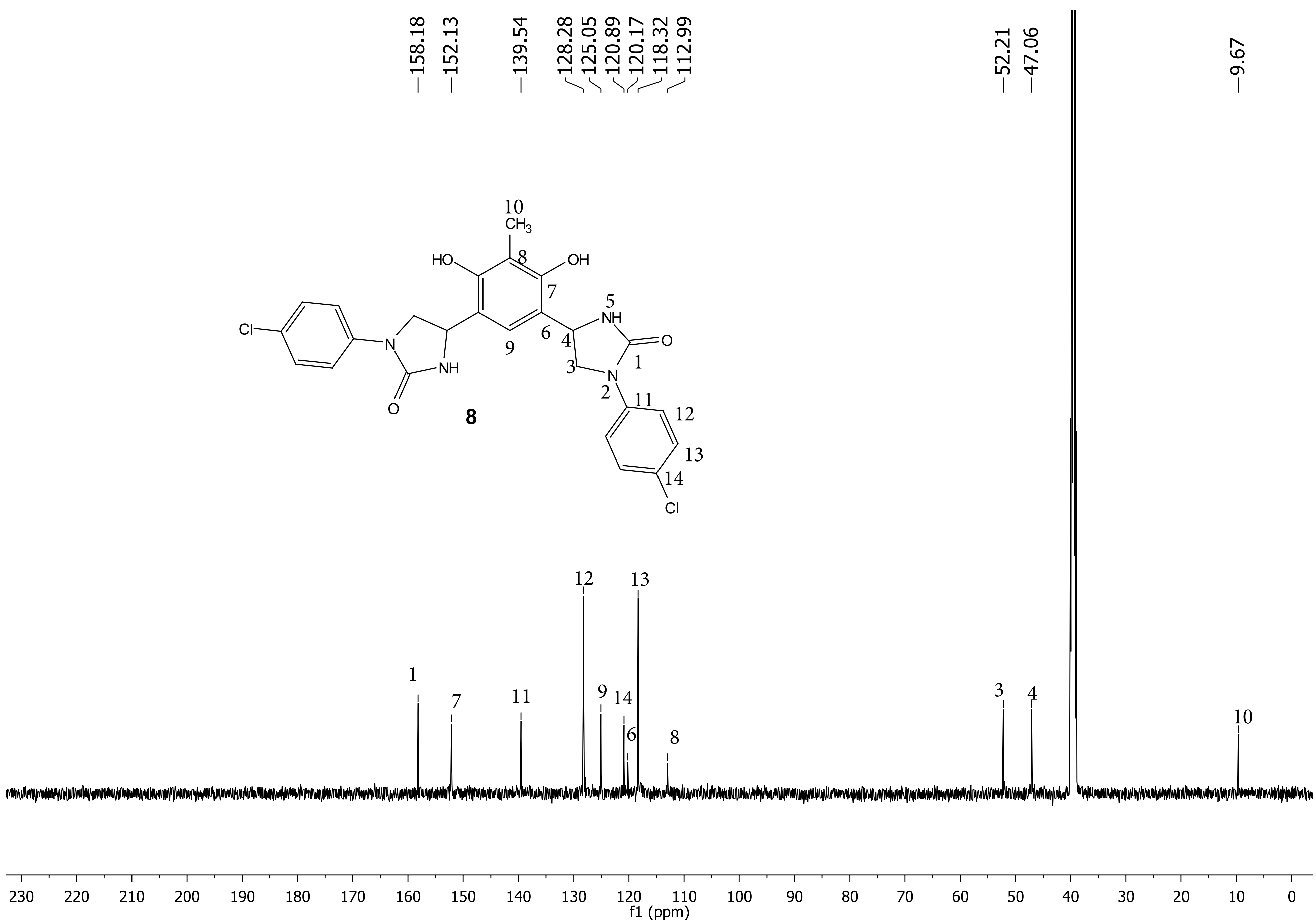

Figure S93. ^{13}C NMR spectrum (DMSO-d_6, 151MHz, 303K) of the compound **8**

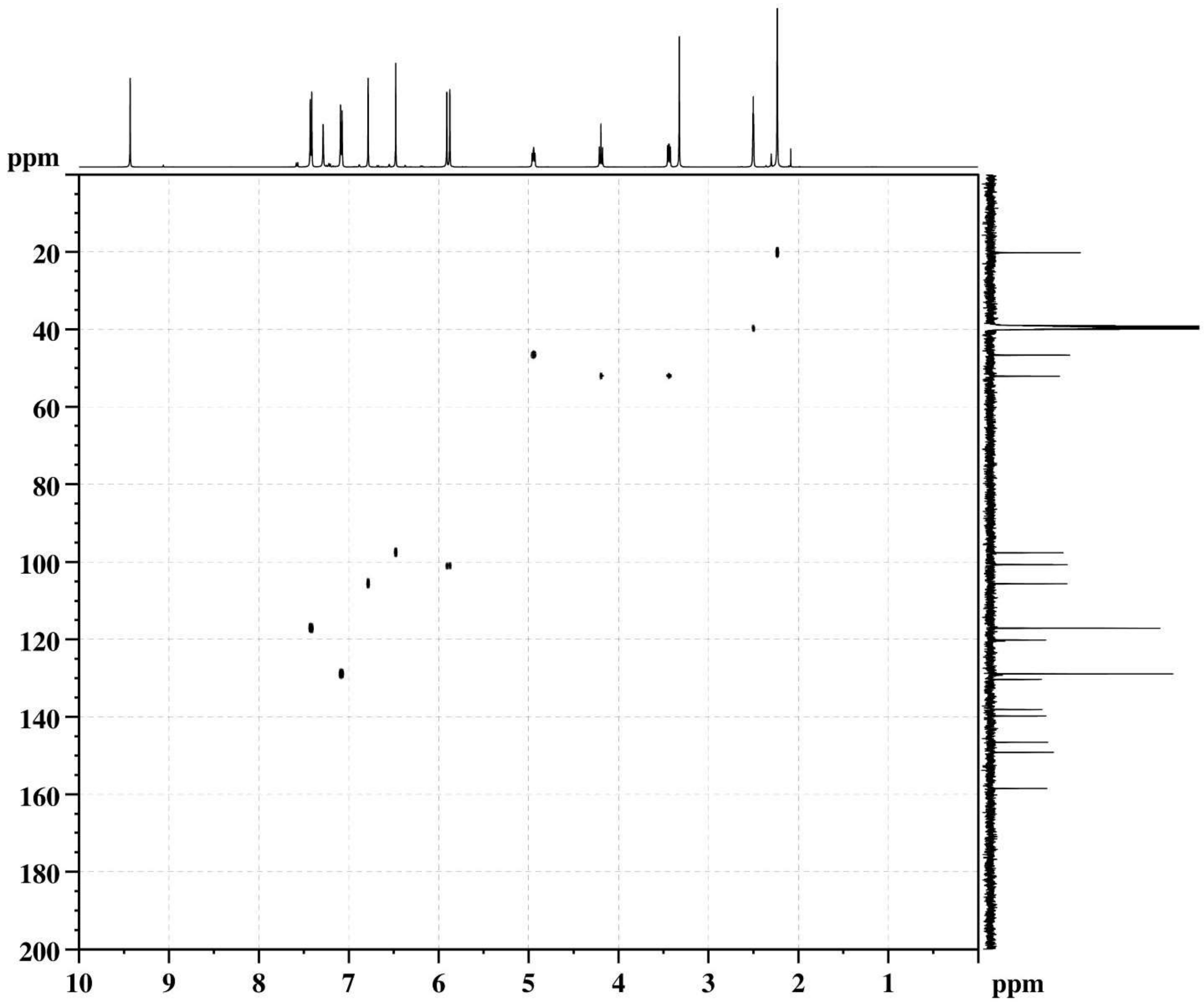

Figure S94. ^{1}H-^{13}C HSQC NMR spectrum of the compound **2c** (DMSO-d_6, 303K)

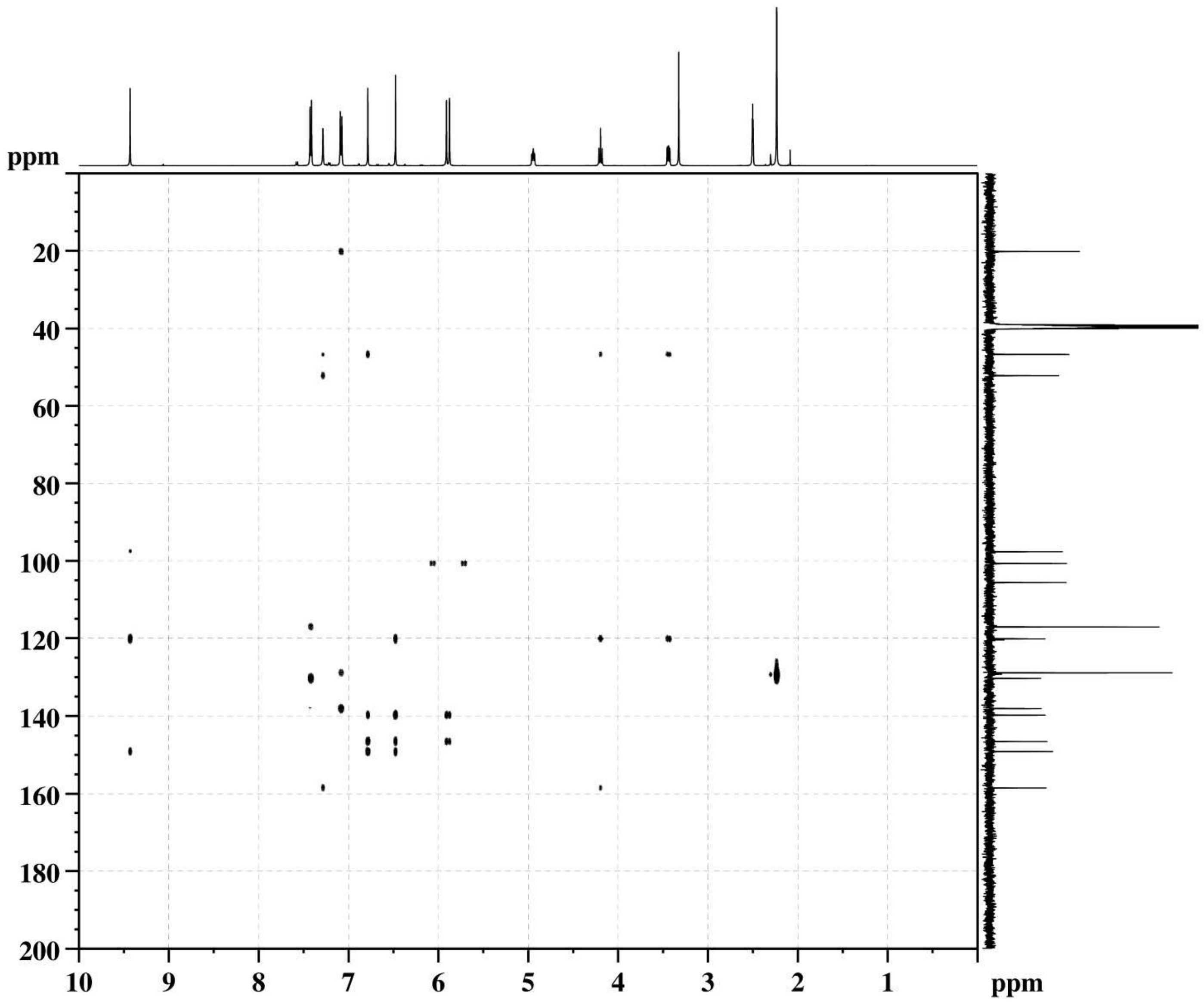

Figure S95. ^{1}H-^{13}C HMBC NMR spectrum of the compound **2c** (DMSO-d_6, 303K)

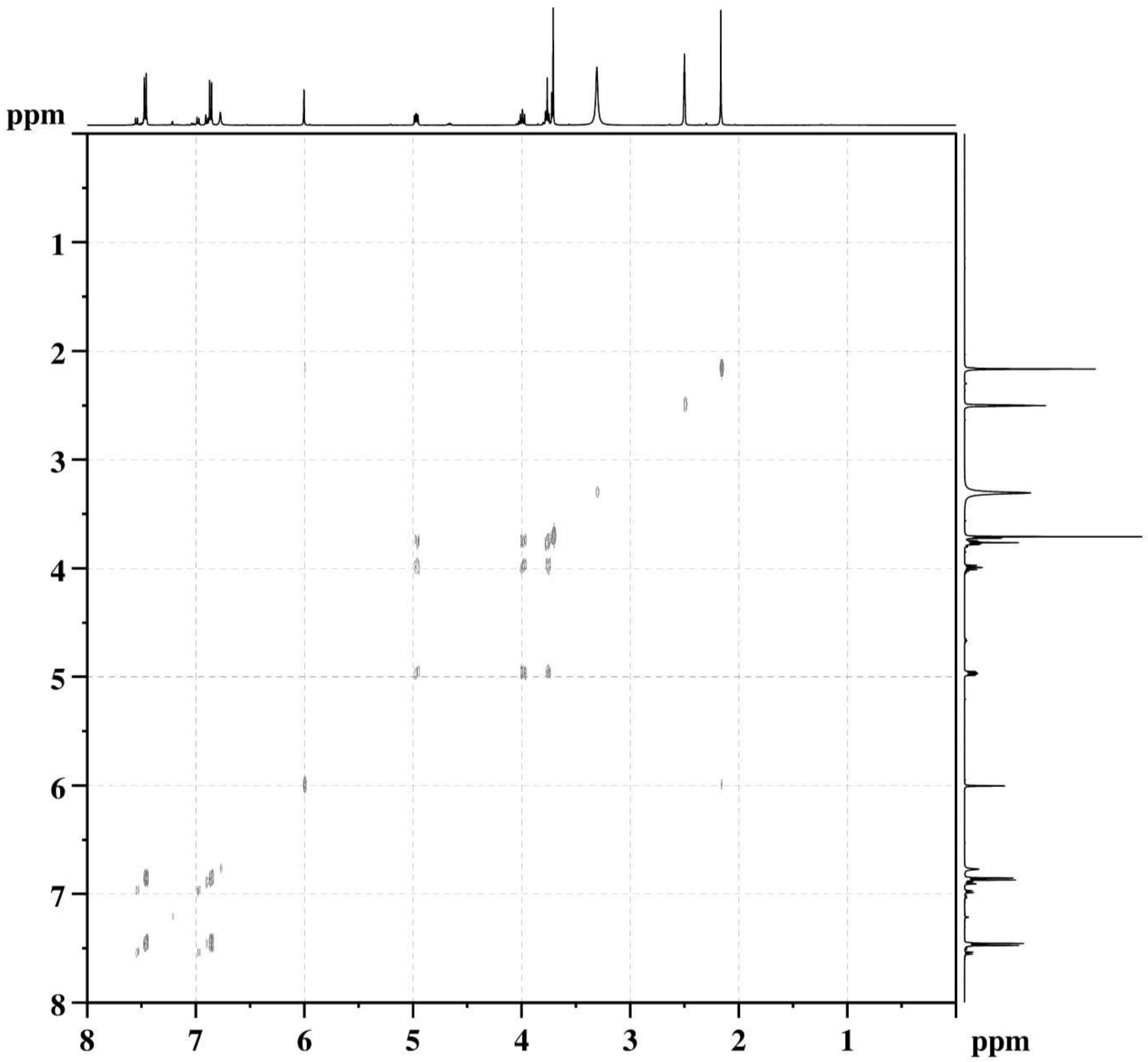

Figure S96. ^{1}H-^{1}H COSY NMR spectrum of the compound **5d** (DMSO-d_6, 303K)

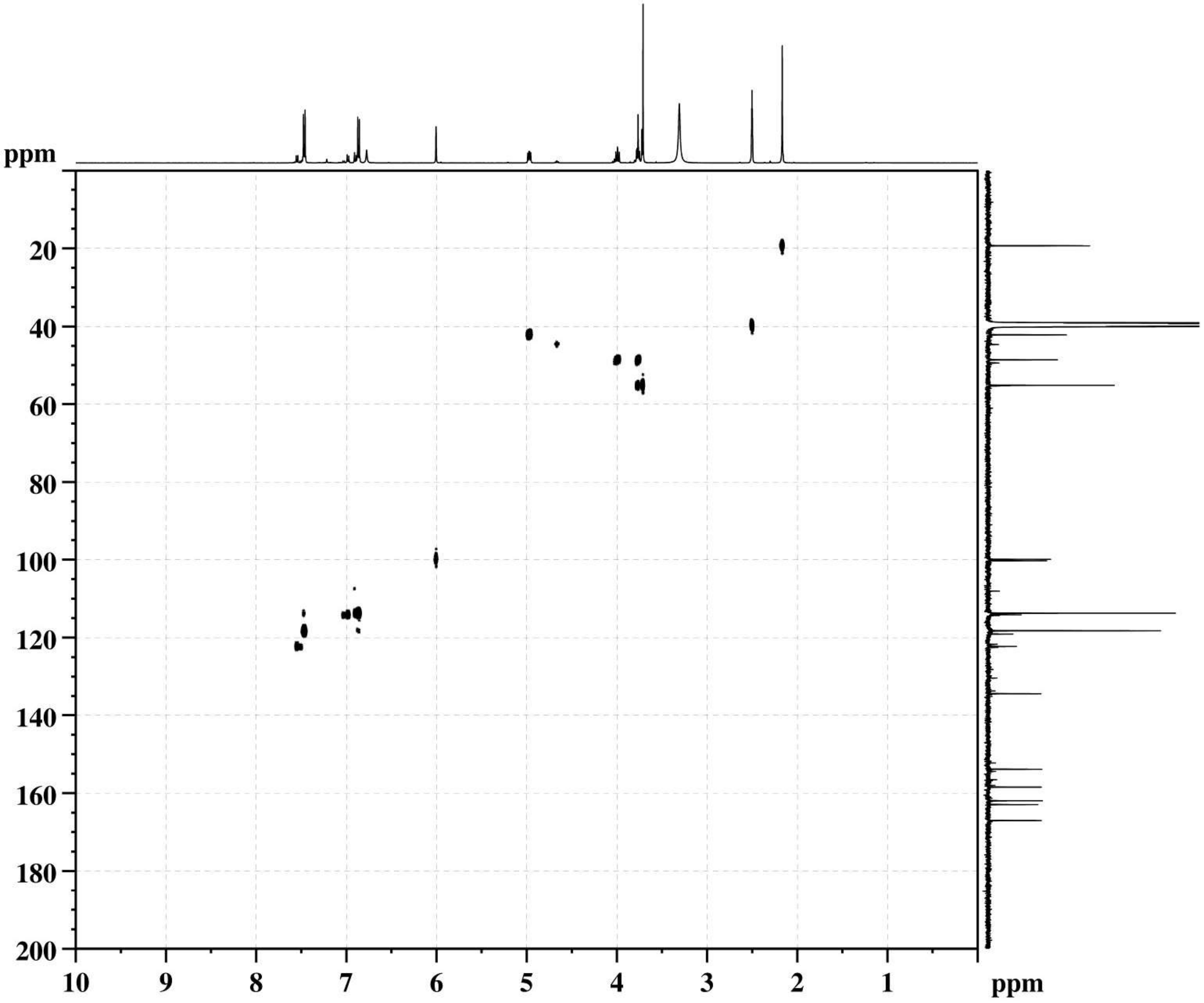

Figure S97. ^{1}H-^{13}C HSQC NMR spectrum of the compound **5d** (DMSO-d_6, 303K)

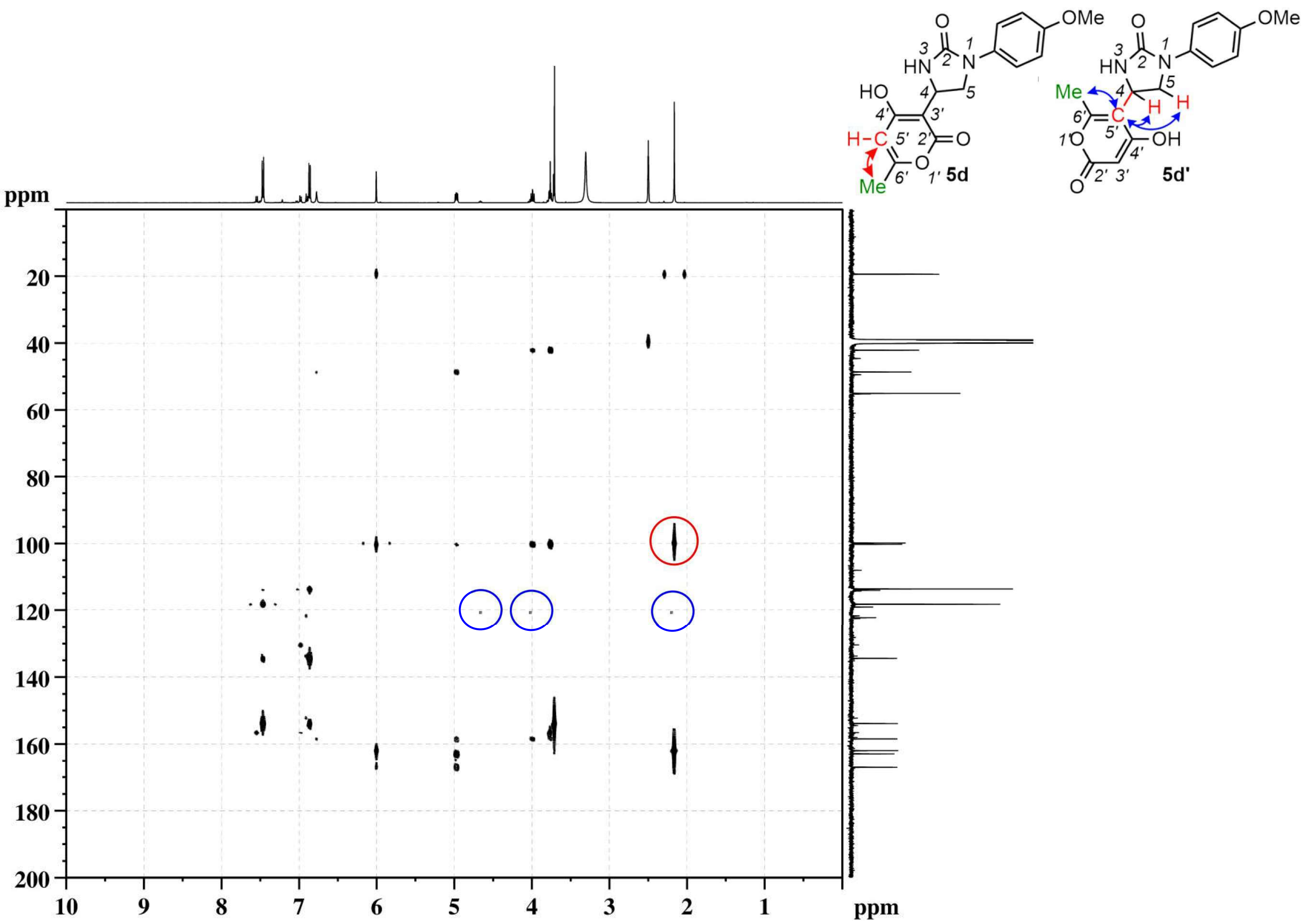

Figure S98. ^{1}H-^{13}C HMBC NMR spectrum of the compound **5d** (DMSO-d_6, 303K)
